# Synthesis of Novel Quinazolinone Analogues for Quorum Sensing Inhibition

**DOI:** 10.3390/antibiotics12071227

**Published:** 2023-07-24

**Authors:** Sahil Shandil, Tsz Tin Yu, Shekh Sabir, David StC. Black, Naresh Kumar

**Affiliations:** School of Chemistry, The University of New South Wales, Sydney, NSW 2052, Australiad.black@unsw.edu.au (D.S.B.)

**Keywords:** quorum sensing inhibition, *pqs*, *Pseudomonas aeruginosa*, quinazolinone

## Abstract

As bacteria continue to develop resistance mechanisms against antimicrobials, an alternative method to tackle this global concern must be developed. As the *pqs* system is the most well-known and responsible for biofilm and pyocyanin production, quinazolinone inhibitors of the *pqs* system in *P. aeruginosa* were developed. Molecular docking following a rationalised medicinal chemistry approach was adopted to design these analogues. An analysis of docking data suggested that compound **6b** could bind with the key residues in the ligand binding domain of PqsR in a similar fashion to the known antagonist M64. The modification of cyclic groups at the 3-position of the quinazolinone core, the introduction of a halogen at the aromatic core and the modification of the terminal group with aromatic and aliphatic chains were investigated to guide the synthesis of a library of 16 quinazolinone analogues. All quinazolinone analogues were tested in vitro for *pqs* inhibition, with the most active compounds **6b** and **6e** being tested for biofilm and growth inhibition in *P. aeruginosa* (PAO1). Compound **6b** displayed the highest *pqs* inhibitory activity (73.4%, 72.1% and 53.7% at 100, 50 and 25 µM, respectively) with no bacterial growth inhibition. However, compounds **6b** and **6e** only inhibited biofilm formation by 10% and 5%, respectively.

## 1. Introduction

In order to minimise the development of antibiotic resistance, research has shifted towards new classes of antimicrobial agents that target quorum sensing (QS), rather than cell viability like conventional antibiotics [1,2,3]. QS is a cell-signalling mechanism that allows bacteria to communicate with each other, causing a population of unicellular bacteria to behave similarly to a multicellular organism [2,3]. As the population density of bacteria grows, small molecules known as autoinducers (AIs) that regulate the expression of virulent genes are produced [2,3]. These AIs are produced intracellularly before being released actively or passively into the extracellular environment [3]. Once the concentration threshold of AIs is reached (known as a ‘quorum’), the signalling molecules can be recognised by the cognate receptors [3]. This results in the activation of signal transduction pathways that regulate a wide range of activities such as biofilm maturation, virulence factor production, secondary metabolite production and antibiotic resistance [2,3].

QS has become an emerging drug discovery target as it provides an avenue to prevent virulence and biofilm maturation [2,3,4]. As QS inhibitors (QSIs) disrupt bacterial communication through competitive inhibition instead of targeting cell viability, QSIs impose a less selective pressure on bacteria compared to traditional antibiotics, which reduces the chance of bacteria developing resistance against QSIs [4]. When used in conjunction with growth inhibitory antibiotics, QSIs are capable of combatting against biofilm formation and the virulence of bacteria while simultaneously reducing the associated antibiotic resistance [4].

In 2013, the U.S. Center for Disease Control and Prevention stated that Gram-negative bacteria accounted for a large proportion of resistance threats [5]. In particular, the Gram-negative bacterium *Pseudomonas aeruginosa* has been identified as a multidrug-resistant species that plays a crucial part in numerous diseases and infections [6,7]. *P. aeruginosa* has three major interconnected QS systems: *las, rhl* and *pqs* [7,8]. The *Pseudomonas* quinolone signal (*pqs*) system is known to be the most clinically relevant target as the biosynthesis and signal transduction pathways are now well-known and has proven to play a significant role in virulence and biofilm formation [7,8]. Furthermore, crystal structures for the essential components of the *pqs* system, such as PqsR, have been determined, making it an attractive target for QSIs [7]. Conversely, there has not been sufficient published work on the *Rhl* system to validate it as a possible drug discovery target [7]. Although the *las* system has been studied intensively, most inhibitors have failed to proceed to preclinical development due to structural drawbacks of the developed drugs (i.e., hydrolytically/metabolically labile groups), unfavourable physiochemical properties, weak potency as well as the frequent lasR mutants that arise in chronic *P. aeruginosa* infections, making it difficult to target [7]. Therefore, the *pqs* system is a viable target for quorum sensing inhibition.

## 2. Results and Discussion

### 2.1. Design of Quinazolinone Inhibitors

**Figure 1:**
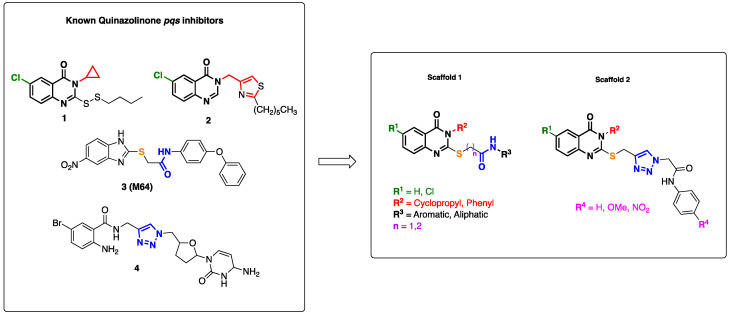
Scaffold 1 and Scaffold 2 for synthesis of quinazolinone compounds based on the structure of known *pqs* inhibitors.

Many reported *pqs* inhibitors contain quinazolinone scaffolds to mimic the quinolone core of 2-heptyl-3-hydroxyquinolin-4(1*H*)-one (PQS) and 2-heptylquinolin-4(1*H*)-one (HHQ) to increase their binding affinity to PqsR [2,9]. The conversion of the quinolone core of PQS into a quinazolinone scaffold whilst replacing the hydroxyl group at the 3-position of PQS with a cyclic group, as shown in quinazolinones **1** and **2**, provided high pyocyanin and biofilm inhibition against *P. aeruginosa* PAO1 [2,9]. Molecular docking studies show that the increased affinity of quinazolinone compounds is due to the aromatic stacking between the quinazoline core and LEU208 and ILE236 of PqsR, and the hydrogen bond between the carbonyl group on the ring and the hydroxyl group of Thr265 in the ligand binding domain of PqsR [10]. Research has also shown that a 6-chloro-substituted quinazolinone (e.g., **2**) demonstrated better activity than 7-chloro-substituted quinazolinones (e.g., **1**) [7]. Additional docking studies have shown that the amide group in M64 (**3**) participates in hydrogen bonding with amino acid residue Gln194 in PqsR and is able to fit into a hydrophobic binding pocket by bending at the central sulfur atom [5]. A secondary scaffold involving the incorporation of a triazole ring was explored, as compounds with this moiety have been shown to inhibit biofilm formation and virulence (**4**) [11]. Therefore, the new scaffolds for the PQS inhibitor analogues will incorporate important aspects of previously known inhibitors, specifically a quinazolinone core, sulfur atom, an amide group and a triazole ring (Figure 1).

### 2.2. Molecular Docking Using GOLD

In silico computational docking experiments were conducted using GOLD software in conjunction with Discovery Studio (DS) to predict intermolecular interactions between the quinazolinone ligands and PqsR receptor ligand binding domain (LBD). These docking studies facilitated a rational approach to SAR analysis and drug design to develop new scaffolds for the synthesis of *pqs* inhibitors.

#### 2.2.1. Interactions of M64 and PQS with PqsR

**Figure 2:**
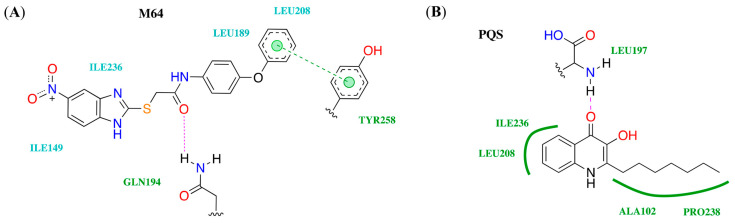
(**A**) M64 and (**B**) PQS interactions with key amino acids in the PqsR receptor. Pink dashed lines indicate hydrogen bonds. Green dashed lines connecting two green dots indicates aromatic stacking. Green solid lines indicate hydrophobic interactions around M64. Amino acid residues capable of making hydrophobic interactions are shown in light blue.

M64 is a known pqs inhibitor that has shown great therapeutic efficiency in the treatment of *P. aeruginosa* infections [12]. A cocrystal of M64 in complex with the PqsR ligand binding domain obtained from the Protein Data Bank (PDB) of Research Collaboratory for Structural Bioinformatics (RCSB) (PDB-6B8A) was used to observe key ligand–receptor interactions in DS [13]. A site-directed mutagenesis of amino acid residues in the PqsR binding pocket have concluded that hydrogen bonding with GLN194 and aromatic stacking interactions with TYR258 strongly contribute towards its affinity and *pqs* inhibition (Figure 2A) [13,14]. Molecular docking of M64 also suggests that the *sp^3^* nature of the central sulfur atom allows for the correct geometry for the compound to fit into the hydrophobic binding pocket. Additionally, the docking of the natural ligand PQS into the LBD (Figure 2B) revealed that hydrogen bonding between LEU197 and the carbonyl moiety of the quinolone core could be a crucial interaction for its affinity with the receptor. Therefore, possible quinazolinone inhibitors were designed to incorporate these intermolecular interactions to achieve high affinity to the PqsR receptor.

#### 2.2.2. Docking Quinazolinone Analogues with PqsR Receptor

Synthetically possible molecules in a library were docked into the PqsR receptor to compare the ligand–receptor interactions with M64 and PQS. The incorporation of cyclic groups at the R^2^ position and amide groups have been shown to increase pqs inhibition in known inhibitors and were thus considered when designing possible analogues [15]. Since the natural ligand PQS has a long alkyl chain extending into the hydrophobic pocket of PqsR, the effect of substituting different-sized alkyl and aromatic amines on predicted interactions were also examined at the R^3^ and R^4^ positions in the respective scaffolds.

**Figure 3:**
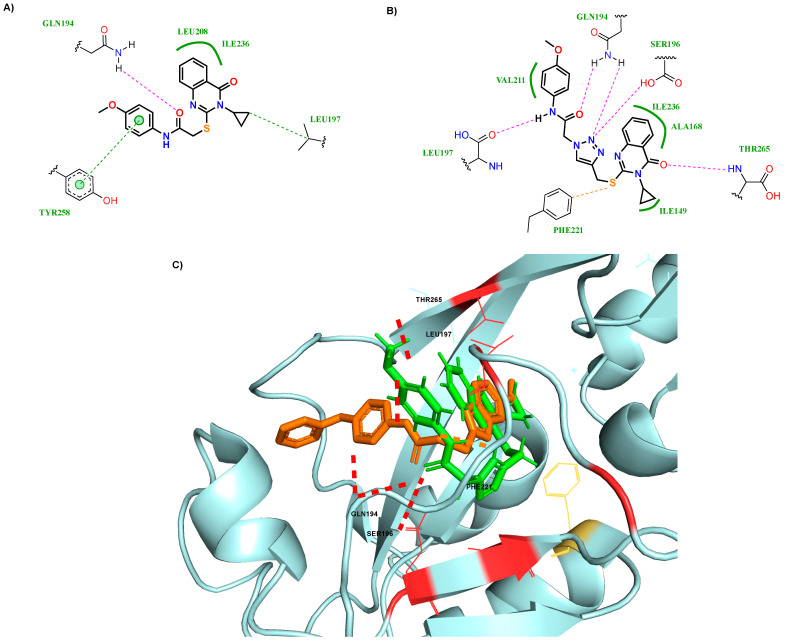
(**A**) Predicted interactions between **5e** and key amino acids in the PqsR receptor. (**B**) Predicted interactions between **6b** and key amino acids in the PqsR receptor. Pink dashed lines indicate hydrogen bonds. Orange dashed lines indicate pi-sulfur interactions. Green solid lines indicate hydrophobic interactions around **6b**. (**C**) Crystal structure of **6b** (green) and M64 (orange) bound to PqsR.

Based on docking results, the quinazolinone amide **5a** containing a cyclopropyl group at the R^2^ position and a butyl chain attached to the amide was designed from Scaffold 1. This analogue showed potential interactions with key residues GLN194 and TYR258 through hydrogen bonding and hydrophobic interactions, respectively. However, aromatic stacking with TYR258 was not observed between **5a** and PqsR. To increase the likelihood of the quinazolinone analogue participating in the key aromatic stacking interaction, the butyl chain was replaced with a 4-methoxyphenyl functional group (**5e**). The docking results indicated that aromatic stacking interactions with TYR258 were predicted to occur, increasing the compound’s ability to bind to the PqsR receptor (Figure 3A). Further synthesis thus involved a focus on incorporating a 4-methoxyphenyl group at the R^3^ position. 

To increase the QSI’s ability to interact with key polar residues in the PqsR receptor, a secondary scaffold was developed, which includes a 1,2,3-triazole ring in between the quinazolinone core and the amide moiety. Triazole rings were chosen because they are known amide bioisosteres and have been proven to be effective in biofilm and virulence inhibition, as they have been used in previous pqs inhibitors such as **4** [11,16]. The amide was incorporated to increase the compound’s likelihood of hydrogen bonding with LEU197, a key amino acid residue with which PQS is able to interact.

Molecular docking of quinazolinone-1,2,3-triazole-phenylacetamide **6b** supported the above hypotheses, as the desired interactions were predicted. The amide group was predicted to form hydrogen bonds with GLN194 and LEU207. The carbonyl group on the quinazolinone core was predicted to form a hydrogen bond with THR265, which is an interaction that was predicted to occur with effective *pqs* inhibitors developed by Grossman et al. in 2018 [10]. Additionally, a pi-sulfur bond was predicted between the sulfur atom and PHE221 (Figure 3B). A previous site-directed mutagenesis study showed that PHE221 played a significant role in *pqs* inhibition, suggesting that the interaction between **6b** and the receptor could be key for *pqs* inhibition [14]. The importance of this sulfur group for orienting the molecule into a hydrophobic binding pocket is reflected in Figure 3C as it mimics the binding position of the known PQS inhibitor M64. Furthermore, an additional hydrogen bond between the triazole ring and SER196 was predicted. While no interactions with TYR258 were predicted to occur and the position of **6b** and M64 do not overlap completely in the receptor (Figure 3C), they still interact with key residues, implying that **6b** should still be antagonistic to *pqs* signalling.

### 2.3. Synthesis of Analogues

**Scheme 1:**
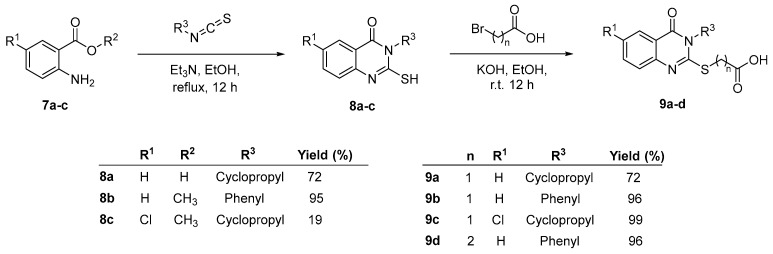
Synthesis of quinazolinone carboxylic acids **9a**–**d**.

The synthesis of 2-mercaptoquinazolin-4(3*H*)-one analogues was essential for the preparation of possible *pqs* inhibitors containing a quinazolinone core. Anthranilic acid **7a**, methyl anthranilate **7b** and 5-chloro-substituted methyl anthranilate **7c** were chosen as the precursors for this reaction series. Anthranilates **7a**–**c** were reacted with an appropriate isothiocyanate and triethylamine in ethanol under reflux conditions to afford the 2-mercaptoquinazolin-4(3*H*)-one intermediates **8a**–**c** in 19–95% yield (Scheme 1). Quinazolinone thiol **8c** was obtained in low yields due to the electron-withdrawing nature of chlorine in **7c**, which withdraws electrons from the amino group, decreasing its nucleophilicity. The quinazolinone thiols were converted to quinazolinone carboxylic acids using a modified version of the procedure described by Savino et al. (2018) [17]. In these reactions, the thiols **8a**–**8c** were stirred with 2.0 equivalents of bromoacetic acid and potassium carbonate in ethanol at room temperature for 12 h to generate **9a**–**d** in a 72–99% yield (Scheme 1).

**Scheme 2:**
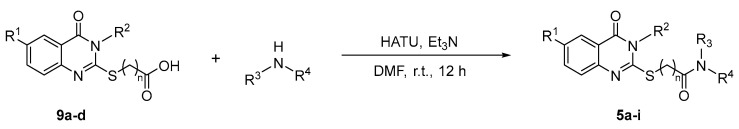
Synthesis of quinazolinone-based amides **5a**–**i**.

Amide functional groups have proven to be critical for *pqs*, biofilm and pyocyanin inhibition in a variety of known inhibitors [11,18]. These amide bonds are generally synthesised by coupling an amine with a carboxylic acid using stoichiometric amounts of a coupling reagent to ensure that the reaction occurs at ambient temperatures [19]. The synthesis of quinazolinone-based amides was attempted under different reaction conditions, and it was identified that the use of hexafluorophosphate azabenzotriazole tetramethyl uronium (HATU) as the coupling reagent provided the highest yield for the reaction (Scheme S1, Table S1). In such an attempt, **9a** was dissolved in anhydrous dimethylformamide (DMF) in the presence of triethylamine and 4-methoxyaniline before HATU was added and the reaction mixture was stirred at room temperature overnight, following a modified procedure reported by Guardia et al. (2016) [20]. After the completion of reaction, water was added to the reaction mixture to precipitate the product and afford pure quinazolinone-based amide **5e** in 64% yield. With HATU identified as the optimal acid–amine coupling regent, compounds **9a**–**d** were reacted with appropriate amines in analogous reactions to afford quinazolinone-based amides **5a**–**i** (Scheme 2, Table 1).

**Scheme 3:**
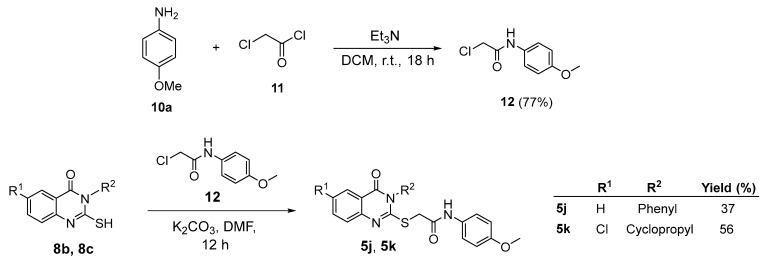
Synthesis of quinazolinone-based amides **5j** and **5k** from thiol analogues **8b** and **8c,** respectively.

Given that not all quinazolinone-based amides were able to be successfully synthesised by reacting carboxylic acid **9** with a substituted amine and HATU, an alternate strategy was proposed: this involved reacting 2-chloroacetamide **12** with **8b** and **8c** to form the corresponding quinazolinone-based amides. 4-Methoxyaniline **10a** was first converted to 2-chloroacetamide **12** by nucleophilic addition with chloroacetyl chloride **11** at room temperature for 18 h. 2-Chloroacetamide **12** was then reacted with **8b** and **8c** in DMF at 50 °C to produce their corresponding quinazolinone-based amides **5j** and **5k** in 37% and 56% yield, respectively (Scheme 3).

**Scheme 4:**
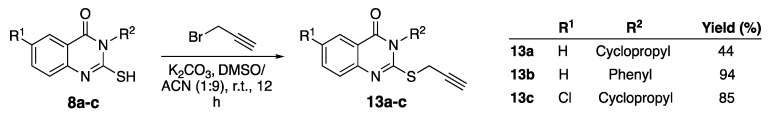
Synthesis of alkynyl quinazolinones **13a**–**c**.

To synthesise target Scaffold 2 in the effort to develop possible *pqs* inhibitors, alkynyl quinazolinones **13a**–**c** were reacted with substituted 2-bromoacetamides **15a**–**c** to synthesise a series of quinazolinone-1,2,3-triazole-phenylacetamide derivatives **6a**–**e**. These alkynyl quinazolinones **13a**–**c** were synthesised by reacting thiol analogues **8a**–**c** with propargyl bromide in the presence of potassium carbonate to initiate a nucleophilic substitution reaction (Scheme 4). 

**Scheme 5:**
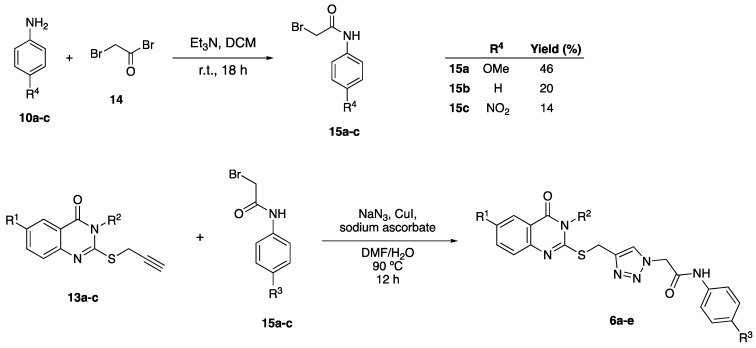
Synthesis of quinazolinone-1,2,3-triazole-phenylacetamides **6a**–**e**.

In order to incorporate the triazole moiety, a one-pot synthesis of quinazolinone-1,2,3-triazole-phenylacetamide was carried out by performing click chemistry reactions via copper-catalysed azide–alkyne cycloaddition. The required 2-bromoacetamides **15a**–**c** for the click chemistry reactions were first prepared by reacting aniline derivatives **10a**–**c** with bromoacetic acid **14** (Scheme 5). These 2-bromoacetamides **15a**–**c** were then reacted with **13a**–**c** in the presence of sodium azide, sodium ascorbate and a catalytic amount of CuI in DMF/H_2_O at 90 °C to afford **6a**–**e** in 10–55% yield (Scheme 5, Table 2). 2-Bromoacetamides **15a**–**c** were used in this reaction instead of the corresponding 2-chloro-substituted acetamides because bromide ion is a better leaving group than chloride ion due to its size and ability to stabilise a negative charge, hence resulting in a more efficient substitution reaction. 

**Figure 4:**
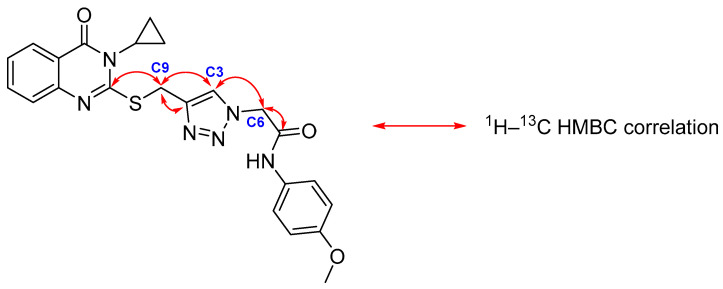
Key HMBC correlations in compound **6b**.

The regioselectivity of the copper-catalysed azide–alkyne 1,3-dipolar cycloaddition reactions in the formation of products **6a**–**e** was confirmed using 2D ^1^H:^13^C HMBC NMR spectroscopy. As a representative example, in the ^1^H:^13^C HMBC NMR of compound **6b**, the protons at both CH_2_ groups on C6 (5.26 ppm) and C9 (4.57 ppm) showed a correlation with the aromatic CH carbon on the triazole ring at C3, indicating the successful formation of the 1,3-disubstituted triazole ring (Figure 4 and Figure S1).

### 2.4. PQS Inhibition Assays and Structure–Activity Relationships

The ability of the synthesised quinazolinone analogues in inhibiting the *pqs* system was evaluated using a *pqsA:gfp* reporter assay, which measures the PqsR regulated expression of the pqsABCDE operon [21]. Ideal lead compounds as QS inhibitors would effectively inhibit cell communication via *pqs* with a minimal inhibition of bacterial growth to reduce the exerted selective pressure and decrease the likelihood resistance development. The results of the PQS inhibition assay and growth inhibition assay are shown in Table 3 and Table S2, respectively. By developing 16 analogues across two scaffolds (quinazolinone amides **5a**–**5k** and quinazolinone-1,2,3-triazole-phenylacetamides **6a**–**e**), it was discovered that **6b** exhibited the most potent *pqs* inhibition (73.4%, 72.1% and 53.7% at 100, 50 and 25 µM, respectively). These results were consistent with the numerous interactions with key amino acid residues (i.e., GLN194, LEU207, THR265, SER196 and PHE221) observed in the molecular docking of **6b** (Figure 3C). Moreover, all the analogues did not inhibit the growth of bacteria substantially.

While the difference in activity between quinazolinone-based amides and quinazolinone-1,2,3-triazole-phenylacetamides **6a**–**e** is not substantial, the general trend was that compounds containing the electron-donating 4-methoxyphenyl substituent at the terminal position possessed a slightly higher QSI activity, with **6b** displaying the highest levels of QSI activity (73.4%, 72.1% and 53.7% at 100, 50 and 25 µM, respectively). However, it should be noted that **6e** possessed the second highest QSI activity of 63.3% at 100 µM and contained an electron-withdrawing nitro group. With the exception of **6b**, the minimal difference in activity across the two scaffolds was unexpected as numerous existing compounds containing triazole rings exhibited a greater *pqs* inhibition compared to quinazolinone-based amides. Nevertheless, these analogues displayed slightly higher levels of *pqs* inhibition compared to the quinazolinone-based amides **5a**–**k**. 

The introduction of chlorine at the 6-position of the quinazolinone core in **5d** and **5i** had no significant effect on their QSI activity at 100 µM despite the previous literature studies showing a significant increase in *pqs* inhibition [7,15]. A previous study by Sabir et al. found that quinazolinone analogues containing a cyclopropyl group on the 3-position of the quinazolinone core tended to have higher levels of *pqs* inhibitory activity than compounds containing a phenyl group [14]. The results for the quinazolinone-based amides **5 a**–**k** were not consistent with these findings for the synthesised quinazolinone-based amides. Compounds **5c**, **5h** and **5j** containing a phenyl group had a slightly higher or similar *pqs* inhibitory activity at all tested concentrations compared to analogous compounds containing a cyclopropyl group. In contrast, quinazolinone-1,2,3-triazole-phenylacetamides **6d** with a phenyl group at the 3-position of the quinazolinone core exhibited significantly lower *pqs* inhibition compared to the corresponding compound **6b** bearing a cyclopropyl group, resulting in a similar trend to that observed for quinazolinone-1,2,3-triazole-phenylacetamides in the study by Sabir et al. Since the cyclopropyl-containing compound **6b** possessed the highest activity of all compounds, it was concluded that the installation of the cyclopropyl group significantly contributes to *pqs* inhibitory activity. 

The difference in QSI activity between **5e** and **5j** was also unexpected as molecular docking shows that **5j** was unable to participate in pi-sulfur bonds with PqsR, yet it still possesses a higher QSI activity than **5e** at 25 and 50 µM. This could be due to the increased number of hydrophobic interactions between the phenyl substituent and the receptor, which could accumulate to provide a stronger interaction than a pi-sulfur bond. Moreover, it can be seen that **5j** with an ethyl linker had an inhibitory activity of 43.0% at 50 µM while **5f** with a propyl linker had an inhibitory activity of 11.7% at 50 µM, suggesting that increasing the carbon chain on the amide group decreases *pqs* inhibition. Initial docking studies showed that quinazolinone analogues containing primary amides were predicted to make a higher number of hydrophobic interactions than secondary amides, potentially increasing their affinity to PqsR and thus *pqs* inhibition. Indeed, compounds containing the secondary amide from morpholine (**5g**–**i**) generally showed a lower *pqs* inhibitory activity at 100 and 25 µM. However, this trend was less clear at 50 µM, with **5h** and **5i** bearing a secondary amide moiety having a similar QSI activity as their corresponding compounds **5c** and **5d,** which bear a primary amide moiety.

Docking studies of quinazolinone-1,2,3-triazole-phenylacetamides **6a,c**–**e** showed that these compounds were not predicted to participate in the key ligand–receptor interactions observed for **6b**, especially the hydrogen bonds with SER196 and LEU207, and the pi-sulfur bond with PHE221. As **6b** was the most active compound at all tested concentrations, the *pqs* inhibition assay suggests that interactions with these residues of the receptor may be significant for QSI activity. Additionally, compound **6e** with a 4-nitro group at the terminal phenyl ring had a QSI activity of 63.3% at 100 µM and was the second most active compound at that concentration. However, at a lower concentration of 25 µM, its corresponding unsubstituted compounds **6a** had a higher QSI activity of 21.6% compared to compound **6e** with a QSI activity of 7.3%. These suggested that the introduction of the electron-donating 4-methoxy group at the terminal phenyl ring of quinazolinone-1,2,3-triazole-phenylacetamides had a larger effect on QSI activity compared with compounds containing the electron-withdrawing nitro group.

### 2.5. Biofilm Inhibition

Selected potent compounds **6b** and **6e** identified in the QS inhibition assay were tested for their ability to inhibit the formation of *P. aeruginosa* biofilm. In this assay, compounds **6b** and **6e** at 25, 50 and 100 µM were incubated with *P. aeruginosa* at 37 °C under static conditions overnight. After incubation, the supernatant was removed, and the loosely bound and planktonic bacterial cells were washed away. *P. aeruginosa* biofilms, which adhered to the plate substratum, were then quantified by crystal violet staining [22,23].

**Figure 5:**
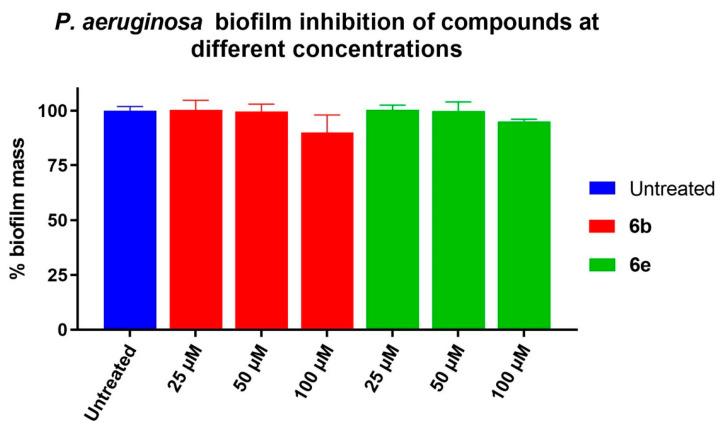
Inhibition of *P. aeruginosa* biofilm formation by test compounds at different concentrations. Error bars represent the standard error of at least triplicate (n ≥ 3).

Both compounds **6b** and **6e** were poor *P. aeruginosa* biofilm inhibitors as they only managed to inhibit 10% and 5%, respectively, of *P. aeruginosa* biofilm formation at 100 µM (Figure 5). At lower concentrations of 25 and 50 µM, no inhibition of *P. aeruginosa* biofilm formation was observed. The results were in contradiction with the QS inhibition assay, as it was expected that these compounds would be able to significantly inhibit the formation of *P. aeruginosa* biofilm via the *pqs*-based QS system due to their high QSI activity. This discrepancy could be because of the formation of biofilm via other QS systems, and this will be explored in future studies [24].

## 3. Materials and Methods

### 3.1. In Silico Studies

Ligands were initially sketched and protons were added before performing full energy minimisation using Discovery Studio Client 2018 (Accelrys Inc., San Diego, CA, USA) with CHARM forcefield and default setting with max steps set to 10,000. All minimised ligands were docked using GOLD (Cambridge Crystallography Date Centre, Cambridge, UK) through Discovery Studio onto orthorhombic space group c222_1_, which is the M64 ligand binding domain of PqsR and OdDHL ligand binding domain of LasR. The protocol was run with default settings except for: poses = 100; detect cavity = false, early termination = false, flexibility–intramolecular hydrogen bond = true. The docking pose in the largest, highest scoring cluster between 2–3 Å RMSD of ligand heavy atoms was used for all analyses. Images of docked compounds were generated in Discovery Studio 18.1 and ChemDraw 19.0.

### 3.2. Synthesis of Analogues

#### 3.2.1. General Information

Bruker Avance III 300 and Bruker Avance III HD 400 spectrometers (Bruker Pty Ltd., Preston, Victoria, Australia) were used to obtain all ^1^H and ^13^C NMR spectra with the respective solvents using chemical shifts (δ) in parts per million (ppm). Multiplicities for NMR spectra have been assigned using singlet (s), doublet (d), doublet of doublet (dd), doublet of doublet of doublet (ddd), doublet of triplet (dt), triplet (t), quartet (q), doublet of quartet (dq), pentet (p), hextet (h), septet (sept), multiplet (m) and broad singlet (br) as necessary and coupling constants (*J*) in Hertz (Hz). Optimelt melting point apparatus (SRS, Sunnyvale, CA, USA) was used for all measurements of all melting points, uncorrected. High-resolution mass spectra (HRMS) were conducted using Thermo LTQ Orbitrap XL instrument (Thermo Scientific, Waltham, MA, USA) under positive-mode electrospray ionisation. Infrared (IR) spectra were recorded using a Cary 630 FTIR spectrometer (Agilent, Mulgrave, Victoria, Australia) fitted with a diamond attenuated total reflectance (ATR) sample interface. Flash column chromatography was performed using Grace Davisil LC60A silica. All reagents were bought commercially from Sigma Aldrich (Castle Hill, NSW, Australia), Alfa Aesar (Haverhill, MA, USA) and ChemImpex (Wood Dale, IL, USA) and used without extra purification. Anhydrous solvents were acquired from PureSolv MD Solvent Purification System (Inert, Amesbury, MA, USA).

#### 3.2.2. Synthetic Procedures and Experimental Characterisation Data

General synthetic procedure A for 2-mercaptoquinazoline **8a**–**c**

A mixture of corresponding methyl anthranilate **7** (1.0 equivalent), substituted isothiocyanate (1.2 equivalent) and triethylamine (1.2 equivalent) in ethanol (30 mL) was stirred and heated at 100 °C under reflux for 8 h, using thin layer chromatography (TLC) to monitor the reaction. The reaction mixture was then cooled to room temperature. The resulting white solid precipitate was filtered and washed with diethyl ether. The solid was then dried to afford the corresponding 2-mercaptoquinazoline-4(3*H*)-one **8**.

3-Cyclopropyl-2-mercaptoquinazolin-4(3*H*)-one (**8a**)

The title compound **8a** was synthesised from methyl anthranilate (1.00 g, 6.61 mmol), 2-cyclopropyl isothiocyanate (0.673 mL, 7.27 mmol) and triethylamine (1.10 mL, 7.90 mmol) following general synthetic procedure A. The product was obtained as a white fluffy solid (0.72 g, 50%); mp 245.7–250.0 °C; ^1^H NMR (400 MHz, DMSO-*d*_6_) δ 12.77 (s, 1H), 7.93 (dd, *J* = 7.9, 1.4 Hz, 1H), 7.68–7.72 (m, 1H), 7.27–7.36 (m, 2H), 2.79–2.85 (m, 1H), 1.10–1.19 (m, 2H), 0.78–0.83 (m, 2H); ^13^C NMR (100 MHz, DMSO–*d*_6_) δ 117.54, 161.20, 139.75, 135.52, 127.58, 124.53, 116.75, 115.53; IR (ATR): *v*_max_ 2163, 1685, 1621, 1545 cm^−1^; HRMS (+ESI): Found m/z 219.0587, [M + H]^+^, C_11_H_11_N_2_OS required 219.0587.

2-Mercapto-3-phenylquinazolin-4(3*H*)-one (**8b**)

The title compound **8b** was synthesised from anthranilic acid (1.00 g, 7.29 mmol), phenyl isothiocyanate (1.08 g, 8.02 mmol) and triethylamine (1.22 mL, 8.75 mmol) following general synthetic procedure A. The product was obtained as a light grey solid (1.77 g, 95%); mp 294.2–303.6 °C; ^1^H NMR (400 MHz, DMSO-*d*_6_) δ 13.04 (s, 1H), 7.96 (dd, *J* = 8.0, 1.5 Hz, 1H), 7.77–7.81 (m, 1H), 7.33–7.51 (m, 5H), 7.28 (dd, *J* = 7.2, 1.7 Hz, 2H); ^13^C NMR (100 MHz, DMSO-*d*_6_) δ 176.54, 160.30, 140.14, 139.80, 136.07, 129.47, 129.37, 128.57, 127.88, 124.81, 116.69, 116.22; IR (ATR): *v*_max_ 3250, 3218, 3138, 3072, 3033, 1662, 1621, 1527 cm^−1^; HRMS (+ESI): Found m/z 277.0406, [M + Na]^+^, C_14_H_10_N_2_OSNa required 277.0406.

6-Chloro-3-cyclopropyl-2-mercaptoquinazolin-4(3*H*)-one (**8c**)

The title compound **8c** was synthesised from 5-chloro methyl anthranilate (0.500 g, 2.69 mmol), cyclopropyl isothiocyanate (0.274 mL, 2.96 mmol) and triethylamine (0.449 mL, 3.23 mmol) following general synthetic procedure A. The product was obtained as a white fluffy solid (0.132 g, 19%); mp 187.7–202.2 °C; ^1^H NMR (400 MHz, DMSO-*d*_6_) δ 12.88 (s, 1H), 7.86 (d, *J* = 2.4 Hz, 1H), 7.75 (dd, *J* = 8.8, 2.5 Hz, 1H), 7.36 (d, *J* = 8.8 Hz, 1H), 2.79–2.84 (m, 1H), 1.14–1.19 (m, 2H), 0.79–0.83 (m, 2H); ^3^C NMR (100 MHz, DMSO-*d*_6_) δ 177.45, 160.28, 138.59, 135.41, 128.30, 126.52, 118.19, 118.14, 30.20, 12.07 cm^−1^; IR (ATR): *v*_max_ 3169, 3102, 2168, 2009, 1701, 1622, 1540; HRMS (+ESI): Found m/z 253.0197, [M + H]^+^, C_11_H_10_ClN_2_OS required 253.0197.

General synthetic procedure B for quinazolinone carboxylic acid derivatives **9**

Appropriate carboxylic acid (1.7 equivalent) in EtOH (5 mL) was added to a solution of compound **8** (1.0 equivalent) in EtOH (5 mL). Potassium hydroxide (10 mL) was then added. The reaction mixture was stirred at room temperature overnight. After completion, water (10 mL) was added to the reaction mixture, which was acidified with HCl to pH 2 to produce a cloudy white suspension. The crude product was then filtered and washed with water before being purified with flash column chromatography on silica gel using *n*-hexane/EtOAc as eluent to afford a pure white solid product **9**.

2-((3-Cyclopropyl-4-oxo-3,4-dihydroquinazolin-2-yl)thio)acetic acid (**9a**)

The title compound **9a** was synthesised from thiol **8a** (0.500 g, 2.29 mmol) and bromoacetic acid (0.541 g, 3.89 mmol) following general synthetic procedure B. The product was obtained as a fluffy white solid (0.458 g, 64%); mp 165.2–191.6 °C; ^1^H NMR (400 MHz, DMSO-*d*_6_) δ 8.03 (dd, *J* = 7.2, 3.6 Hz, 1H), 7.73–7.77 (m, 1H), 7.40–7.44 (m, 2H), 4.03 (s, 2H), 2.90–2.95 (m, 1H), 1.21–1.26 (m, 2H), 0.95–0.99 (m, 2H); ^13^C NMR (100 MHz, DMSO-*d*_6_) δ 170.25, 161.90, 159.17, 146.87, 134.93, 126.77, 126.23, 126.08, 119.84, 34.86, 26.93, 11.07; IR (ATR): *v*_max_ 2645, 2160, 1719, 1688, 1630, 1608, 1553 cm^−1^; HRMS (+ESI): Found m/z 299.0458, [M + Na]^+^, C_13_H_12_N_2_O_3_SNa required [M + Na]^+^ 299.0461.

2-((4-Oxo-3-phenyl-3,4-dihydroquinazolin-2-yl)thio)acetic acid (9b)

The title compound **9b** was synthesised from thiol **8b** (0.500 g, 1.96 mmol) and bromoacetic acid (0.464 g, 3.34 mmol) following general synthetic procedure B. The product was obtained as an off-white solid (0.599 g, 96%); mp 192.2–203.7 °C; ^1^H NMR (400 MHz, DMSO-*d*_6_) δ 8.09 (dd, *J* = 8.0, 1.5 Hz, 1H), 7.82–7.86 (m, 1H), 7.55–7.63 (m, 4H), 7.46–7.51 (m, 3H); ^13^C NMR (100 MHz, DMSO-*d*_6_) δ 169.97, 161.11, 157.15, 147.54, 136.19, 135.45, 130.50, 130.05, 129.87, 127.08, 126.58, 126.48, 119.95, 35.13, 19.02; IR (ATR): *v*_max_ 2645, 2160, 1719, 1688, 1630, 1608, 1553 cm^−1^; HRMS (+ESI): Found m/z 335.0460, [M + Na]^+^, C_16_H_12_N_2_O_3_SNa required 335.0461.

2-((6-Chloro-3-cyclopropyl-4-oxo-3,4-dihydroquinazolin-2-yl)thio)acetic acid (**9c**)

The title compound **9c** was synthesised from thiol **8c** (0.200 g, 0.791 mmol) and bromoacetic acid (0.187 g, 1.582 mmol) following general synthetic procedure B. The product was obtained as a white solid (0.241 g, 99%); mp 187.7–202.2 °C; ^1^H NMR (400 MHz, DMSO-*d*_6_) δ 7.95 (d, 1H), 7.78 (dd, *J* = 8.0 Hz, 1H), 7.45 (d, *J* = 8.8 Hz, 1H), 4.03 (s, 2H), 2.91–2.95 (m, 1H), 1.19–1.26 (m, 2H), 0.95–1.00 (m, 2H); ^13^C NMR (100 MHz, DMSO-*d*_6_) δ 170.15, 160.91, 160.08, 145.58, 134.97, 130.15, 128.32, 125.73, 121.10, 34.94, 27.08, 11.04; IR (ATR): *v*_max_ 3214, 2217, 2916, 2087, 1640, 1574 cm^−1^; HRMS (+ESI): m/z 333.0072, [M + Na]^+^, C_13_H_11_ClN_2_O_3_SNa required 333.0071.

3-((4-Oxo-3-phenyl-3,4-dihydroquinazolin-2-yl)thio)propanoic acid (**9d**)

The title compound **9d** was synthesised from thiol **8b** (0.250 g, 0.983 mmol) and 3-bromopropanoic acid (0.256 g, 1.67 mmol) following general synthetic procedure B. The product was obtained as a white solid (0.136 g, 96%); mp 187.1–230.7 °C; ^1^H NMR (400 MHz, DMSO-*d*_6_) δ 8.09 (dd, *J* = 7.9, 1.5 Hz, 1H), 7.82–7.87 (m, 1H), 7.52–7.65 (m, 4H), 7.47–7.51 (m, 1H), 7.43–7.47 (m, 2H), 3.29 (t, *J* = 7.0 Hz, 2H), 2.66–2.81 (m, 2H); ^13^C NMR (100 MHz, DMSO-*d*_6_) δ 161.13, 156.19, 147.57, 136.03, 135.47, 130.57, 130.03, 129.93, 127.09, 126.69, 126.65, 120.09, 79.72, 74.50, 20.88; IR (ATR): *v*_max_ 3226, 1996, 1665, 1622, 1533 cm^−1^; HRMS (+ESI): Found m/z 327.0798, [M + H]^+^, C_17_H_15_N_2_O_3_S required 327.0798.

General synthetic procedure C for quinazolinone-based amides **5a**–**i**

A solution of the appropriate acetic acid **9** (1.0 equivalent), substituted amine (1.1 equivalent) and triethylamine (2.0 equivalent) in anhydrous DMF (2 mL) was prepared. HATU (1.1 equivalent) was then added to the solution and the reaction mixture was stirred at room temperature overnight. After completion of the reaction, water was added and the mixture extracted with EtOAc (3 × 15 mL). The combined organic extracts were washed with brine (30 mL), dried over anhydrous magnesium sulfate, and concentrated in vacuo. The crude mixture was purified with flash column chromatography on silica gel using n-hexane/EtOAc as eluent to afford the solid product **5a**–**i**.

*N*-Butyl-2-((3-cyclopropyl-4-oxo-3,4-dihydroquinazolin-2-yl)thio)acetamide (**5a**)

The title compound **5a** was synthesised from quinazolinone carboxylic acid **9a** (0.100 g, 0.362 mmol), butylamine (0.039 mL, 0.398 mmol), triethylamine (0.100 mL, 0.724 mmol) and HATU (0.151 g, 0.398 mmol) following general synthetic procedure C. The product was obtained as a white solid (20 mg, 17%); mp 92.4–100.6 °C; ^1^H NMR (400 MHz, DMSO-*d*_6_) δ 8.16 (t, *J* = 5.7 Hz, 1H), 8.04 (dd, *J* = 8.0 Hz, 1H), 7.74–7.78 (m, 1H), 7.39–7.48 (m, 2H), 3.92 (s, 2H), 3.07 (q, *J* = 4.0 Hz, 2H), 2.89–2.94 (m, 1H), 1.31–1.41 (m, 2H), 1.20–1.29 (m, 4H), 0.95–1.00 (m, 2H), 0.81 (t, *J* = 7.3 Hz, 3H); ^13^C NMR (100 MHz, DMSO-*d*_6_) δ 167.16, 161.96, 159.48, 146.98, 134.83, 126.75, 126.14, 126.06, 119.89, 36.30, 31.65, 26.94, 19.96, 14.08, 11.10; IR (ATR): *v*_max_ 3312, 3090, 2962, 2870, 1680, 1648, 16,608, 1579, 1550 cm^−1^; HRMS (+ESI): Found m/z 332.1427, [M + H^+^], C_17_H_22_N_3_O_2_S required 332.1427.

*N*-Benzyl-2-((3-cyclopropyl-4-oxo-3,4-dihydroquinazolin-2-yl)thio)acetamide (**5b**)

The title compound **5b** was synthesised from quinazolinone carboxylic acid **9a** (0.100 g, 0.36 mmol), benzylamine (0.0435 mL, 0.400 mmol), triethylamine (0.100 mL, 0.724 mmol) and HATU (0.151 g, 0.398 mmol) following general synthetic procedure C. The product was obtained as a white solid (40 mg, 48%); mp 157.3–159.5 °C; ^1^H NMR (400 MHz, DMSO *d*_6_) δ 8.71 (t, *J* = 6.0 Hz, 1H), 8.04 (dd, *J* = 8.3, 1.6 Hz, 1H), 7.74–7.78 (m, 1H), 7.41–7.44 (m, 2H), 7.18–7.24 (m, 5H), 4.02 (s, 2H), 2.90–2.95 (m, 1H), 1.20–1.25 (m, 2H), 0.97–1.01 (m, 2H); ^13^C NMR (100 MHz, DMSO-*d*_6_) δ 166.31, 161.90, 159.48, 146.94, 134.93, 126.77, 126.14, 126.10, 119.85, 66.63, 66.53, 46.50, 42.62, 40.63, 40.42, 40.21, 40.00, 39.79, 39.59, 39.38, 35.16, 26.93, 11.20; IR (ATR): *v*_max_ 3362, 2013, 1663, 1610, 1580, 1530 cm^−1^; HRMS (+ESI): Found m/z 388.1091, [M + Na]^+^, C_20_H_19_N_3_O_2_SNa required 388.1090.

*N*-Benzyl-2-((4-oxo-3-phenyl-3,4-dihydroquinazolin-2-yl)thio)acetamide (**5c**)

The title compound **5c** was synthesised from quinazolinone carboxylic acid **9b** (0.100 mg, 0.320 mmol), benzylamine (0.0384 mL, 0.352 mmol), triethylamine (0.0890 mL, 0.640 mmol) and HATU (0.134 g, 0.352 mmol) following general synthetic procedure C. The product was obtained as a white solid (12 mg, 15%); mp 186.6–189.9 °C; ^1^H NMR (400 MHz, DMSO-*d*_6_) δ 8.68 (t, *J* = 6.0 Hz, 1H), 8.09 (dd, *J* = 8.1, 1.5 Hz, 1H), 7.83–7.87 (m, 1H), 7.41– 7.66 (m, 7H), 7.26 (d, *J* = 4.5 Hz, 4H), 4.30 (d, *J* = 6.0 Hz, 2H), 3.95 (s, 2H); ^13^C NMR (100 MHz, DMSO-*d*_6_) δ 167.20, 161.17, 157.41, 147.63, 139.62, 136.28, 135.34, 130.43, 130.00, 129.91, 128.67, 127.53, 127.20, 127.03, 126.56, 126.49, 120.01, 42.95, 36.48; IR (ATR): *v*_max_ 3294, 1689, 1643, 1607, 1547 cm^−1^. HRMS (+ESI): Found m/z 424.1085, [M + Na]^+^, C_23_H_19_N_3_O_2_SNa required 424.1090.

*N*-Benzyl-2-((6-chloro-3-cyclopropyl-4-oxo-3,4-dihydroquinazolin-2-yl)thio)acetamide (**5d**)

The title compound **5d** was synthesised from quinazolinone carboxylic acid **9c** (0.100, 0.322 mmol), benzylamine (0.0387 mL, 0.354 mmol), triethylamine (0.0897 mL, 0.0.644 mmol) and HATU (0.135 g, 0.354 mmol) following general synthetic procedure C. The product was obtained as a white solid (13 mg, 14%); mp 145.7–192.2 °C; ^1^H NMR (400 MHz, DMSO-*d*_6_) δ 8.71 (t, *J* = 6.1 Hz, 1H), 7.96 (t, *J* = 2.4 Hz, 1H), 7.79 (dd, *J* = 2.6 Hz, 1H), 7.43 (d, *J* = 8.7 Hz, 1H), 7.19–7.24 (m, 5H), 4.26 (d, *J* = 6.0 Hz, 2H), 4.02 (s, 2H), 2.91–2.96 (m, 1H), 1.19–1.25 (m, 2H), 0.97–1.02 (m, 2H); ^13^C NMR (100 MHz, DMSO-*d*_6_) δ 67.40, 161.01, 160.31, 145.68, 1 39.67, 134.86, 130.08, 128.62, 128.43, 127.48, 127.17, 125.66, 121.15, 42.95, 27.11, 11.04; IR (ATR): *v*_max_ 3281, 1685, 1647, 1547, 1510 cm^−1^; HRMS (+ESI): Found m/z 422.0696, [M + Na]^+^, C_20_H_18_ClN_3_O_2_SNa required 422.0700.

2-((3-Cyclopropyl-4-oxo-3,4-dihydroquinazolin-2-yl)thio)-*N*-(4-methoxyphenyl)acetamide (**5e**)

The title compound **5e** was synthesised from quinazolinone carboxylic acid **9a** (0.100, 0.362 mmol), 4-methoxyaniline (0.0490 g, 0.398 mmol), triethylamine (0.100 mL, 0.724 mmol) and HATU (0.151 g, 0.398 mmol) following general synthetic procedure C. The product was obtained as a light purple solid (88 mg, 64%); mp 187.6–205.1 °C; ^1^H NMR (400 MHz, DMSO-*d*_6_) δ 10.21 (s, 1H), 8.03 (dd, *J* = 7.9, 1.5 Hz, 1H), 7.72–7.76 (m, 1H), 7.49–7.53 (m, 2H), 7.39–7.46 (m, 2H), 4.12 (s, 2H), 3.72 (s, 3H), 2.91–2.97 (m, 1H), 1.22–1.27 (m, 2H), 0.97–1.02 (m, 2H); ^13^C NMR (100 MHz, DMSO-*d*_6_) δ 165.95, 134.91, 126.01, 121.19, 114.37, 55.63, 37.33, 26.98, 11.10; IR (ATR): *v*_max_ 3278, 2894, 2020, 1682, 1652, 1598, 1557, 1536, 1514 cm^−1^; HRMS (+ESI): Found m/z 404.1032, [M + Na]^+^, C_20_H_19_N_3_O_3_SNa required 404.1039.

N-(4-Methoxyphenyl)-3-((4-oxo-3-phenyl-3,4-dihydroquinazolin-2-yl)thio)propenamide (5f)

The title compound **5f** was synthesised from quinazolinone carboxylic acid **9d** (0.0500 g, 0.153 mmol), 4-methoxyaniline (0.0207 g, 0.168 mmol), triethylamine (0.031 mL, 0.306 mmol) and HATU (0.0640 g, 0.168 mmol) following general synthetic procedure C. The product was obtained as a white solid (38 mg, 54%); mp 180.0–191.2 °C; ^1^H NMR (400 MHz, DMSO-*d*_6_) δ 9.81 (s, 1H), 8.10 (dd, 1H), 7.83–7.87 (m, 1H), 7.64 (d, 1H), 7.51–7.55 (m, 4H), 7.45–7.49 (m, 4H), 3.72 (s, 3H), 3.40 (t, *J* = 6.7 Hz, 2H), 2.73–2.78 (m, 2H). ^13^C NMR (100 MHz, DMSO-*d*_6_) δ 169.17, 161.27, 157.65, 155.60, 147.78, 136.43, 135.38, 132.67, 130.27, 129.91, 129.87, 127.04, 126.61, 126.43, 121.26, 121.07, 121.03, 120.05, 114.38, 114.29, 55.62, 38.72, 35.62, 28.25; IR (ATR): *v*_max_ 3309, 3064, 2927, 2954, 1735, 1692, 1649, 1600, 1544, 1510 cm^−1^; HRMS (+ESI): m/z 432.1375, [M + H]^+^, C_24_H_22_N_3_O_3_S required 432.1376.

3-Cyclopropyl-2-((2-morpholino-2-oxoethyl)thio)quinazolin-4(3*H*)-one (**5g**)

The title compound **5g** was synthesised from quinazolinone carboxylic acid **9a** (0.100 g, 0.362 mmol), morpholine (0.0347 mL, 0.398 mmol), triethylamine (0.100 mL, 0.724 mmol) and HATU (0.151 g, 0.398 mmol) following general synthetic procedure C. The product was obtained as a white solid (61 mg, 49%); mp 181.0–238.3 °C; ^1^H NMR (400 MHz, DMSO-*d*_6_) δ 8.04 (dd, *J* = 8.0 Hz, 1H), 7.74–7.79 (m, 1H), 7.40–7.46 (m, 2H), 4.25 (s, 2H), 3.68 (s, 4H), 3.58 (t, *J* = 4.8 Hz, 2H), 3.47 (t, *J* = 4.7 Hz, 2H), 2.90–2.95 (m, 1H), 1.22–1.27 (m, 2H), 0.95–0.99 (m, 2H); ^13^C NMR (100 MHz, DMSO-*d*_6_) δ 166.32, 161.91, 159.48, 146.94, 134.94, 126.77, 126.15, 126.11, 66.62, 66.53, 46.50, 42.62, 35.14, 26.93, 11.19; IR (ATR): *v*_max_ 3082, 2964, 2917, 2853, 1988, 1672, 1645, 1608, 1547 cm^−1^; HRMS (+ESI): Found m/z 368.1040, [M + Na]^+^, C_17_H_19_N_3_O_3_SNa required 368.1039.

2-((2-Morpholino-2-oxoethyl)thio)-3-phenylquinazolin-4(3*H*)-one (**5h**)

The title compound **5h** was synthesised from quinazolinone carboxylic acid **9b** (0.100 g, 0.320 mmol), morpholine (0.031 mL, 0.352 mmol), triethylamine (0.0650 mL, 0.640 mmol) and HATU (0.134 g, 0.352 mmol) following general synthetic procedure C. The product was obtained as a white solid (39 mg, 32%); mp 181.6–228.9 °C; ^1^H NMR (400 MHz, DMSO-*d*_6_) δ 8.03 (dd, *J* = 8.0, 1.5 Hz, 1H), 7.74–7.78 (m, 1H), 7.40–7.48 (m, 2H), 4.25 (s, 2H), 3.68 (s, 4H), 3.48 (t, *J* = 4.8 Hz, 2H), 2.90–2.93 (m, 1H), 1.22–1.27 (m, 2H), 0.95–0.99 (m, 2H); ^13^C NMR (100 MHz, DMSO-*d*_6_) δ 166.04, 162.79, 161.11, 157.50, 147.62, 136.28, 135.45, 130.46, 130.03, 129.88, 127.07, 126.51, 119.97, 66.57, 66.48, 46.49, 42.55, 36.26, 35.75, 31.25; IR (ATR): *v*_max_ 3055, 2856, 2204, 1673, 1650, 1604, 1546 cm^−1^; HRMS (+ESI): Found m/z 382.1223, [M + H]^+^, C_20_H_20_N_3_O_2_S required 382.1220.

6-Chloro-3-cyclopropyl-2-((2-morpholino-2-oxoethyl)thio)quinazolin-4(3*H*)-one (**5i**)

The title compound **5i** was synthesised from quinazolinone carboxylic acid **9c** (0.0500 g, 0.161 mmol), morpholine (0.0154 mL, 0.177 mmol), triethylamine (0.0500 mL, 0.322 mmol) and HATU (0.0673 g, 0.177 mmol) following general synthetic procedure C. The product was obtained as a white solid (59 mg, 97%); mp 180.0–191.2 °C, ^1^H NMR (400 MHz, DMSO-*d*_6_) δ 7.96 (d, *J* = 2.5 Hz, 1H), 7.79 (dd, *J* = 8.7 Hz, 2.5 Hz, 1H), 7.51 (d, *J* = 8.7 Hz, 1H), 4.26 (s, 2H), 3.67 (s, 4H), 3.58 (t, *J* = 5.3 Hz, 2H), 3.47 (t, *J* = 5.2 Hz, 2H), 2.90–2.95 (m, 1H), 1.22–1.27 (m, 2H), 0.96–1.00 (m, 2H); ^13^C NMR (100 MHz, DMSO-*d*_6_) δ 166.18, 162.00, 157.94, 146.87, 134.98, 130.54, 128.40, 125.72, 121.11, 66.61, 48.41, 48.03, 42.61, 35.34, 27.09, 11.17. IR (ATR): *v*_max_ 3063, 2967, 2855, 1674, 1651, 1604, 1542 cm^−1^; HRMS (+ESI): Found m/z 402.0656, [M + Na]^+^, C_17_H_18_ClN_3_O_3_SNa required 402.0650.

2-Chloro-*N*-(4-methoxyphenyl)acetamide (**12**)

A stirred solution of 4-methoxyaniline **10a** (0.500 g, 4.06 mmol, 1.0 equivalent) in dichloromethane (DCM) (15 mL) was cooled to −10 °C under an argon atmosphere. Triethylamine (0.622 mL, 0.470 mmol, 1.1 equivalent) and chloroacetyl chloride **11a** (0.550 g, 4.872 mmol, 1.2 equivalent) were added successively and the reaction mixture was stirred at room temperature overnight. The resulting reaction mixture was treated with 10% HCl (10 mL) and then added to ice. The product was extracted into DCM (3 × 30 mL), and the combined organic extracts washed with brine, dried over anhydrous magnesium sulfate and concentrated in vacuo. The resulting crude compound was purified with flash column chromatography on silica gel using DCM/MeOH as eluent to afford the acetamide **12a** as an off-white solid powder (0.626 g, 77%); mp 120.9–123.9 °C; ^1^H NMR (400 MHz, DMSO-*d*_6_) δ 10.15 (s, 1H), 7.36–7.57 (m, 2H), 6.81–7.01 (m, 2H), 4.22 (s, 2H), 3.73 (s, 3H); ^13^C NMR (100 MHz, DMSO-*d*_6_) 164.60, 156.09, 132.02, 121.42, 114.43, 55.64, 43.98; IR (ATR): *v*_max_ 3275, 2954, 2182, 1672, 1574, 1543 cm^−1^; HRMS (+ESI): Found m/z 222.0291, [M + H^+^], C_9_H_11_ClNO_2_ required 222.0292.

General synthetic procedure D for quinazolinone-based amides 5j and 5k

A solution containing the appropriate thiol compound **8b** or **9c** (1.0 equivalent), 2-chloroacetamide **12a** (1.0 equivalent) and potassium carbonate (2.0 equivalent) in DMF was heated at 50 °C for 12 h. After completion, the reaction mixture was poured into water/ice and the product was extracted with EtOAc (3 × 15 mL). The combined organic extracts were washed with brine, dried over anhydrous magnesium sulfate and concentrated in vacuo to afford the final solid product (**5j** or **5k**).

*N*-(4-Methoxyphenyl)-2-((4-oxo-3-phenyl-3,4-dihydroquinazolin-2-yl)thio)acetamide (**5j**)

The title compound **5j** was synthesised from quinazolinone carboxylic acid **9b** (0.100, 0.393 mmol), 2-chloroacetamide **12a** (0.0785 g, 0.393 mmol) and potassium carbonate (0.109 g, 0.786 mmol) following general synthetic procedure D. The product was obtained as a light pink solid (61 mg, 37%); mp 188.8–201.1 °C; ^1^H NMR (400 MHz, DMSO-*d*_6_) δ 10.20 (s, 1H), 8.09 (dd, 1H), 7.82–7.8 (m, 1H), 7.57–7.63 (m, 4H), 7.45–7.51 (m, 5H), 4.06 (s, 2H), 3.72 (s, 3H); ^13^C NMR (100 MHz, DMSO-*d*_6_) δ 165.61, 161.11, 157.48, 155.80, 147.63, 136.28, 135.43, 132.55, 130.47, 130.02, 129.91, 127.09, 126.51, 126.39, 121.16, 120.03, 114.37, 55.63, 37.70; IR (ATR): *v*_max_ 3271, 3064, 1682, 1652, 1602, 1544, 1510 cm^−1^; HRMS (+ESI): Found m/z 440.1036, [M + Na]^+^, C_23_H_19_N_3_O_3_SNa required 440.1039.

2-((6-Chloro-3-cyclopropyl-4-oxo-3,4-dihydroquinazolin-2-yl)thio)-*N*-(4-methoxyphenyl)acetamide (**5k**)

The title compound **5k** was synthesised from quinazolinone carboxylic acid **9c** (0.100, 0.396 mmol), 2-chloroacetamide **12a** (0.0789 g, 0.396 mmol) and potassium carbonate (0.109 g, 0.792 mmol) following general synthetic procedure D. The product was obtained as a white solid (93 mg, 56%); mp 221.0–223.8 °C; ^1^H NMR (400 MHz, DMSO-*d*_6_) δ 10.21 (s, 1H), 7.95 (d, *J* = 2.5 Hz, 1H), 7.78 (dd, *J* = 8.7, 2.6 Hz, 1H), 7.48–7.52 (m, 3H), 6.74–7.06 (m, 2H), 4.12 (s, 2H), 3.72 (s, 3H), 2.92–2.97 (m, 1H), 1.21–1.27 (m, 2H), 0.98–1.02 (m, 2H); ^13^C NMR (100 MHz, DMSO-*d*_6_) δ 165.86, 160.94, 160.40, 155.81, 145.68, 134.98, 132.60, 130.11, 128.24, 125.73, 121.24, 121.16, 114.37, 55.63, 37.37, 27.12, 11.07; IR (ATR): *v*_max_ 3267, 2933, 2837, 1669, 1607, 1572, 1535, 1511 cm^−1^; HRMS (+ESI): Found m/z 438.0647, [M + Na]^+^, C_20_H_18_ClN_3_O_3_SNa required 438.0650.

General synthetic procedure E for alkynyl quinazolinones 13a–c

To a solution of the appropriate thiol compound **8** (1.0 equivalent) in dimethyl sulfoxide (DMSO)/acetonitrile (ACN) (1:9), propargyl bromide (1.2 equivalent) and potassium carbonate (3.0 equivalent) was added. The reaction mixture was stirred at room temperature for 8 h. After completion, water was added to the reaction mixture and the product was extracted with EtOAc (3 × 15 mL). The combined organic extracts were washed with brine, dried over anhydrous magnesium sulfate and concentrated in vacuo to afford the final solid product **13**.

3-Cyclopropyl-2-(prop-2-yn-1-ylthio)quinazolin-4(3*H*)-one (**13a**)

The title compound **13a** was synthesised from thiol **8a** (0.300 g, 1.37 mmol), propargyl bromide (0.125 mL, 1.64 mmol) and potassium carbonate (0.570 g, 4.11 mmol) following general synthetic procedure E. The product was obtained as a white solid (0.156 g, 44%); mp 138.6–152.4 °C; ^1^H NMR (400 MHz, DMSO-*d*_6_) δ 8.05 (dd, *J* = 7.9 Hz, 1H), 7.75–7.79 (m, 1H), 7.54 (d, 1H), 7.42–7.46 (m, 1H), 4.09 (s, 2H), 3.21 (t, *J* = 2.6 Hz, 1H), 2.88–2.93 (m, 1H), 1.19–1.24 (m, 2H), 0.94–0.98 (m, 2H). ^13^C NMR (100 MHz, DMSO-*d*_6_) δ 161.88, 158.21, 146.89, 134.96, 126.79, 126.37, 126.27, 119.94, 79.96, 74.42, 26.84, 20.60, 11.03; IR (ATR): *v*_max_ 3232, 1672, 1608, 1539 cm^−1^; HRMS (+ESI): Found m/z 279.0562, [M + Na]^+^, C_14_H_12_N_2_OSNa required 279.0563.

3-Phenyl-2-(prop-2-yn-1-ylthio)quinazolin-4(3*H*)-one (**13b**)

The title compound **13b** was synthesised from thiol **6b** (0.200 g, 0.786 mmol), propargyl bromide (0.0713 mL, 0.943 mmol) and potassium carbonate (0.326 g, 2.36 mmol) following general synthetic procedure E. The product was obtained as a white solid (195 mg, 85%); mp 179.7–194.1 °C; ^1^H NMR (400 MHz, DMSO-*d*_6_) δ 8.03 (dd, *J* = 8.0, 1.6 Hz, 1H), 7.88 (ddd, *J* = 8.5, 7.2, 1.6 Hz, 1H), 7.68 (dd, *J* = 8.3, 1.1 Hz, 1H), 7.61–7.58 (m, 3H), 7.51–7.46 (m, 3H), 4.01 (d, *J* = 2.6 Hz, 2H), 3.18 (t, *J* = 2.6 Hz, 1H), 2.55 (s, 1H); ^13^C NMR (100 MHz, DMSO-*d*_6_) δ 161.13, 156.19, 147.57, 136.03, 135.47, 130.57, 130.03, 129.93, 127.09, 126.69, 126.65, 120.09, 79.72, 74.50, 40.63, 40.42, 40.21, 40.00, 39.79, 39.58, 39.37, 20.88. IR (ATR): *v*_max_ 3228, 1990, 1677, 1605, 1533 cm^−1^; HRMS (+ESI): Found m/z 315.0562, [M + Na]^+^, C_17_H_12_N_2_OSNa required 315.0563.

6-Chloro-3-cyclopropyl-2-(prop-2-yn-1-ylthio)quinazolin-4(3*H*)-one (**13c**)

The title compound **13c** was synthesised from thiol **6c** (0.200 g, 0.791 mmol), propargyl bromide (0.0720 mL, 0.950 mmol) and potassium carbonate (0.328 g, 2.37 mmol) following general synthetic procedure E. The product was obtained as a white solid (82 mg, 94%); mp 179.7–194.1 °C; ^1^H NMR (400 MHz, DMSO-*d*_6_) δ 7.97 (d, *J* = 2.5 Hz, 1H), 7.80 (dd, *J* = 8.7, 2.5 Hz, 1H), 7.56 (d, *J* = 8.7 Hz, 1H), 4.09 (s, 2H), 3.22 (t, 1H), 2.88–2.94 (m, 1H), 1.19–1.24 (m, 2H), 0.95–0.99 (m, 2H); ^13^C NMR (100 MHz, DMSO-*d*_6_) δ 160.92, 159.11, 145.61, 134.99, 130.31, 128.54, 125.73, 121.22, 79.82, 74.52, 26.99, 20.69, 11.00; IR (ATR): *v*_max_ 3245, 2184, 1672, 1574, 1543 cm^−1^; HRMS (+ESI): Found m/z 313.0172 [M + Na]^+^, C_14_H_11_ClN_2_OSNa required 313.0173.

General synthetic procedure F for 2-bromoacetamide derivatives **15a**–**c**

A solution of appropriate aniline **10** (1.0 equivalent), bromoacetyl bromide **11b** (1.2 equivalent) and triethylamine (1.1 equivalent) in DCM (20 mL) was stirred at room temperature overnight. 10% HCl (10 mL) was then added to the reaction mixture. The product was extracted into DCM (3 × 30 mL), and the combined organic extracts washed with brine, dried over anhydrous magnesium sulfate and concentrated in vacuo. The resulting crude compound was purified with flash column chromatography on silica gel using DCM/MeOH as eluent to afford the acetamide **12** as a solid powder.

2-Bromo-*N*-(4-methoxyphenyl)acetamide (**15a**)

This title compound **15a** was synthesised from 4-methoxyaniline **10a** (1.00 g, 8.12 mmol), bromoacetyl bromide **11b** (0.852 mL, 9.74 mmol) and triethylamine (1.24 mL, 8.93 mmol) following general synthetic procedure F. The product was obtained as an off-white solid (0.908 g, 46%); mp 129.7–131.9 °C; ^1^H NMR (400 MHz, DMSO-*d*_6_) δ 10.24 (s, 1H), 7.45–7.54 (m, 2H), 6.86–6.97 (m, 2H), 4.01 (s, 2H), 3.73 (s, 3H); ^13^C NMR (100 MHz, DMSO-*d*_6_) δ 164.71, 156.06, 132.18, 121.22, 114.45, 55.65, 30.90; IR (ATR): *v*_max_ 3280, 3165, 2946, 2052, 1655, 1620, 1542 cm^−1^; HRMS (+ESI): Found m/z 265.9788, [M + Na]^+^, C_9_H_10_BrNO_2_Na required 265.9787.

2-Bromo-*N*-phenylacetamide (**15b**)

The title compound **15b** was synthesised from aniline **10b** (1 mL, 10.7 mmol), bromoacetyl bromide **11b** (1.12 mL, 12.9 mmol) and triethylamine (1.65 mL, 11.8 mmol) following general synthetic procedure F. The product was obtained as an off-white solid (0.465 g, 20%); mp 134.7–165.2 °C; ^1^H NMR (400 MHz, DMSO-*d*_6_) δ 10.37 (s, 1H), 7.54–7.62 (m, 2H), 7.34 (t, *J* = 8.0 Hz, 2H), 7.09 (t, 7.2, 1.3 Hz, 1H), 4.04 (s, 1H); ^13^C NMR (100 MHz, DMSO-*d*_6_) δ 165.24, 139.07, 129.34, 124.30, 119.97, 119.69, 30.89; IR (ATR): 3138, 2270, 2223, 2169, 1991, 1699, 1671, 1604, 1543, 1514; IR (ATR): *v*_max_ 3272, 3215, 3148, 3100, 2052, 1655, 1608, 1553 cm^−1^; HRMS (+ESI): Found m/z 235.9683, [M + Na]^+^, C_8_H_8_BrNONa required 235.9681.

2-Bromo-*N*-(4-nitrophenyl)acetamide (**15c**)

This title compound **15c** was synthesised from 4-nitroaniline **10c** (0.70 mL, 7.24 mmol), bromoacetyl bromide **11b** (0.852 mL, 9.74 mmol) and K_2_CO_3_ (1.5 g, 8.93 mmol) following general synthetic procedure F. The product was obtained as a yellow solid (0.908 g, 46%); mp 152.7–175.6 °C; ^1^H NMR (400 MHz, DMSO-*d*_6_) δ 10.98 (s, 1H), 8.21–8.32 (m, 2H), 7.80–7.88 (m, 2H), 4.11 (s, 2H); ^13^C NMR (100 MHz, DMSO-*d*_6_) δ 166.26, 145.16, 143.11, 125.55, 119.51, 30.65. IR (ATR): *v*_max_ 3275, 3227, 3167, 3106, 2945, 1677, 1620, 1599, 1561 cm^−1^; HRMS (+ESI): Found m/z 280.9533, [M + Na]^+^, C_8_H_7_BrN_2_O_3_Na required 280.9532.

General synthetic procedure G for quinazolinone-1,2,3-triazole-phenylacetamide derivatives 6a–e

To a solution of appropriate alkyne **13** (1.0 equivalent) and 2-bromoacetamide **15b**–**d** (1.2 equivalent) in DMF/H_2_O (2:1), sodium azide (1.2 equivalent), CuI (0.2 equivalent) and sodium ascorbate (0.1 equivalent) were added. The reaction was heated to reflux overnight. After the reaction was completed, ice-cold water was added to the reaction mixture and the product was extracted using EtOAc (3 × 15 mL). The combined organic extracts were washed with brine, dried over anhydrous magnesium sulfate and concentrated in vacuo. The resulting crude compound was purified with flash column chromatography on silica gel using *n*-hexane/EtOAc as eluent to afford the solid product.

2-(4-(((3-Cyclopropyl-4-oxo-3,4-dihydroquinazolin-2-yl)thio)methyl)-1*H*-1,2,3-triazol-1-yl)-*N*-phenylacetamide (**6a**)

The title compound **6a** was synthesised from alkyl quinazolinone **13a** (0.050 g, 0.195 mmol), 2-bromoacetamide **12b** (0.0498 g, 0.234 mmol) and sodium azide (0.0150 g, 0.234 mmol) following general synthetic procedure G. The product was obtained as an off-white solid (14 mg, 42%); mp 171.3–206.0 °C; ^1^H NMR (400 MHz, DMSO-*d*_6_) δ 10.46 (s, 1H), 8.14 (s, 1H), 8.05 (dd, *J* = 8.2 Hz, 1H), 7.76–7.80 (m, 1H), 7.62 (dd, *J* = 8.2 Hz, 1H), 7.54 (dd, *J* = 4.0 Hz, 2H), 7.42–7.45 (m, 1H), 7.31–7.35 (m, 2H), 7.07–7.10 (m, 1H), 5.30 (s, 2H), 5.58 (s, 2H), 2.87–2.90 (m, 1H), 1.18–1.24 (m, 2H), 0.93–0.97 (m, 2H); ^13^C NMR (100 MHz, DMSO-*d*_6_) δ 164.62, 162.08, 159.11, 147.00, 142.85, 138.74, 134.98, 129.40, 126.74, 126.34, 126.29, 126.04, 124.34, 119.89, 119.71, 52.65, 26.87, 11.11; IR (ATR): *v*_max_ 3138, 2270, 2223, 2169, 1991, 1699, 1671, 1604, 1543, 1514 cm^−1^; HRMS (+ESI): Found m/z 433.1448, [M + H]^+^, C_22_H_22_N_6_O_2_S required 433.1441.

2-(4-(((3-Cyclopropyl-4-oxo-3,4-dihydroquinazolin-2-yl)thio)methyl)-1*H*-1,2,3-triazol-1-yl)-*N*-(4-methoxyphenyl)acetamide (**6b**)

The title compound **6b** was synthesised from alkyl quinazolinone **13a** (0.0500 g, 0.195 mmol), 2-bromoacetamide **12c** (0.0570 g, 0.234 mmol) and sodium azide (0.0152 g, 0.234 mmol) following general synthetic procedure G. The product was obtained as a green solid (50 mg, 55%); mp 143.6–168.0 °C; ^1^H NMR (400 MHz, DMSO-*d*_6_) δ 10.30 (s, 1H), 8.15 (s, 1H), 8.05 (dd, *J* = 1.5 Hz, 1H), 7.76–7.80 (m, 1H), 7.62 (dd, *J* = 8.2, 1.1 Hz, 1H), 7.42–7.51 (m, 3H), 6.88–6.92 (m, 2H), 5.26 (s, 2H), 4.57 (s, 2H), 3.72 (s, 3H), 2.85–2.90 (m, 1H), 1.18–1.23 (m, 2H), 0.92–0.97 (m, 2H); ^13^C NMR (100 MHz, DMSO-*d*_6_) δ 170.36, 164.49, 159.17, 156.81, 148.02, 136.56, 133.47, 132.23, 126.75, 126.35, 126.21, 121.35, 121.22, 114.47, 114.43, 55.64, 52.62, 51.69, 26.86, 11.10; IR (ATR): *v*_max_ 3260, 3142, 3081, 2917, 2838, 2107, 2016, 1664, 1607, 1547, 1510 cm^−1^; HRMS (+ESI): Found m/z 463.1545, [M + H]^+^, C_23_H_23_N_6_O_3_S required 463.1547.

2-(4-(((6-Chloro-3-cyclopropyl-4-oxo-3,4-dihydroquinazolin-2-yl)thio)methyl)-1*H*-1,2,3-triazol-1-yl)-*N*-(4-methoxyphenyl)acetamide (**6c**)

The title compound **6c** was synthesised from alkyl quinazolinone **13c** (0.0500 g, 0.172 mmol), 2-bromoacetamide **12c** (0.0500 g, 0.206 mmol) and sodium azide (0.0133 g, 0.206mmol) following general synthetic procedure G. The product was obtained as a green solid (28 mg, 33%); mp 179.7–194.1 °C; ^1^H NMR (400 MHz, DMSO-*d*_6_) δ 10.30 (s, 1H), 8.15 (s, 1H), 8.05 (dd, *J* = 8.0, 1.5 Hz, 1H), 7.76–7.80 (m, 1H), 7.59–7.66 (m, 1H), 7.44– 7.50 (m, 2H), 6.86–6.95 (m, 2H), 5.26 (s, 2H), 4.57 (s, 2H), 3.72 (s, 3H), 2.87–2.91 (m, 1H), 1.18–1.22 (m, 2H), 0.83–0.87 (m, 2H); ^13^C NMR (100 MHz, DMSO-*d*_6_) δ 164.07, 156.01, 145.74, 134.92, 131.91, 121.37, 121.24, 114.47, 114.42, 55.64, 51.68, 27.02, 11.06; IR (ATR): *v*_max_ 3267, 1668, 1608, 1550, 1510 cm^−1^; HRMS (+ESI): Found m/z 519.0976, [M + Na]^+^, C_23_H_21_ClN_6_O_3_SNa required 519.0977.

*N*-(4-Methoxyphenyl)-2-(4-(((4-oxo-3-phenyl-3,4-dihydroquinazolin-2-yl)thio)methyl)-1*H*-1,2,3-triazol-1-yl)acetamide (**6d**)

The title compound **6d** was synthesised from alkyl quinazolinone **13b** (0.100 g, 0.360 mmol), 2-bromoacetamide **12c** (0.1054 g, 0.432 mmol) and sodium azide (0.0280 g, 0.432 mmol) following general synthetic procedure G. The product was obtained as a pale green solid (33 mg, 37%); mp 187.3–208.3 °C; ^1^H NMR (400 MHz, DMSO-*d*_6_) δ 10.29 (s, 1H), 8.09–8.13 (m, 2H), 7.85–7.89 (m, 1H), 7.74 (dd, *J* = 4.0 Hz, 1H), 7.55–7.60 (m, 2H), 7.50–7.53 (m, 1H), 7.45–7.49 (m, 4H), 6.88–6.92 (m, 2H), 5.24 (s, 2H), 4.49 (s, 2H), 3.72 (s, 3H); ^13^C NMR (100 MHz, DMSO-*d*_6_) δ 164.06, 161.19, 157.16, 156.00, 147.71, 136.21, 135.41, 131.94, 130.43, 129.98, 129.89, 127.05, 126.74, 126.55, 126.03, 121.22, 120.09, 114.47, 55.63, 52.59, 27.29; IR (ATR): *v*_max_ 3138, 2270, 2223, 2169, 1991, 1699, 1671, 1604, 1543, 1514 cm^−1^; HRMS (+ESI): Found m/z 521.1362, [M + Na]^+^, C_26_H_22_N_6_O_3_SNa required 521.1366.

2-(4-(((3-Cyclopropyl-4-oxo-3,4-dihydroquinazolin-2-yl)thio)methyl)-1*H*-1,2,3-triazol-1-yl)-*N*-(4-nitrophenyl)acetamide (**6e**)

The title compound **6e** was synthesised from alkyl quinazolinone **13a** (0.100 g, 0.390 mmol), 2-bromoacetamide **12d** (0.151 g, 0.585 mmol) and sodium azide (0.0380 g, 0.585 mmol) following general synthetic procedure G. The product was obtained as a light green solid (15 mg, 10%); mp 208.4–244.0 °C; ^1^H NMR (400 MHz, DMSO-*d*_6_) δ 11.05 (s, 1H), 8.23–8.26 (m, 2H), 8.18 (s, 1H), 8.14 (s, 1H), 8.04–8.06 (dd, *J* = 8.0, 1.6 Hz, 1H), 7.83–7.76 (m, 1H), 7.62 (d, *J* = 8.1, 1H), 7.46–7.42 (m, 1H), 5.39 (s, 2H), 4.58 (s, 2H), 2.86–2.91 (m, 1H), 1.18–1.23 (m, 2H), 0.93–0.97 (m, 2H); ^13^C NMR (100 MHz, DMSO-*d*_6_) δ 165.82, 161.99, 159.13, 147.03, 144.96, 143.07, 134.91, 126.75, 126.36, 126.23, 126.10, 125.59, 119.97, 119.50, 52.79, 26.92, 26.86, 11.11; IR (ATR): *v*_max_ 3445, 3162, 3017, 2178, 1711, 1666, 1629, 1553, 1512 cm^−1^; HRMS (+ESI): Found m/z 500.1109, [M + Na]^+^, C_22_H_19_N_7_O_4_SNa required 500.1111.

### 3.3. PQS Inhibition Assay

The assay for PqsR inhibition activity was performed using the PAO1 *P. aeruginosa* strain carrying the PqsR-regulated *pqsA* promoter fused to *gfp*. The compounds were dissolved in 100% DMSO to make 20 mM stock solutions. The test compounds (serially diluted with medium) were then incubated with overnight cultures of PAO1-*pasA-gfp* using MHB (Mueller–Hinton Broth) in 96-well plates at 37 °C with intermittent shaking. Readings were taken at 30 min intervals for at least 8 h and both GFP fluorescence and OD_600_ were recorded. The fluorescence values shown in the graph were normalised with respect to OD_600_. Negative control refers to the medium containing DMSO (0.5%) as the highest concentration of the test compound. The *pqs* inhibition assay was carried out in triplicate manner [21].

### 3.4. Biofilm Inhibition Assay

A single colony of *P. aeruginosa* was cultured in Mueller–Hinton Broth (MHB) at 37 °C with shaking at 120 rpm for 24 h. The resulting bacterial culture was washed twice with MHB with centrifugation after each wash. The bacterial solution was then diluted with fresh MHB to a turbidity of OD_660_ = 0.1 in MHB (equivalent to 10^8^ colony-forming unit (CFU)/mL of bacteria), followed by diluting to 10^6^ CFU/mL in MHB. 100 µL of the bacterial solution was added to wells of a flat-bottom 96-well plate (Costar) containing 100 µL serially diluted test compound. After incubation at 37 °C for 18 h, loosely bound cells were washed away with 1× phosphate-buffered saline (PBS, pH 7.4). Biofilms adhered to the plate substratum were quantified, using crystal violet staining as described previously [22,23]. Untreated bacteria were used as a negative control, where the percentage of biofilm mass reflected 100% biofilm growth. The experiment was performed in triplicate.

## 4. Conclusions

In conclusion, GOLD docking studies were carried out in order to guide the synthesis of quinazolinone analogues targeting the PqsR receptor in *P. aeruginosa*. Modifications of two quinazolinone-based scaffolds led to the synthesis of a library of 16 quinazolinone analogues using three different synthetic pathways. Eleven quinazolinone-based amides **5a**–**i** were generated via acid–amine coupling using quinazolinone carboxylic acids **9a**–**d** with substituted amines. Another two quinazolinone-based amides **5j** and **5k** were synthesised via a nucleophilic substitution of 2-chloroacetamide **12a** using thiol compounds **8a**–**d**. Furthermore, quinazolinone-1,2,3-triazole-phenylacetamides **6a**–**e** were synthesised utilising 1,3-dipolar cycloaddition of alkyl quinazolinones **13a**–**c** and 2-bromoacetamides **12b**–**d**.

In vitro *pqs* inhibition assays of compounds **6a**–**e** identified that introducing the methoxy electron-donating group on the 4-position of the terminal phenyl ring (**6b**) could play a significant role in *pqs* inhibition. The *pqs* inhibition results of **6a** and **6e** emphasise this as analogues without a substituent or bearing an electron-withdrawing nitro group, respectively, possessed lower levels of *pqs* inhibition. This suggests that the higher levels of *pqs* inhibition could be due to the hydrogen bonding interactions with GLN194, LEU207, THR265 and SER196, pi-sulfur bonds with PHE221 and hydrophobic interactions with TYR258 and LEU197, as shown by in silico molecular docking studies.

## Figures and Tables

**Table 1:** Yields of quinazolinone-based amides **5a**–**i**.

	n	R^1^	R^2^	R^3^	R^4^	Yield (%)
**5a**	1	H	Cyclopropane	H	Butyl	17
**5b**	1	H	Cyclopropane	H	Benzyl	30
**5c**	1	H	Phenyl	H	Benzyl	15
**5d**	1	Cl	Cyclopropane	H	Benzyl	14
**5e**	1	H	Cyclopropane	H	Anisole	64
**5f**	2	H	Phenyl	H	Anisole	57
**5g**	1	H	Cyclopropane	-	Morpholine	49
**5h**	1	H	Phenyl	-	Morpholine	32
**5i**	1	Cl	Cyclopropane	-	Morpholine	97

**Table 2:** Yields of quinazolinone-1,2,3-triazole-phenylacetamides **6a**–**e**.

	R^1^	R^2^	R^3^	Yield (%)
**6a**	H	Cyclopropane	H	42
**6b**	H	Cyclopropane	Methoxy	55
**6c**	Cl	Cyclopropane	Methoxy	33
**6d**	H	Phenyl	Methoxy	37
**6e**	H	Cyclopropane	Nitro	10

**Table 3:** Percentage PQS inhibition on PqsR system of *P. aeruginosa* (PAO1-*pqsA-gfp*) using percentage of green fluorescent protein (GFP) fluorescence at 485 nm. All measurements were performed in triplicates with ±standard deviation from mean.

Compound	Concentration		
	100 μM	50 μM	25 μM
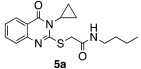	^a^ 62.1 ± 2.9	^a^ 38.6 ± 0.9	^b^ 28.0 ± 1.1
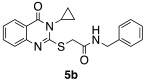	^a^ 51.0 ± 2.9	^a^ 34.8 ± 2.5	^a^ 24.4 ± 1.9
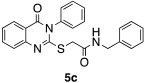	^a^ 46.0 ± 3.7	^a^ 29.8 ± 1.8	^a^ 18.4 ± 5.1
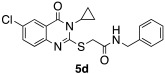	^c^ 49.9 ± 4.1	^b^ 29.5 ± 3.1	^b^ 47.0 ± 8.2
** 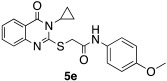 **	^a^ 50.2 ± 3.7	^a^ 34.3 ± 0.8	^a^ 11.7 ± 4.2
** 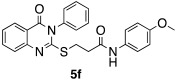 **	^b^ 39.5 ± 8.5	^a^ 11.7 ± 1.0	NA
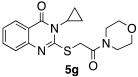	^a^ 35.6 ± 5.2	^b^ 16.2 ± 1.0	^b^ 9.3 ± 1.9
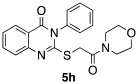	^a^ 39.8 ± 6.5	^a^ 27.5 ± 1.5	^a^ 13.0 ± 1.6
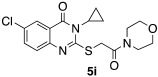	^b^ 33.3 ± 4.9	^b^ 30.3 ± 5.8	^b^ 10.5 ± 4.2
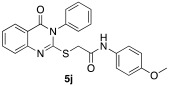	^c^ 44.3 ± 3.8	^b^ 43.0 ± 8.0	^b^ 26.2 ± 7.1
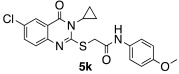	^a^ 58.3 ± 5.9	^a^ 33.0 ± 5.6	^a^ 10.9 ± 0.3
** 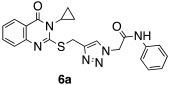 **	NT	NT	^a^ 21.6 ± 3.6
** 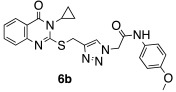 **	^a^ 73.4 ± 0.0	^b^ 72.1 ± 2.4	^b^ 53.7 ± 5.2
** 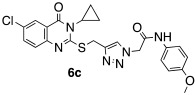 **	^a^ 49.8 ± 1.6	^b^ 36.5± 3.8	^a^ 11.9 ± 3.1
*** 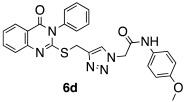 ***	^b^ 60.3 ± 5.3	^b^ 35.1 ± 1.1	^a^ 8.4 ± 0.7
** 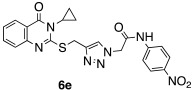 **	^c^ 63.3 ± 2.0	^a^ 37.6 ± 5.5	^a^ 7.3 ± 1.5

^a^ No bacterial growth inhibition; ^b^ growth inhibition between 0 and 10%; ^c^ growth inhibition between 10 and 20%; NA = Not active; NT = Not tested.

## Data Availability

Data are contained within the article and Supplementary Materials.

## Supplementary Materials

The following supporting information can be downloaded at: https://www.mdpi.com/article/10.3390/antibiotics12071227/s1, Figure S1: (A) ^1^H NMR spectrum of compound **6b**. (B) Key ^1^H:^13^C HMBC correlations in compound **6b**; Table S1: Reaction conditions attempted for the synthesis of quinazolinone-based amide **5a**; Table S2: Percentage growth inhibition of *P. aeruginosa* induced by test compounds at 100, 50 and 25 μM; Scheme S1: Synthesis of quinazolinone-based amide **5e**.
