# Supplementary material for: Synthesis of Novel Quinazolinone Analogues for Quorum Sensing Inhibition
Source: Antibiotics (Basel). 2023 Jul 24;12(7):1227. doi: 10.3390/antibiotics12071227 (PMC10376653; doi:10.3390/antibiotics12071227)

Supporting information

Synthesis of Novel Quinazolinone Analogues for Quorum Sensing Inhibition

Sahil Shandil, Tsz Tin Yu *, Shekh Sabir, David StC. Black and Naresh Kumar *

School of Chemistry, The University of New South Wales, Sydney, NSW 2052, Australia; s.shandil@unsw.edu.au (S.S.); s.sabir@student.unsw.edu.au (S.S.); d.black@unsw.edu.au (D.StC.B.)

* Correspondence: tsztin.yu@unsw.edu.au (T.T.Y.); n.kumar@unsw.edu.au (N.K.).

Contents

Supplementary figures for the HMBC spectrum of compound 6b 4
Supplementary scheme and table for optimization of reaction condition in the synthesis of compound 5e 5
Supplementary table for percentage growth inhibition of *P. aeruginosa* induced by test compounds 6
^{1}H NMR and ^{13}C NMR spectra of synthesized compounds 7
N-Butyl-2-((3-cyclopropyl-4-oxo-3,4-dihydroquinazolin-2-yl)thio)acetamide (5a) 7
N-Benzyl-2-((3-cyclopropyl-4-oxo-3,4-dihydroquinazolin-2-yl)thio)acetamide (5b)...........8
N-Benzyl-2-((4-oxo-3-phenyl-3,4-dihydroquinazolin-2-yl)thio)acetamide (**5c**)....................9
N-Benzyl-2-((6-chloro-3-cyclopropyl-4-oxo-3,4-dihydroquinazolin-2-yl)thio)acetamide (**5d**)11
2-((3-Cyclopropyl-4-oxo-3,4-dihydroquinazolin-2-yl)thio)-*N*-(4-methoxyphenyl)acetamide (**5e**) 13
N-(4-Methoxyphenyl)-3-((4-oxo-3-phenyl-3,4-dihydroquinazolin-2-yl)thio)propenamide (**5f**) 14
3-Cyclopropyl-2-((2-morpholino-2-oxoethyl)thio)quinazolin-4(3*H*)-one (5g) 16
2-((2-Morpholino-2-oxoethyl)thio)-3-phenylquinazolin-4(3*H*)-one (**5h**) 17
2-((2-Morpholino-2-oxoethyl)thio)quinazolin-4(3*H*)-one (**5i**)............18
N-(4-Methoxyphenyl)-2-((4-oxo-3-phenyl-3,4-dihydroquinazolin-2-yl)thio)acetamide (5j) 19
2-((6-Chloro-3-cyclopropyl-4-oxo-3,4-dihydroquinazolin-2-yl)thio)-*N*-(4-methoxyphenyl)acetamide (**5k**) 20
2-(4-(((3-Cyclopropyl-4-oxo-3,4-dihydroquinazolin-2-yl)thio)methyl)-1*H*-1,2,3-triazol-1-yl)-*N*-phenylacetamide (**6a**)..21
2-(4-(((3-Cyclopropyl-4-oxo-3,4-dihydroquinazolin-2-yl)thio)methyl)-1*H*-1,2,3-triazol-1-yl)-*N*-(4-methoxyphenyl)acetamide (**6b**).....................22
2-(4-(((6-Chloro-3-cyclopropyl-4-oxo-3,4-dihydroquinazolin-2-yl)thio)methyl)-1*H*-1,2,3-triazol-1-yl)-*N*-(4-methoxyphenyl)acetamide (**6c**)......23
N-(4-Methoxyphenyl)-2-(4-(((4-oxo-3-phenyl-3,4-dihydroquinazolin-2-yl)thio)methyl)-1*H*-1,2,3-triazol-1-yl)acetamide (6d) 24
2-(4-(((3-Cyclopropyl-4-oxo-3,4-dihydroquinazolin-2-yl)thio)methyl)-1*H*-1,2,3-triazol-1-yl)-*N*-(4-nitrophenyl)acetamide (**6e**) 25
3-Cyclopropyl-2-mercaptoquinazolin-4(3*H*)-one (**8a**) 26
2-Mercapto-3-phenylquinazolin-4(3*H*)-one (**8b**) 27

Citation: Shandil, S.; Yu, T.T.; Sabir, S.; Black, D.StC.; Kumar, N. Synthesis of Novel Quinazolinone Analogues for Quorum Sensing Inhibition. *Antibiotics* **2023**, *12*, x. https://doi.org/10.3390/xxxxx

Academic Editor(s): Rok Frlan

Received: 15 June 2023
Revised: 17 July 2023
Accepted: 21 July 2023
Published: date

Copyright: © 2023 by the authors. Submitted for possible open access publication under the terms and conditions of the Creative Commons Attribution (CC BY) license (https://creativecommons.org/licenses/by/4.0/).

6-Chloro-3-cyclopropyl-2-mercaptoquinazolin-4(3*H*)-one (**8c**)....28
2-((3-Cyclopropyl-4-oxo-3,4-dihydroquinazolin-2-yl)thio)acetic acid (**9a**)....29
2-((4-Oxo-3-phenyl-3,4-dihydroquinazolin-2-yl)thio)acetic acid (**9b**)....30
2-((6-Chloro-3-cyclopropyl-4-oxo-3,4-dihydroquinazolin-2-yl)thio)acetic acid (**9c**)....31
3-((4-Oxo-3-phenyl-3,4-dihydroquinazolin-2-yl)thio)propanoic acid (**9d**)....33
2-Chloro-*N*-(4-methoxyphenyl)acetamide (12)....34
3-Cyclopropyl-2-(prop-2-yn-1-ylthio)quinazolin-4(3*H*)-one (13a)....35
3-Phenyl-2-(prop-2-yn-1-ylthio)quinazolin-4(3*H*)-one (**13b**)....37
6-Chloro-3-cyclopropyl-2-(prop-2-yn-1-ylthio)quinazolin-4(3*H*)-one (**13c**)....39
2-Bromo-*N*-(4-methoxyphenyl)acetamide (**15a**)....41
2-Bromo-*N*-phenylacetamide (**15b**)....42
2-Bromo-*N*-(4-nitrophenyl)acetamide (**15c**)....44
High resolution mass spectra (HRMS) of synthesised compounds....45
N-Butyl-2-((3-cyclopropyl-4-oxo-3,4-dihydroquinazolin-2-yl)thio)acetamide (5a)....45
N-Benzyl-2-((3-cyclopropyl-4-oxo-3,4-dihydroquinazolin-2-yl)thio)acetamide (5b)....47
N-Benzyl-2-((4-oxo-3-phenyl-3,4-dihydroquinazolin-2-yl)thio)acetamide (5c)....49
N-Benzyl-2-((6-chloro-3-cyclopropyl-4-oxo-3,4-dihydroquinazolin-2-yl)thio)acetamide (5d)51
N-(4-Methoxyphenyl)-3-((4-oxo-3-phenyl-3,4-dihydroquinazolin-2-yl)thio)propenamide (**5f**)
....53
3-Cyclopropyl-2-((2-morpholino-2-oxoethyl)thio)quinazolin-4(3*H*)-one (**5g**)....55
2-((2-Morpholino-2-oxoethyl)thio)-3-phenylquinazolin-4(3*H*)-one (**5h**)....57
2-((2-Morpholino-2-oxoethyl)thio)quinazolin-4(3*H*)-one (**5i**)....59
N-(4-Methoxyphenyl)-2-((4-oxo-3-phenyl-3,4-dihydroquinazolin-2-yl)thio)acetamide (**5j**)61
2-((6-Chloro-3-cyclopropyl-4-oxo-3,4-dihydroquinazolin-2-yl)thio)-*N*-(4-methoxyphenyl)acetamide (**5k**)....63
2-(4-(((3-Cyclopropyl-4-oxo-3,4-dihydroquinazolin-2-yl)thio)methyl)-1*H*-1,2,3-triazol-1-yl)-*N*-phenylacetamide (**6a**)....65
2-(4-(((3-Cyclopropyl-4-oxo-3,4-dihydroquinazolin-2-yl)thio)methyl)-1*H*-1,2,3-triazol-1-yl)-*N*-(4-methoxyphenyl)acetamide (**6b**)....67
2-(4-(((6-Chloro-3-cyclopropyl-4-oxo-3,4-dihydroquinazolin-2-yl)thio)methyl)-1*H*-1,2,3-triazol-1-yl)-*N*-(4-methoxyphenyl)acetamide (**6c**)....69
N-(4-Methoxyphenyl)-2-(4-(((4-oxo-3-phenyl-3,4-dihydroquinazolin-2-yl)thio)methyl)-1*H*-1,2,3-triazol-1-yl)acetamide (**6d**)....71
2-(4-(((3-Cyclopropyl-4-oxo-3,4-dihydroquinazolin-2-yl)thio)methyl)-1*H*-1,2,3-triazol-1-yl)-*N*-(4-nitrophenyl)acetamide (**6e**)....73
3-Cyclopropyl-2-mercaptoquinazolin-4(3*H*)-one (**8a**)....75
2-Mercapto-3-phenylquinazolin-4(3*H*)-one (**8b**)....77
6-Chloro-3-cyclopropyl-2-mercaptoquinazolin-4(3*H*)-one (**8c**)....78
2-((3-Cyclopropyl-4-oxo-3,4-dihydroquinazolin-2-yl)thio)acetic acid (**9a**)....80
2-((4-Oxo-3-phenyl-3,4-dihydroquinazolin-2-yl)thio)acetic acid (**9b**)....82
2-((6-Chloro-3-cyclopropyl-4-oxo-3,4-dihydroquinazolin-2-yl)thio)acetic acid (**9c**)....84
3-((4-Oxo-3-phenyl-3,4-dihydroquinazolin-2-yl)thio)propanoic acid (**9d**)....86
2-Chloro-*N*-(4-methoxyphenyl)acetamide (**12**)....87
3-Cyclopropyl-2-(prop-2-yn-1-ylthio)quinazolin-4(3*H*)-one (**13a**)....89

3-Phenyl-2-(prop-2-yn-1-ylthio)quinazolin-4(3*H*)-one (**13b**) 91
6-Chloro-3-cyclopropyl-2-(prop-2-yn-1-ylthio)quinazolin-4(3*H*)-one (**13c**) 93
2-Bromo-*N*-(4-methoxyphenyl)acetamide (**15a**) 95
2-Bromo-*N*-phenylacetamide (**15b**) 97
2-Bromo-*N*-(4-nitrophenyl)acetamide (**15c**) 99

Supplementary figures for the HMBC spectrum of compound 6b

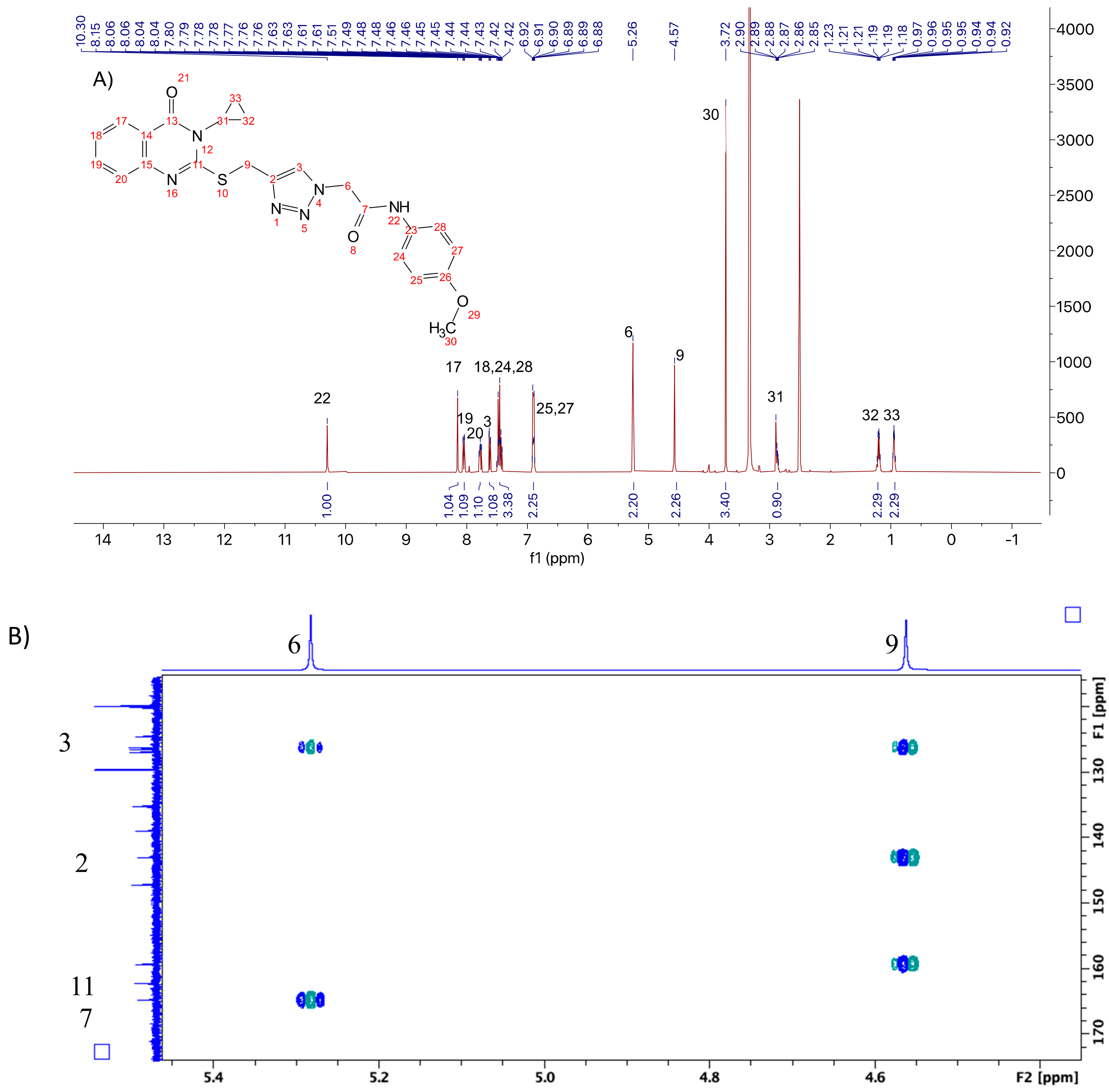

Figure S1. (A) ^{1}H NMR spectrum of compound **6b**. (B) Key ^{1}H:^{13}C HMBC correlations in compound **6b**.

Supplementary scheme and table for optimization of reaction condition in the synthesis of compound 5e

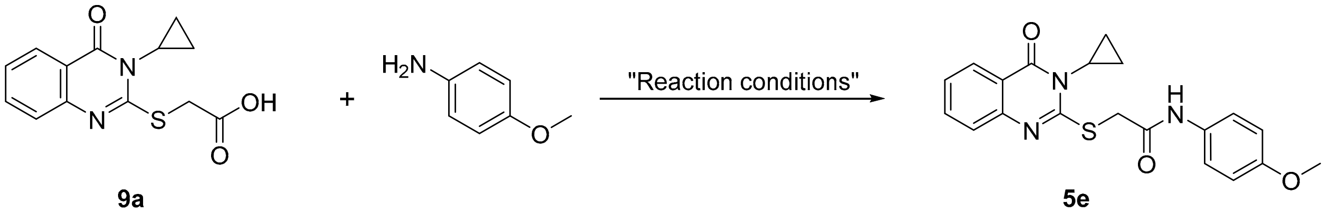

Scheme S1. Synthesis of quinazolinone-based amide **5e**.

Table S1: Reaction conditions attempted for the synthesis of quinazolinone-based amide **5a**.

Entry	Reagents	Solvents	Reaction temperature	Reaction time (h)	Yield (%)
1	$SOCl_2$, Et_3N	DCM	Reflux	1	5
2	EDC-HCl, DIPEA	ACN:DMF (1:1)	r.t.	12	4
3	EDC-HCl, DIPEA	ACN:DMF (1:1)	50 ºC	12	4
4	DCC, DMAP	DCM	r.t.	18	2
5	HATU, Et_3N	DMF	r.t.	12	64

Supplementary table for percentage growth inhibition of *P. aeruginosa* induced by test compounds

Table S2: Percentage growth inhibition of *P. aeruginosa* induced by test compounds at 100, 50 and 25 μM. (All measurements were performed in triplicate with ± standard deviation from mean.)

Compound	Concentration		
	100 μM	50 μM	25 μM
**5a**	0	0	0
**5b**	0	0	0
**5c**	0	0	0
**5d**	10.4 ± 4.1	4.5 ± 1.8	8.9 ± 3.7
**5e**	0	0	0
**5f**	0.5 ± 0.7	0	0
**5g**	0	0.4 ± 2.1	3.1 ± 2.1
**5h**	0	0	0
**5i**	1.2 ± 4.1	0.2 ± 2.0	0.2 ± 3.1
**5j**	16.8 ± 2.3	7.9 ± 8.2	5.8 ± 3.0
**5k**	0	0	0
**6a**	NT	NT	0
**6b**	0	1.0 ± 7.6	1.0 ± 2.4
**6c**	0	0.4 ± 8.4	0
**6d**	7.2 ± 6.2	3.6 ± 3.6	0
**6e**	10.9 ± 4.7	0	0

0 = no growth inhibition; NT = Not tested

^{1}H NMR and ^{13}C NMR spectra of synthesized compounds

N-Butyl-2-((3-cyclopropyl-4-oxo-3,4-dihydroquinazolin-2-yl)thio)acetamide (**5a**)

^{1}H NMR (400 MHz, DMSO-d_6):

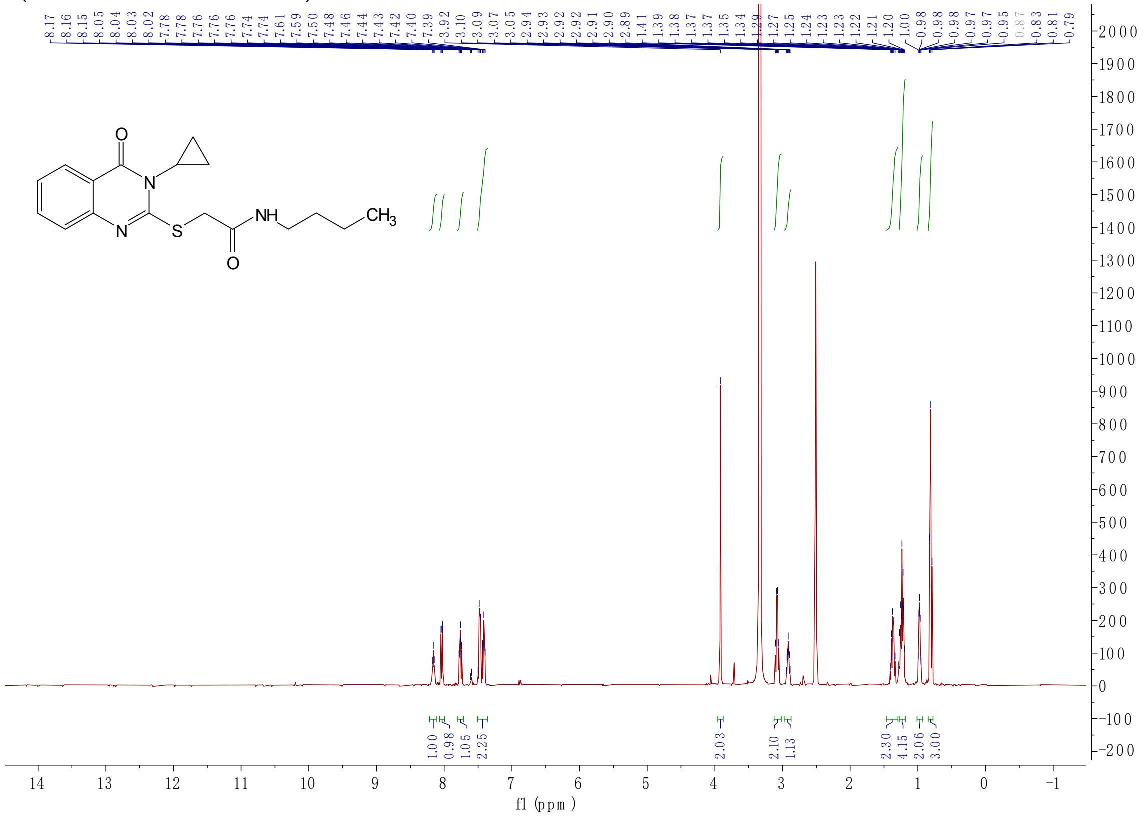

^{13}C NMR (100 MHz, DMSO-d_6):

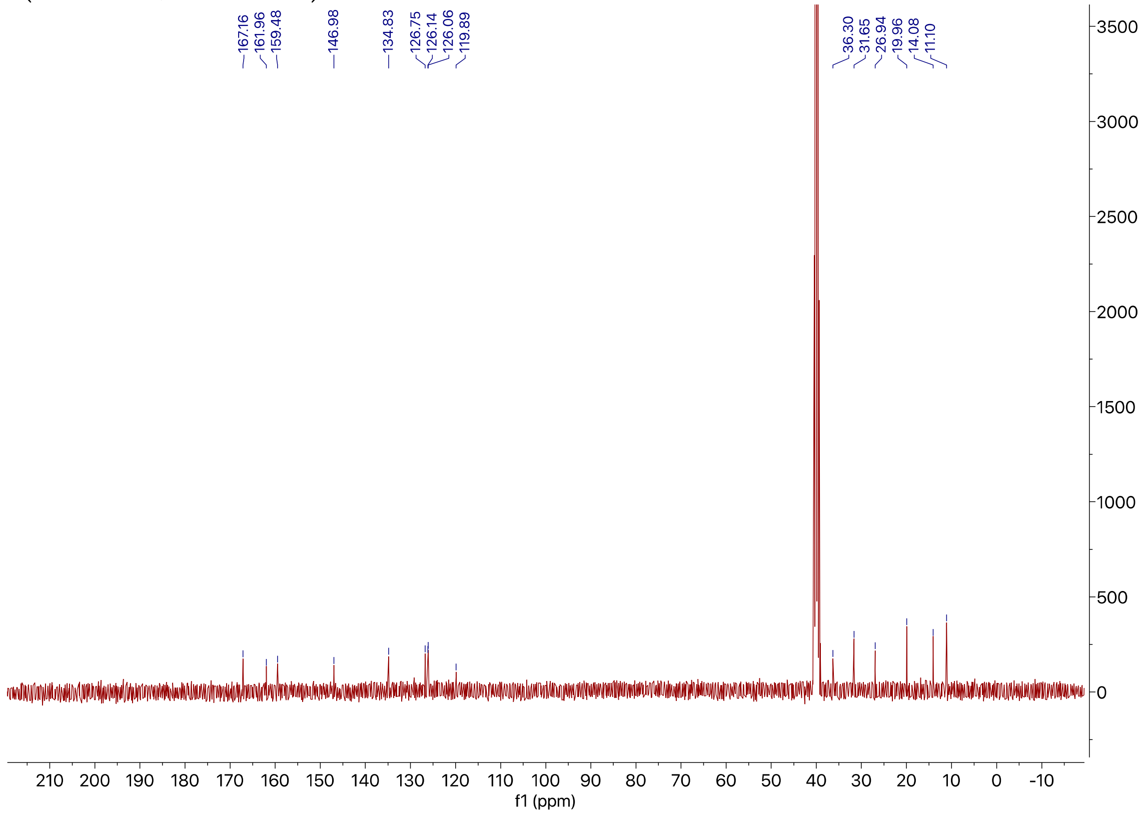

N-Benzyl-2-((3-cyclopropyl-4-oxo-3,4-dihydroquinazolin-2-yl)thio)acetamide (**5b**)

^{1}H NMR (400 MHz, DMSO-d_6):

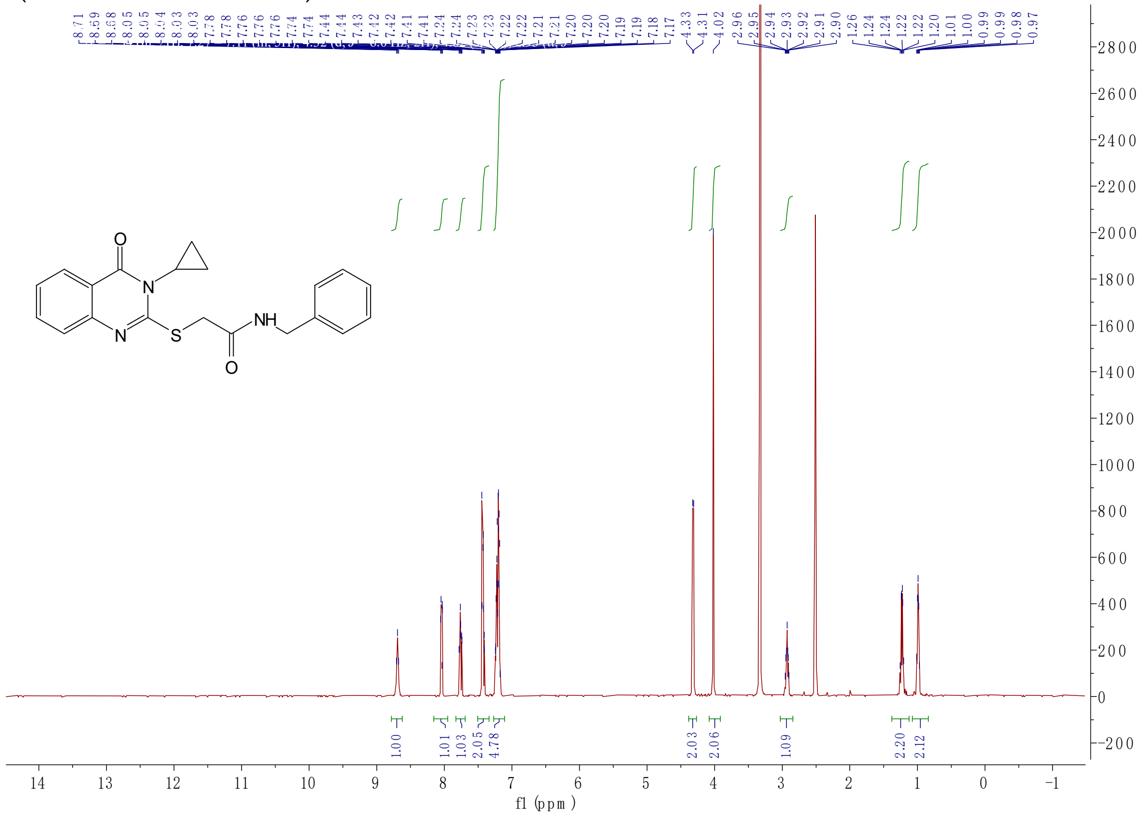

^{13}C NMR (100 MHz, DMSO-d_6):

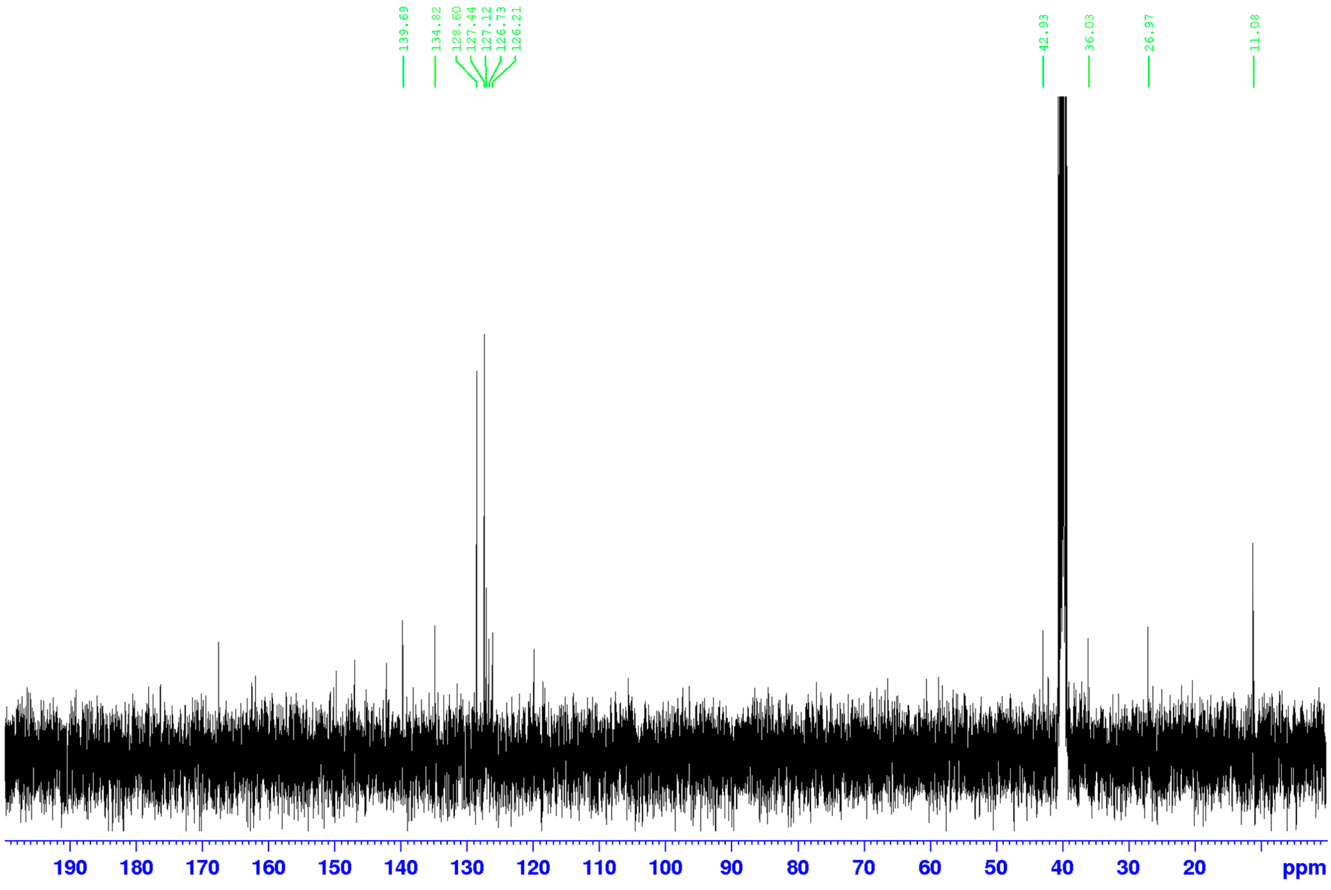

N-Benzyl-2-((4-oxo-3-phenyl-3,4-dihydroquinazolin-2-yl)thio)acetamide (**5c**)

^{1}H NMR (400 MHz, DMSO-*d*$_6$):

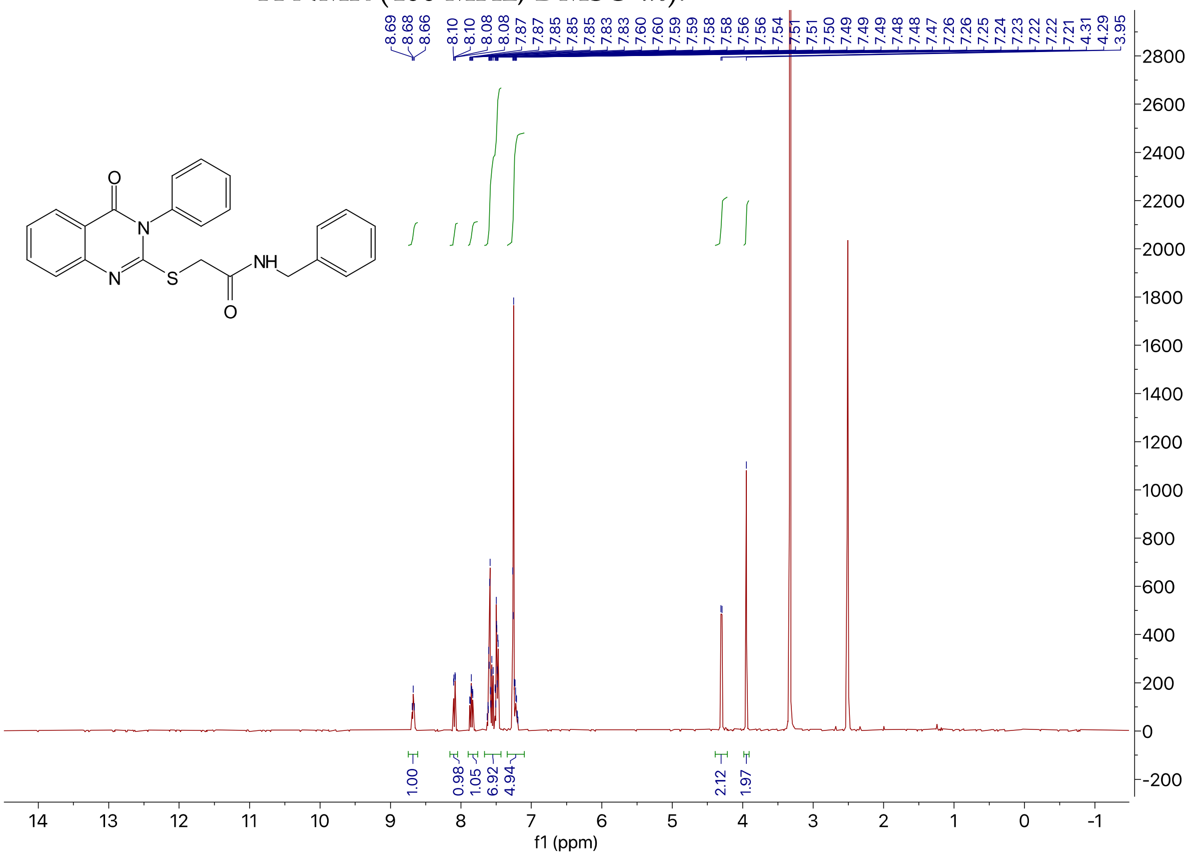

^{13}C NMR (100 MHz, DMSO-*d*$_6$):

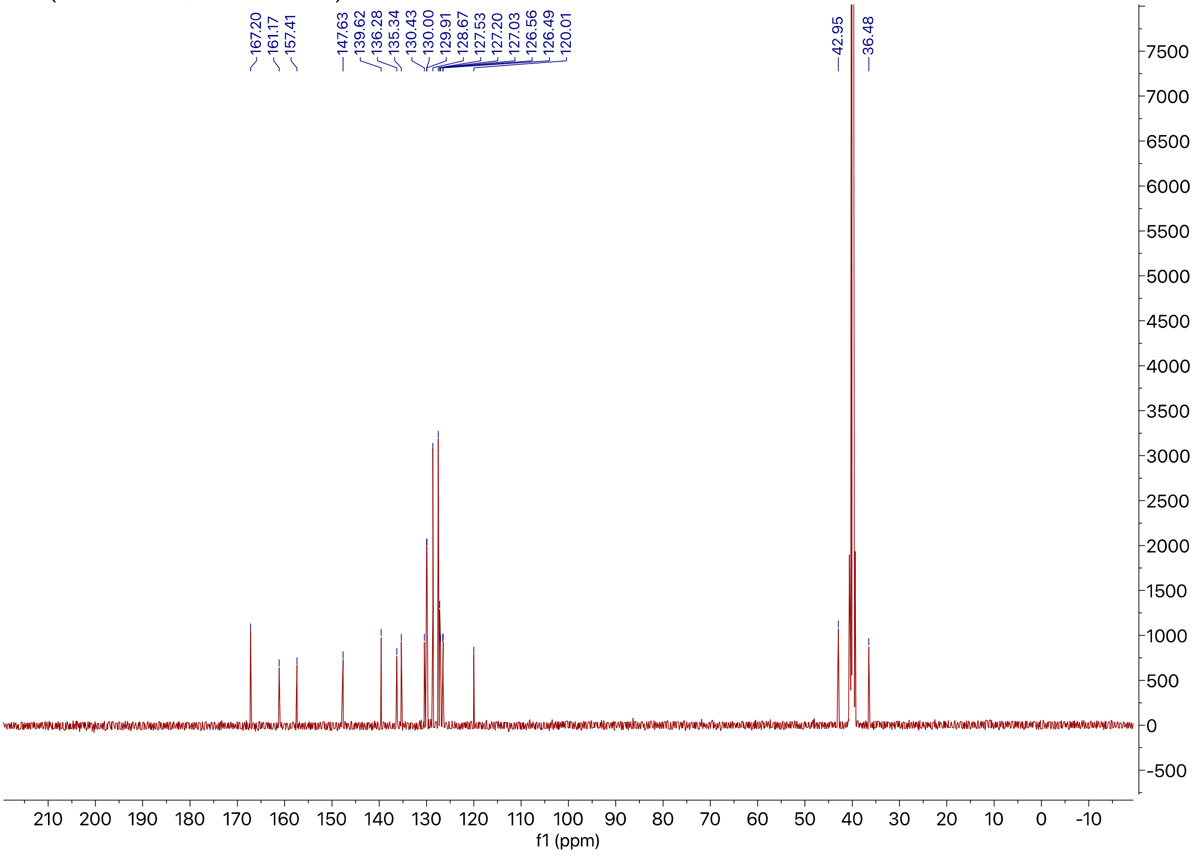

N-Benzyl-2-((6-chloro-3-cyclopropyl-4-oxo-3,4-dihydroquinazolin-2-yl)thio)acetamide (**5d**)

^{1}H NMR (400 MHz, DMSO-d_6):

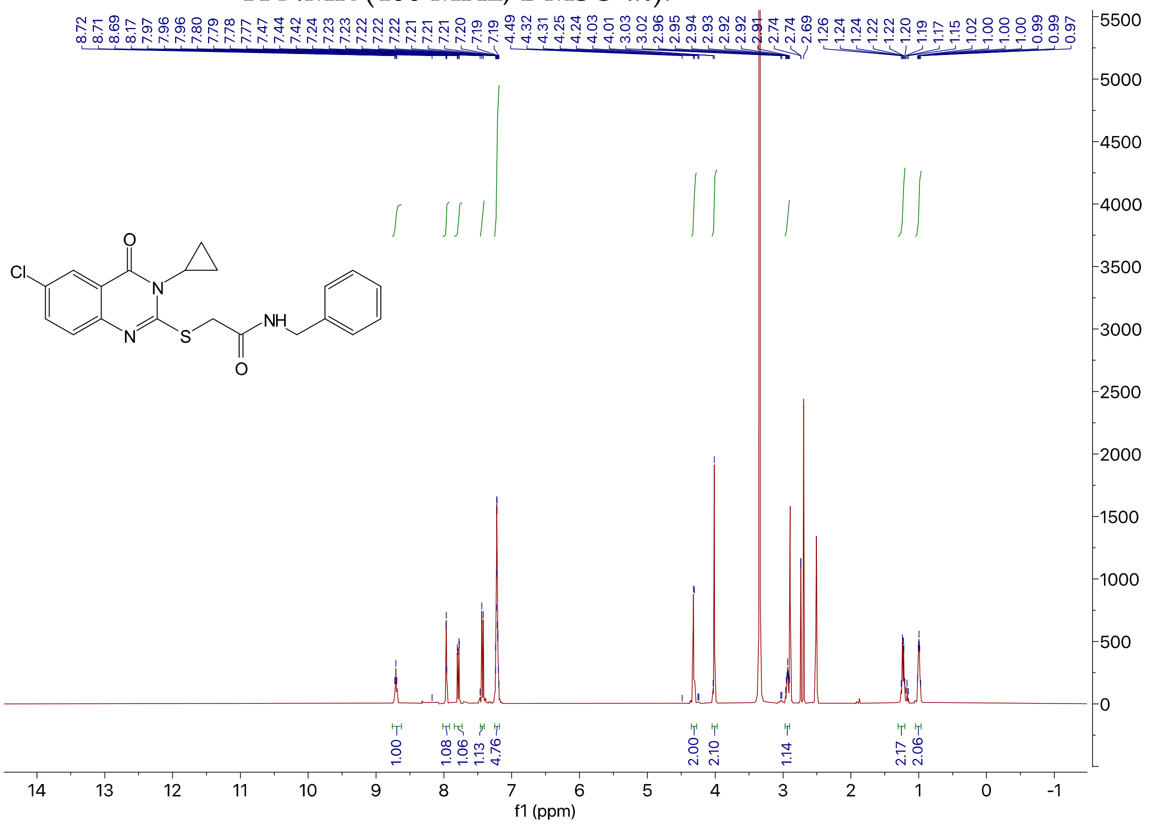

^{13}C NMR (100 MHz, DMSO-d_6):

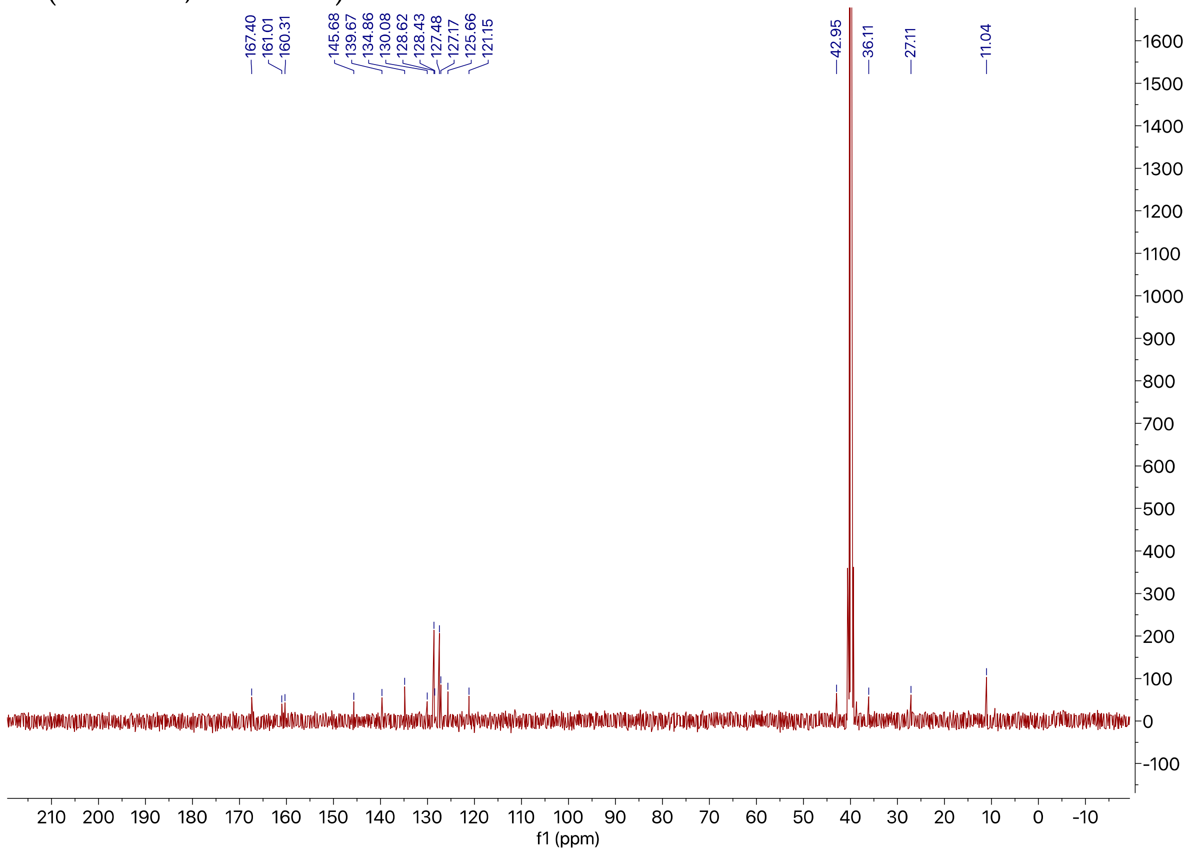

2-((3-Cyclopropyl-4-oxo-3,4-dihydroquinazolin-2-yl)thio)-*N*-(4-methoxyphenyl)acetamide (**5e**)

^{1}H NMR (400 MHz, DMSO-d_6):

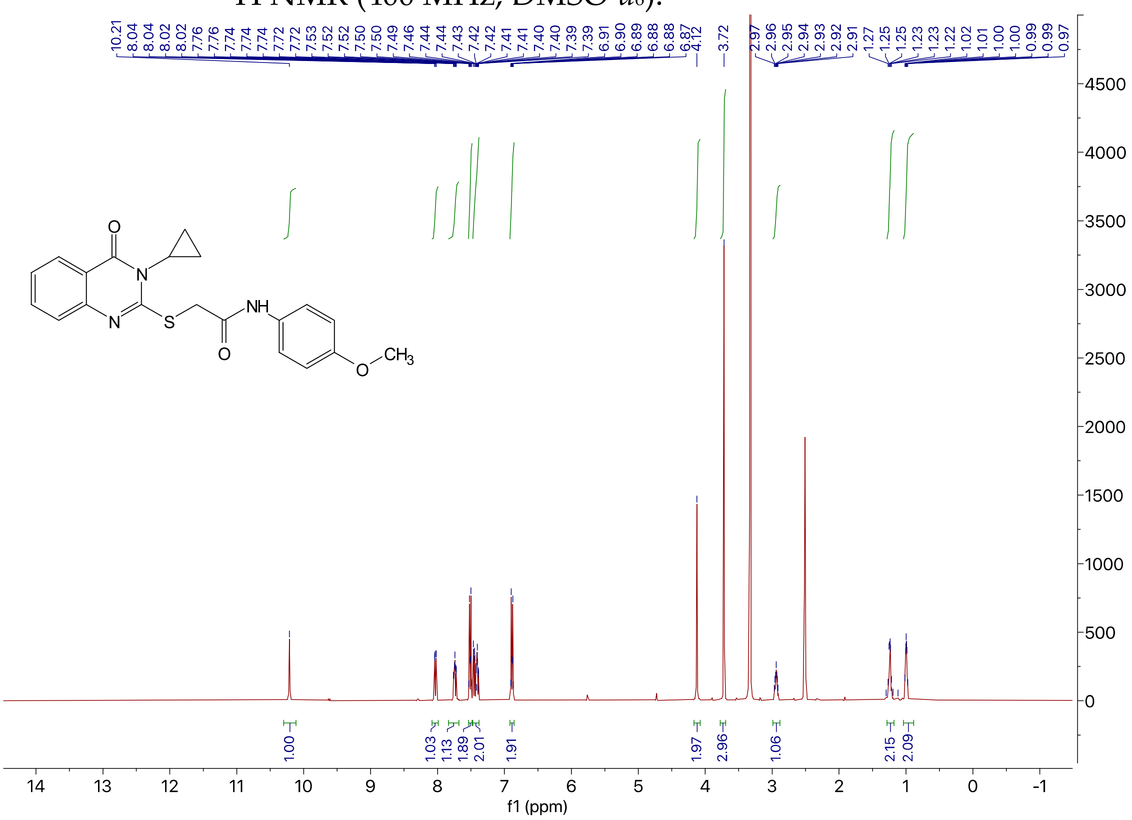

^{13}C NMR (100 MHz, DMSO-d_6):

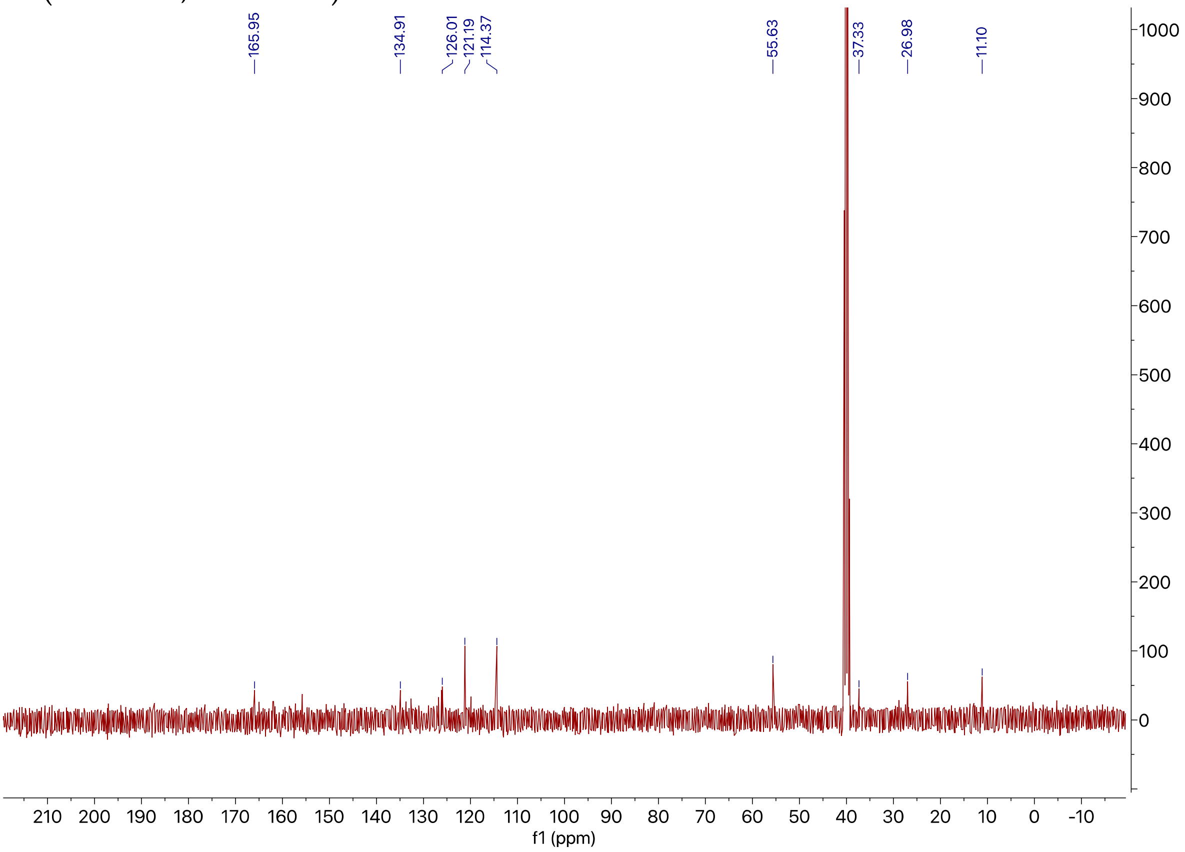

N-(4-Methoxyphenyl)-3-((4-oxo-3-phenyl-3,4-dihydroquinazolin-2-yl)thio)propenamide (**5f**)

^{1}H NMR (400 MHz, DMSO-d_6):

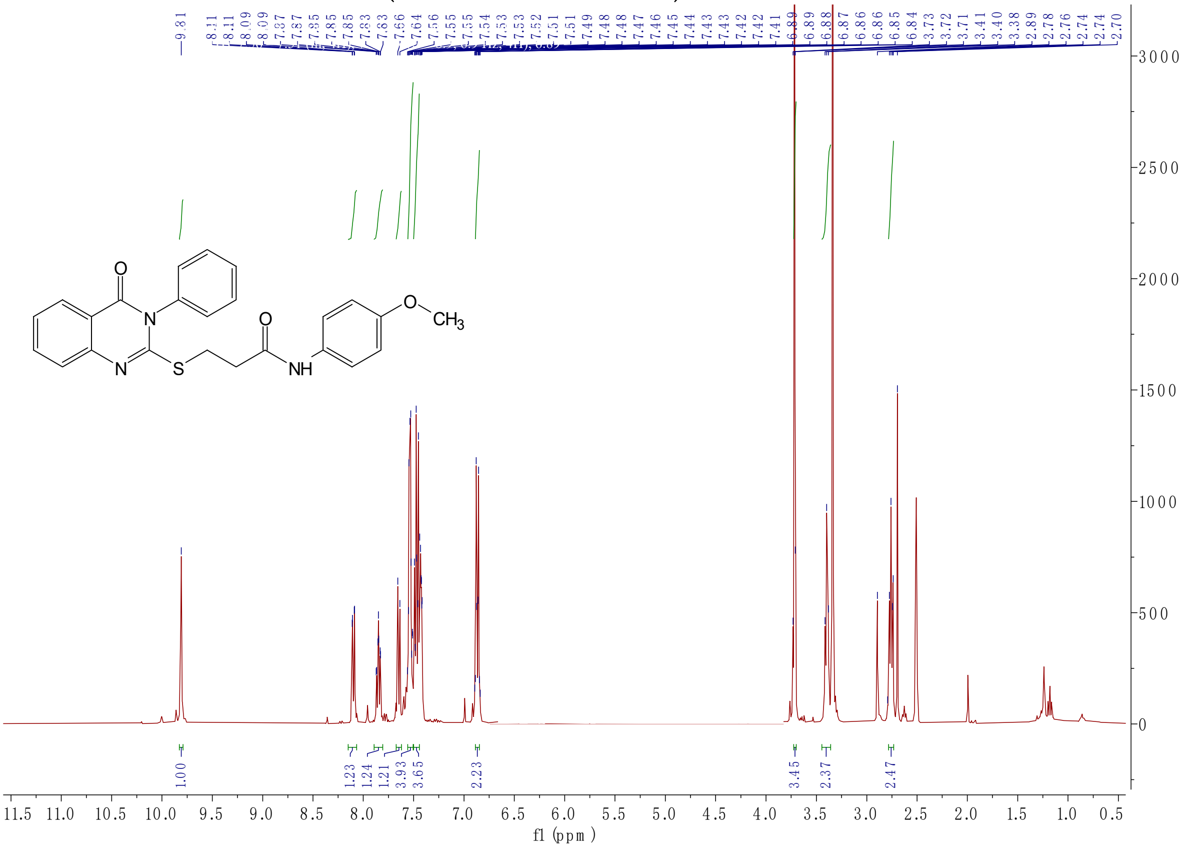

^{13}C NMR (100 MHz, DMSO-d_6):

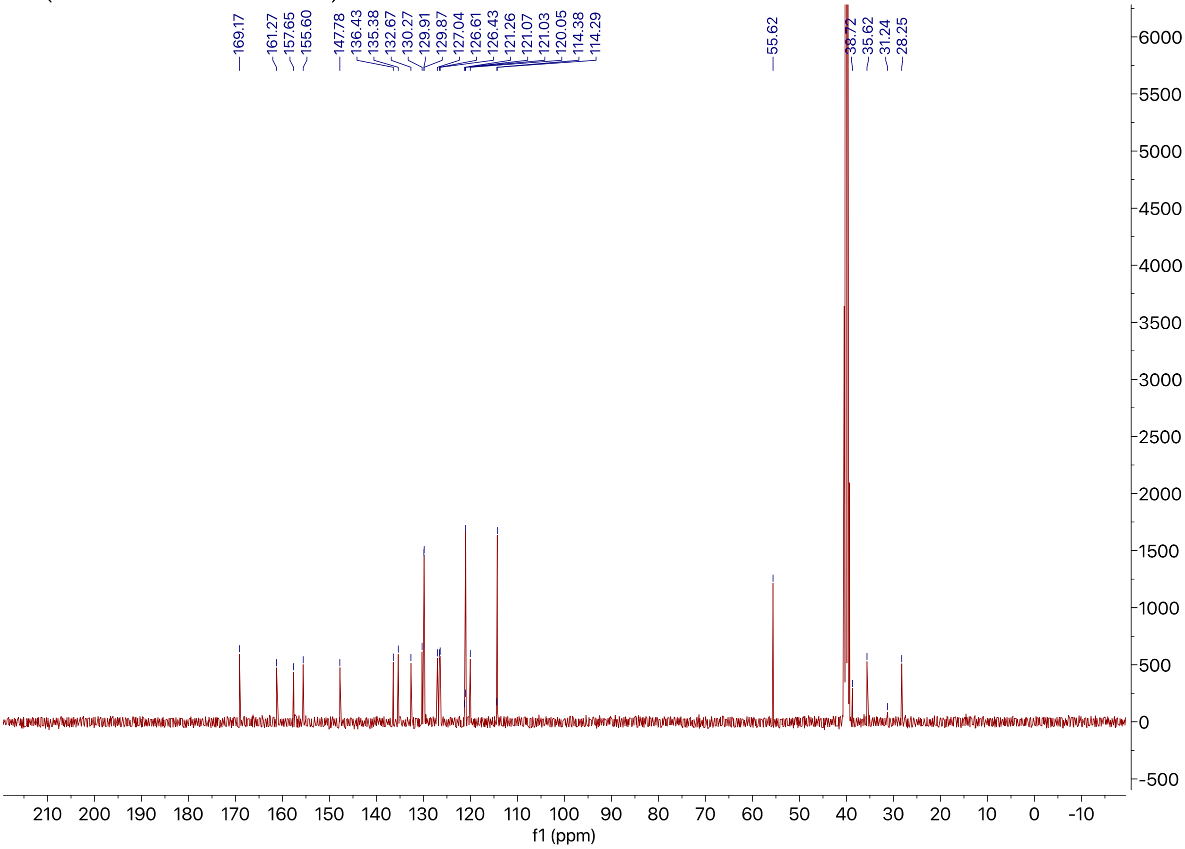

3-Cyclopropyl-2-((2-morpholino-2-oxoethyl)thio)quinazolin-4(3*H*)-one (**5g**)

^{1}H NMR (400 MHz, DMSO-d_6):

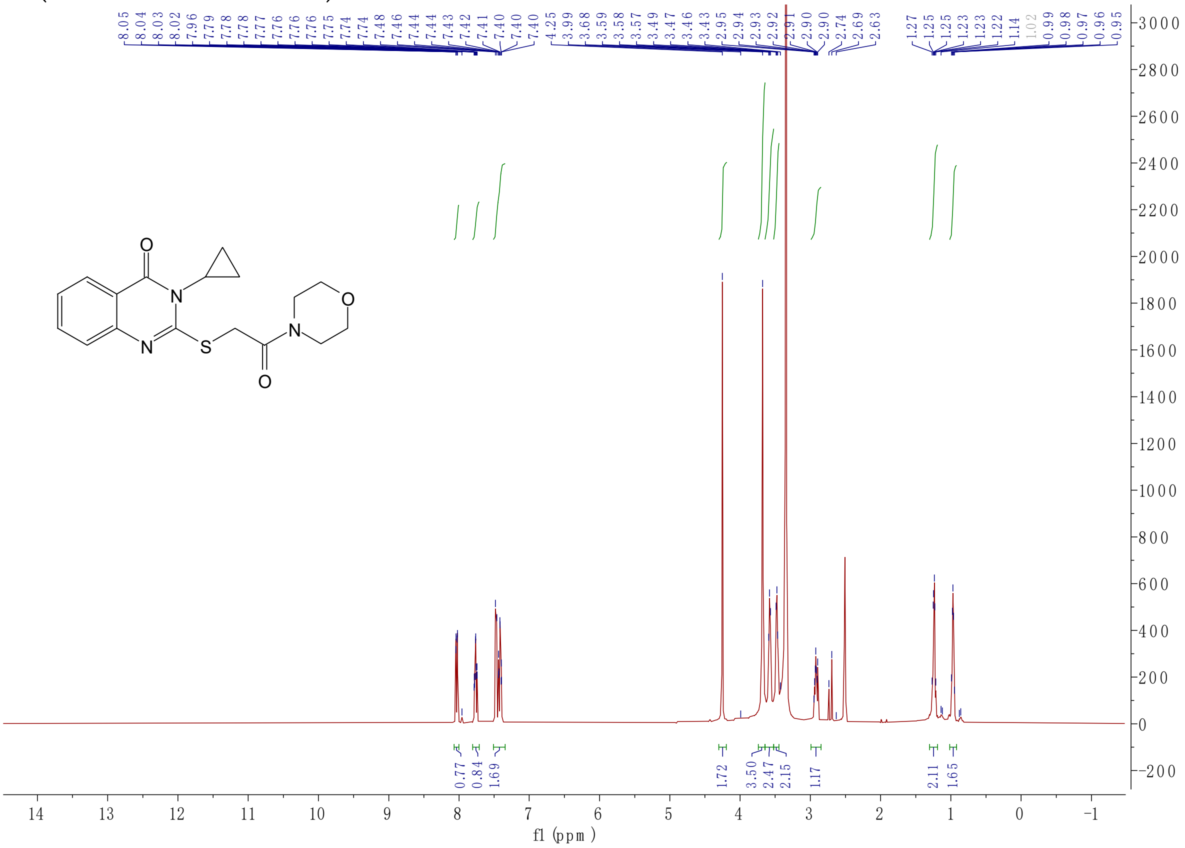

^{13}C NMR (100 MHz, DMSO-d_6):

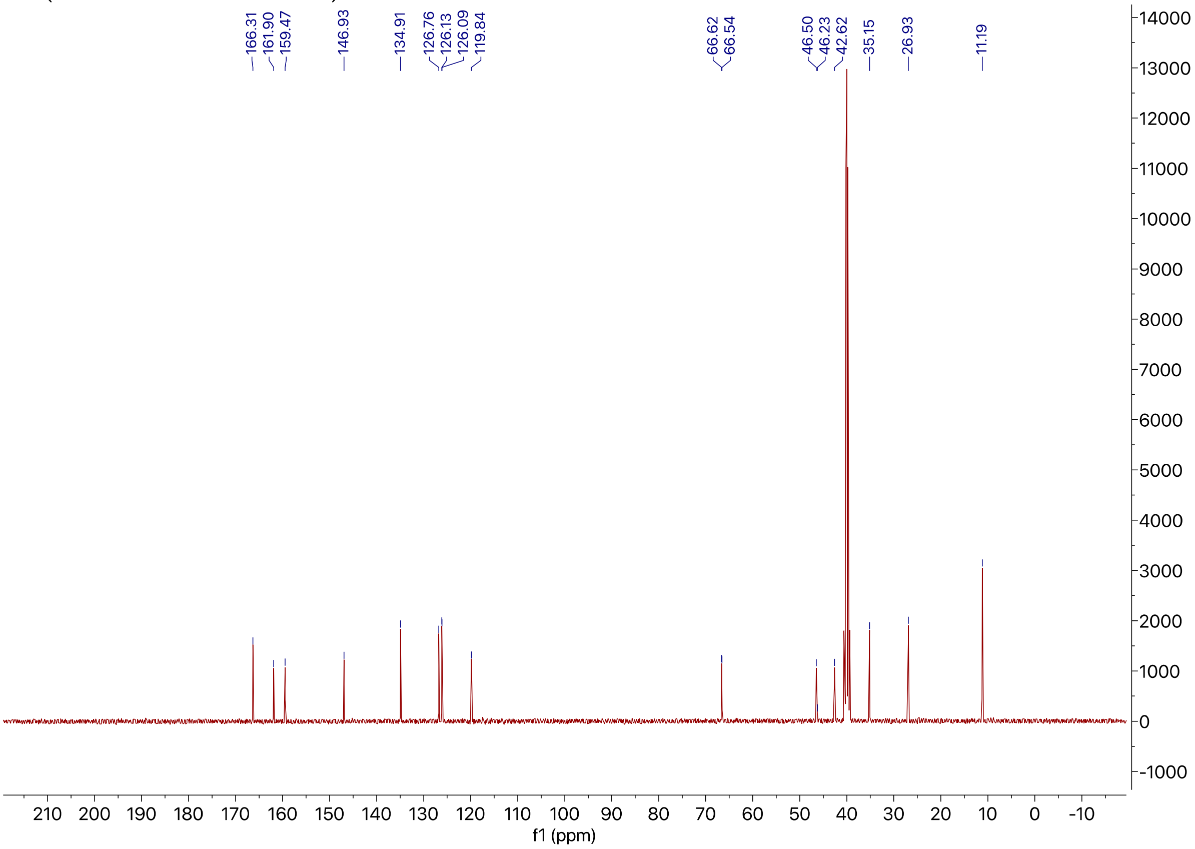

2-((2-Morpholino-2-oxoethyl)thio)-3-phenylquinazolin-4(3*H*)-one (**5h**)

^{1}H NMR (400 MHz, DMSO-d_6):

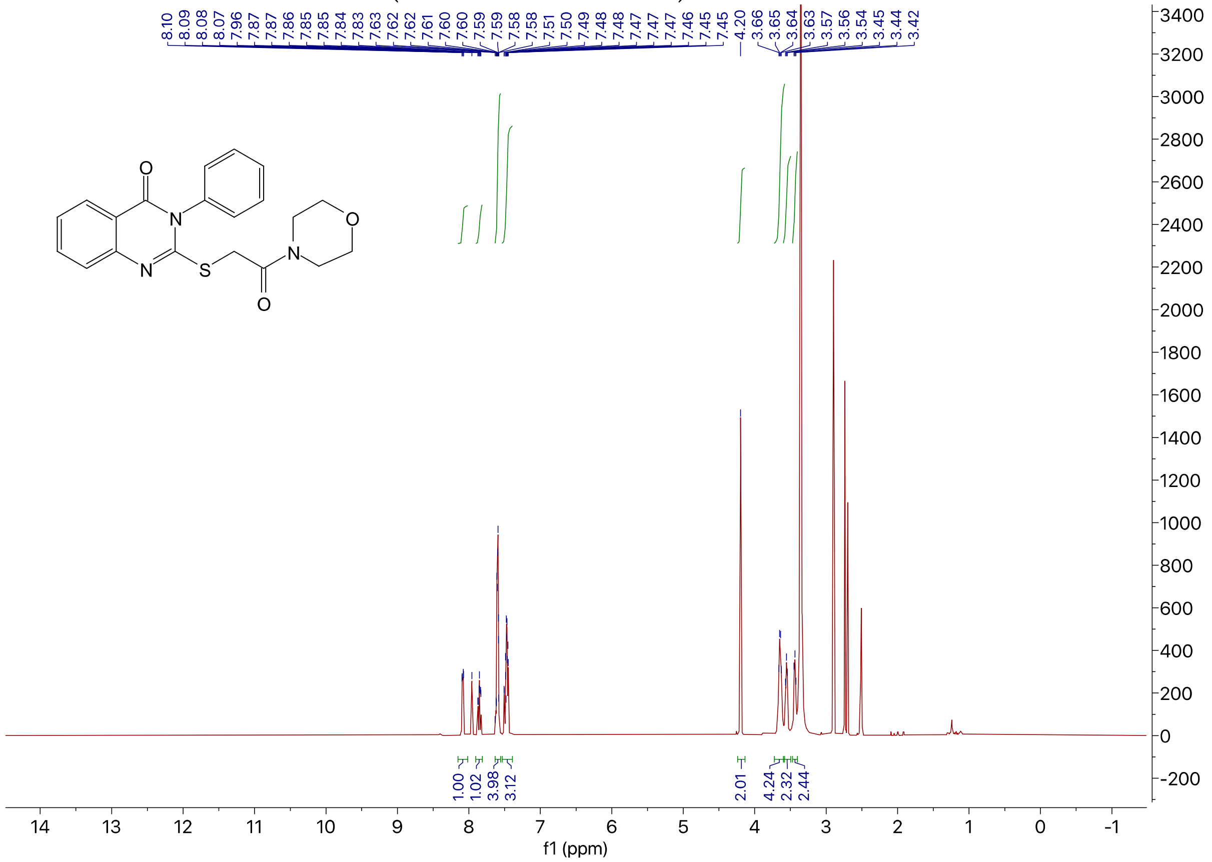

^{13}C NMR (100 MHz, DMSO-d_6):

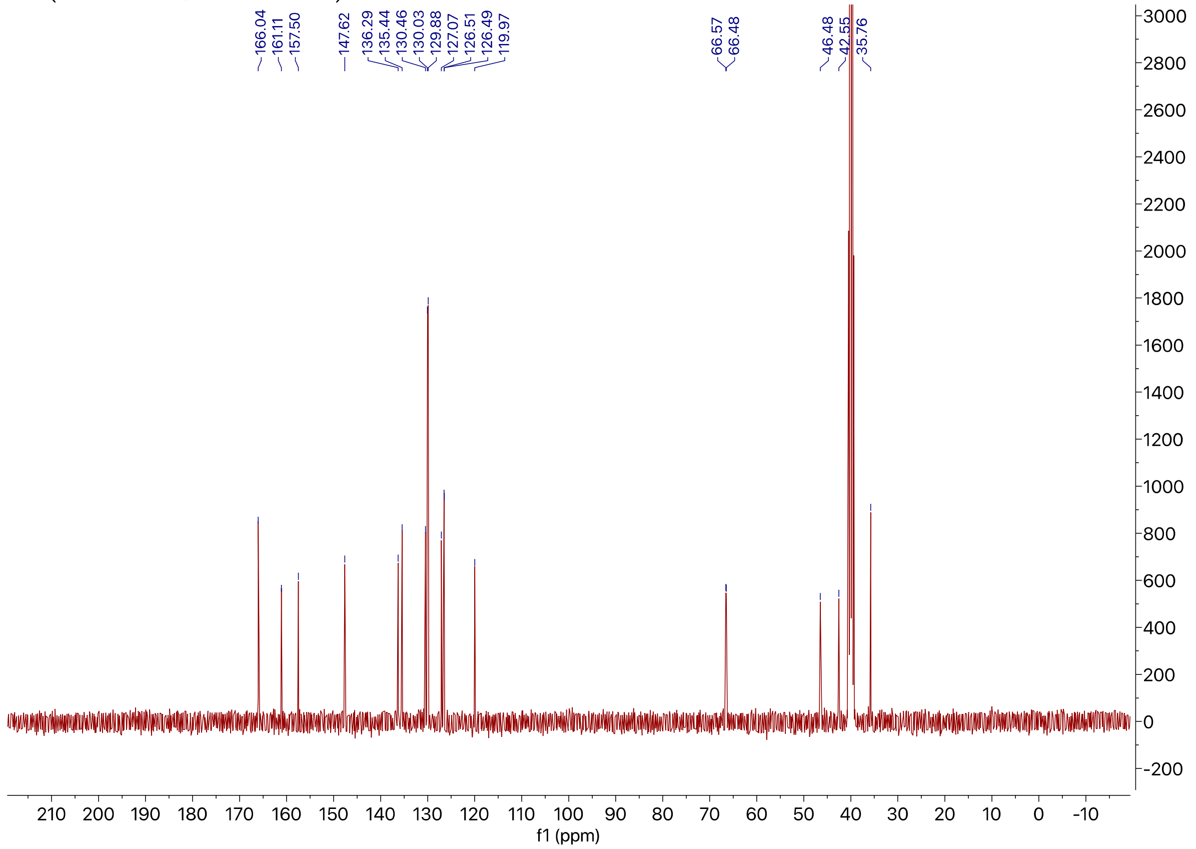

2-((2-Morpholino-2-oxoethyl)thio)quinazolin-4(3*H*)-one (**5i**)

^{1}H NMR (400 MHz, DMSO-d_6):

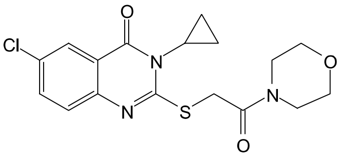

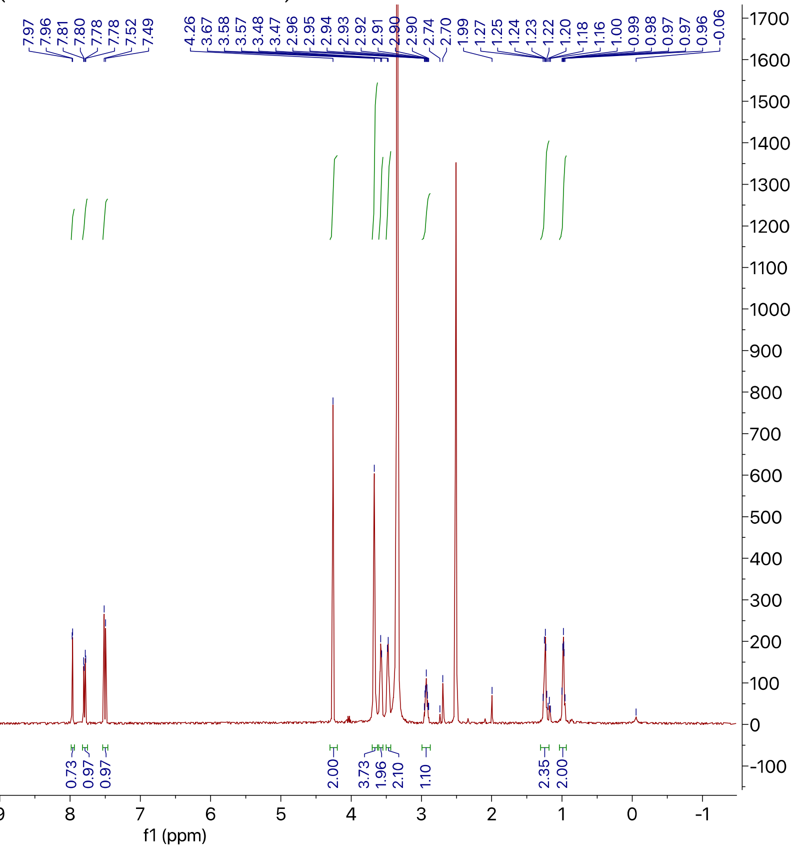

^{13}C NMR (100 MHz, DMSO-d_6):

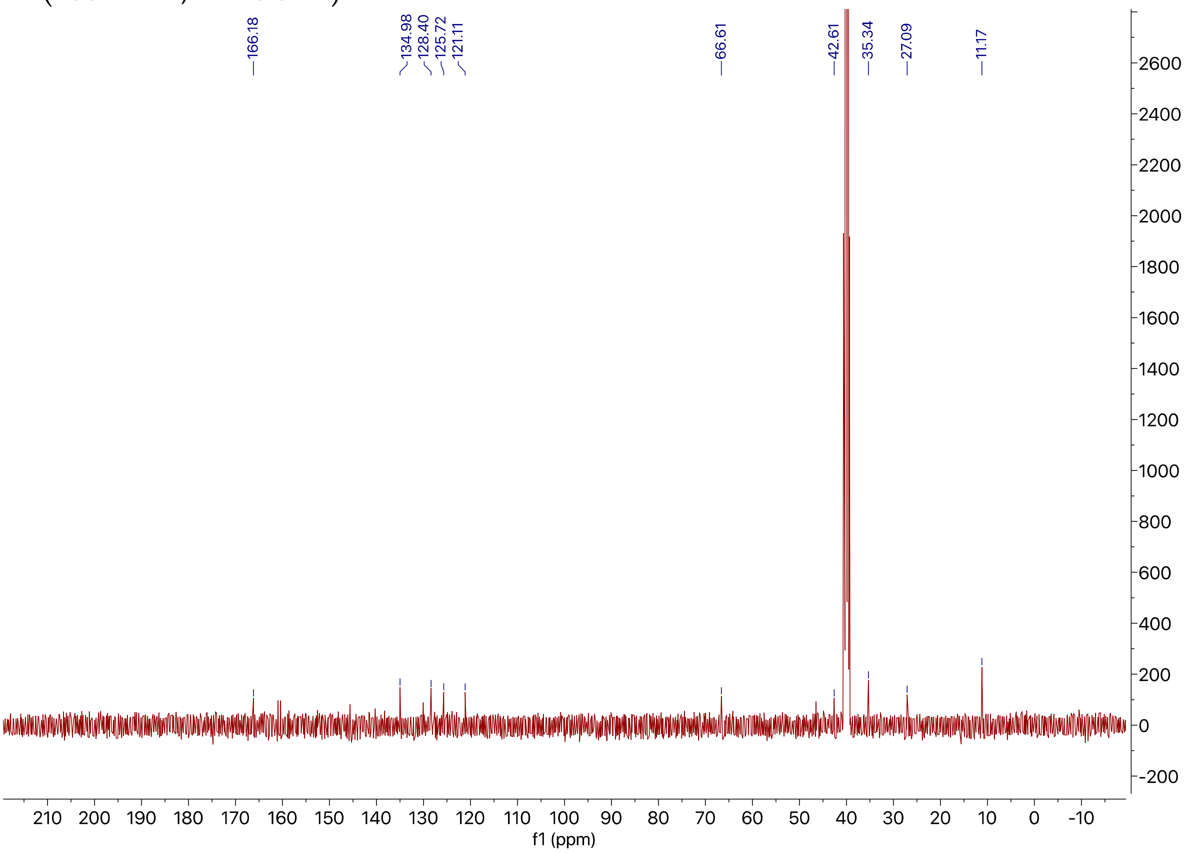

N-(4-Methoxyphenyl)-2-((4-oxo-3-phenyl-3,4-dihydroquinazolin-2-yl)thio)acetamide (**5j**)

^{1}H NMR (400 MHz, DMSO-d_6):

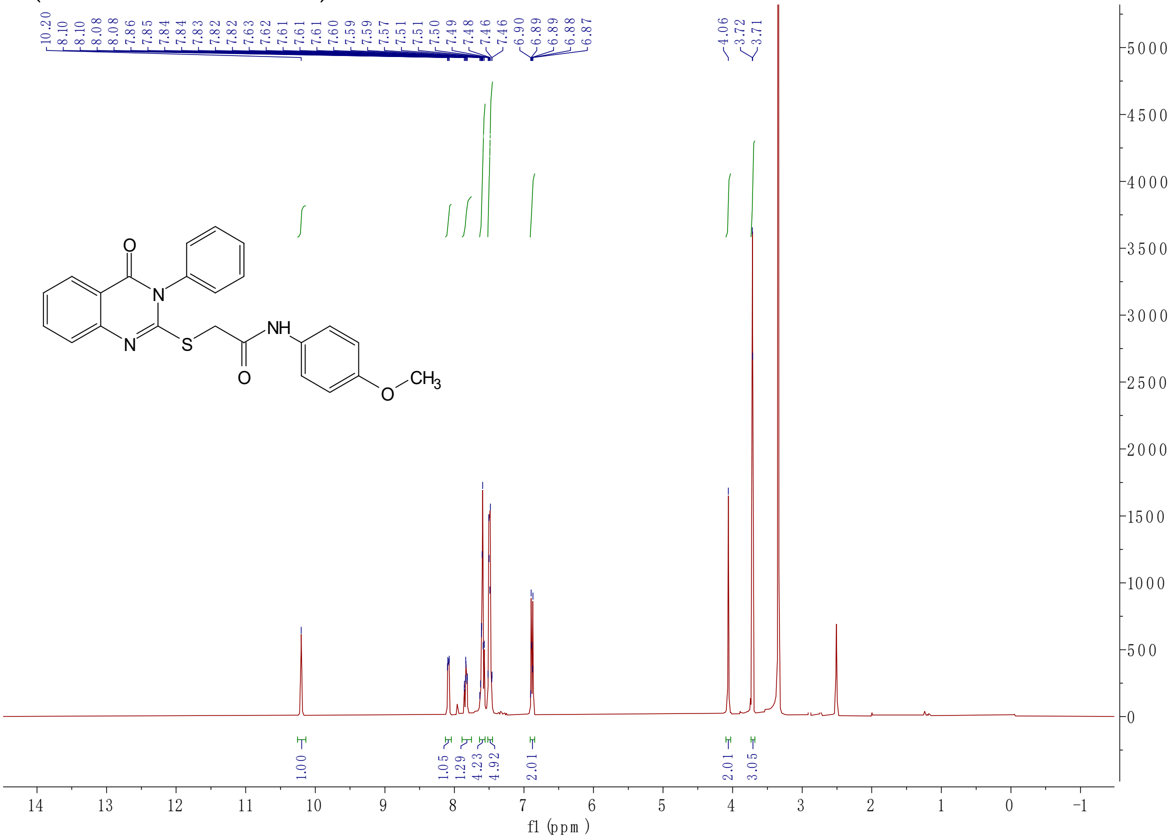

^{13}C NMR (100 MHz, DMSO-d_6):

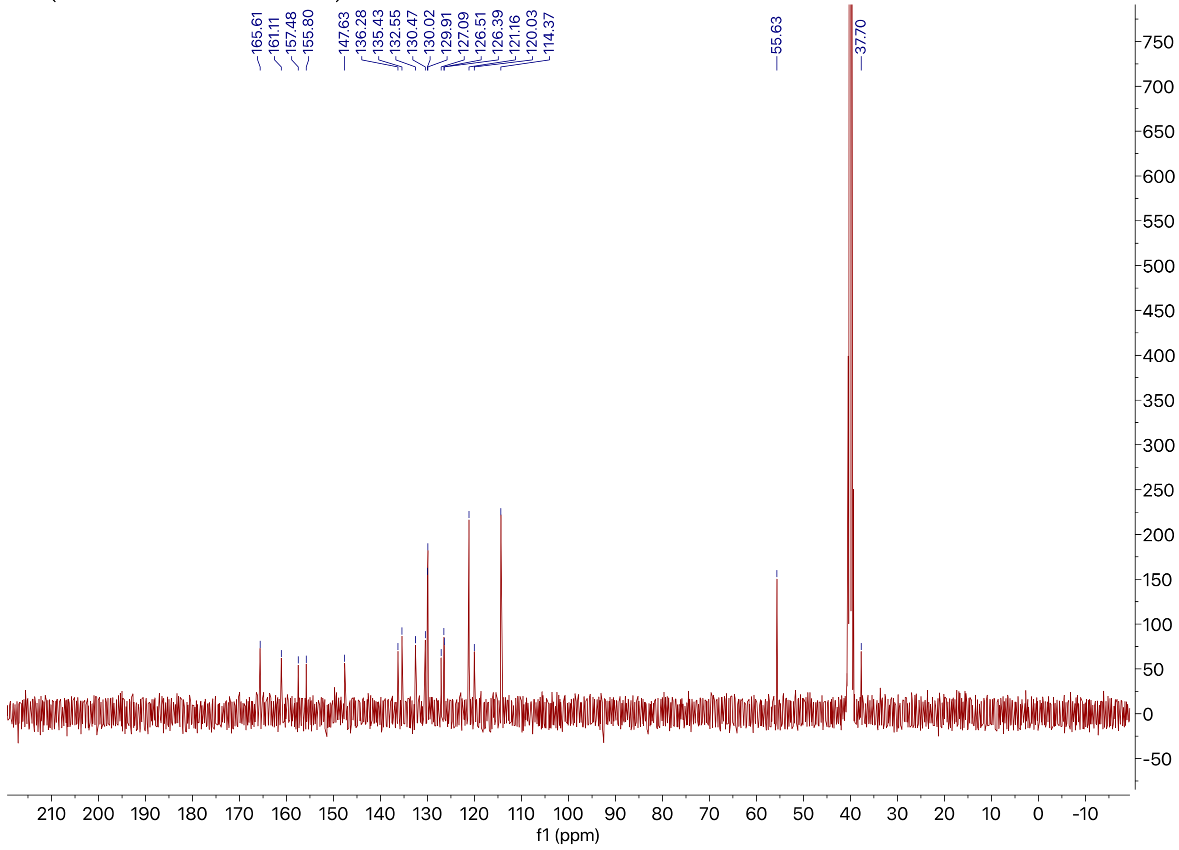

2-((6-Chloro-3-cyclopropyl-4-oxo-3,4-dihydroquinazolin-2-yl)thio)-*N*-(4-methoxyphenyl)acetamide (**5k**)

^{1}H NMR (400 MHz, $CDCl_3$):

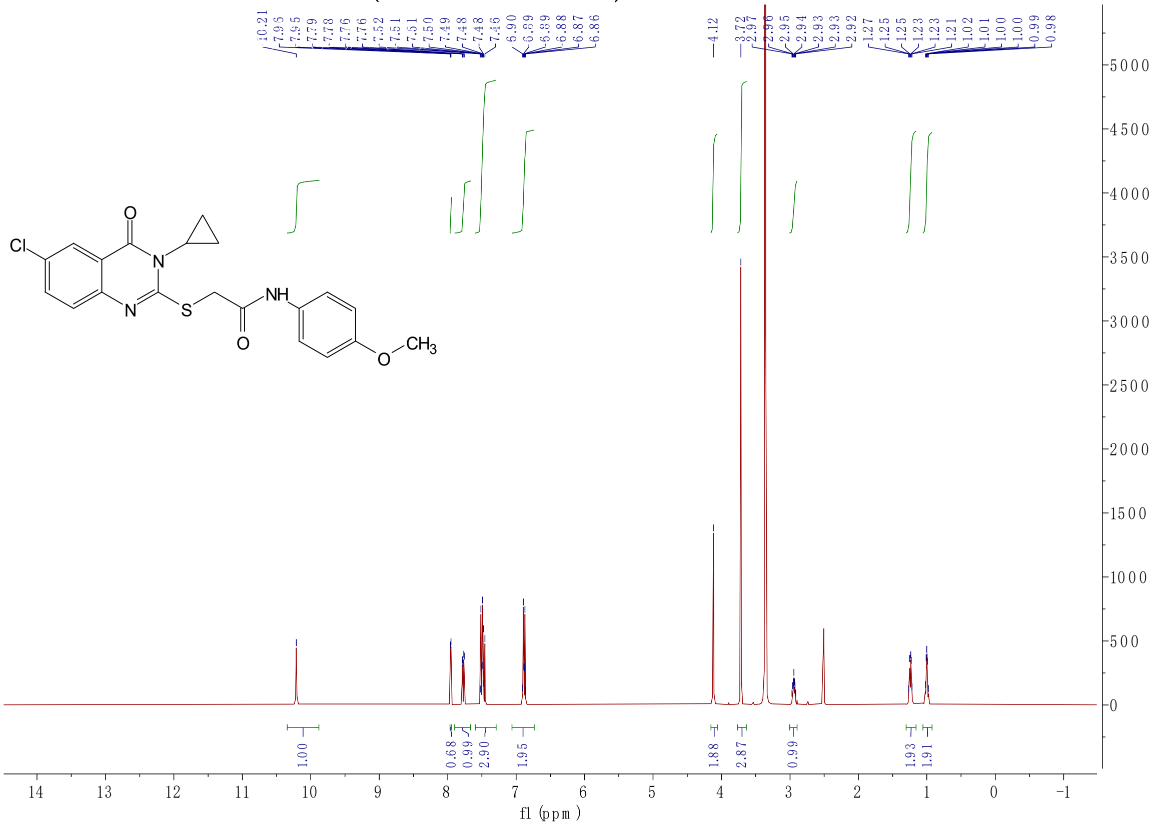

^{13}C NMR (100 MHz, $CDCl_3$):

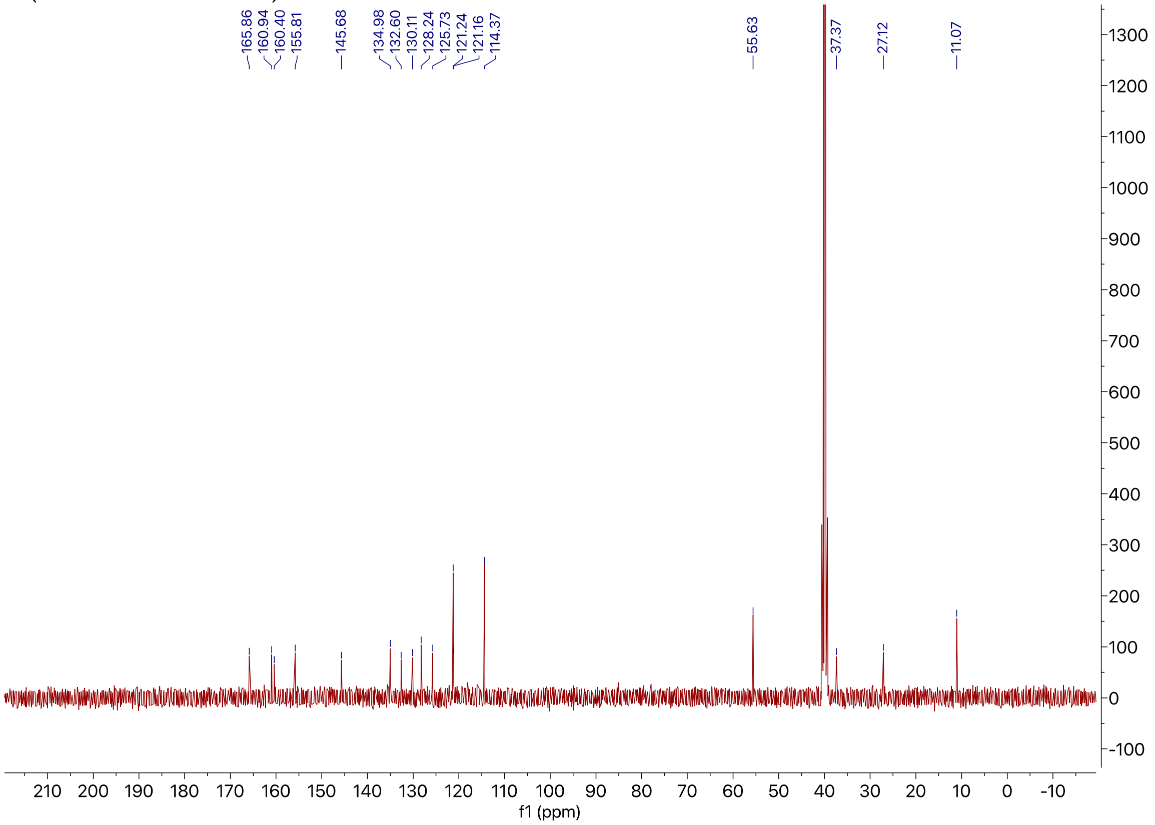

2-(4-(((3-Cyclopropyl-4-oxo-3,4-dihydroquinazolin-2-yl)thio)methyl)-1*H*-1,2,3-triazol-1-yl)-*N*-phenylacetamide (**6a**)

^{1}H NMR (400 MHz, DMSO-d_6):

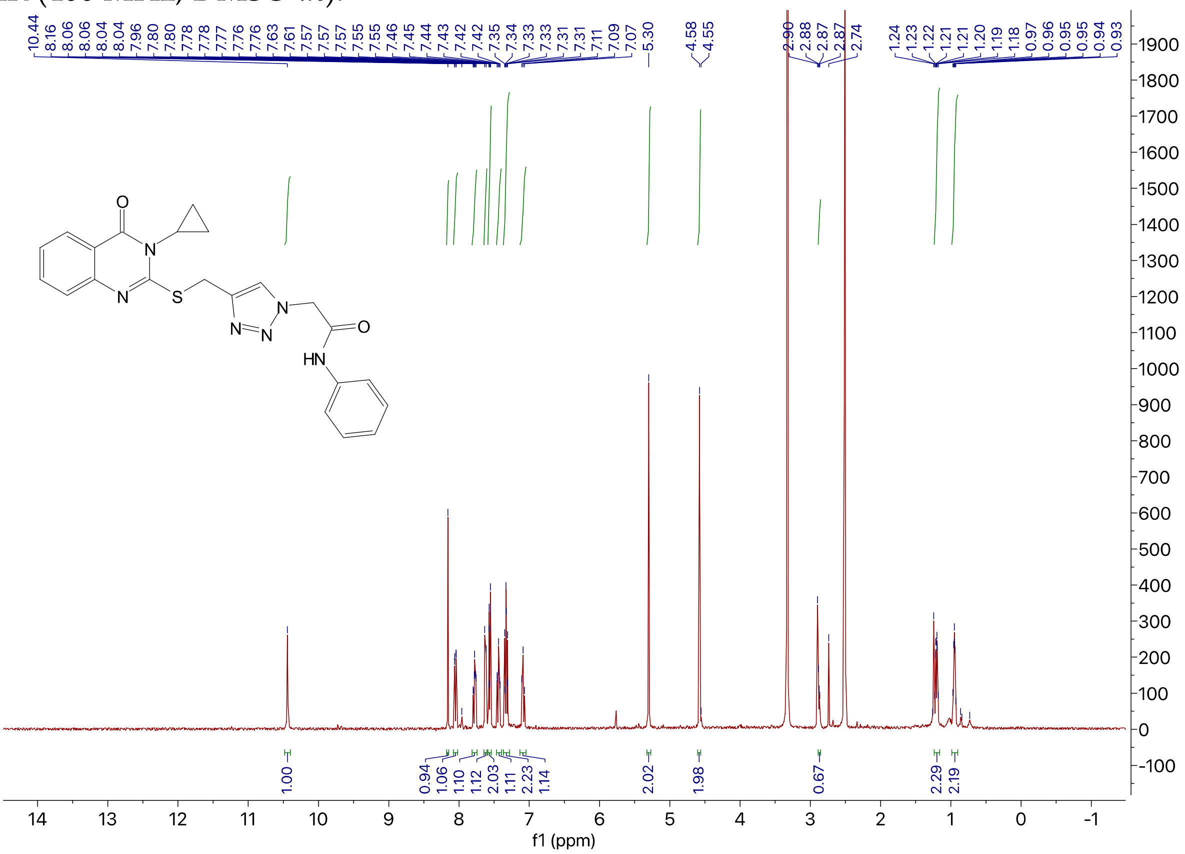

^{13}C NMR (100 MHz, DMSO-d_6):

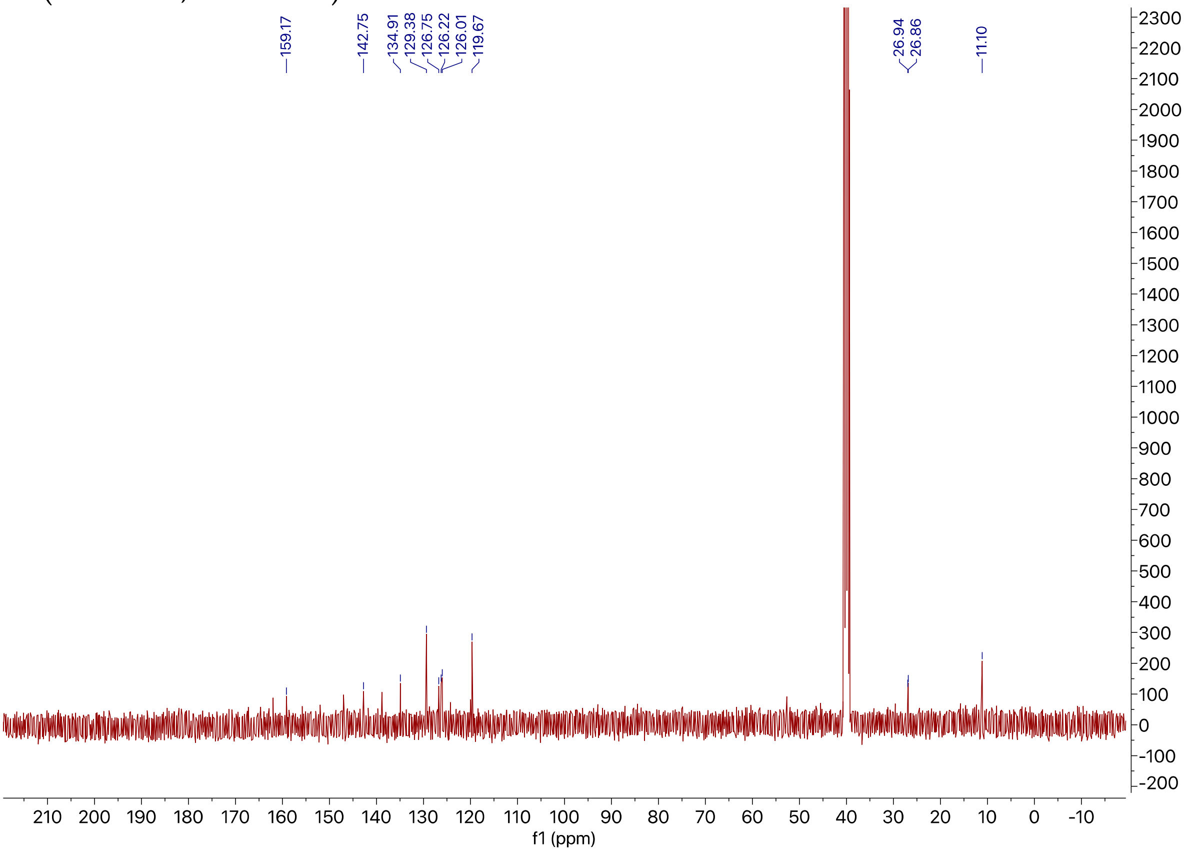

2-(4-(((3-Cyclopropyl-4-oxo-3,4-dihydroquinazolin-2-yl)thio)methyl)-1*H*-1,2,3-triazol-1-yl)-*N*-(4-methoxyphenyl)acetamide (**6b**)

^{1}H NMR (400 MHz, DMSO-d_6):

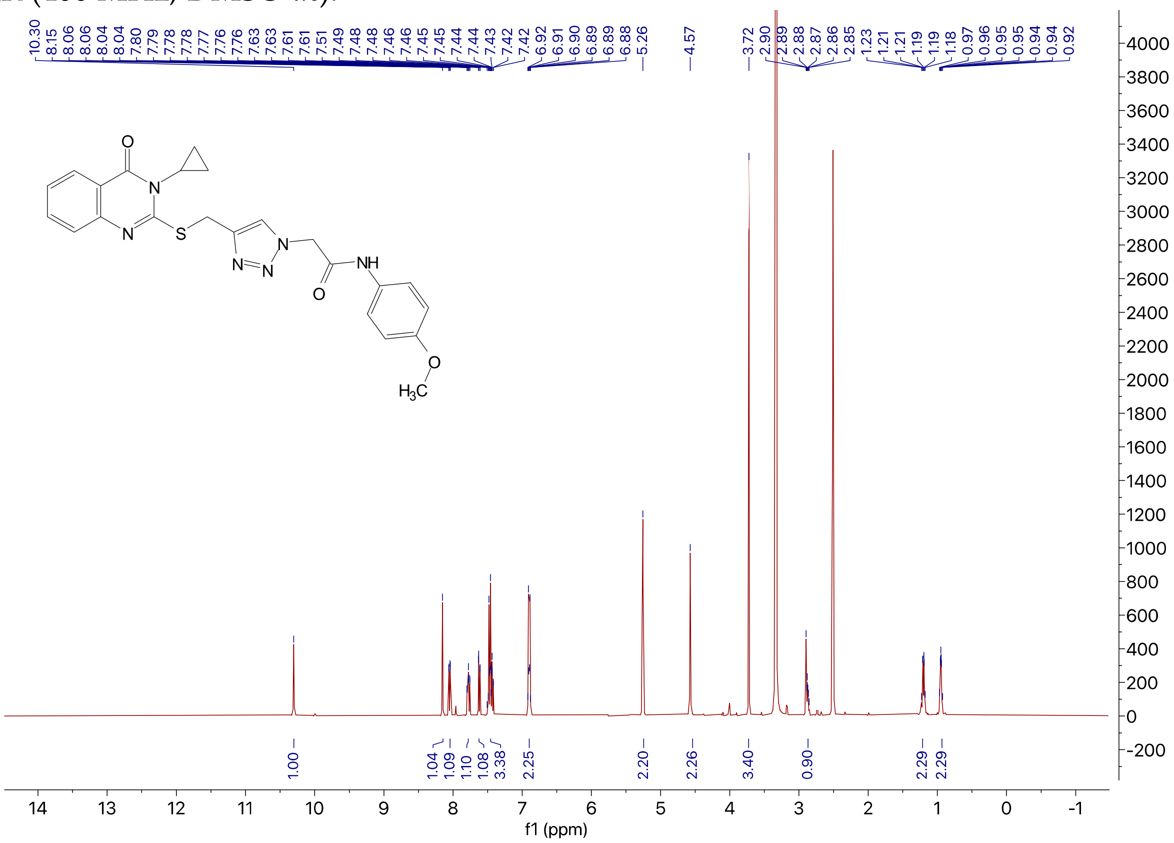

^{13}C NMR (100 MHz, DMSO-d_6):

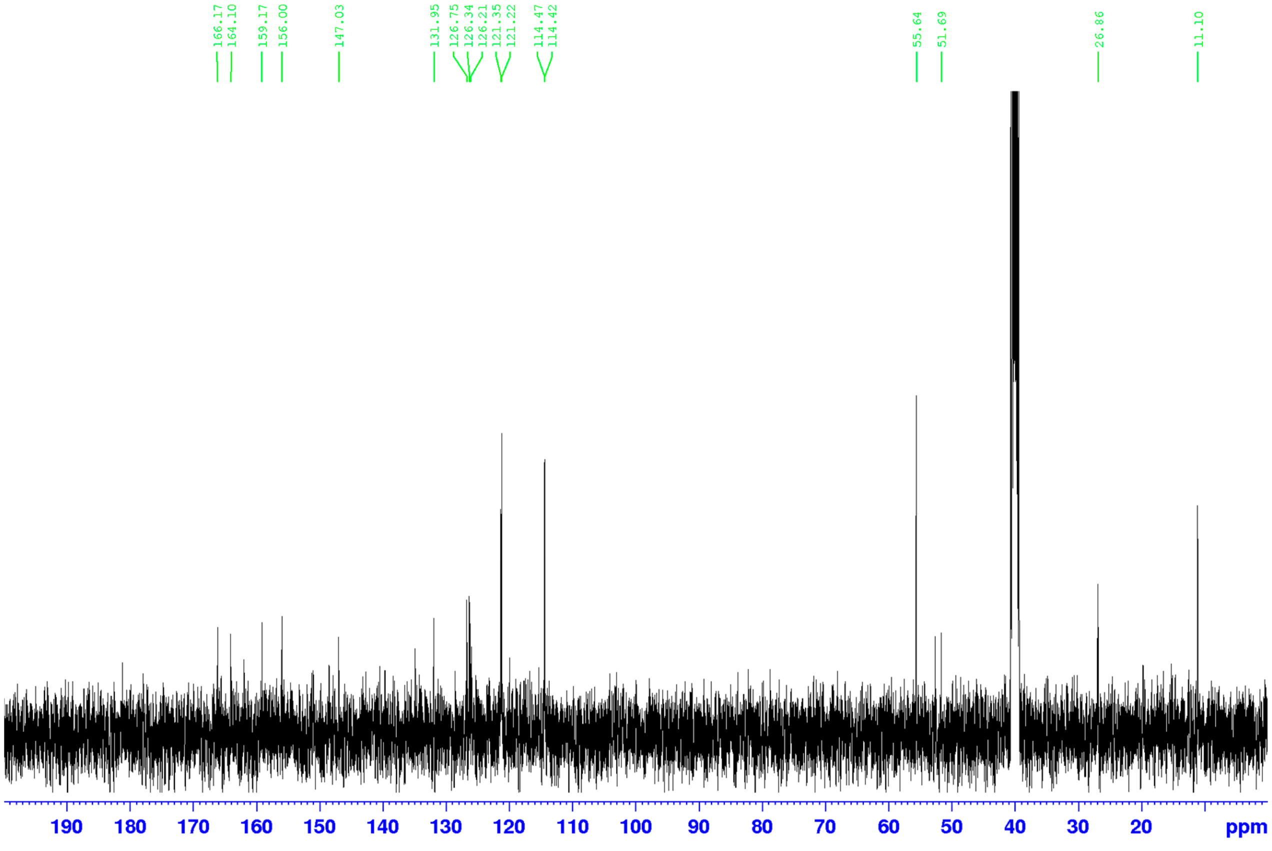

2-(4-(((6-Chloro-3-cyclopropyl-4-oxo-3,4-dihydroquinazolin-2-yl)thio)methyl)-1*H*-1,2,3-triazol-1-yl)-*N*-(4-methoxyphenyl)acetamide (**6c**)

^{1}H NMR (400 MHz, DMSO-d_6):

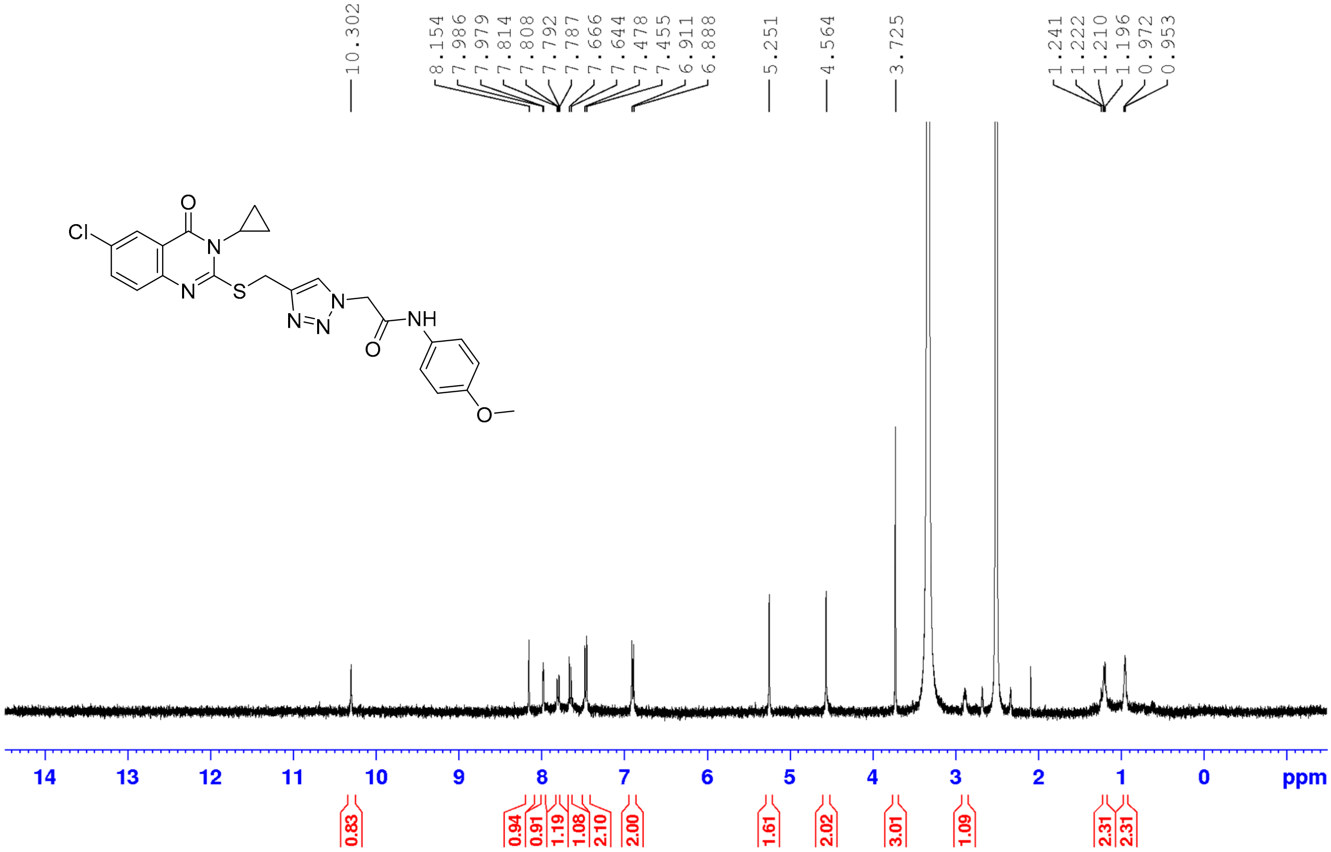

^{13}C NMR (100 MHz, DMSO-d_6):

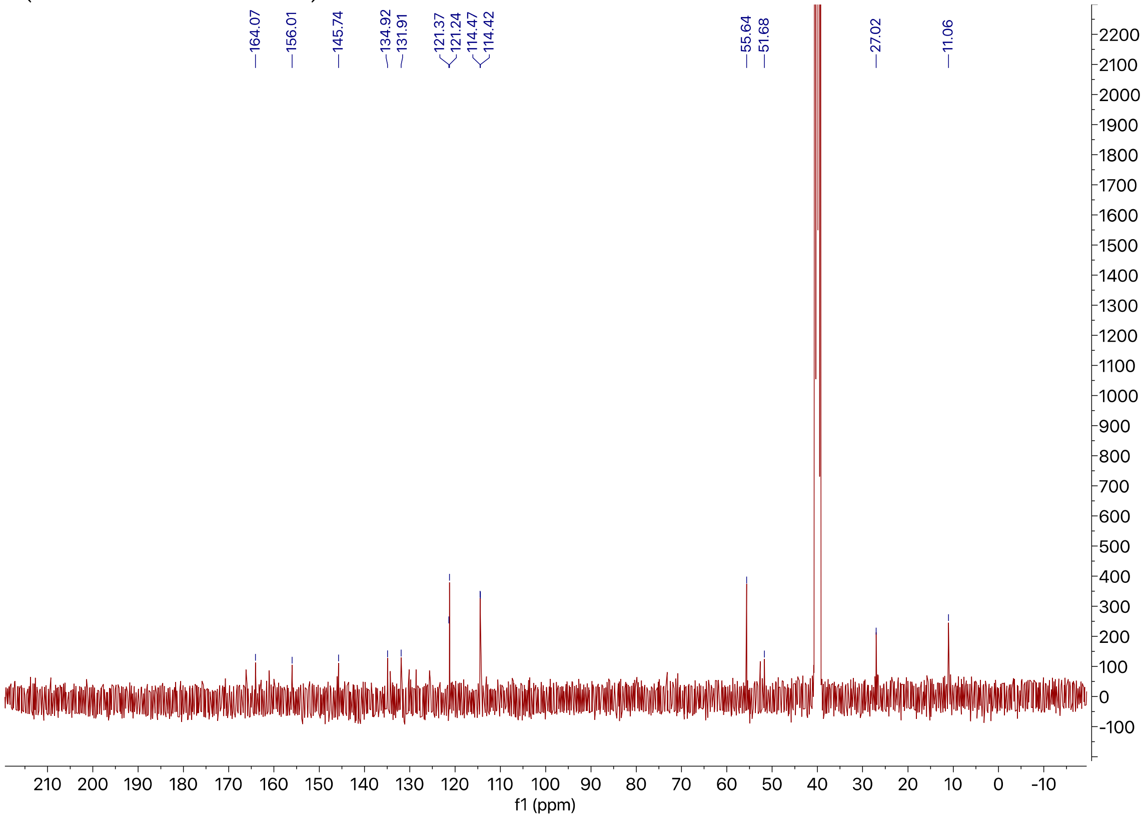

N-(4-Methoxyphenyl)-2-(4-(((4-oxo-3-phenyl-3,4-dihydroquinazolin-2-yl)thio)methyl)-1*H*-1,2,3-triazol-1-yl)acetamide (**6d**)

^{1}H NMR (400 MHz, DMSO-*d*$_6$):

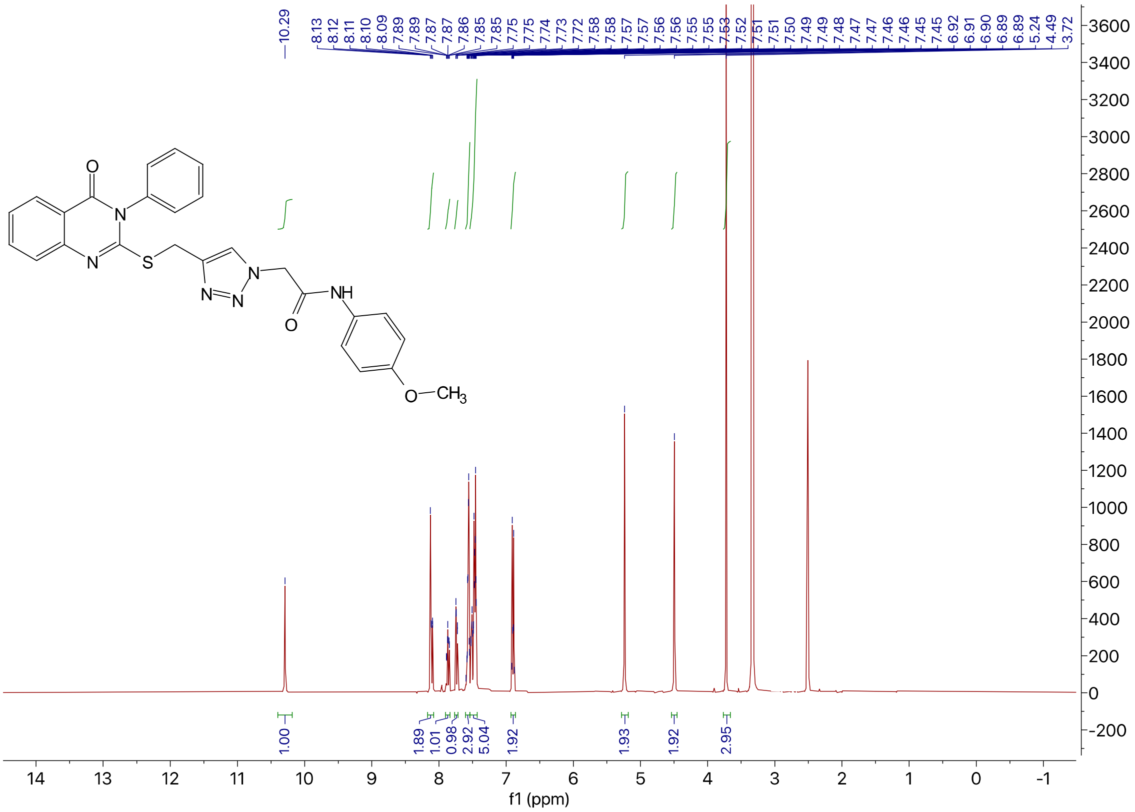

^{13}C NMR (100 MHz, DMSO-*d*$_6$):

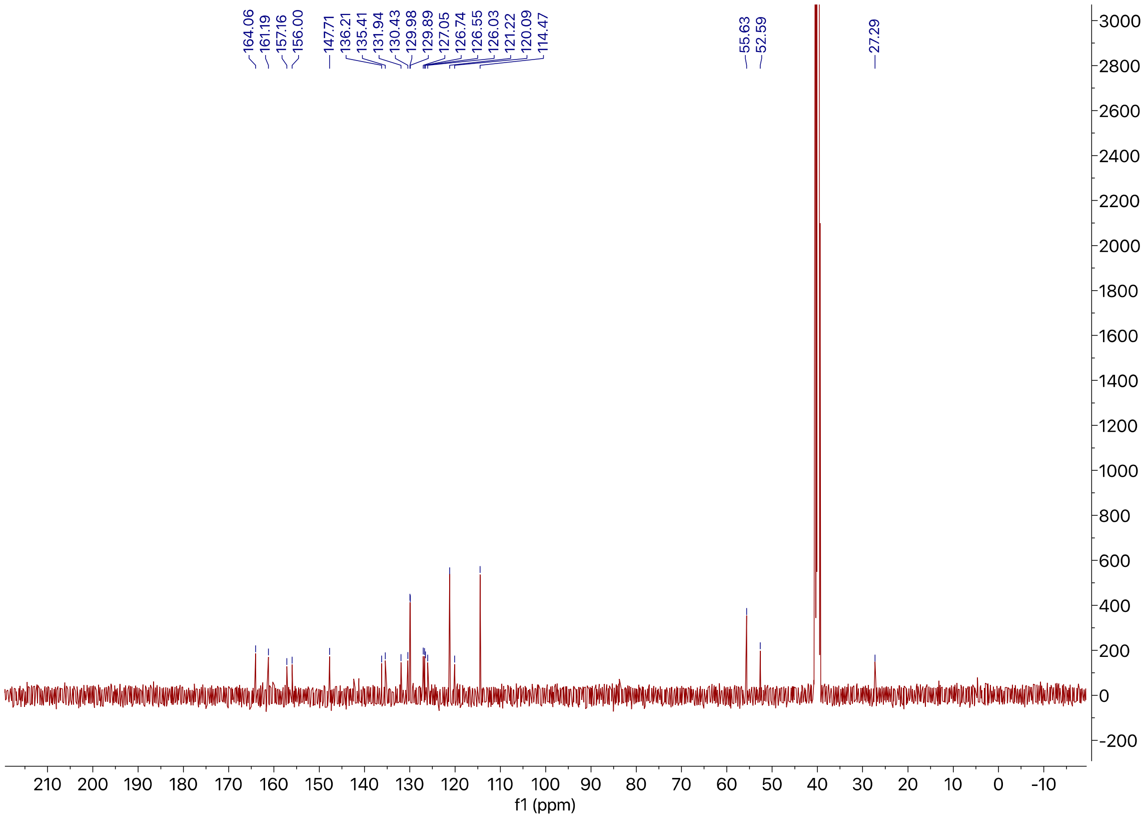

2-(4-(((3-Cyclopropyl-4-oxo-3,4-dihydroquinazolin-2-yl)thio)methyl)-1*H*-1,2,3-triazol-1-yl)-*N*-(4-nitrophenyl)acetamide (**6e**)

^{1}H NMR (400 MHz, DMSO-d_6):

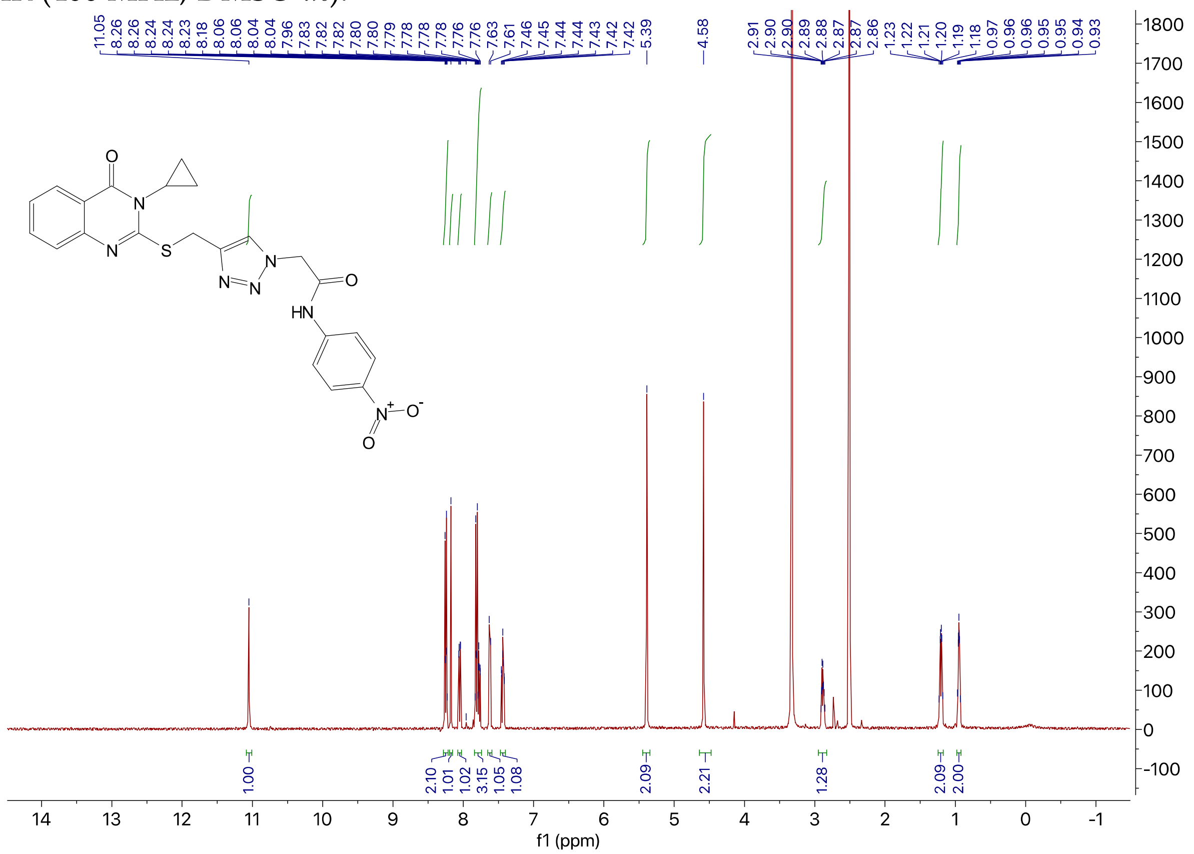

^{13}C NMR (100 MHz, DMSO-d_6):

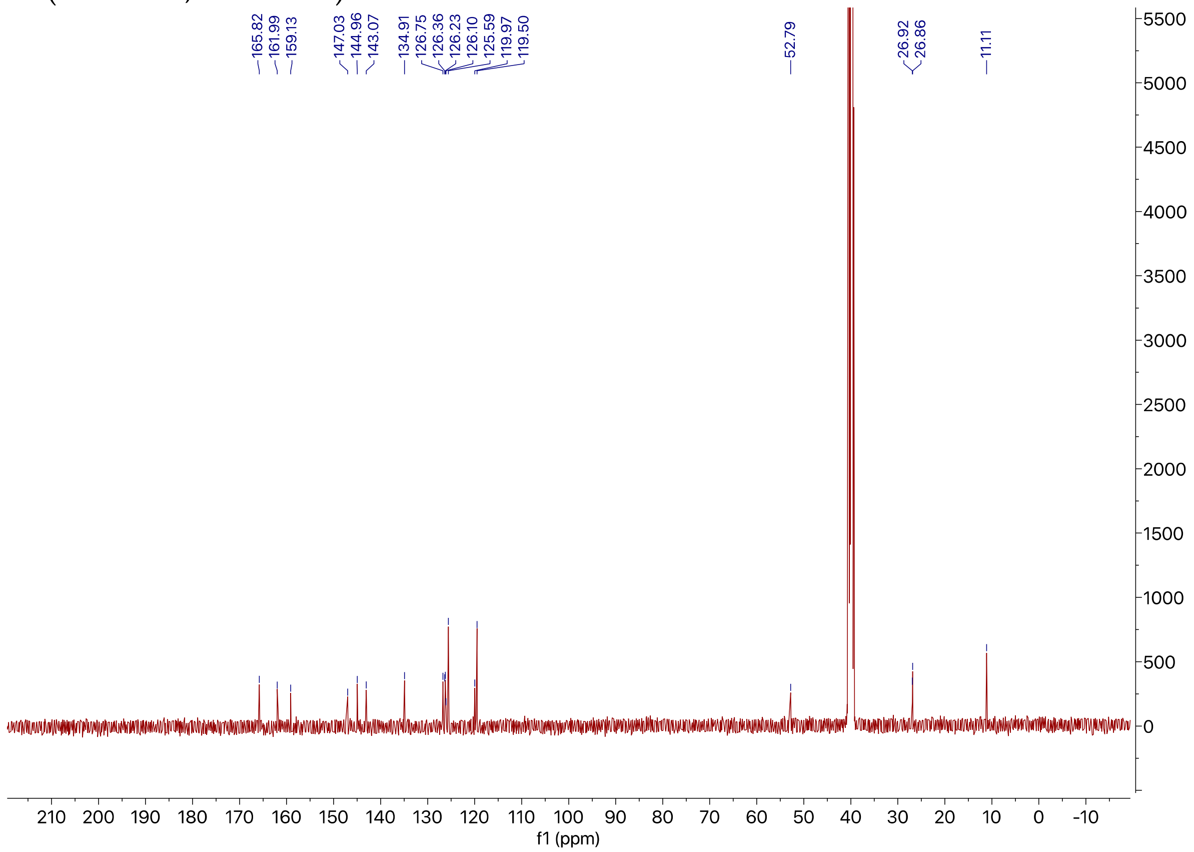

3-Cyclopropyl-2-mercaptoquinazolin-4(3*H*)-one (**8a**)

^{1}H NMR (400 MHz, DMSO-*d*6)

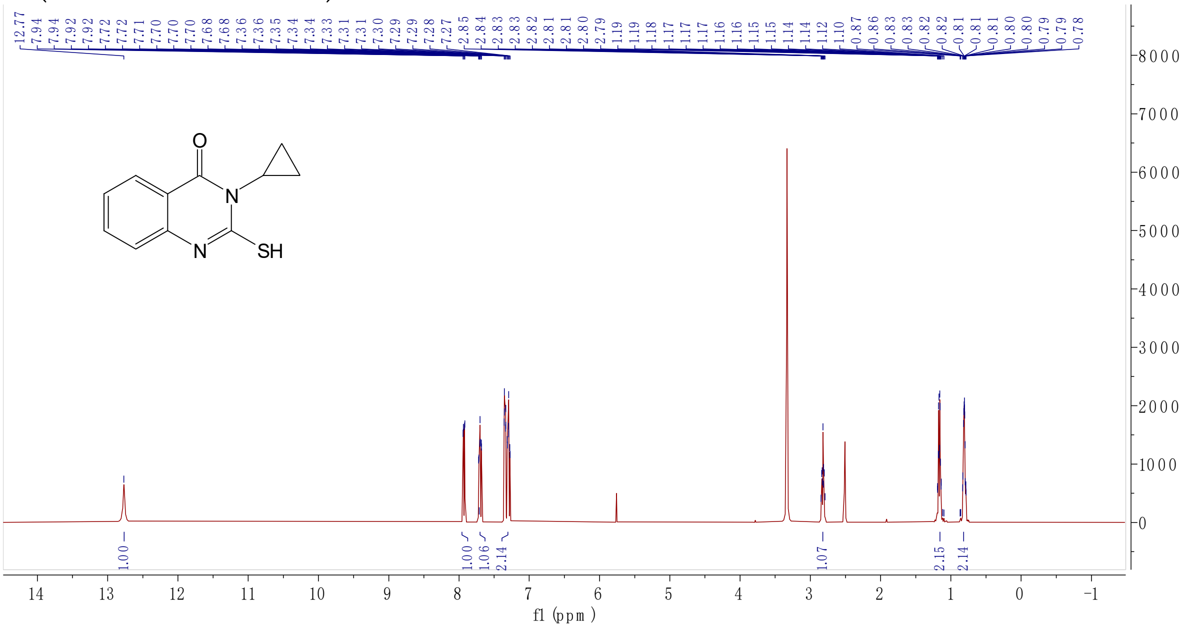

^{13}C NMR (100 MHz, DMSO-*d*6):

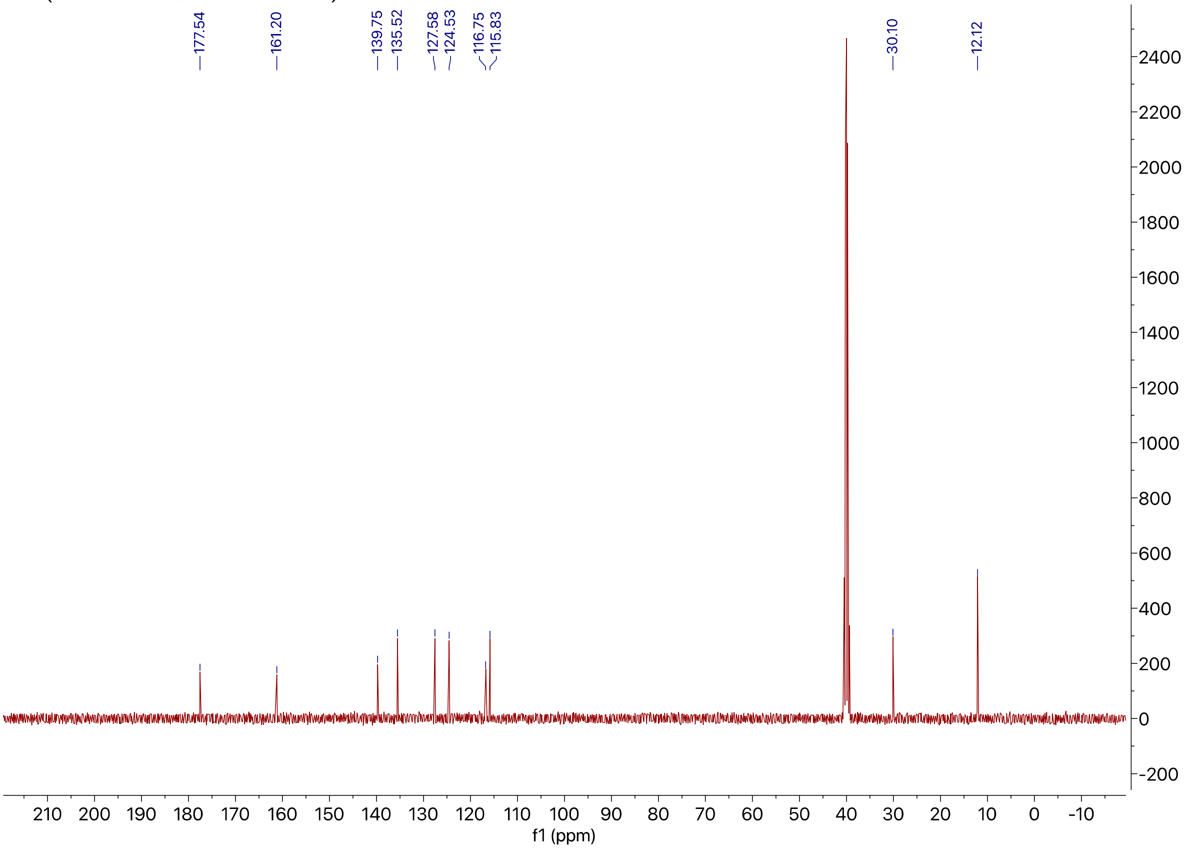

2-Mercapto-3-phenylquinazolin-4(3*H*)-one (**8b**)

^{1}H NMR (400 MHz, DMSO-d_6)

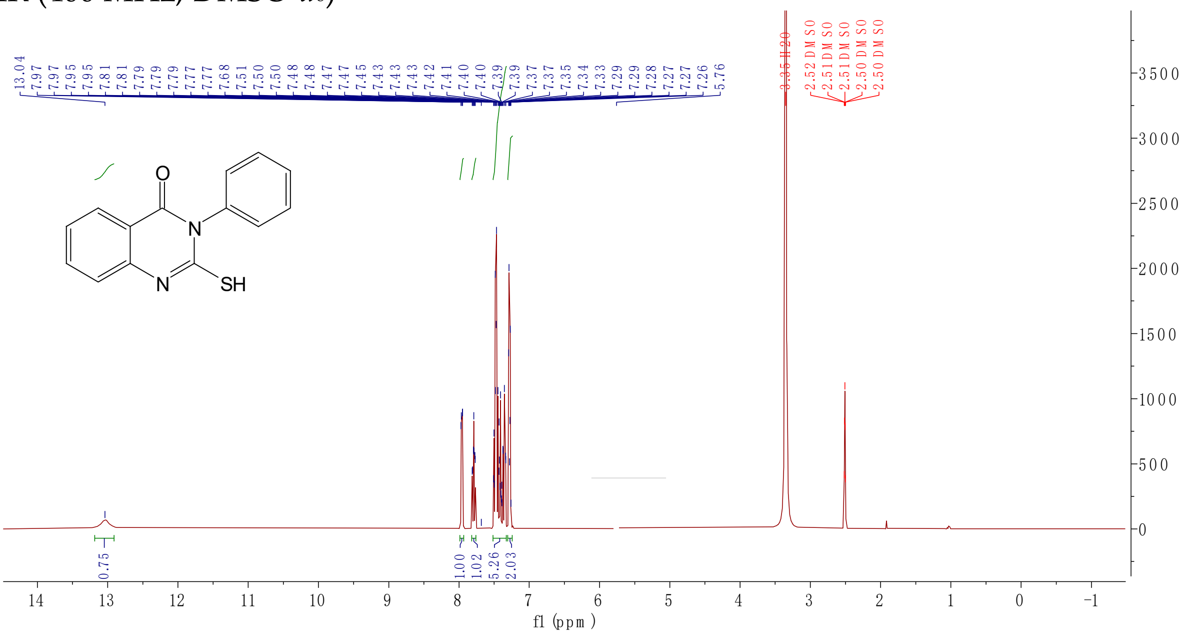

^{13}C NMR (100 MHz, DMSO-d_6):

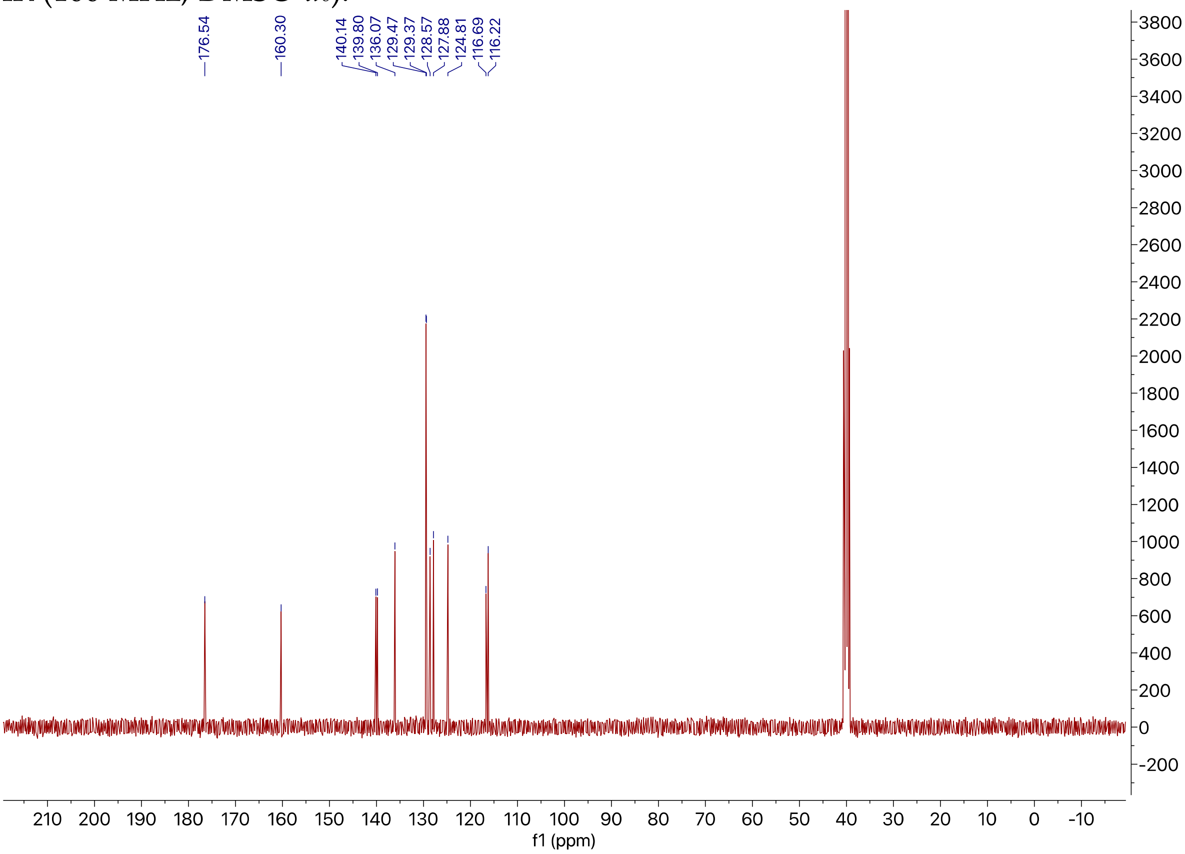

6-Chloro-3-cyclopropyl-2-mercaptoquinazolin-4(3*H*)-one (**8c**)

^{1}H NMR (400 MHz, DMSO-d_6)

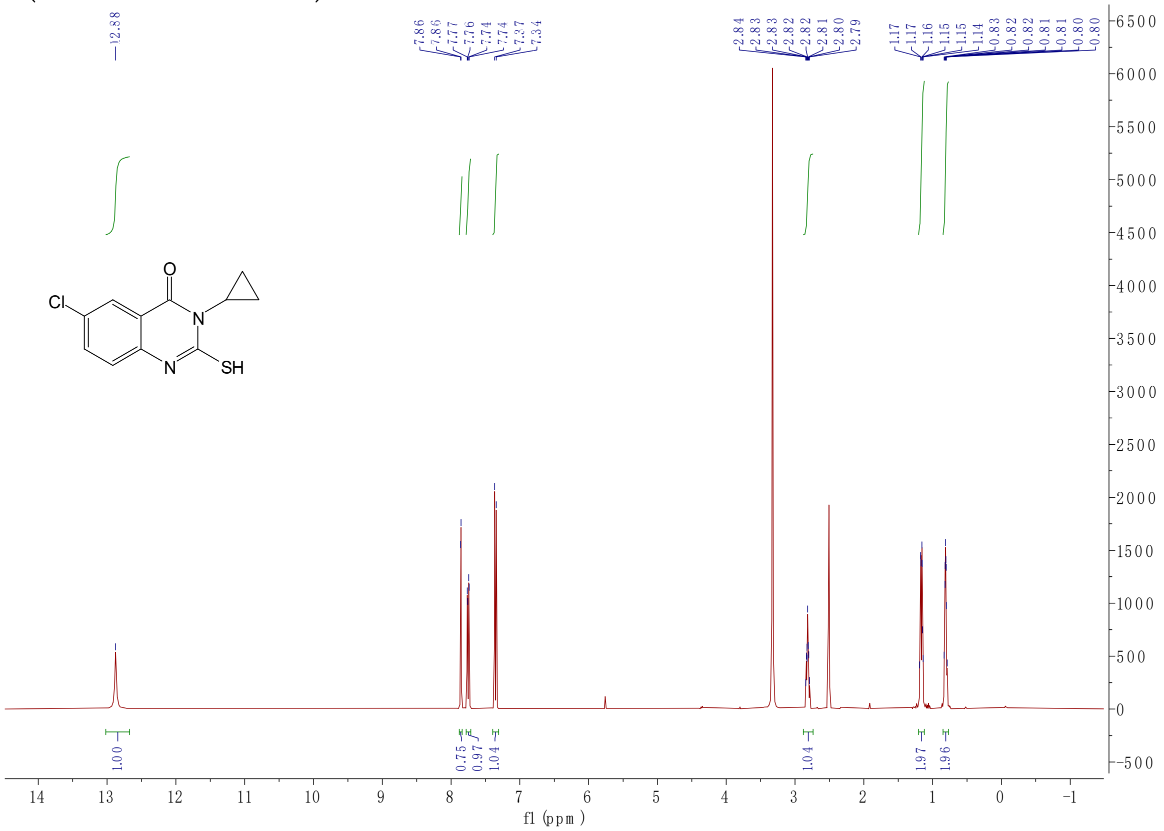

^{13}C NMR (100 MHz, DMSO-d_6):

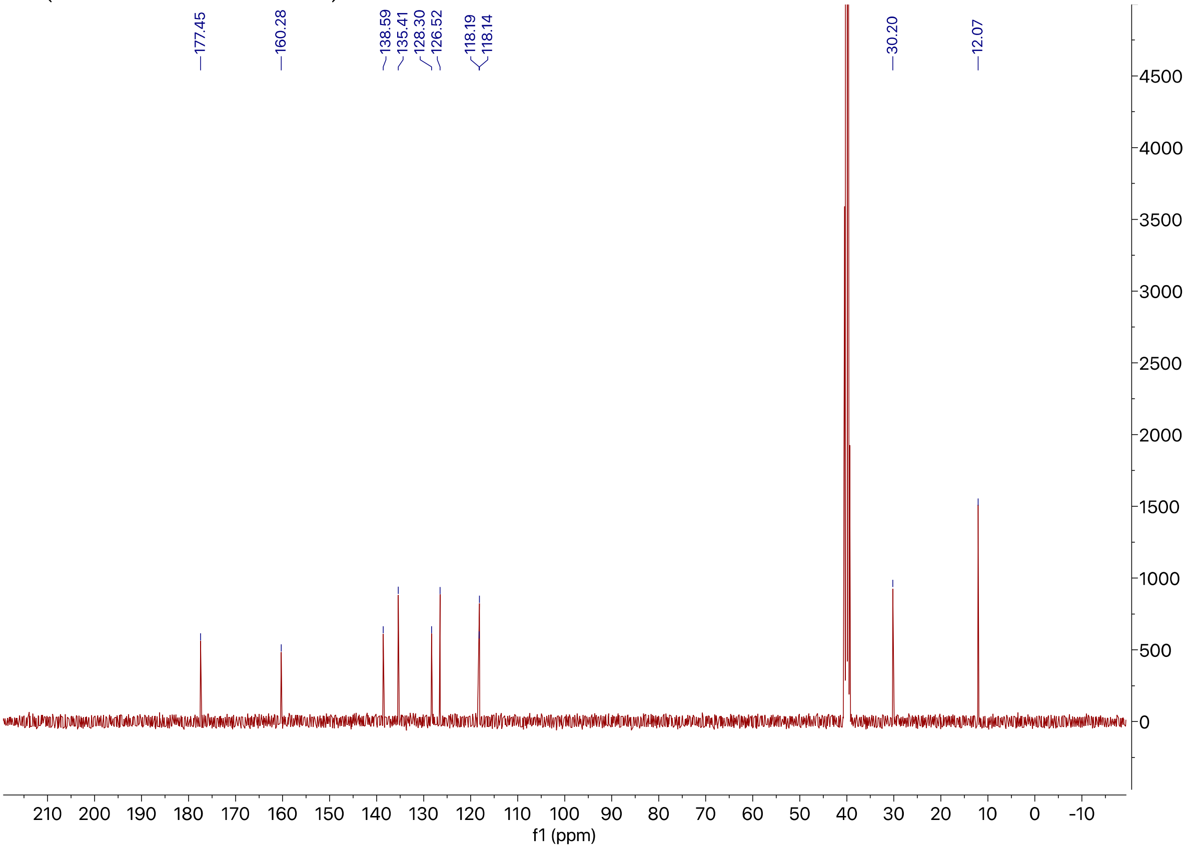

2-((3-Cyclopropyl-4-oxo-3,4-dihydroquinazolin-2-yl)thio)acetic acid (**9a**)

^{1}H NMR (400 MHz, DMSO-d_6):

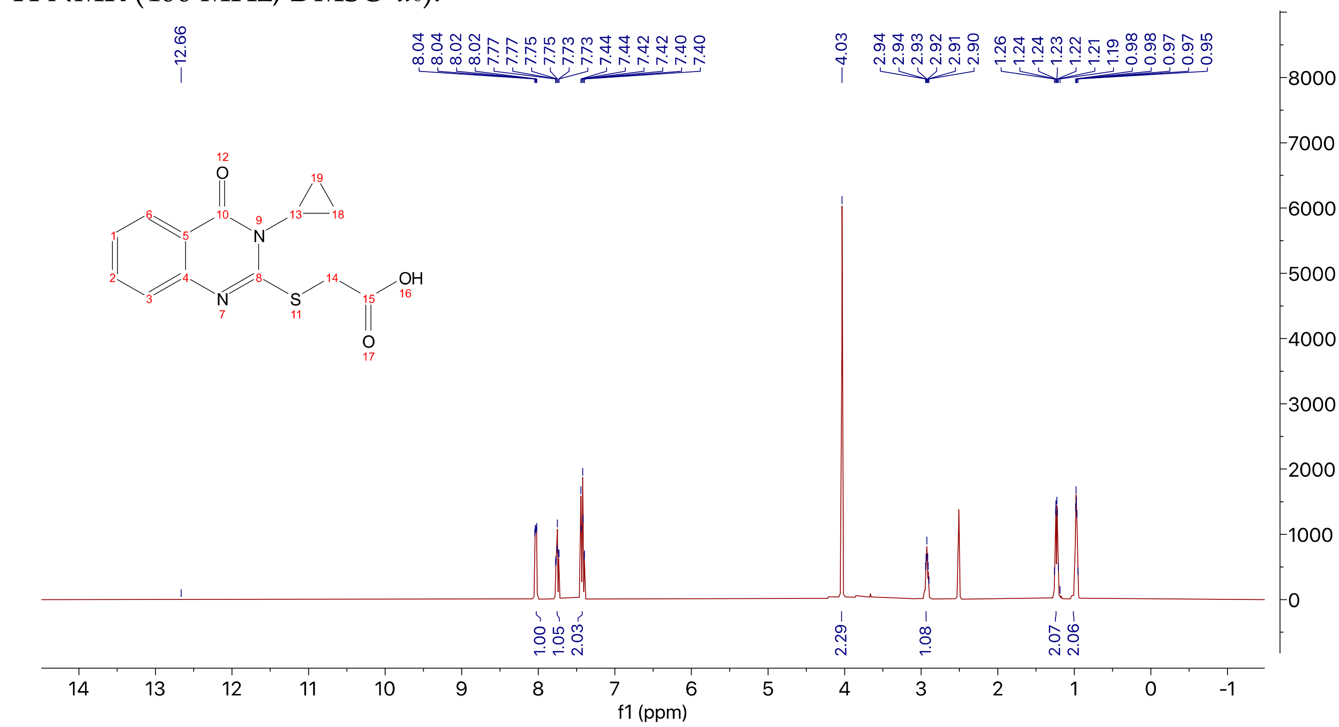

^{13}C NMR (100 MHz, DMSO-d_6):

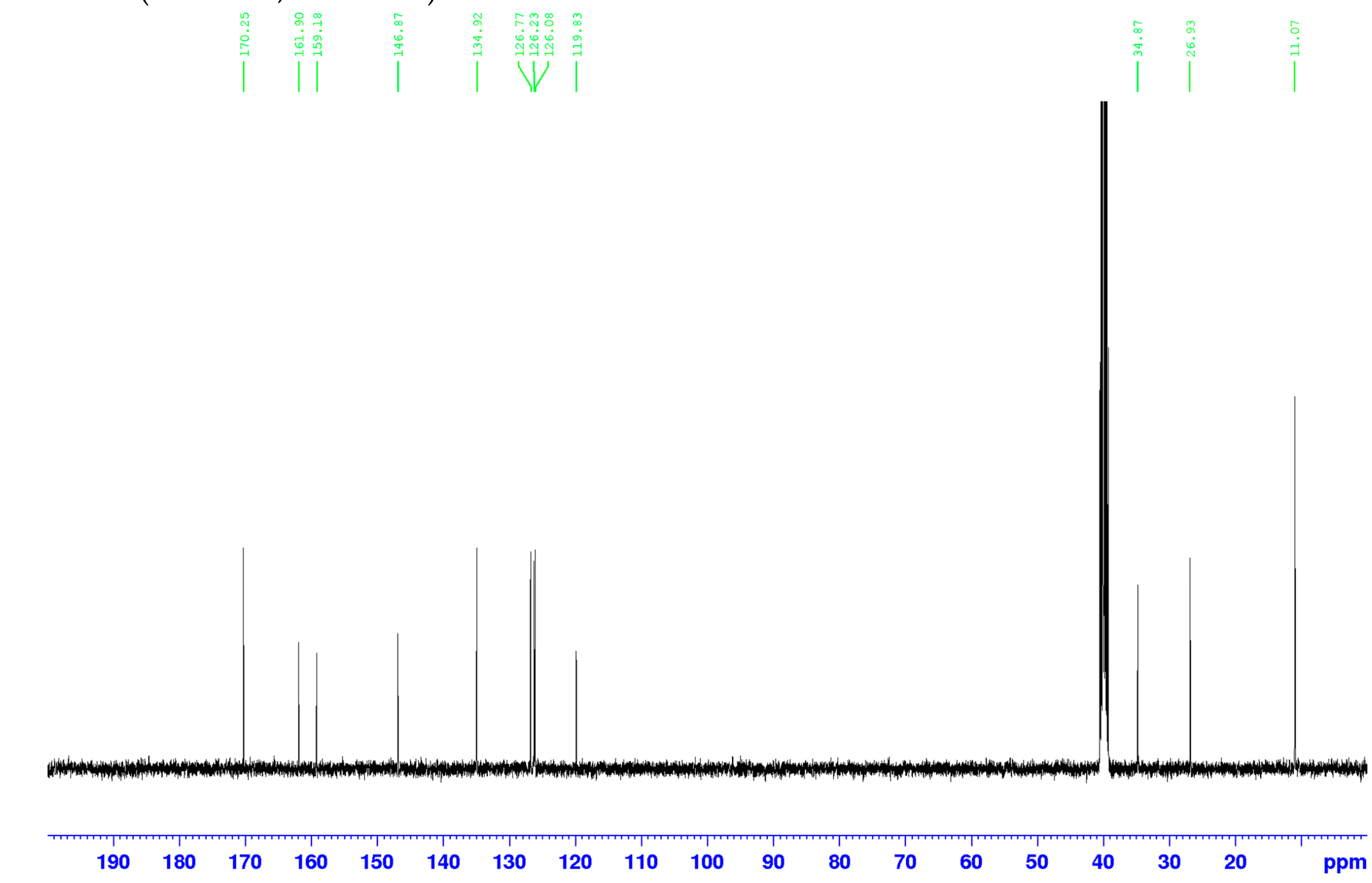

2-((4-Oxo-3-phenyl-3,4-dihydroquinazolin-2-yl)thio)acetic acid (**9b**)

^{1}H NMR (400 MHz, DMSO-d_6):

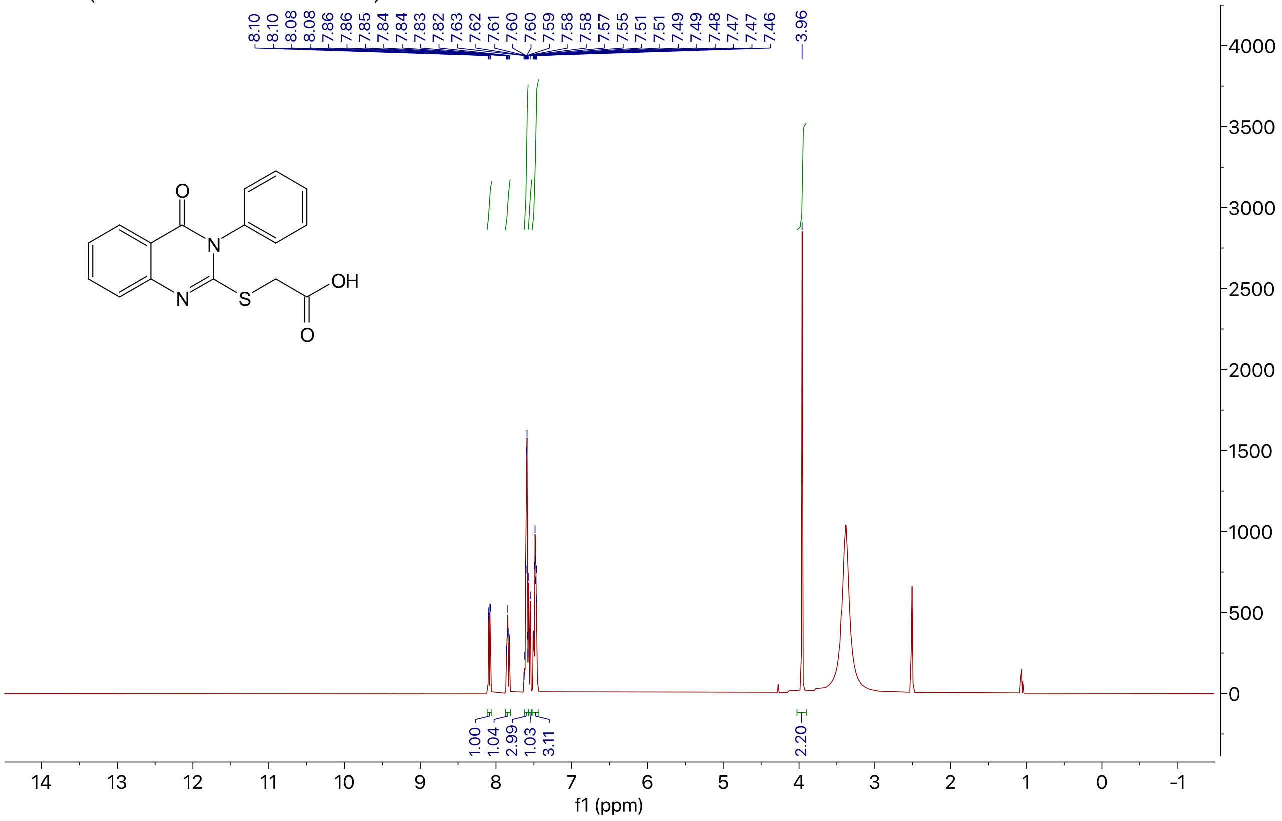

^{13}C NMR (100 MHz, DMSO-d_6):

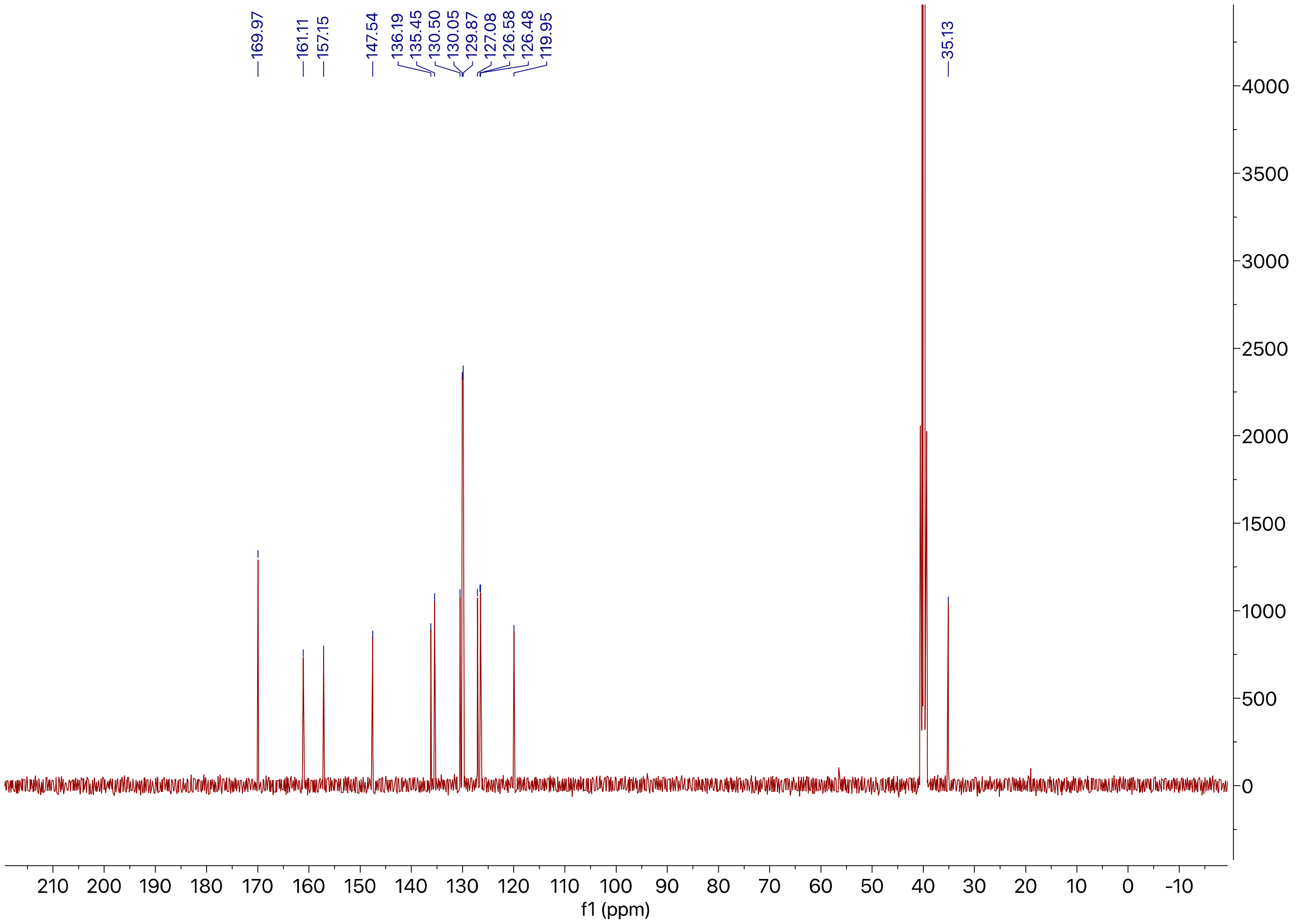

2-((6-Chloro-3-cyclopropyl-4-oxo-3,4-dihydroquinazolin-2-yl)thio)acetic acid (**9c**)

^{1}H NMR (400 MHz, DMSO-d_6):

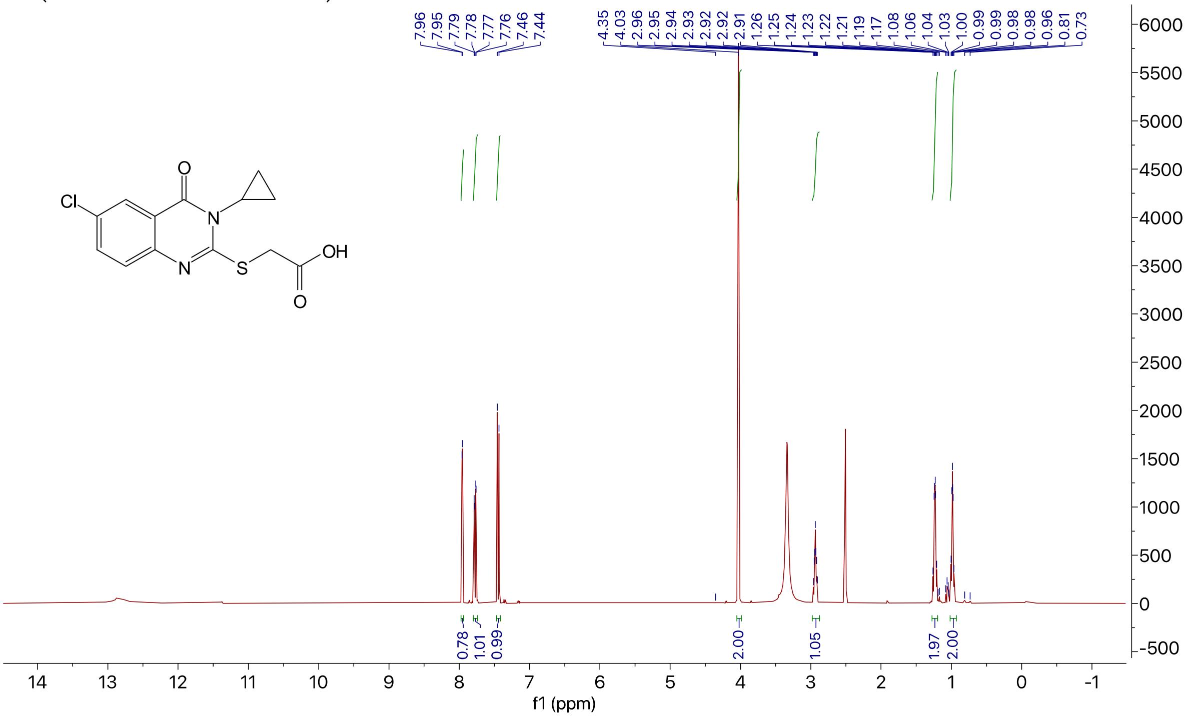

^{13}C NMR (100 MHz, DMSO-d_6):

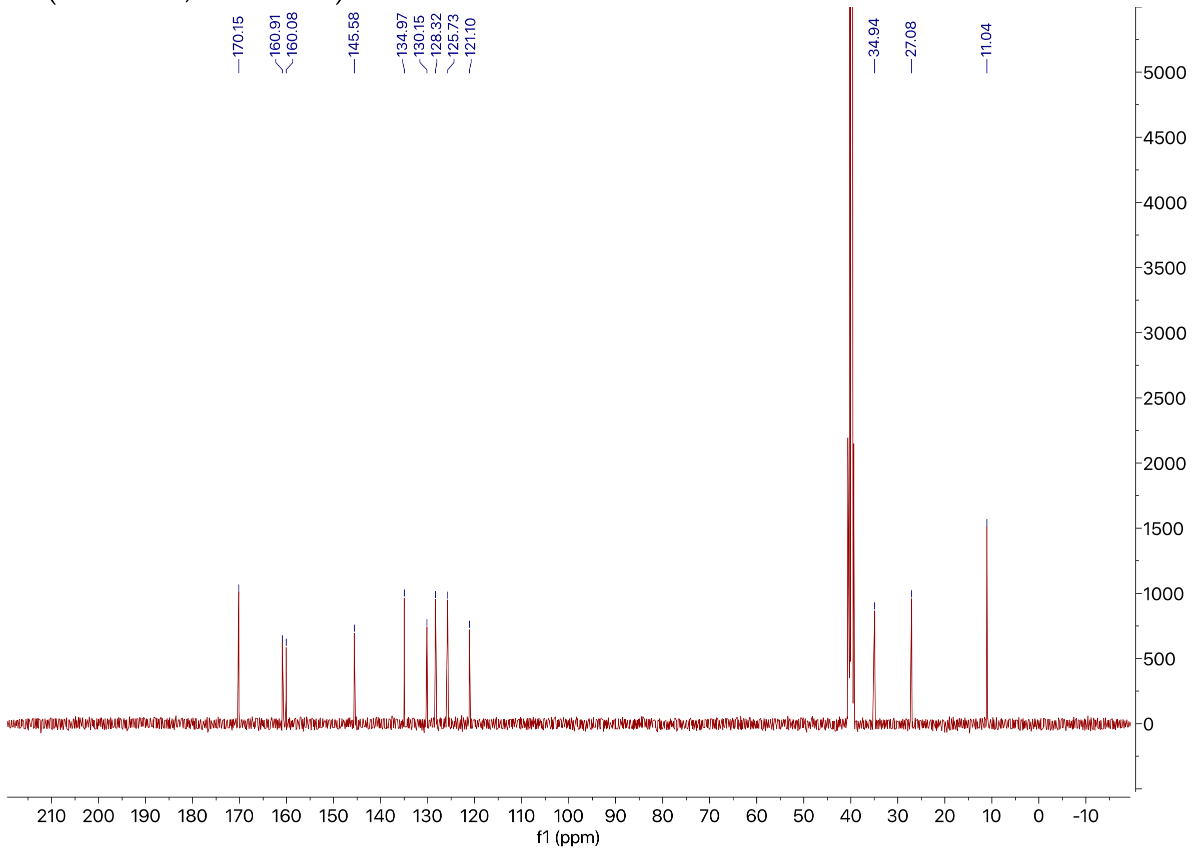

3-((4-Oxo-3-phenyl-3,4-dihydroquinazolin-2-yl)thio)propanoic acid (**9d**)

^{1}H NMR (400 MHz, DMSO-d_6):

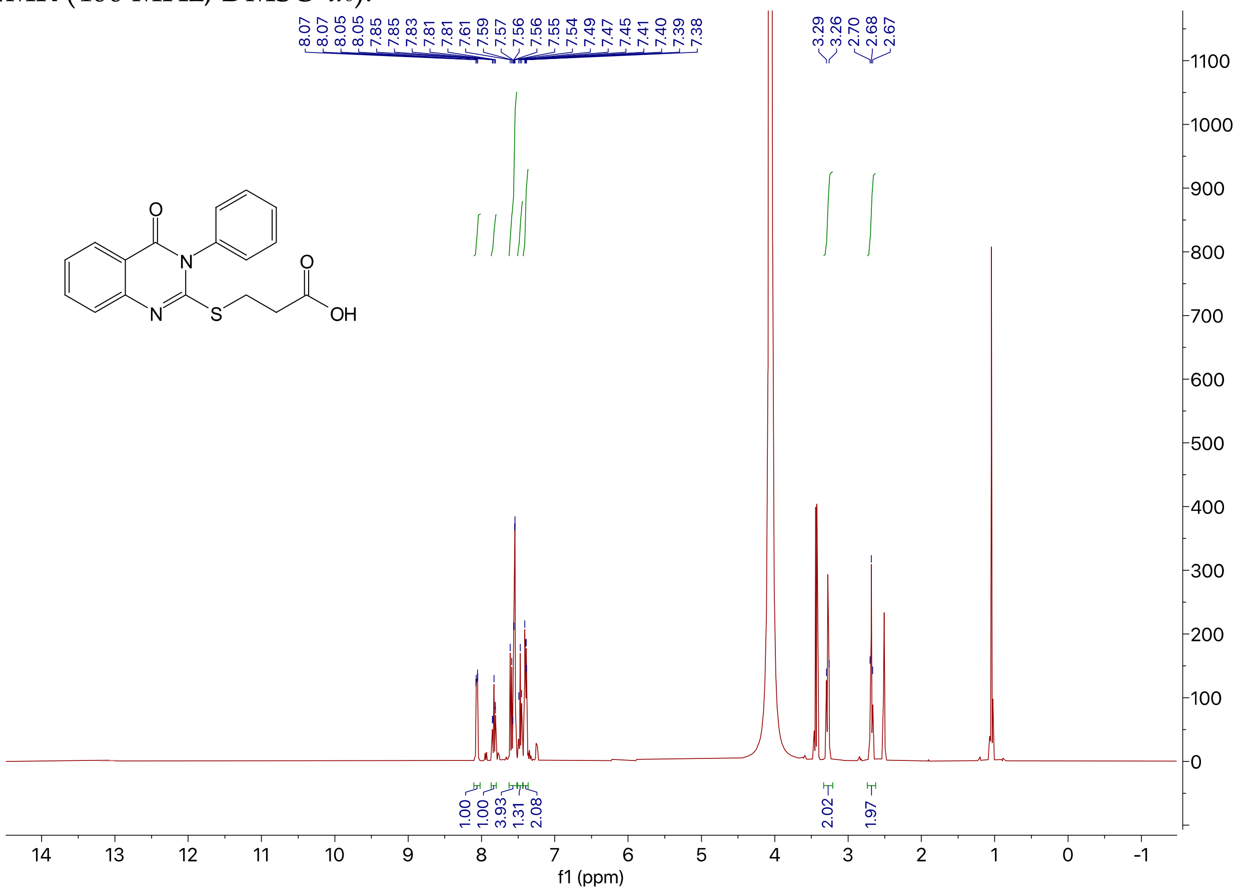

^{13}C NMR (100 MHz, DMSO-d_6):

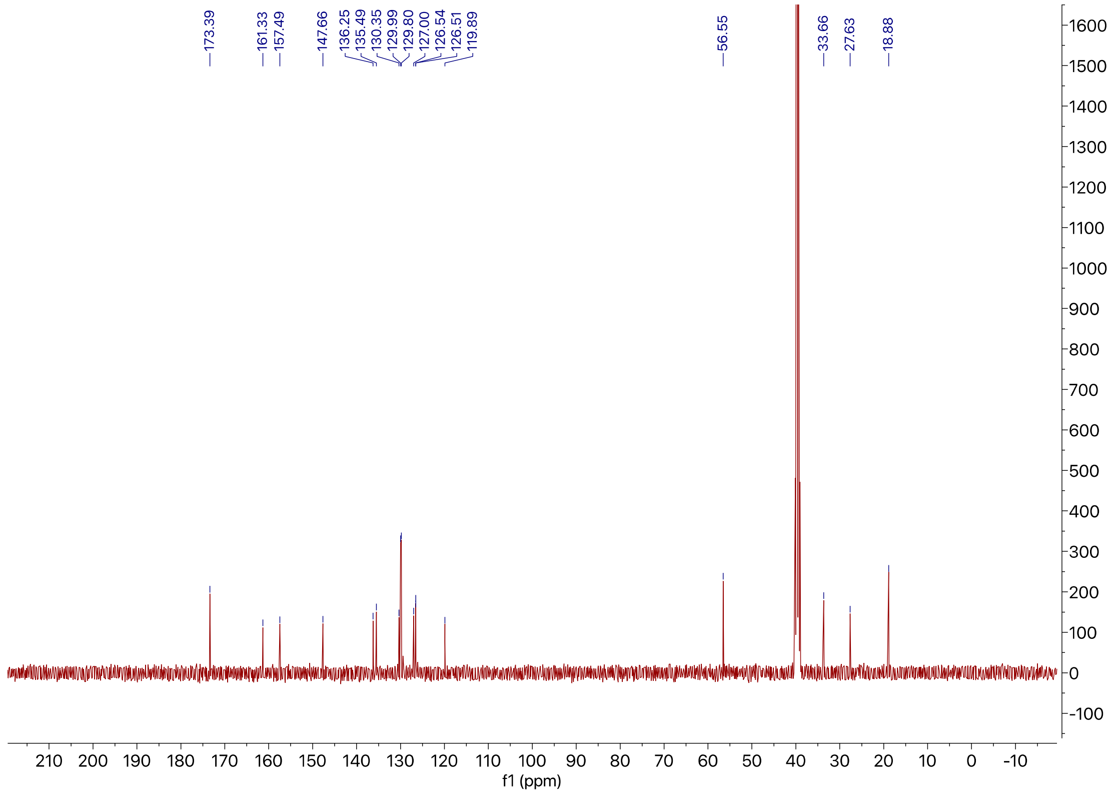

2-Chloro-*N*-(4-methoxyphenyl)acetamide (**12**)

^{1}H NMR (400 MHz, DMSO-d_6):

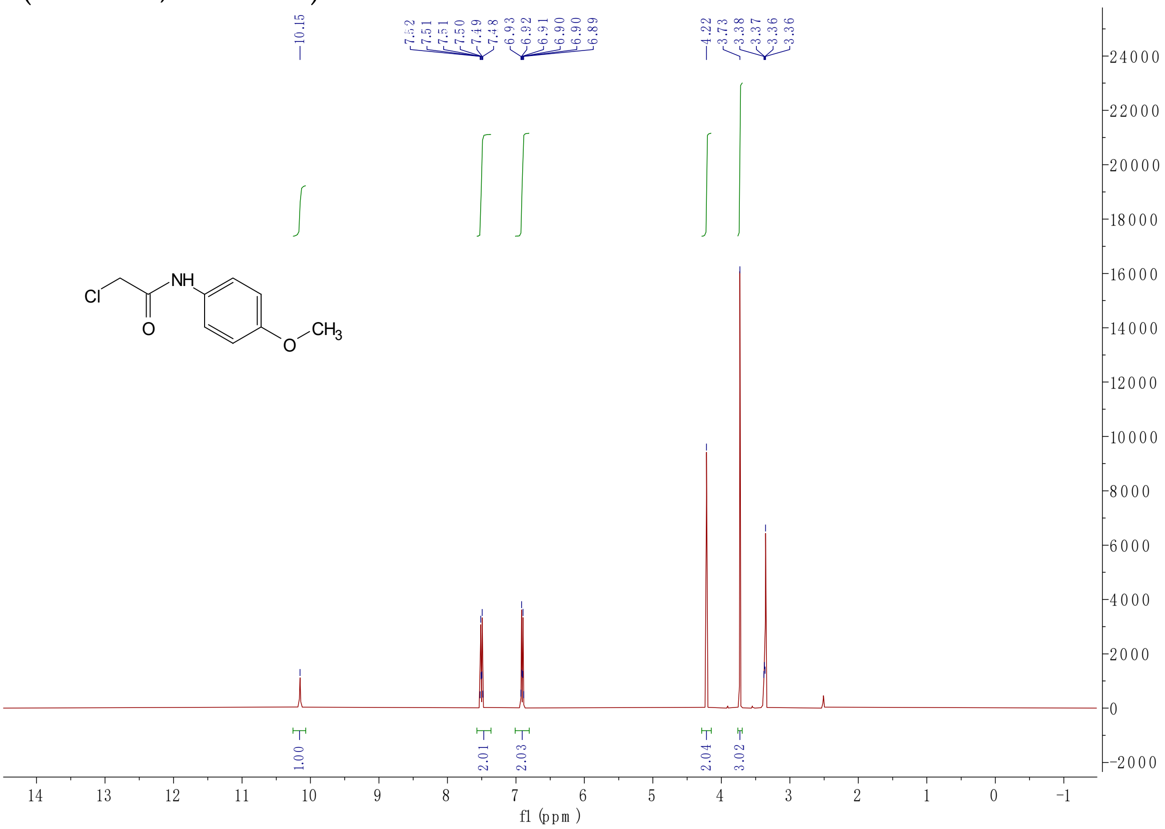

^{13}C NMR (100 MHz, DMSO-d_6):

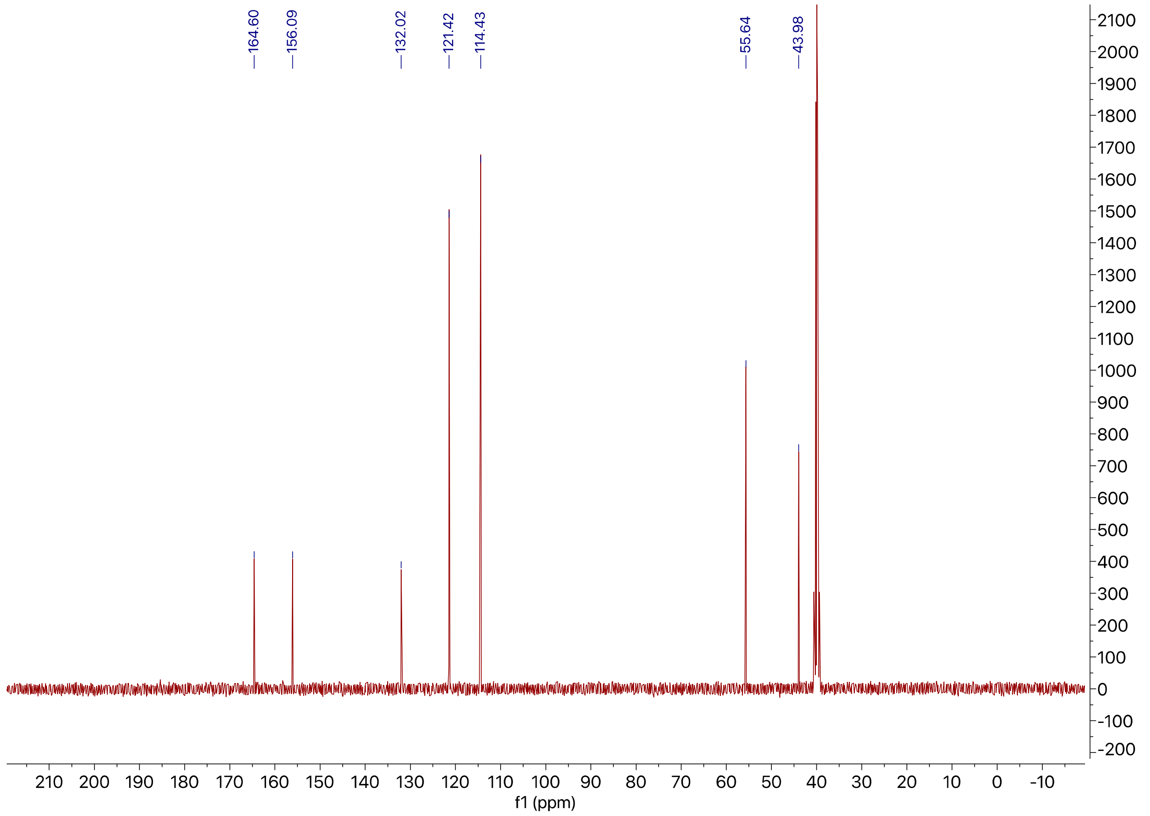

3-Cyclopropyl-2-(prop-2-yn-1-ylthio)quinazolin-4(3*H*)-one (**13a**)

^{1}H NMR (400 MHz, DMSO-d_6):

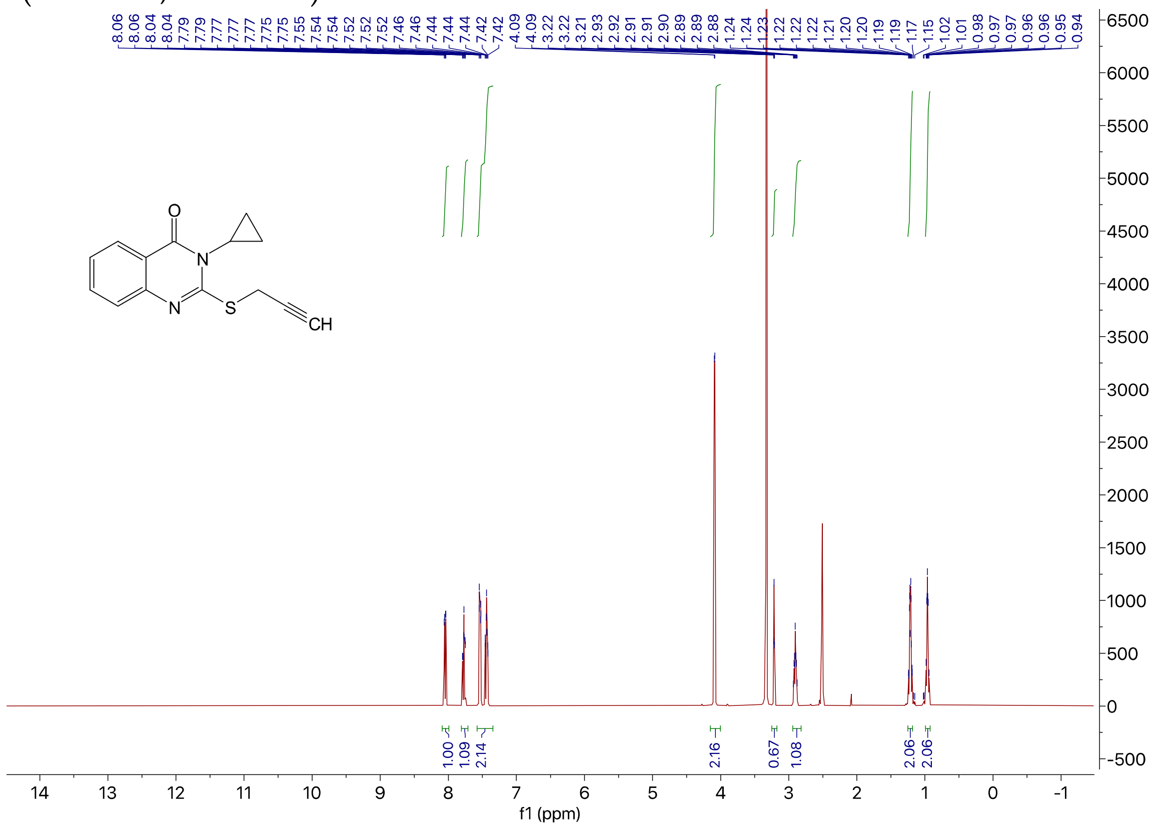

^{13}C NMR (100 MHz, DMSO-d_6):

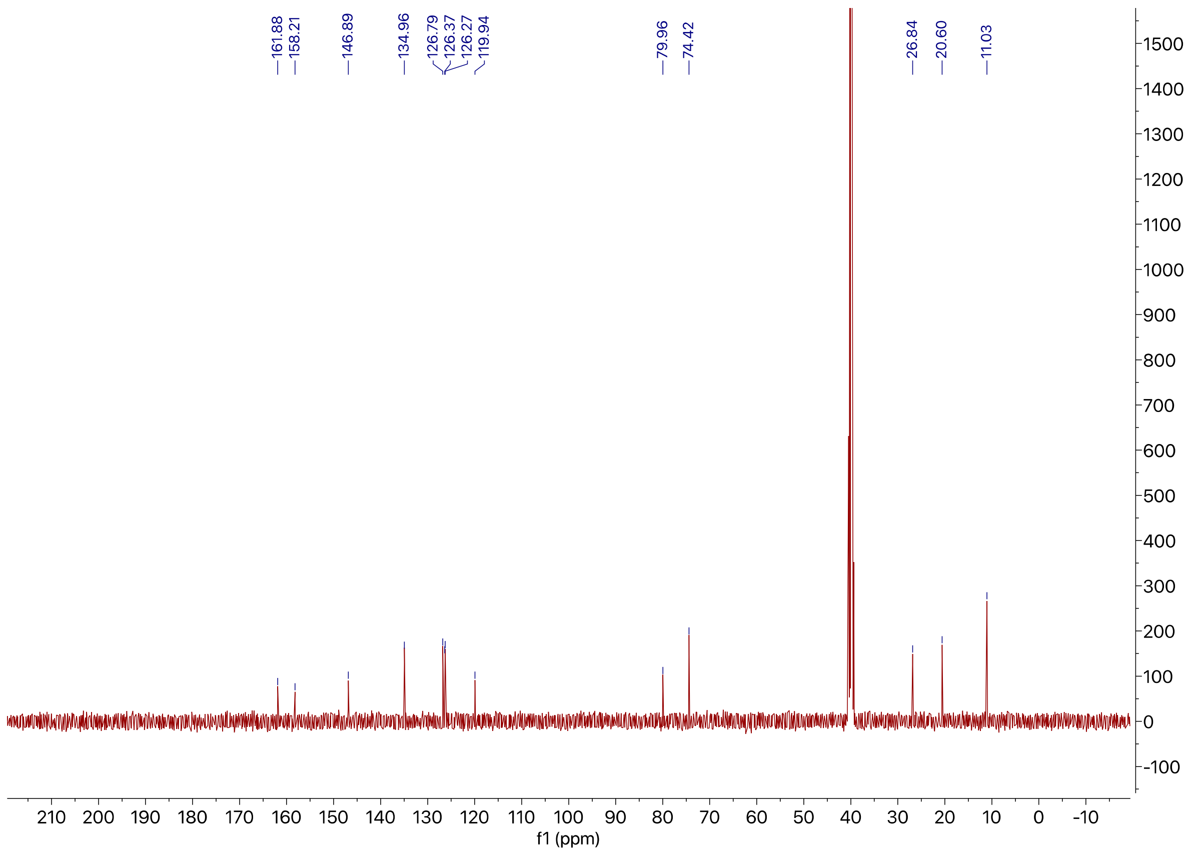
161.88
158.21
146.89
134.96
126.79
126.37
126.27
119.94
79.96
74.42
26.84
20.60
11.03
f1 (ppm)

3-Phenyl-2-(prop-2-yn-1-ylthio)quinazolin-4(3*H*)-one (**13b**)

^{1}H NMR (400 MHz, DMSO-d_6):

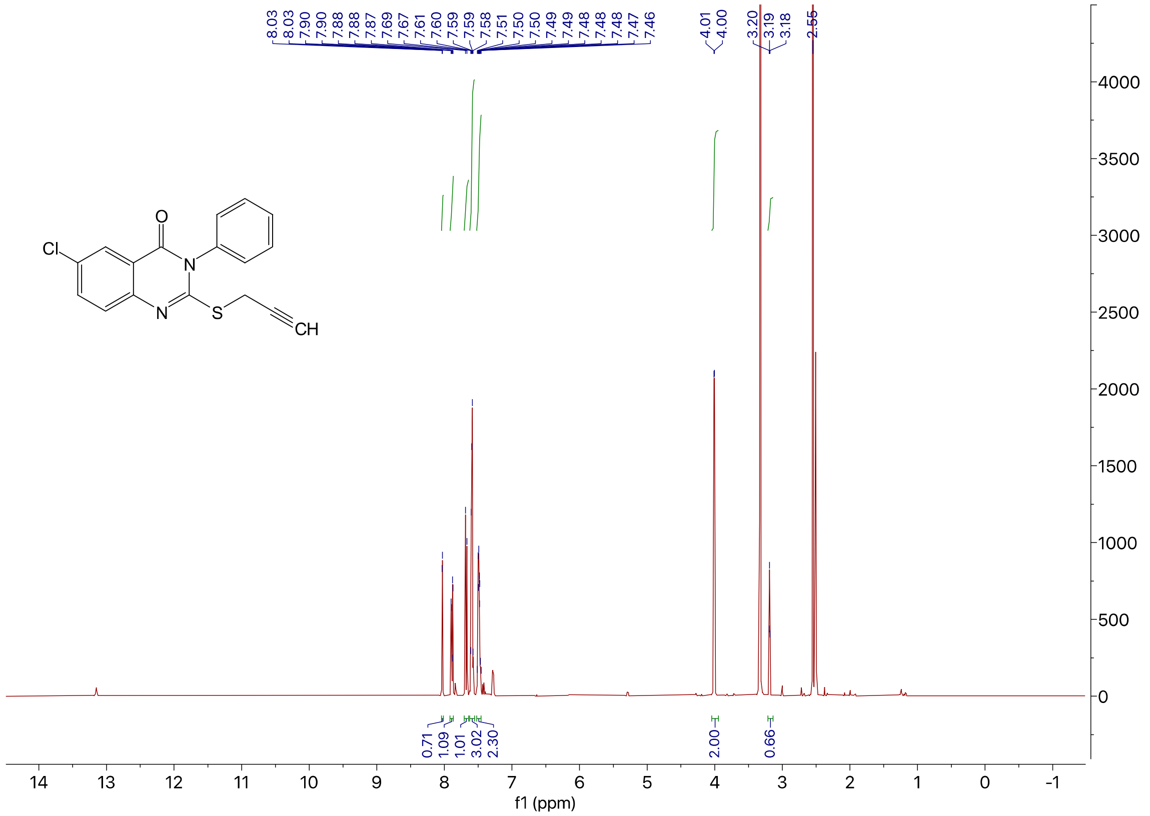

^{13}C NMR (100 MHz, DMSO-d_6):

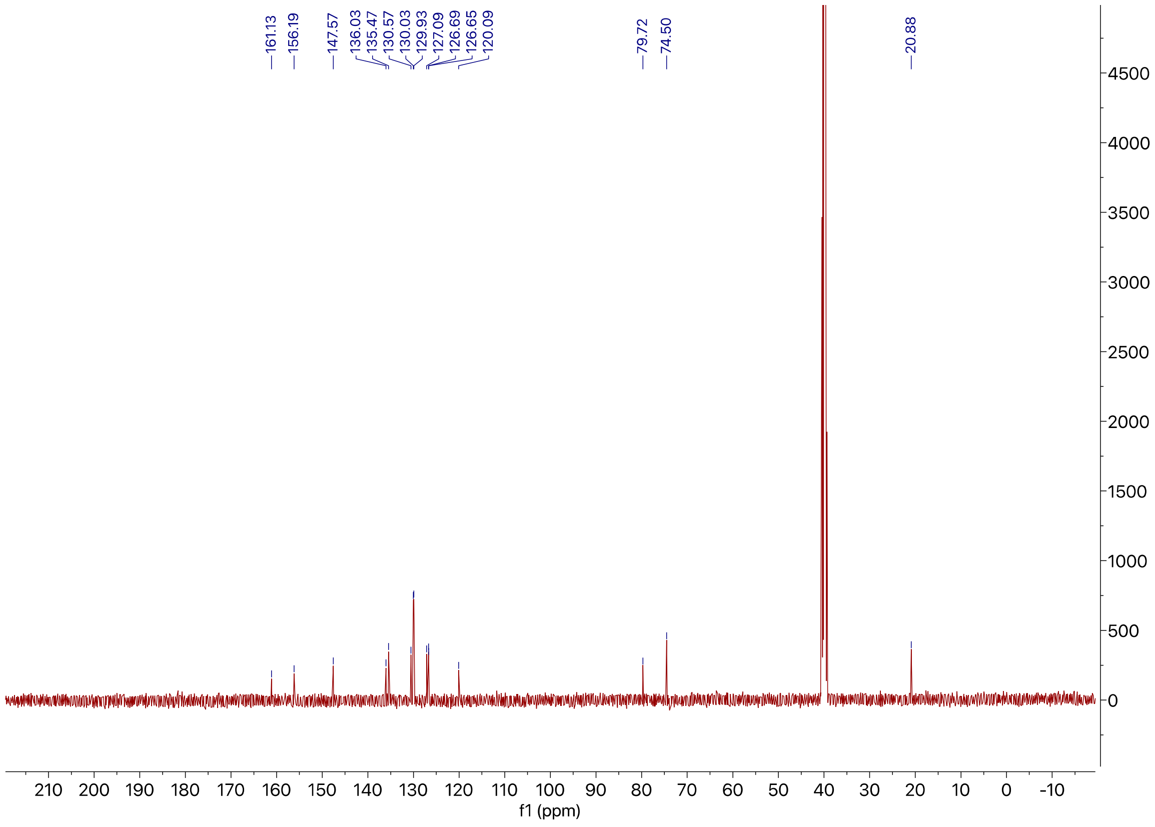

6-Chloro-3-cyclopropyl-2-(prop-2-yn-1-ylthio)quinazolin-4(3*H*)-one (**13c**)

^{1}H NMR (400 MHz, DMSO-d_6):

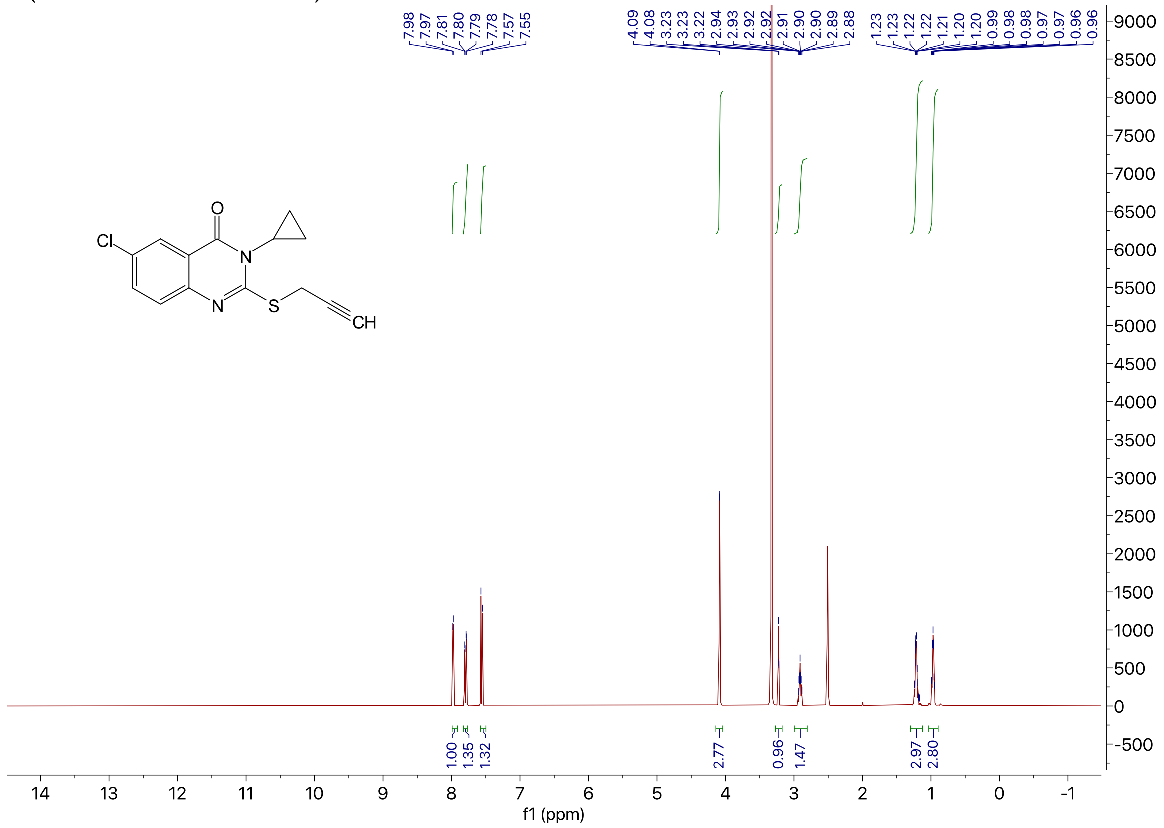

^{13}C NMR (100 MHz, DMSO-d_6):

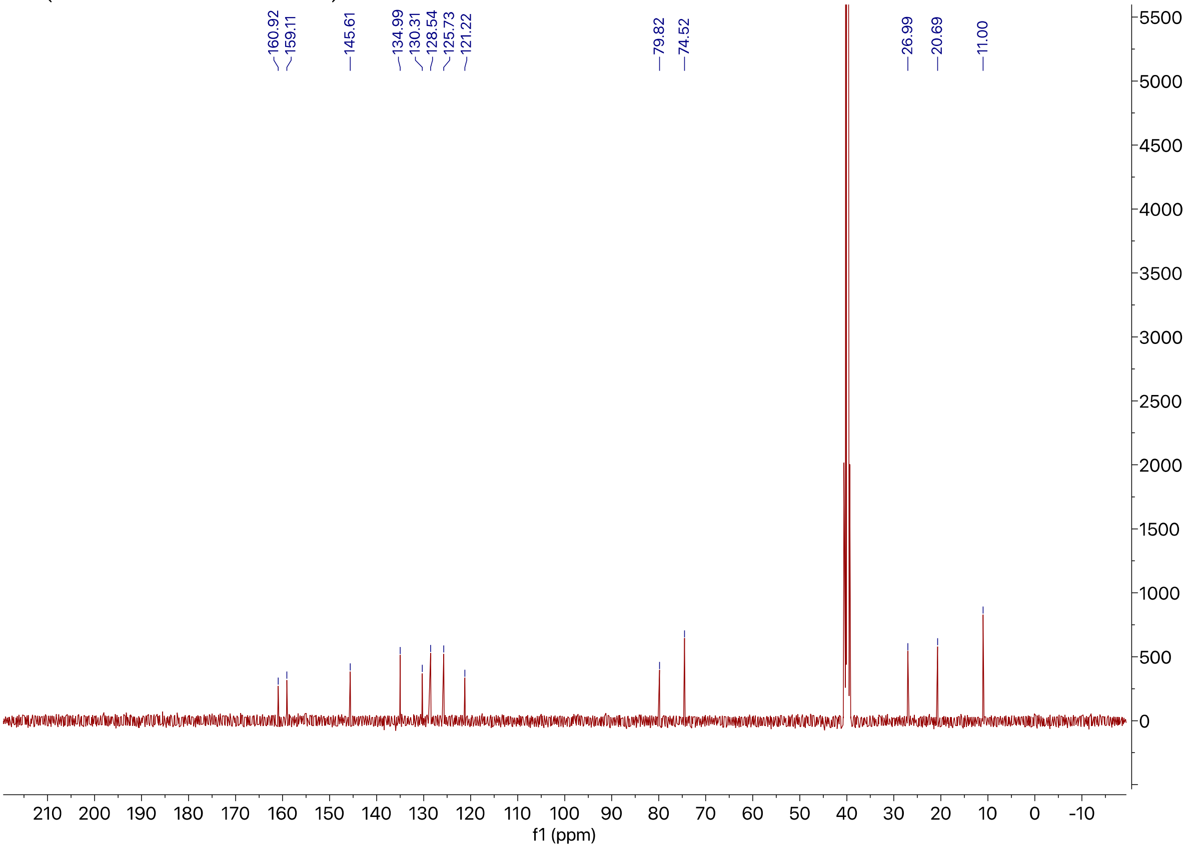

2-Bromo-*N*-(4-methoxyphenyl)acetamide (**15a**)

^{1}H NMR (400 MHz, DMSO-d_6):

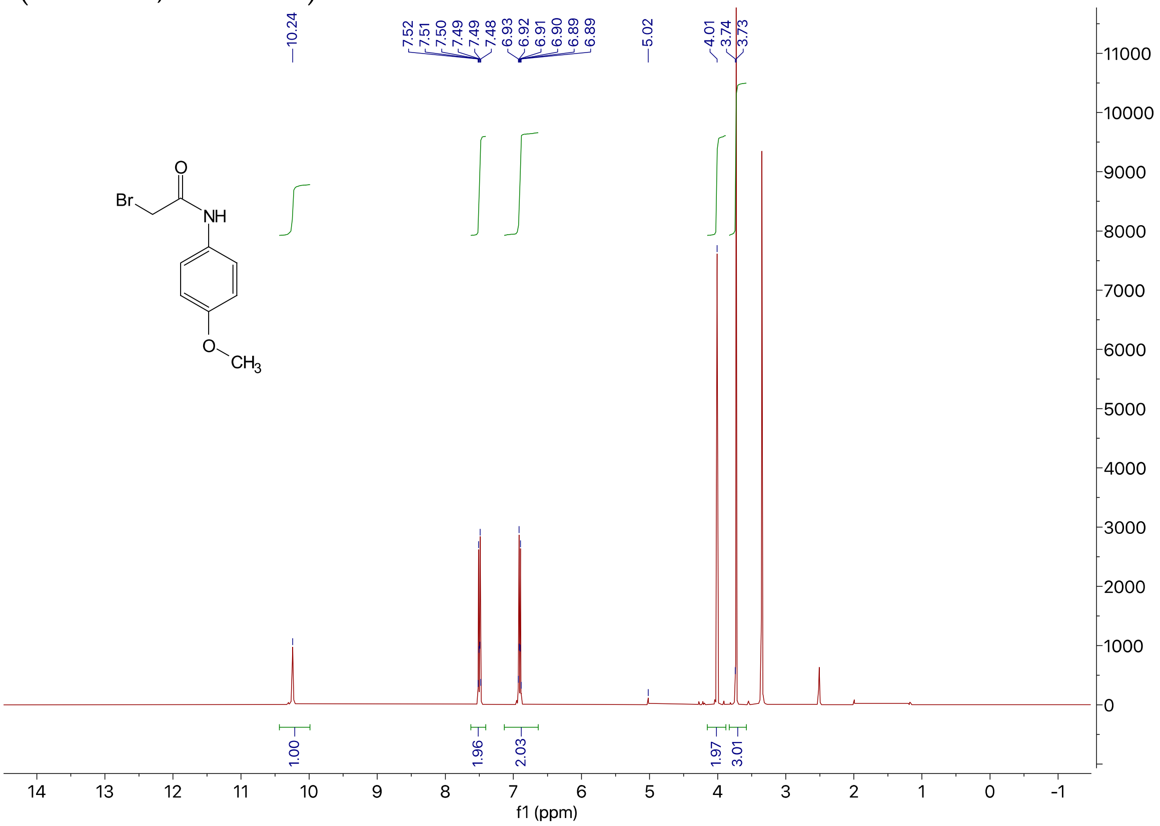

^{13}C NMR (100 MHz, DMSO-d_6):

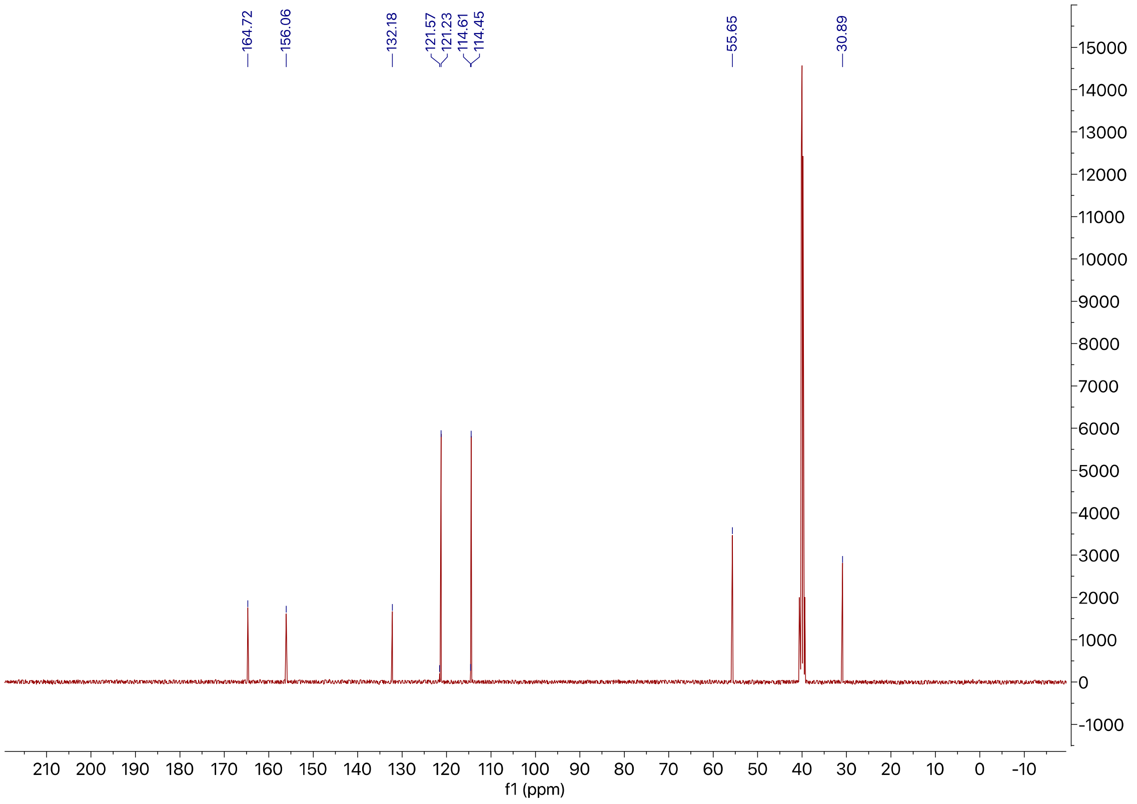

2-Bromo-*N*-phenylacetamide (**15b**)

^{1}H NMR (400 MHz, DMSO-d_6):

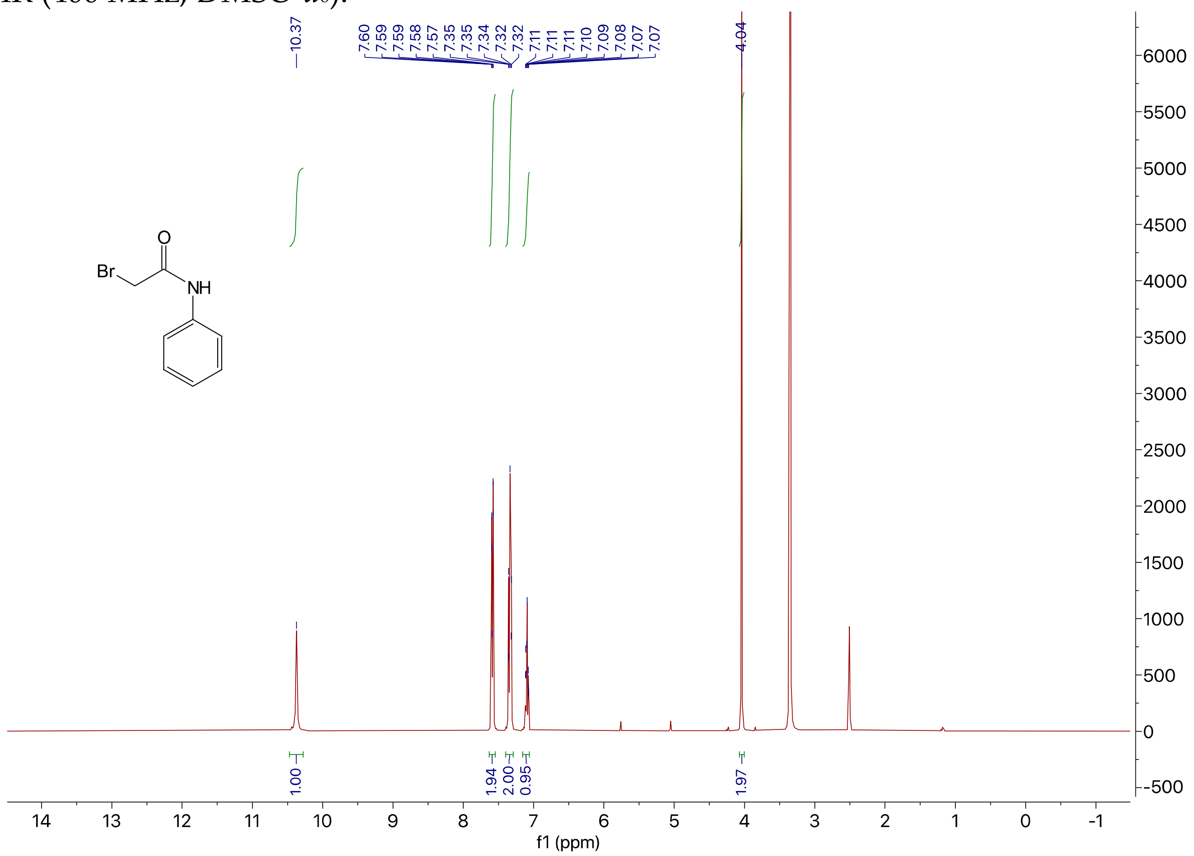

^{13}C NMR (100 MHz, DMSO-d_6):

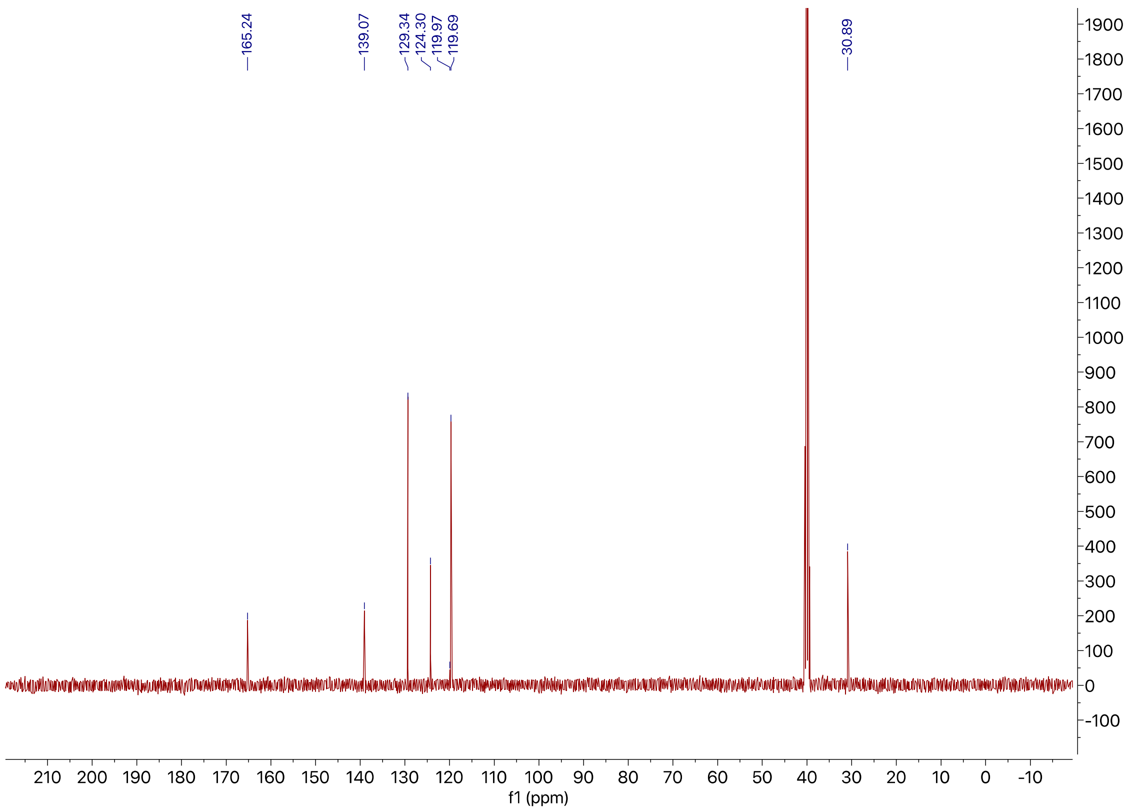

165.24
139.07
129.34
124.30
119.97
119.69
30.89
f1 (ppm)

2-Bromo-*N*-(4-nitrophenyl)acetamide (**15c**)

^{1}H NMR (400 MHz, DMSO-d_6):

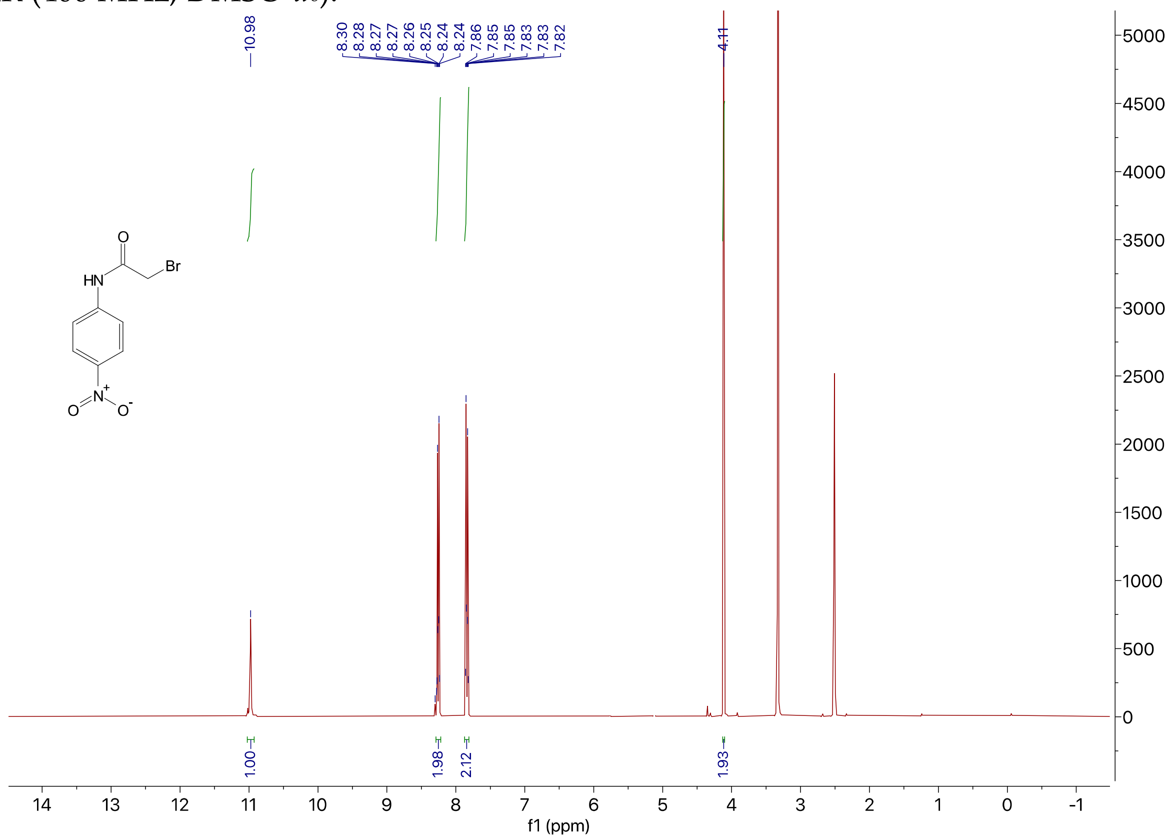

^{13}C NMR (100 MHz, DMSO-d_6):

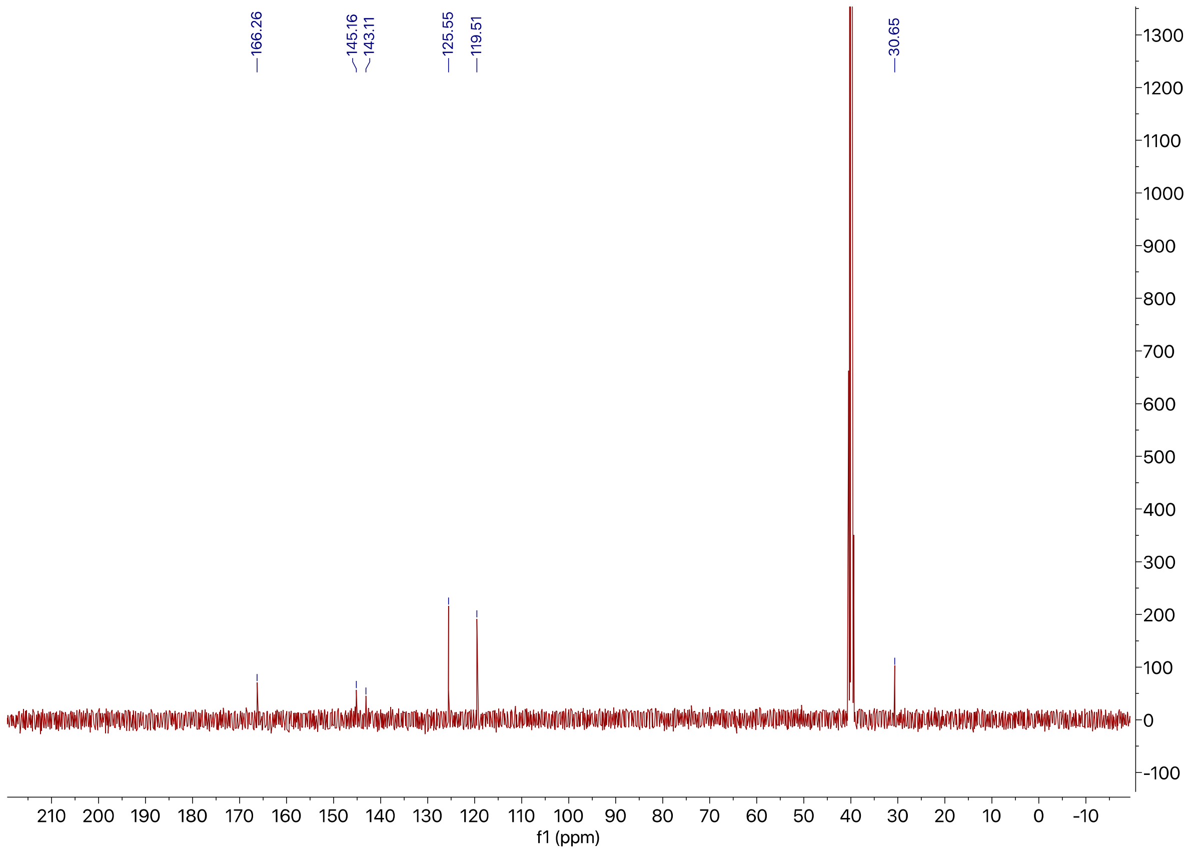

High resolution mass spectra (HRMS) of synthesised compounds

N-Butyl-2-((3-cyclopropyl-4-oxo-3,4-dihydroquinazolin-2-yl)thio)acetamide (**5a**)

Full spectrum

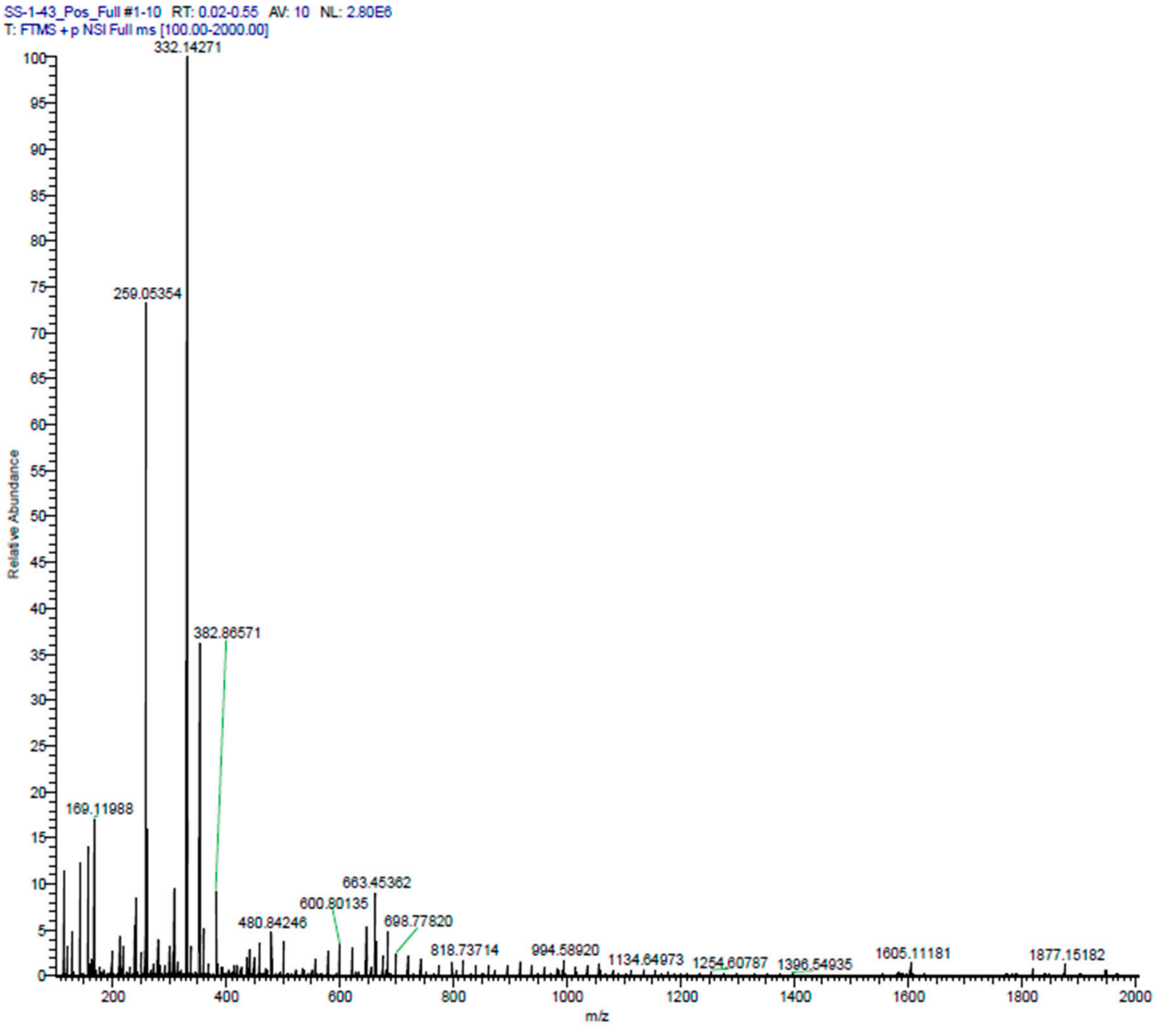

Zoomed spectrum (Top spectrum: measured mass; bottom spectrum: calculated mass)

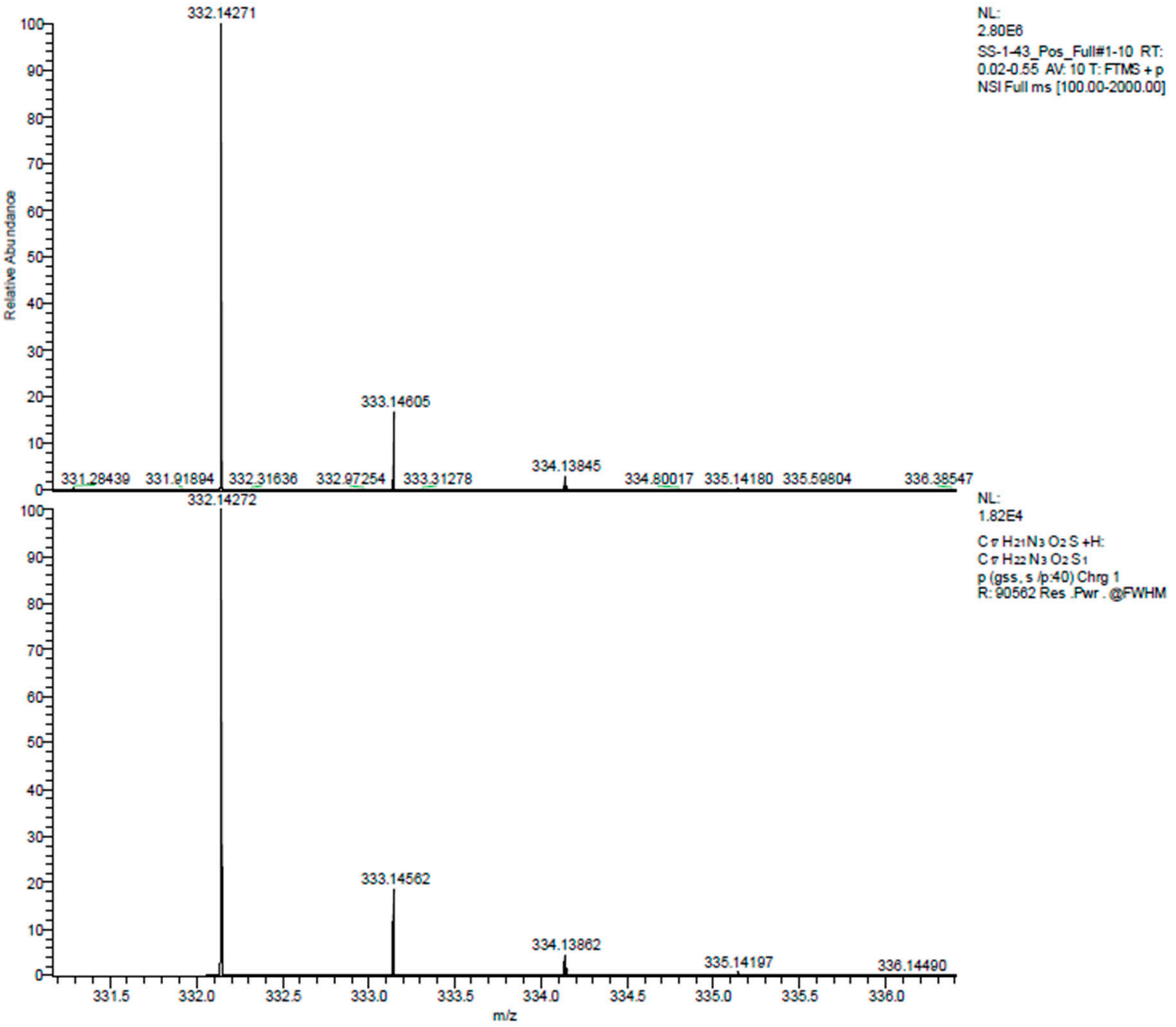

N-Benzyl-2-((3-cyclopropyl-4-oxo-3,4-dihydroquinazolin-2-yl)thio)acetamide (**5b**)

Full spectrum

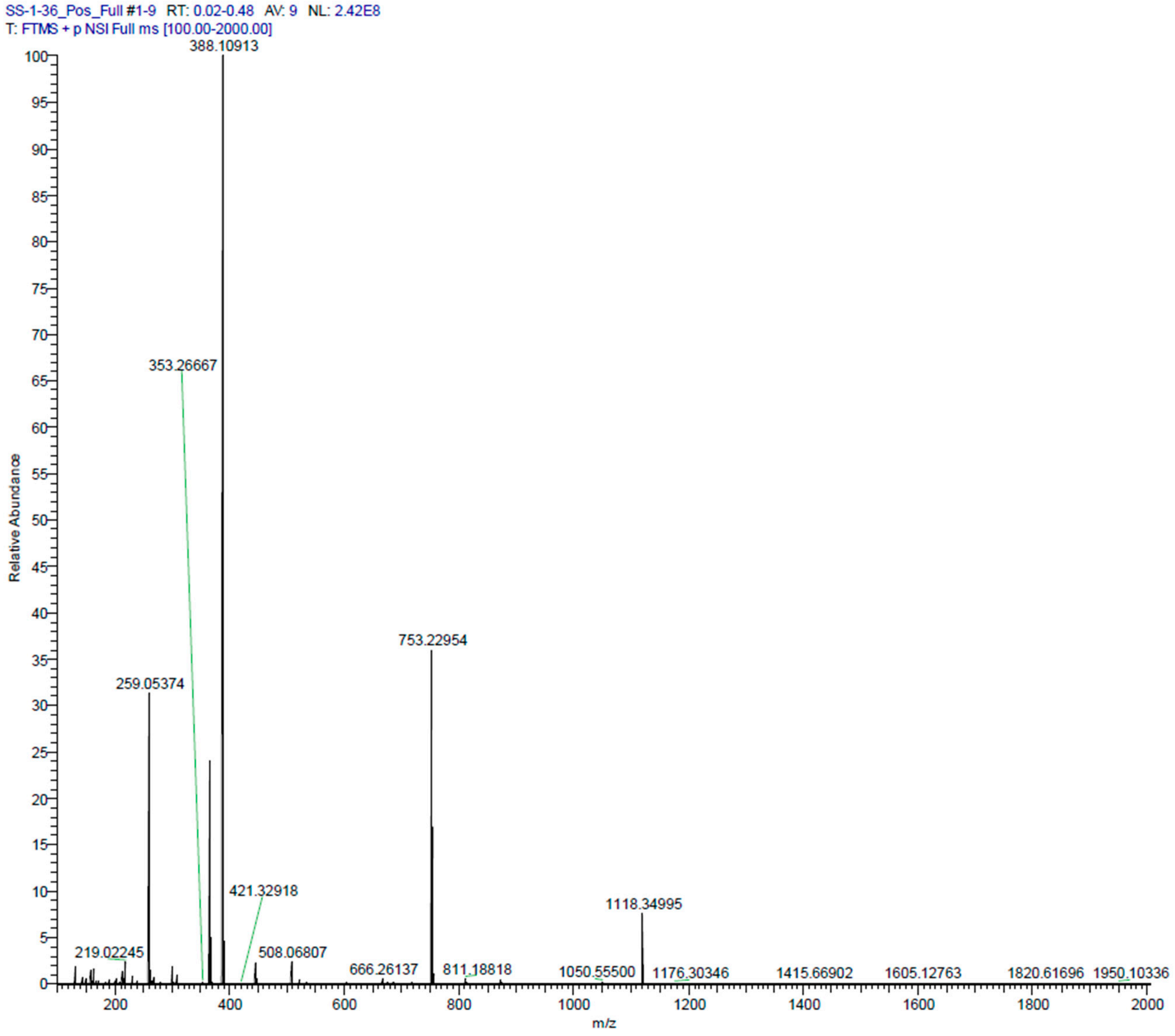

Zoomed spectrum (Top spectrum: measured mass; bottom spectrum: calculated mass)

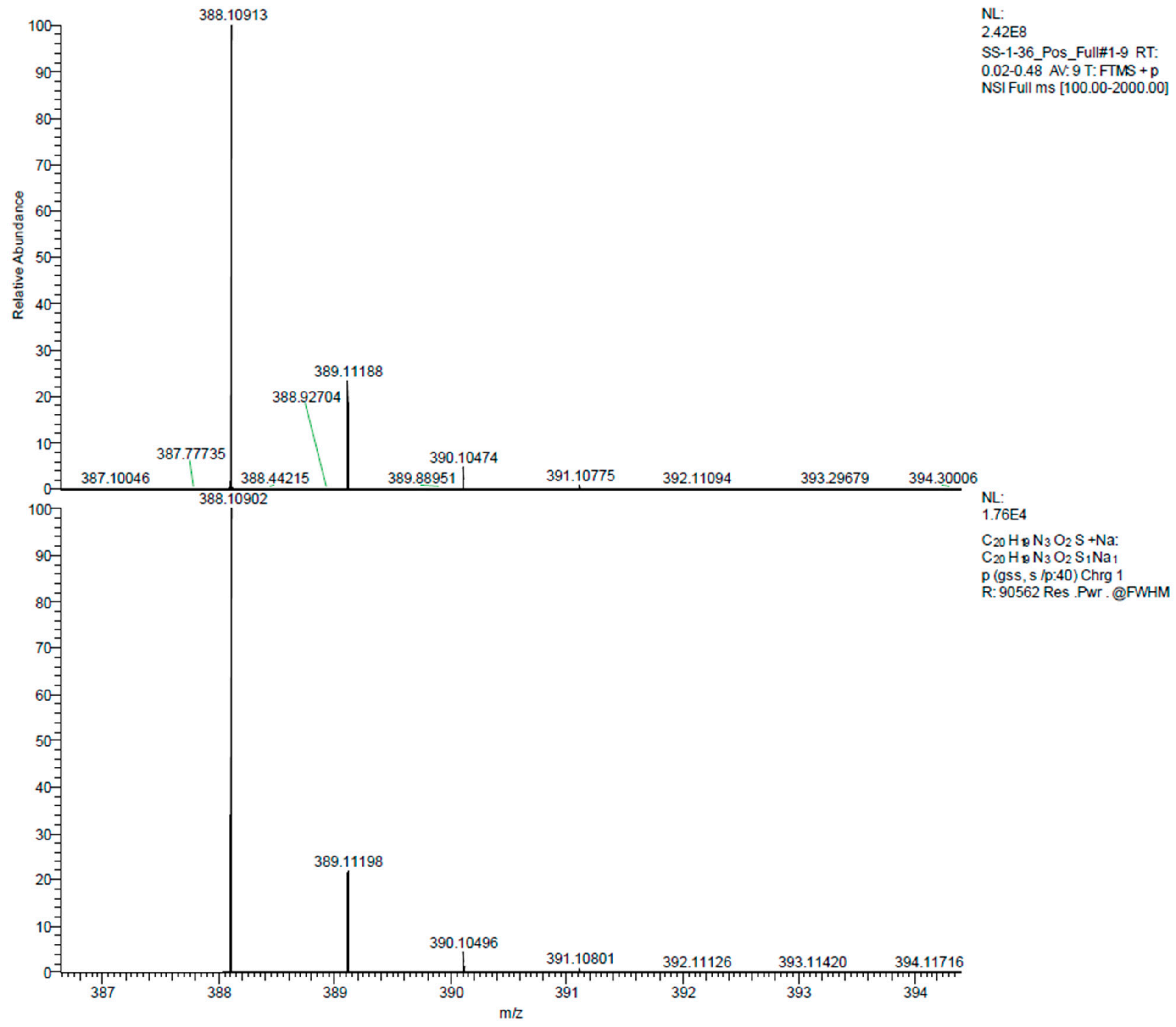

N-Benzyl-2-((4-oxo-3-phenyl-3,4-dihydroquinazolin-2-yl)thio)acetamide (**5c**)

Full spectrum

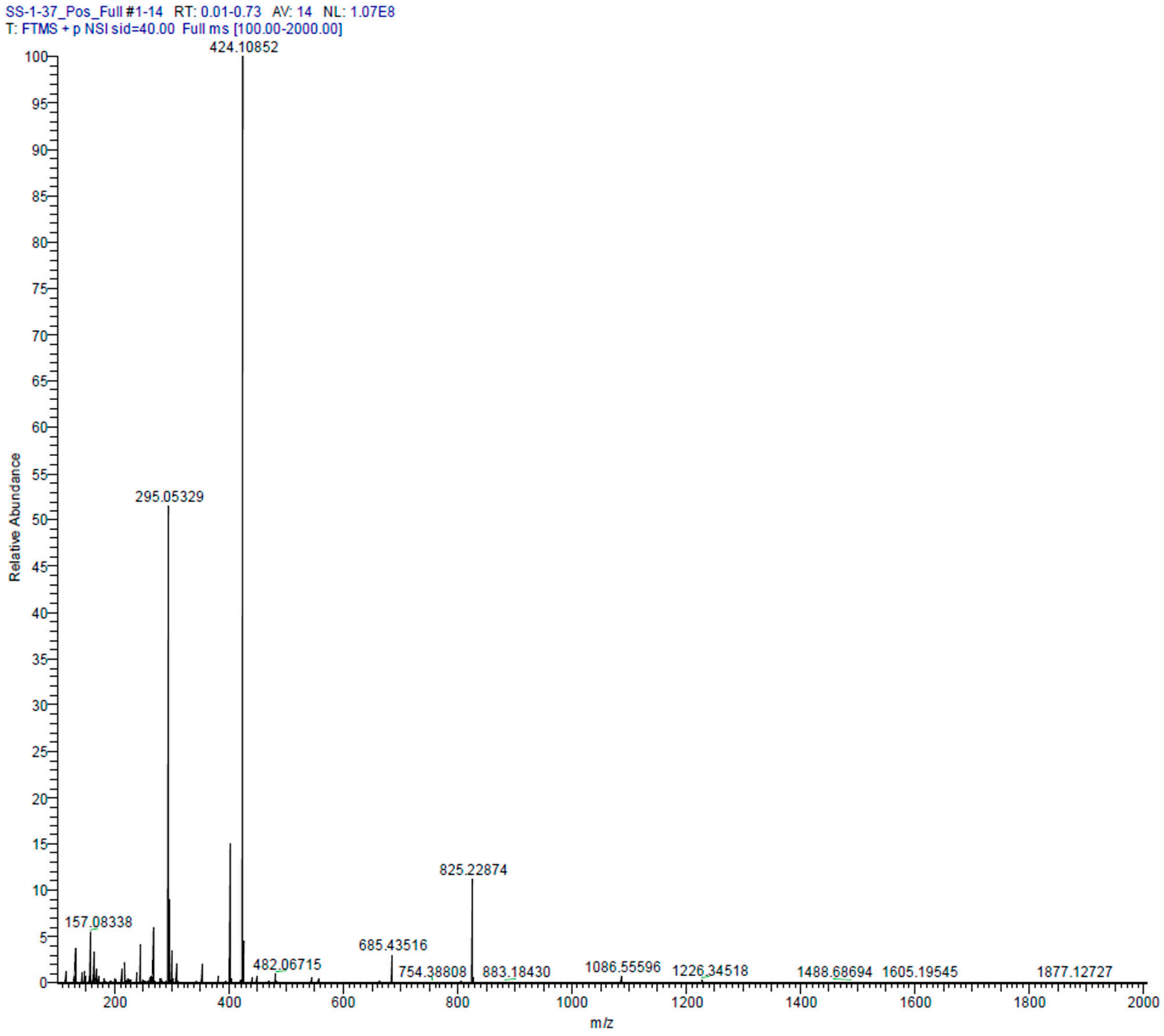

Zoomed spectrum (Top spectrum: measured mass; bottom spectrum: calculated mass)

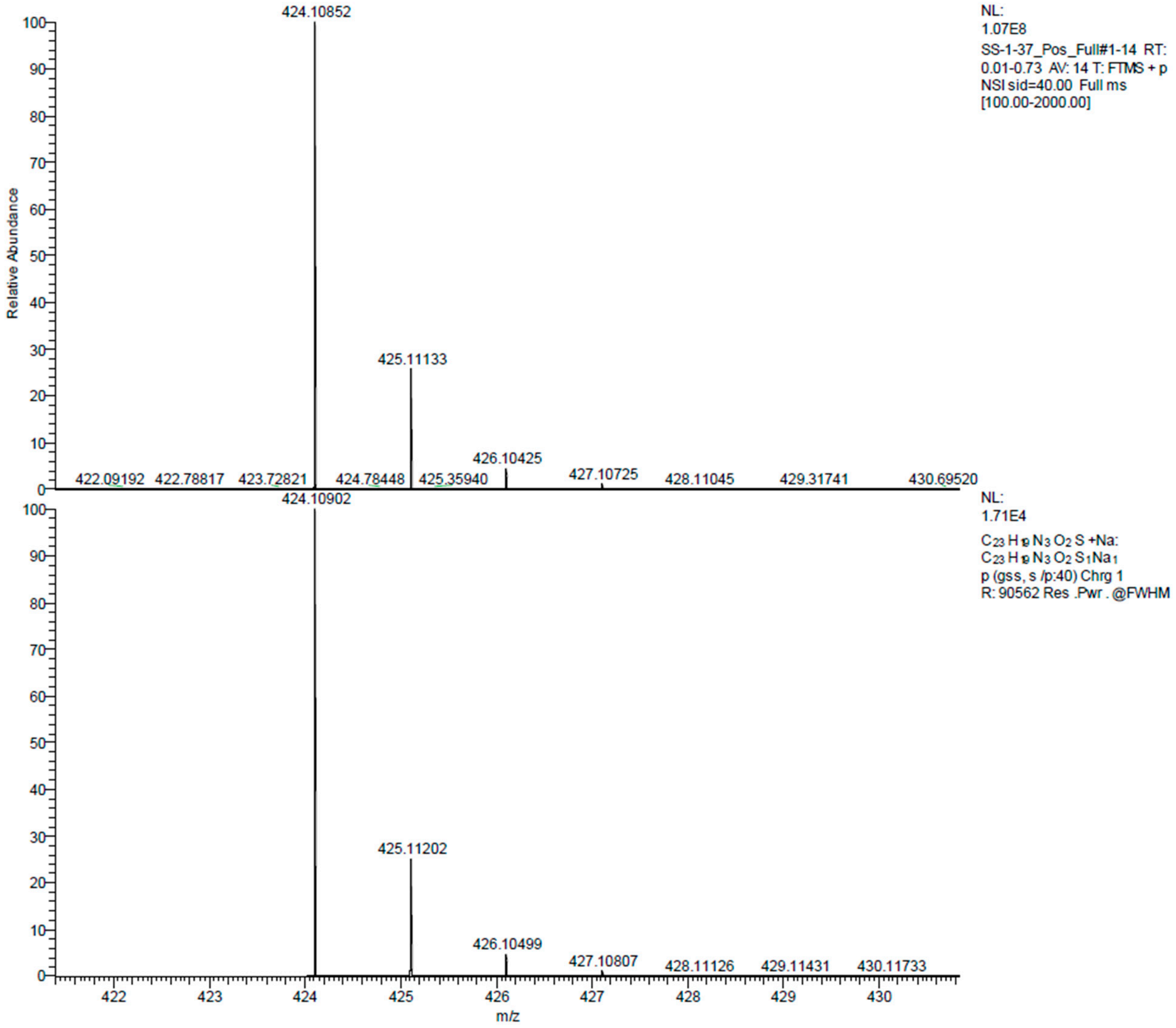

N-Benzyl-2-((6-chloro-3-cyclopropyl-4-oxo-3,4-dihydroquinazolin-2-yl)thio)acetamide (**5d**)

Full spectrum

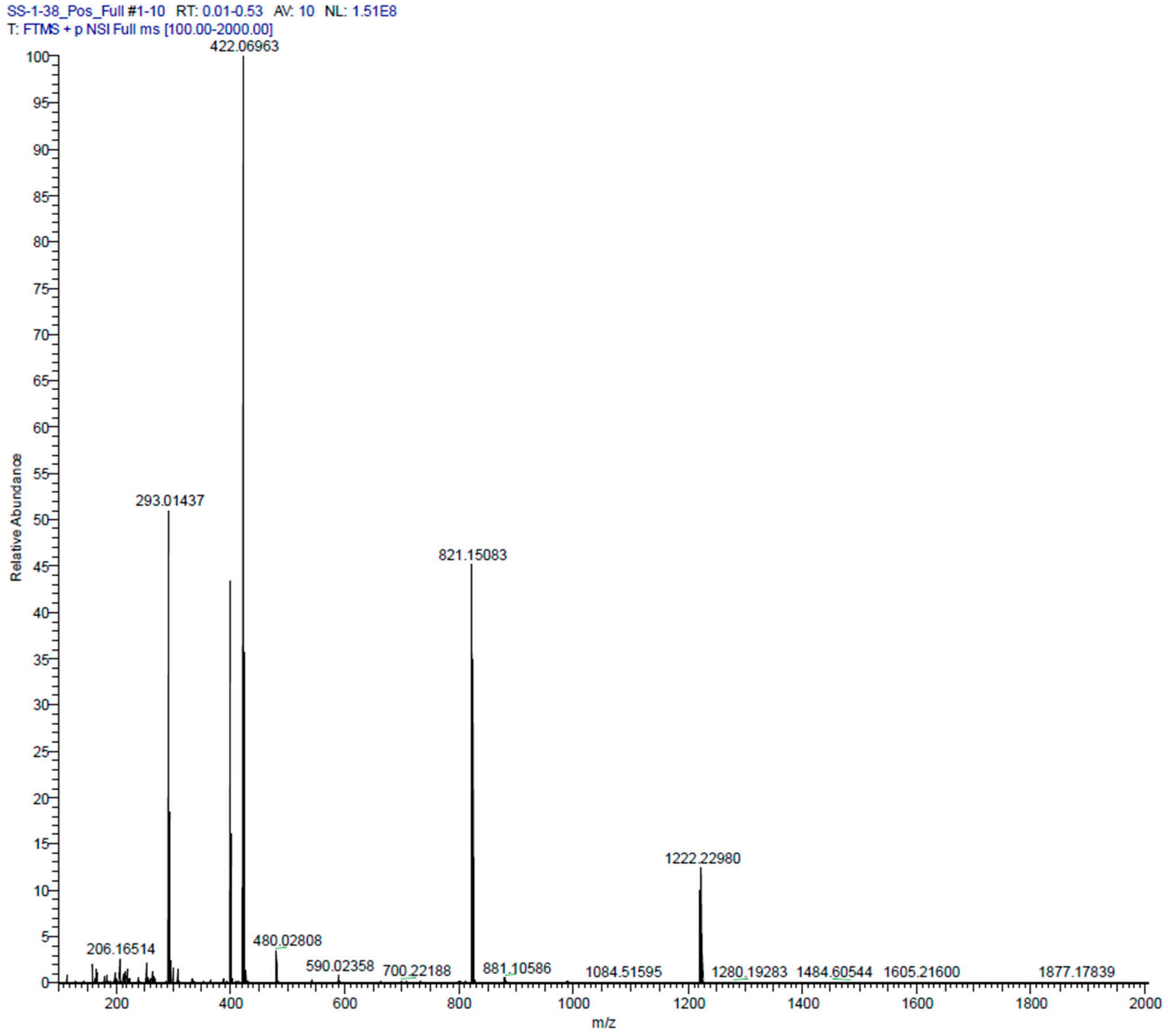

Zoomed spectrum (Top spectrum: measured mass; bottom spectrum: calculated mass)

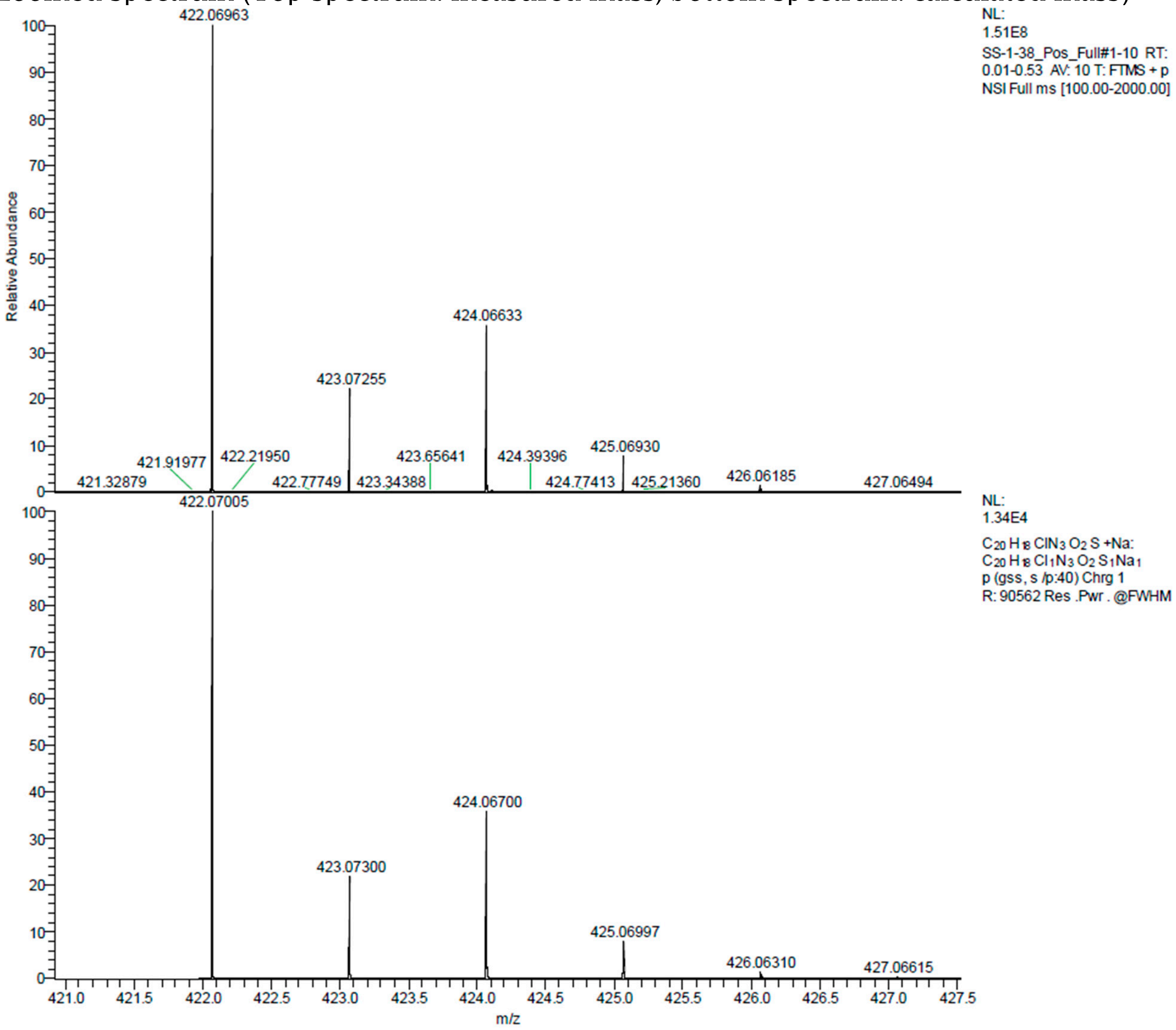

N-(4-Methoxyphenyl)-3-((4-oxo-3-phenyl-3,4-dihydroquinazolin-2-yl)thio)propenamide (**5f**)

Full spectrum

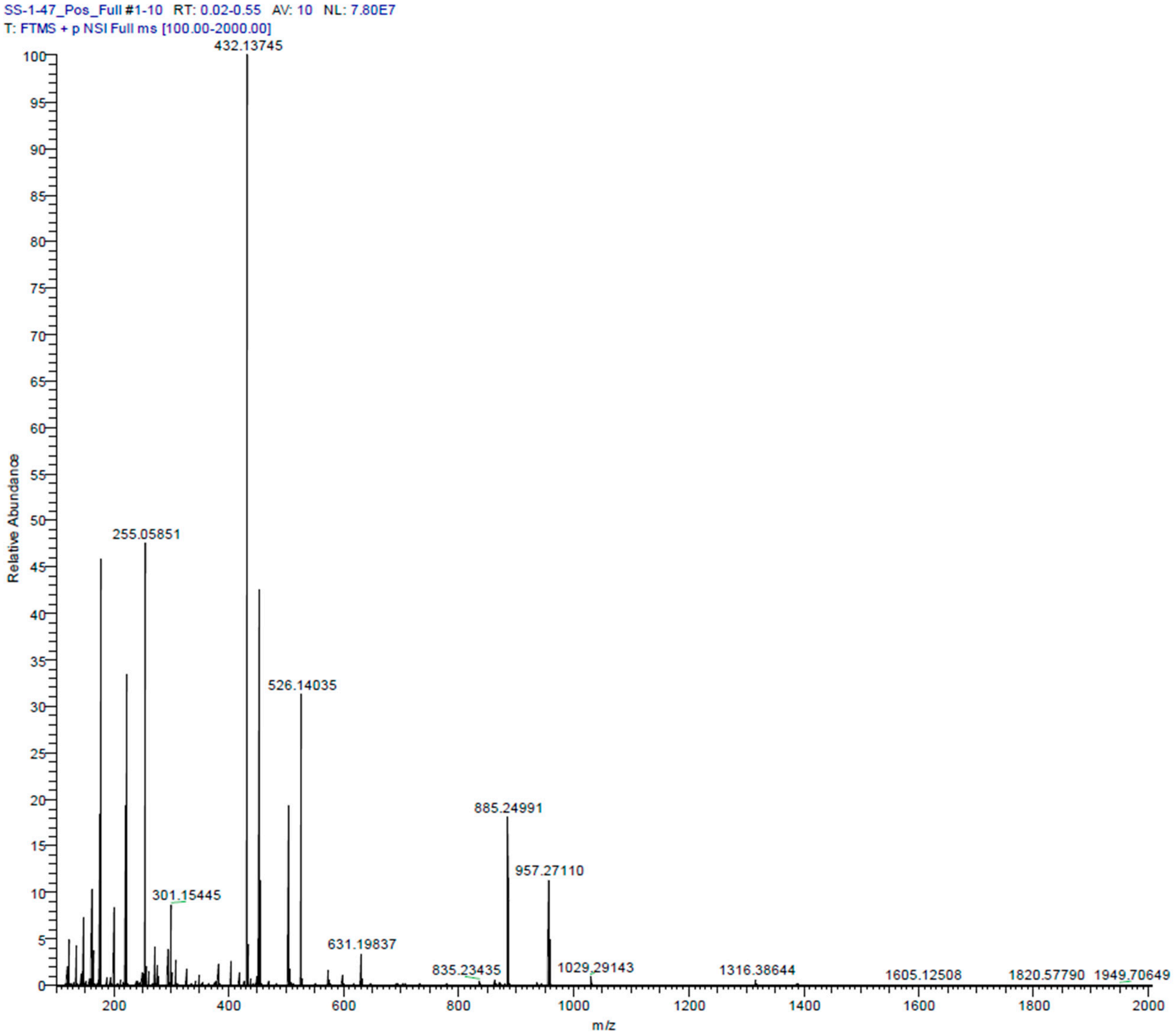

Zoomed spectrum (Top spectrum: measured mass; bottom spectrum: calculated mass)

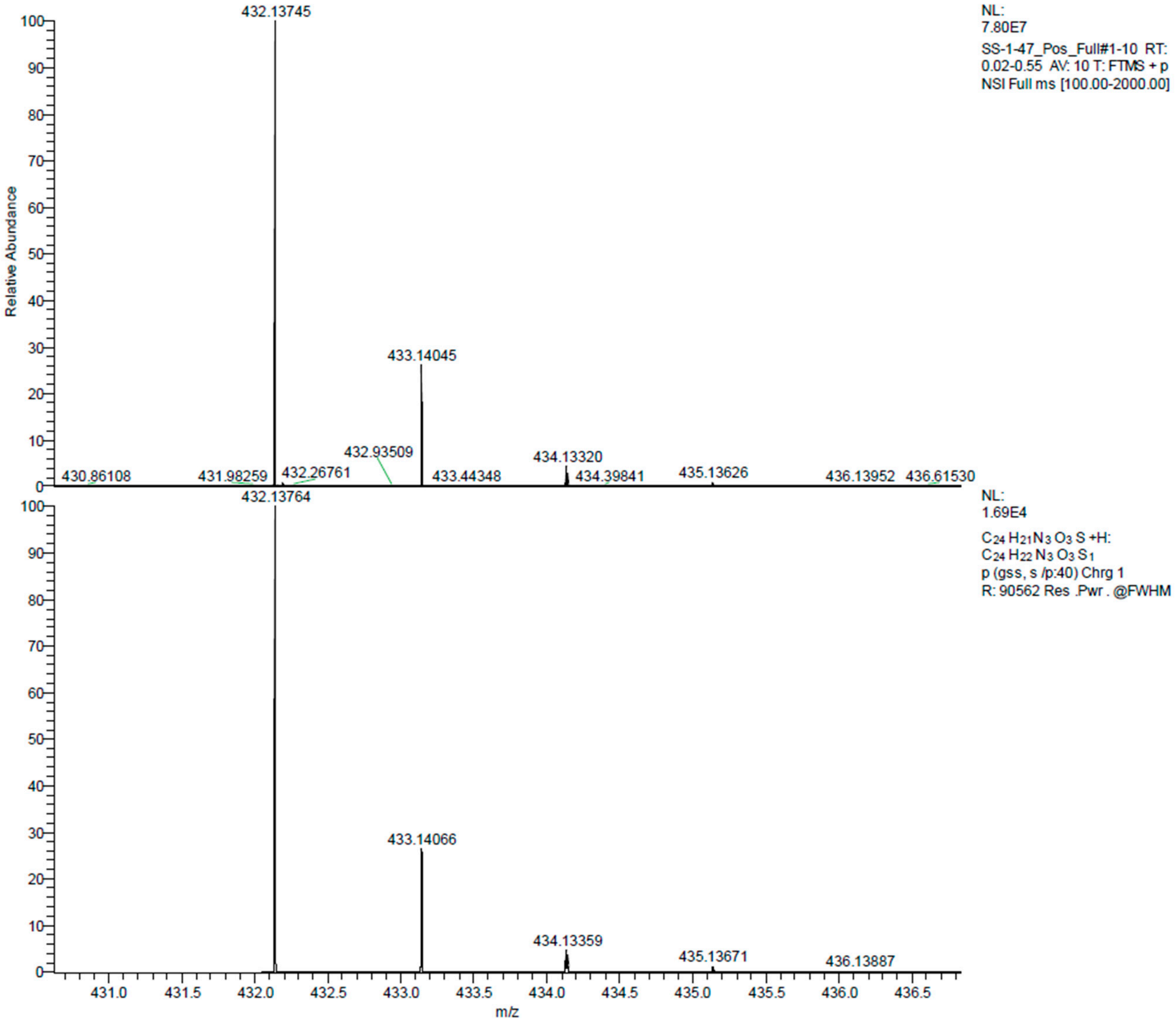

3-Cyclopropyl-2-((2-morpholino-2-oxoethyl)thio)quinazolin-4(3*H*)-one (**5g**)

Full spectrum

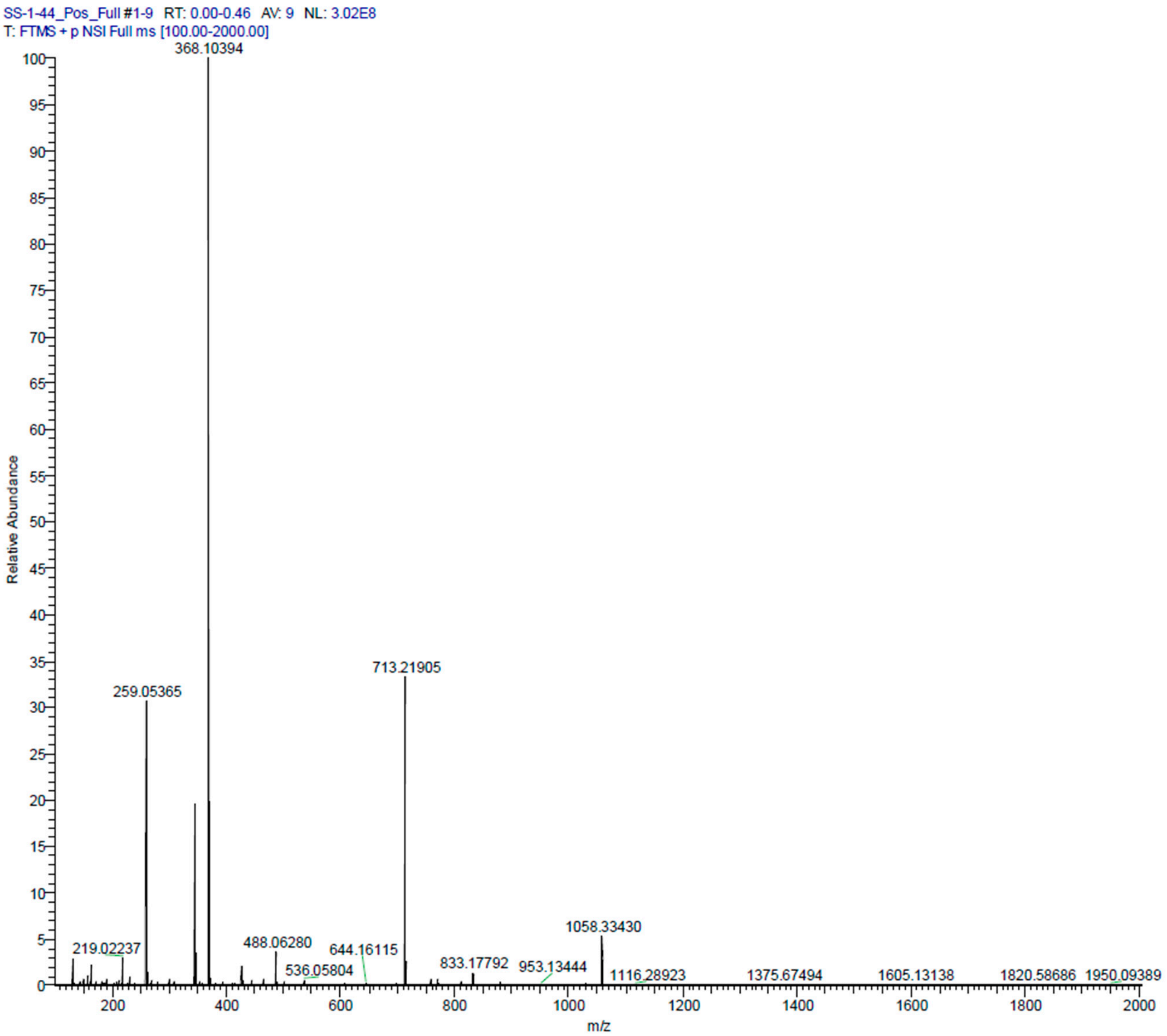

Zoomed spectrum (Top spectrum: measured mass; bottom spectrum: calculated mass)

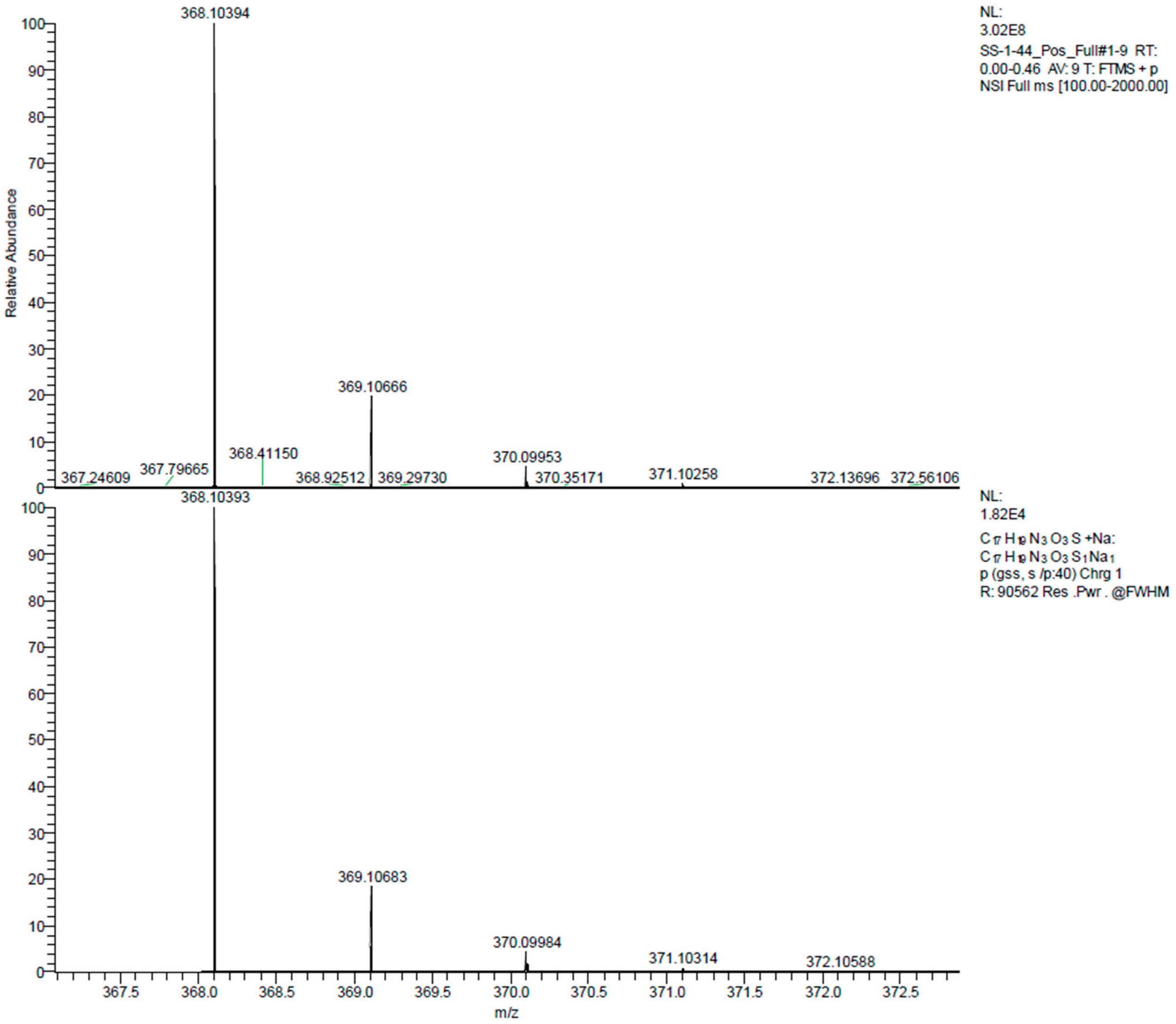

2-((2-Morpholino-2-oxoethyl)thio)-3-phenylquinazolin-4(3*H*)-one (**5h**)

Full spectrum

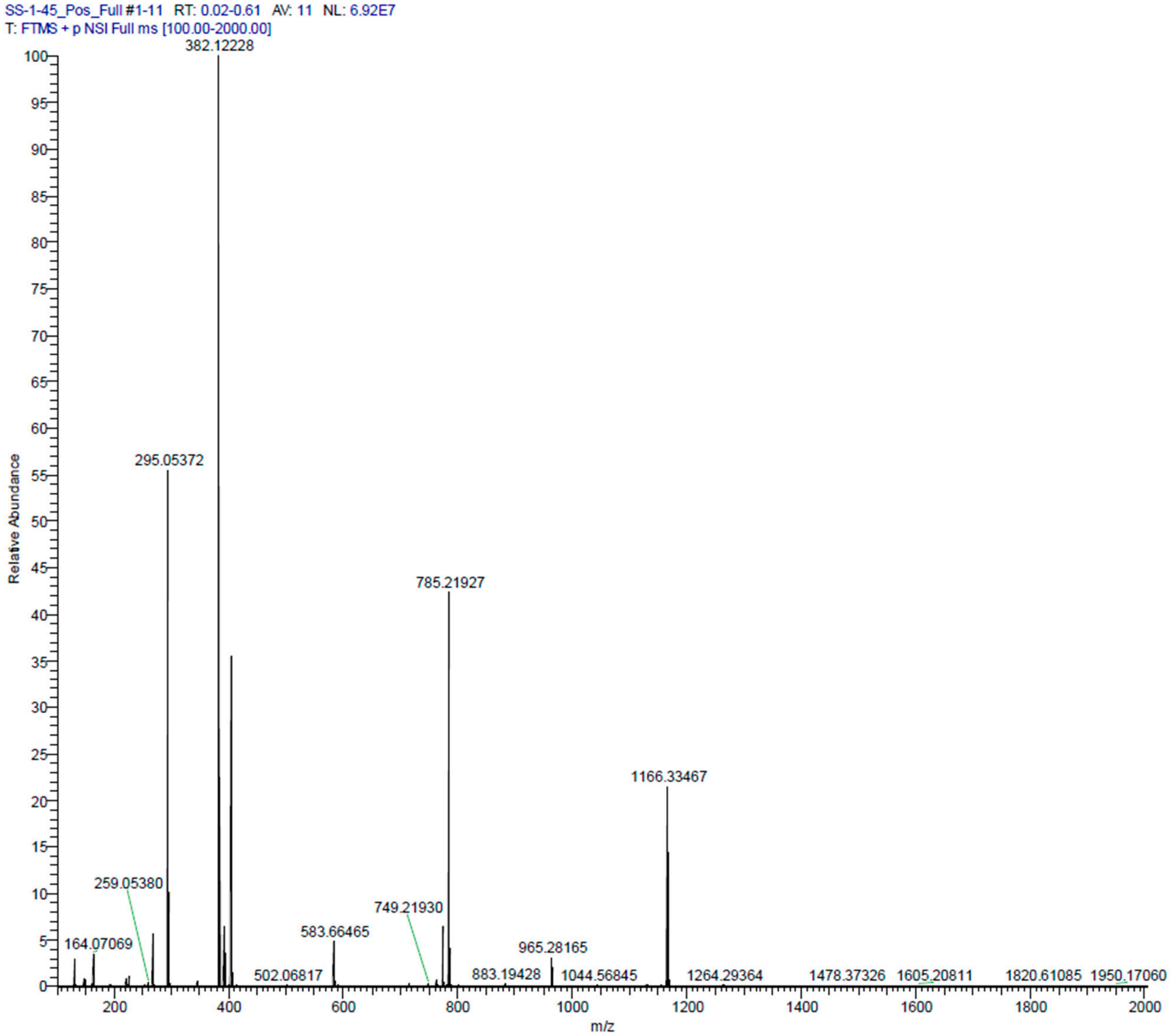

Zoomed spectrum (Top spectrum: measured mass; bottom spectrum: calculated mass)

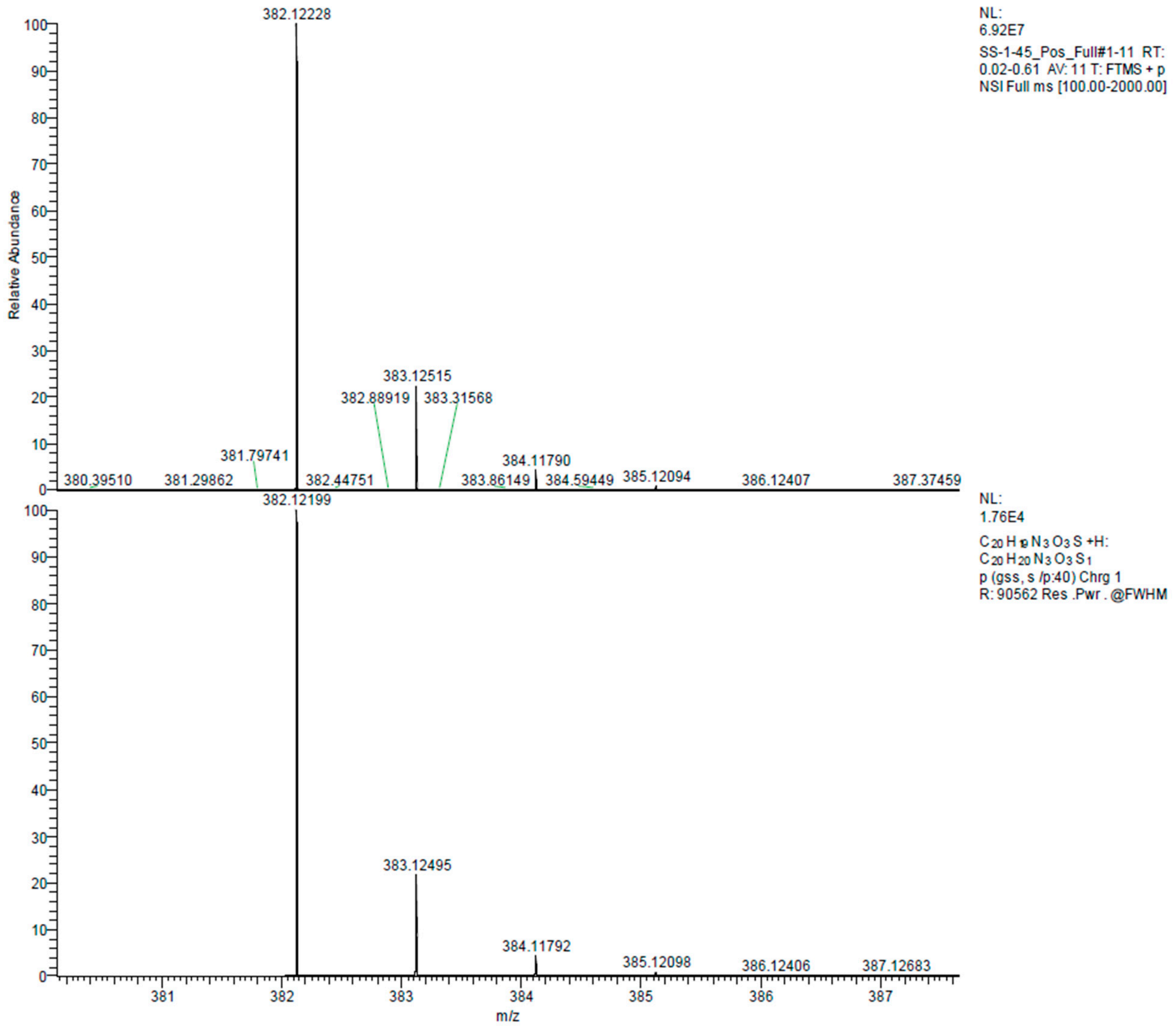

2-((2-Morpholino-2-oxoethyl)thio)quinazolin-4(3*H*)-one (**5i**)

Full spectrum

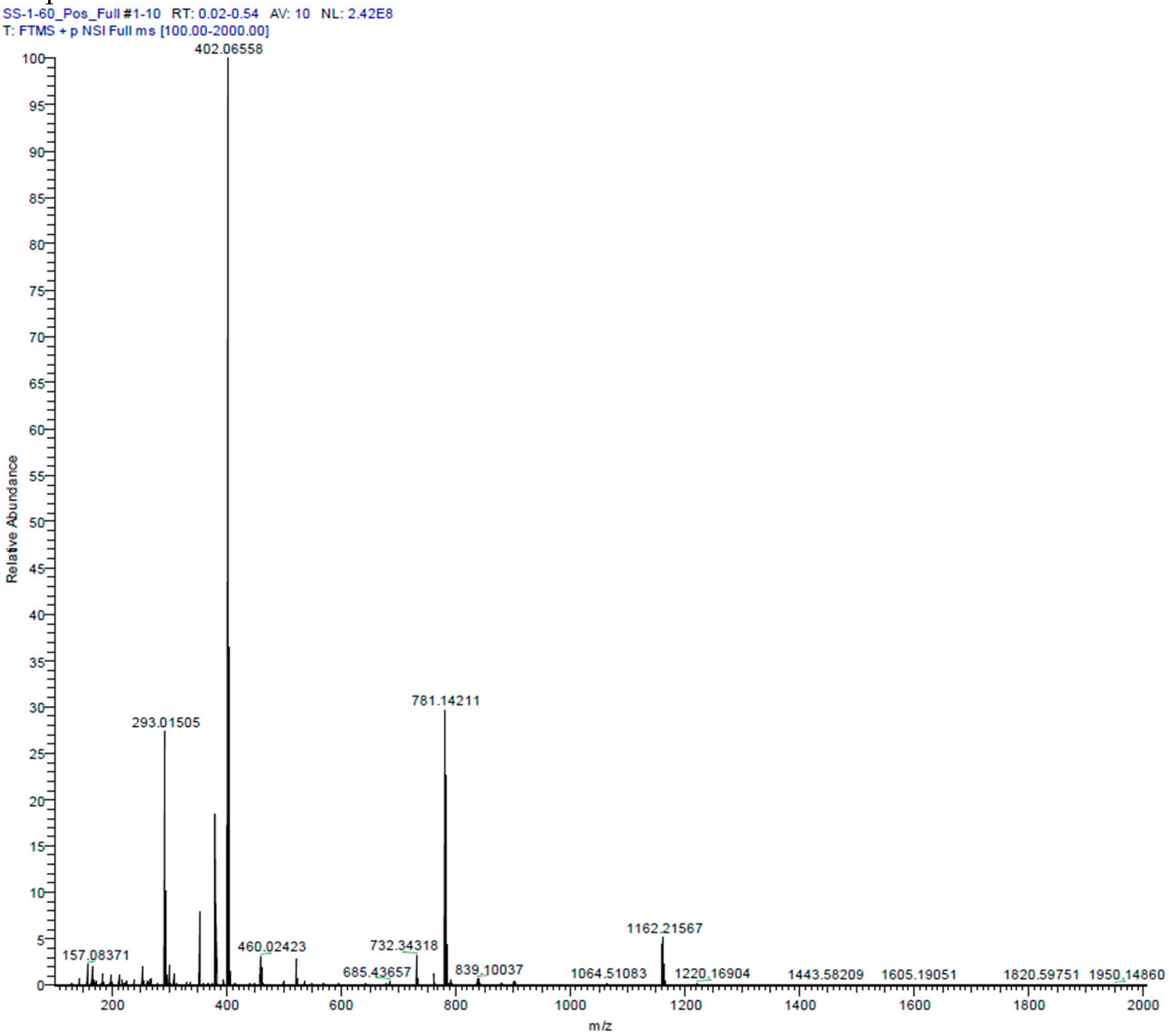

Zoomed spectrum (Top spectrum: measured mass; bottom spectrum: calculated mass)

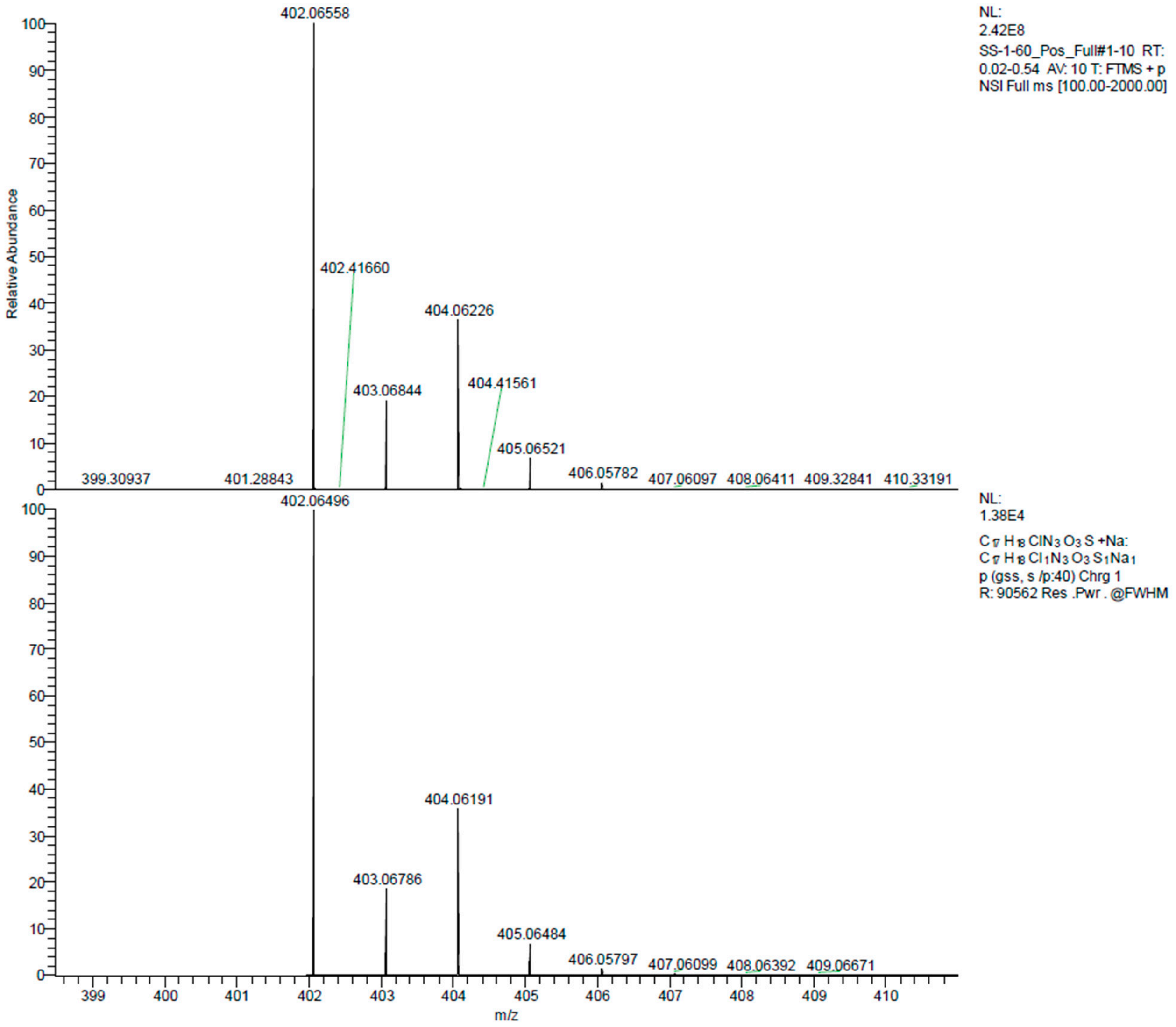

N-(4-Methoxyphenyl)-2-((4-oxo-3-phenyl-3,4-dihydroquinazolin-2-yl)thio)acetamide (**5j**)

Full spectrum

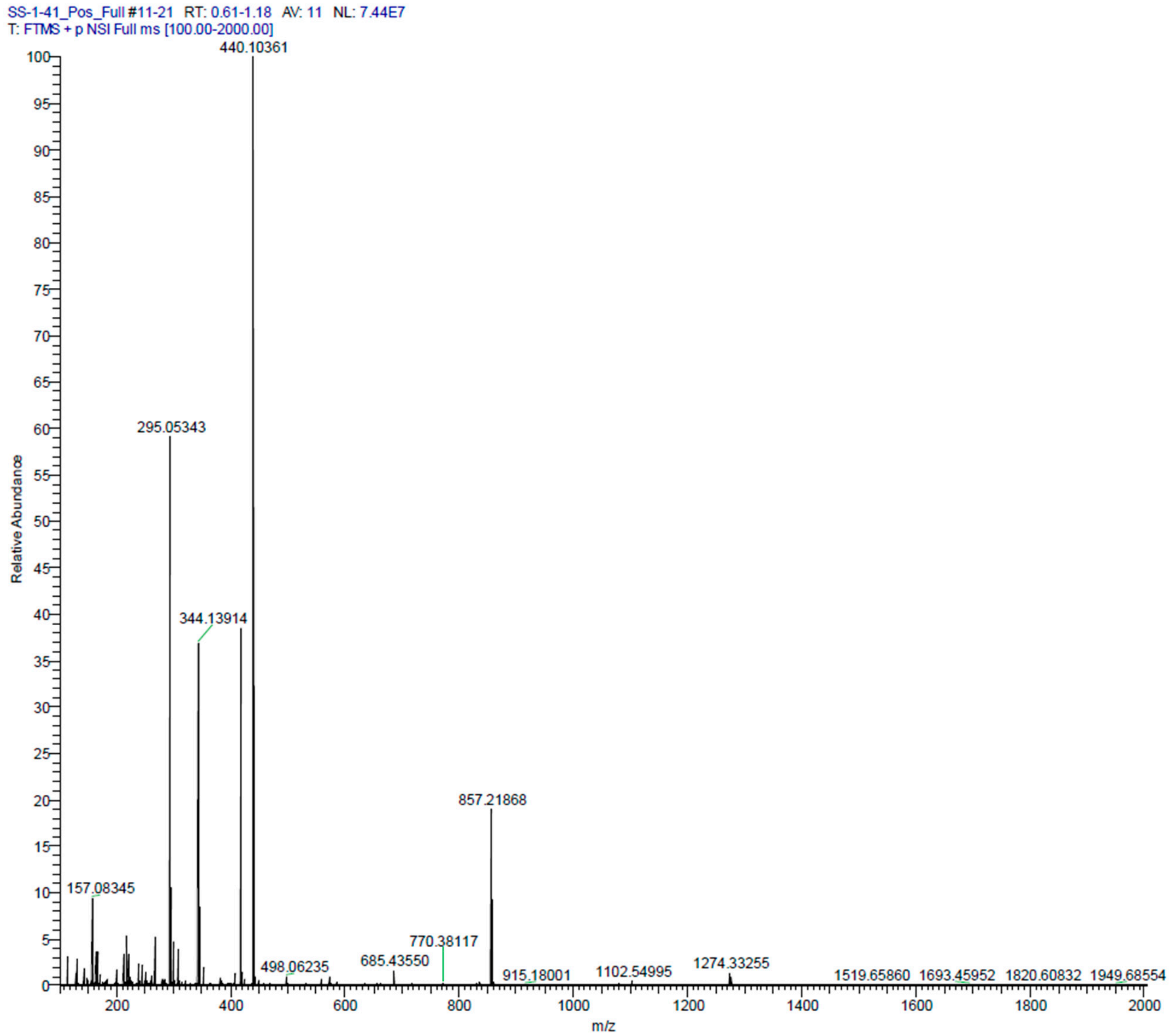

Zoomed spectrum (Top spectrum: measured mass; bottom spectrum: calculated mass)

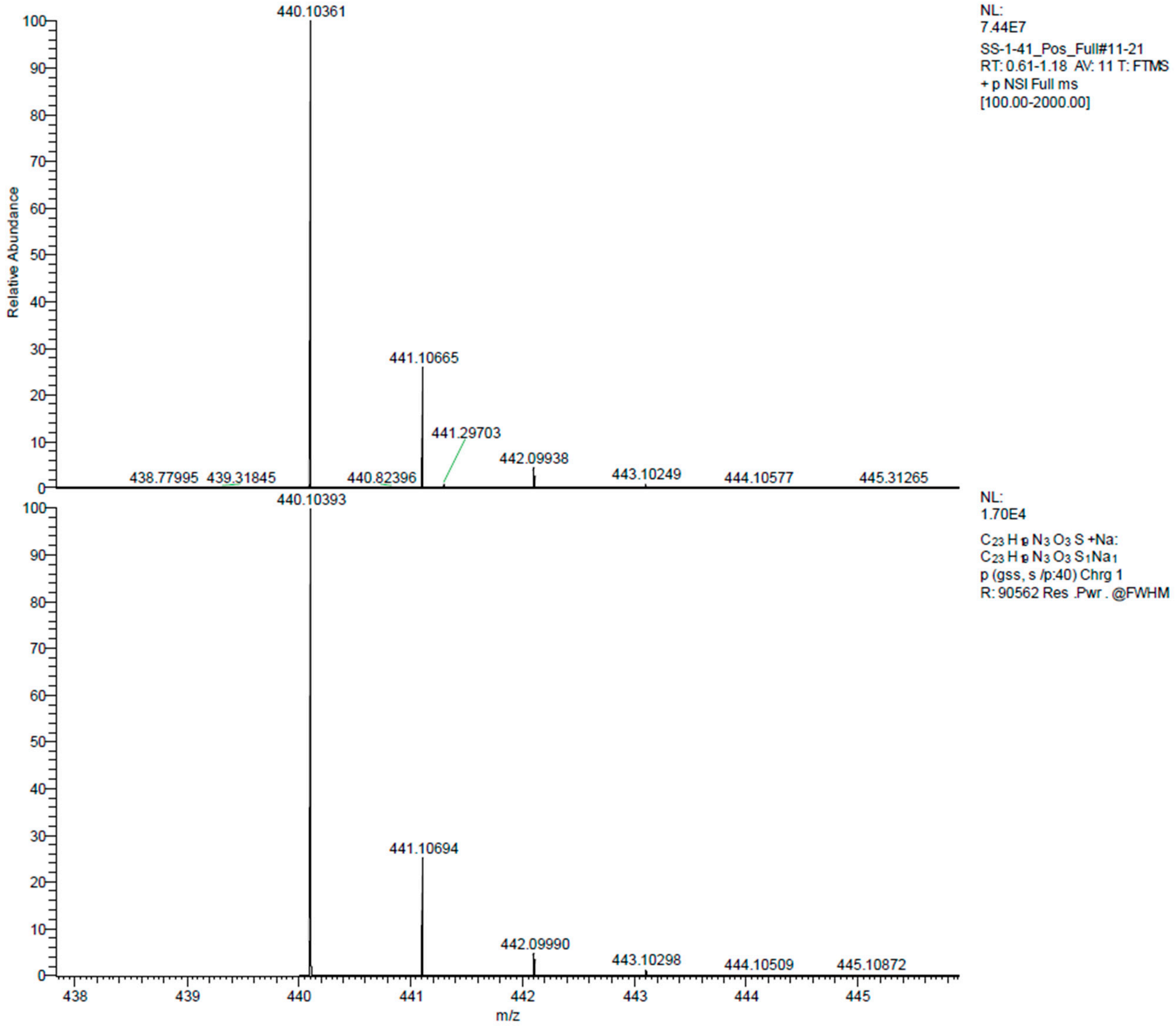

2-((6-Chloro-3-cyclopropyl-4-oxo-3,4-dihydroquinazolin-2-yl)thio)-*N*-(4-methoxyphenyl)acetamide (**5k**)

Full spectrum

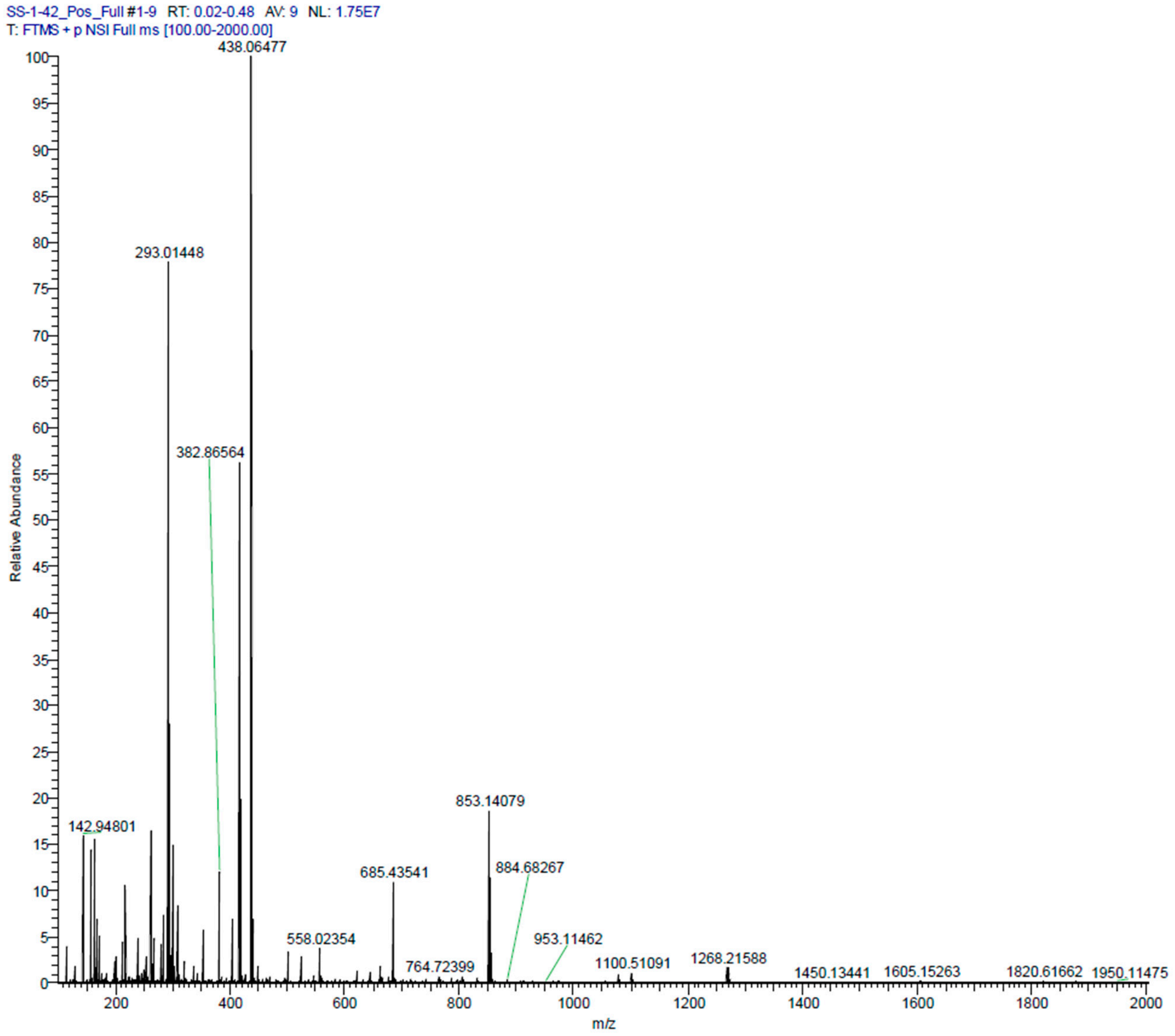

Zoomed spectrum (Top spectrum: measured mass; bottom spectrum: calculated mass)

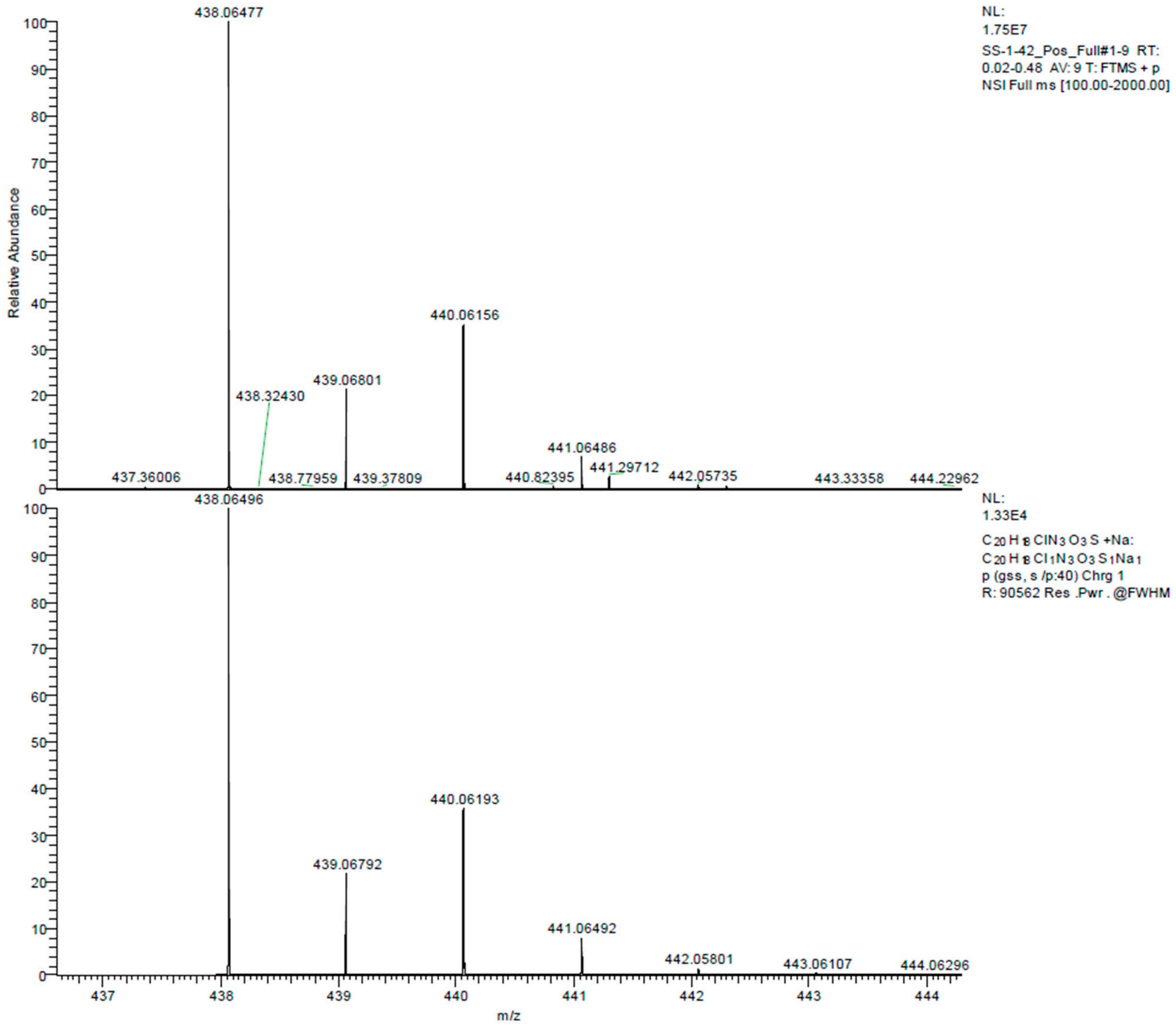

2-(4-(((3-Cyclopropyl-4-oxo-3,4-dihydroquinazolin-2-yl)thio)methyl)-1*H*-1,2,3-triazol-1-yl)-*N*-phenylacetamide (**6a**)

Full spectrum

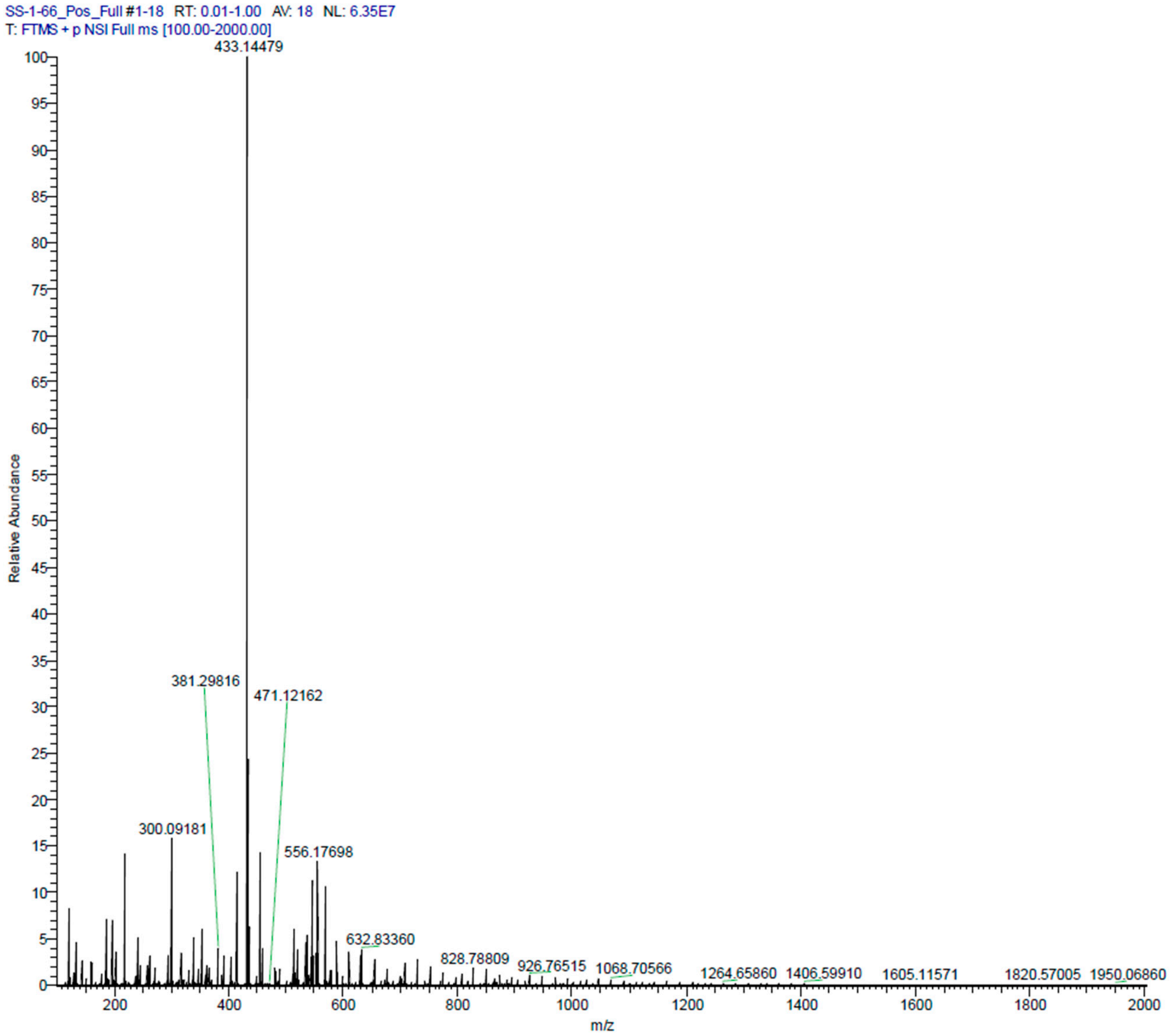

Zoomed spectrum (Top spectrum: measured mass; bottom spectrum: calculated mass)

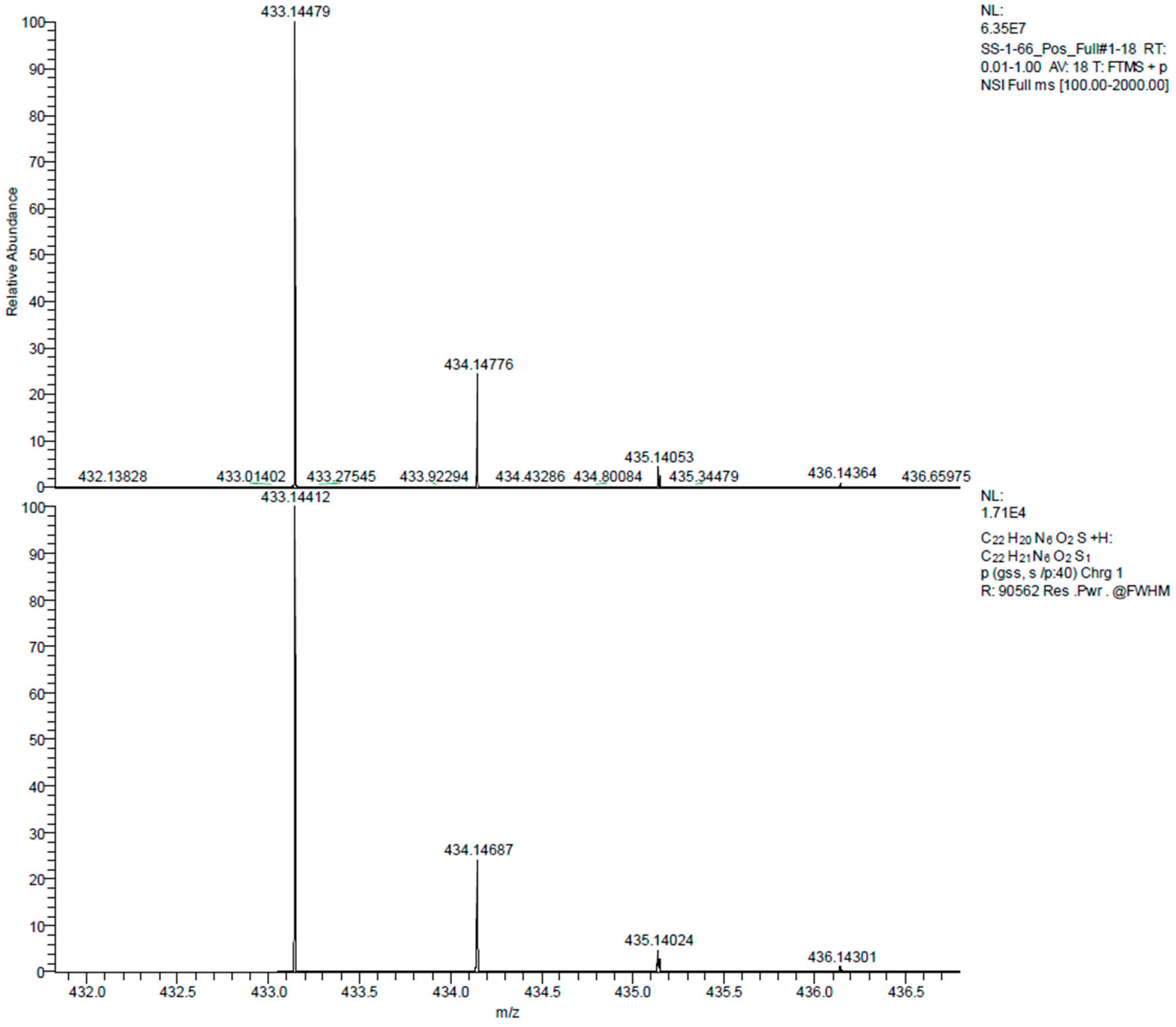

2-(4-(((3-Cyclopropyl-4-oxo-3,4-dihydroquinazolin-2-yl)thio)methyl)-1*H*-1,2,3-triazol-1-yl)-*N*-(4-methoxyphenyl)acetamide (**6b**)

Full spectrum

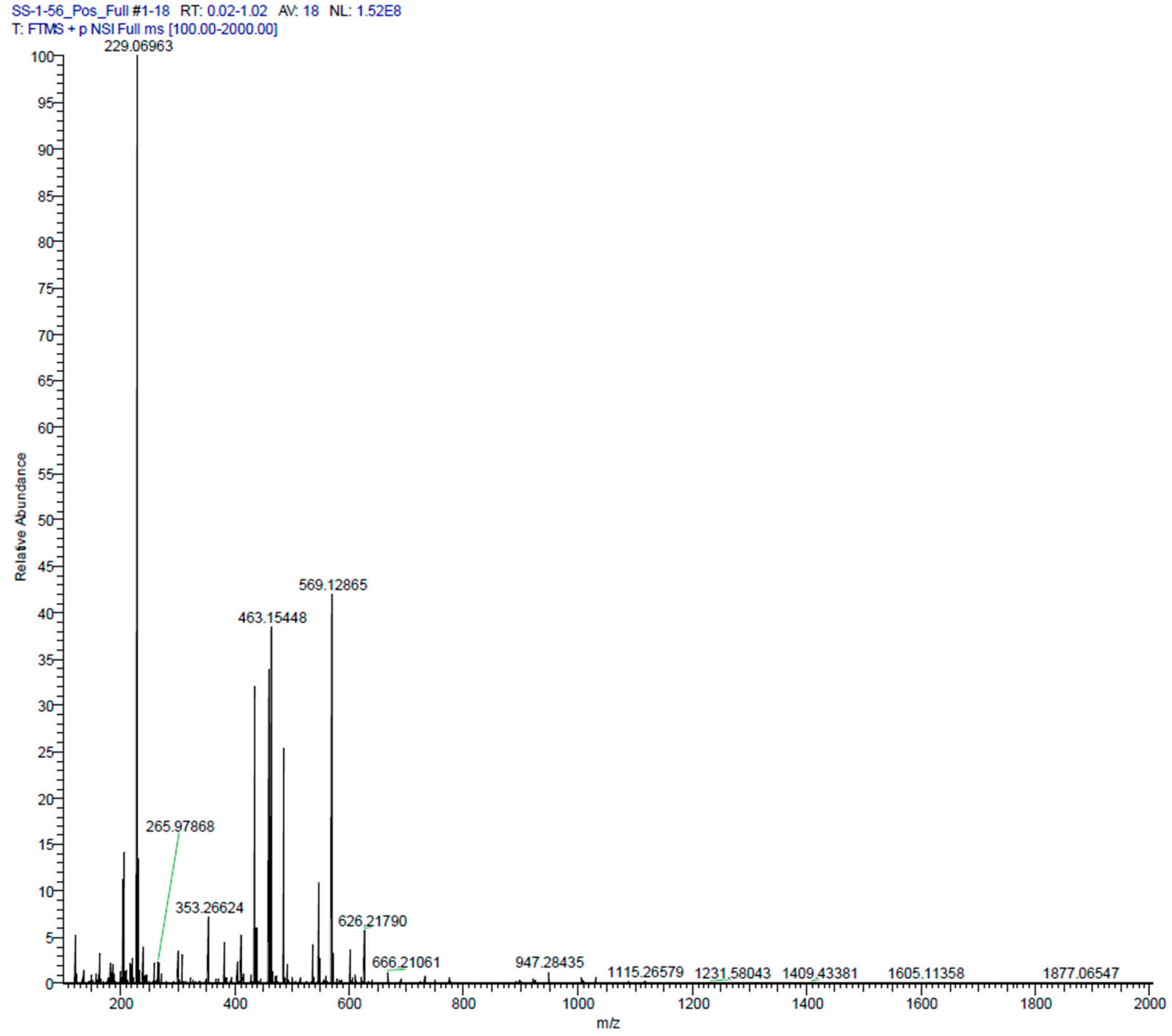

Zoomed spectrum (Top spectrum: measured mass; bottom spectrum: calculated mass)

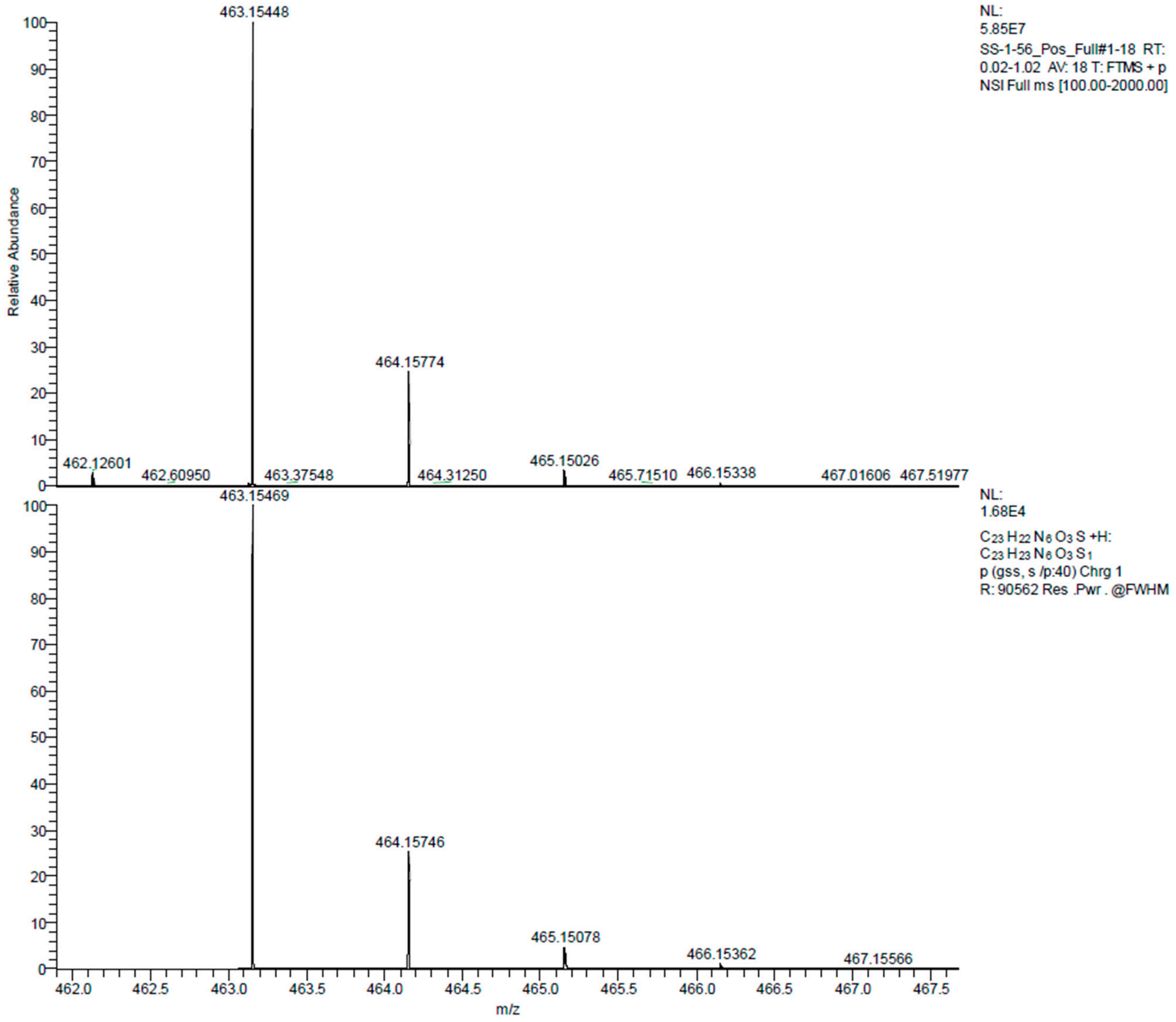

2-(4-(((6-Chloro-3-cyclopropyl-4-oxo-3,4-dihydroquinazolin-2-yl)thio)methyl)-1*H*-1,2,3-triazol-1-yl)-*N*-(4-methoxyphenyl)acetamide (**6c**)

Full spectrum

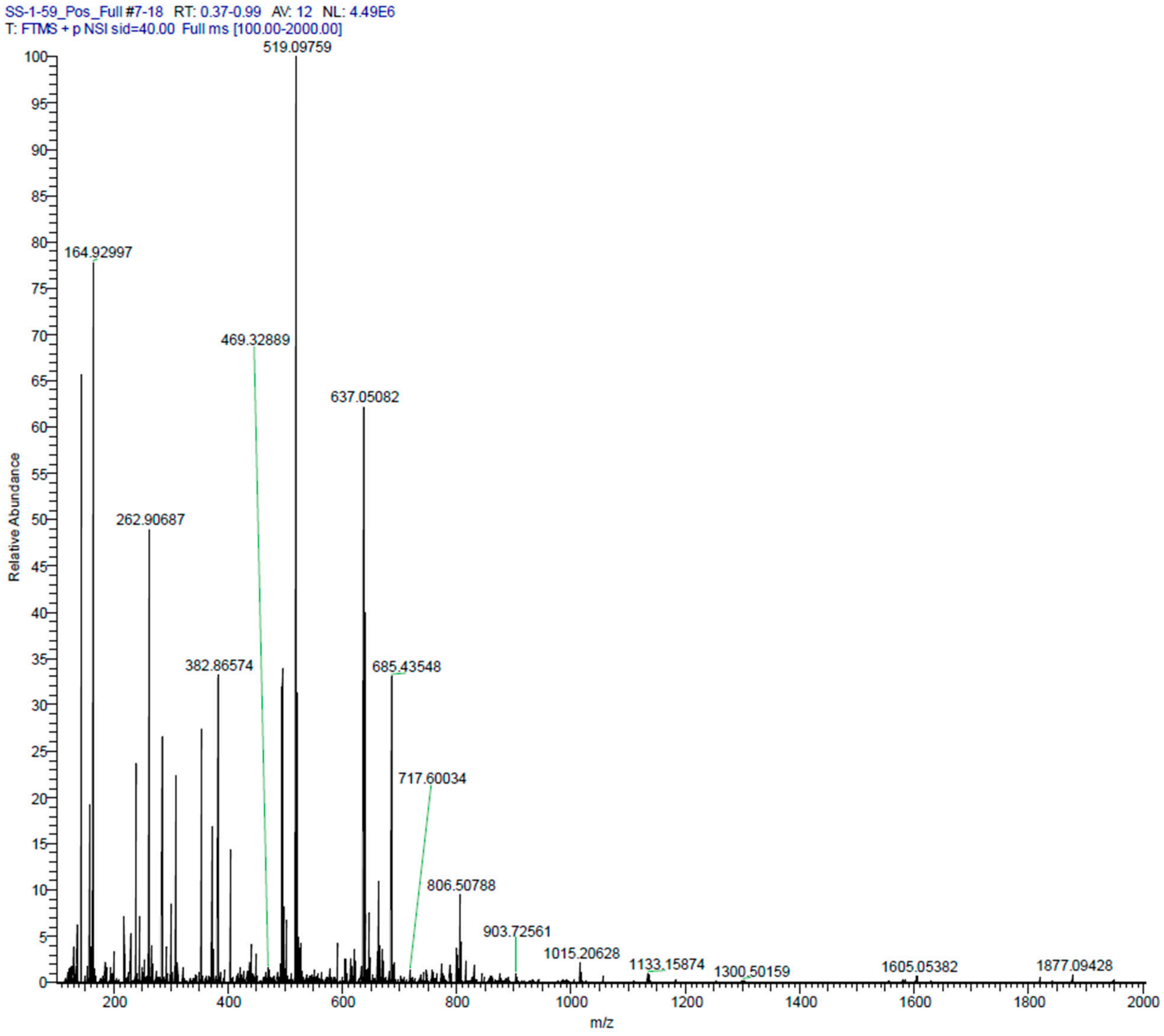

Zoomed spectrum (Top spectrum: measured mass; bottom spectrum: calculated mass)

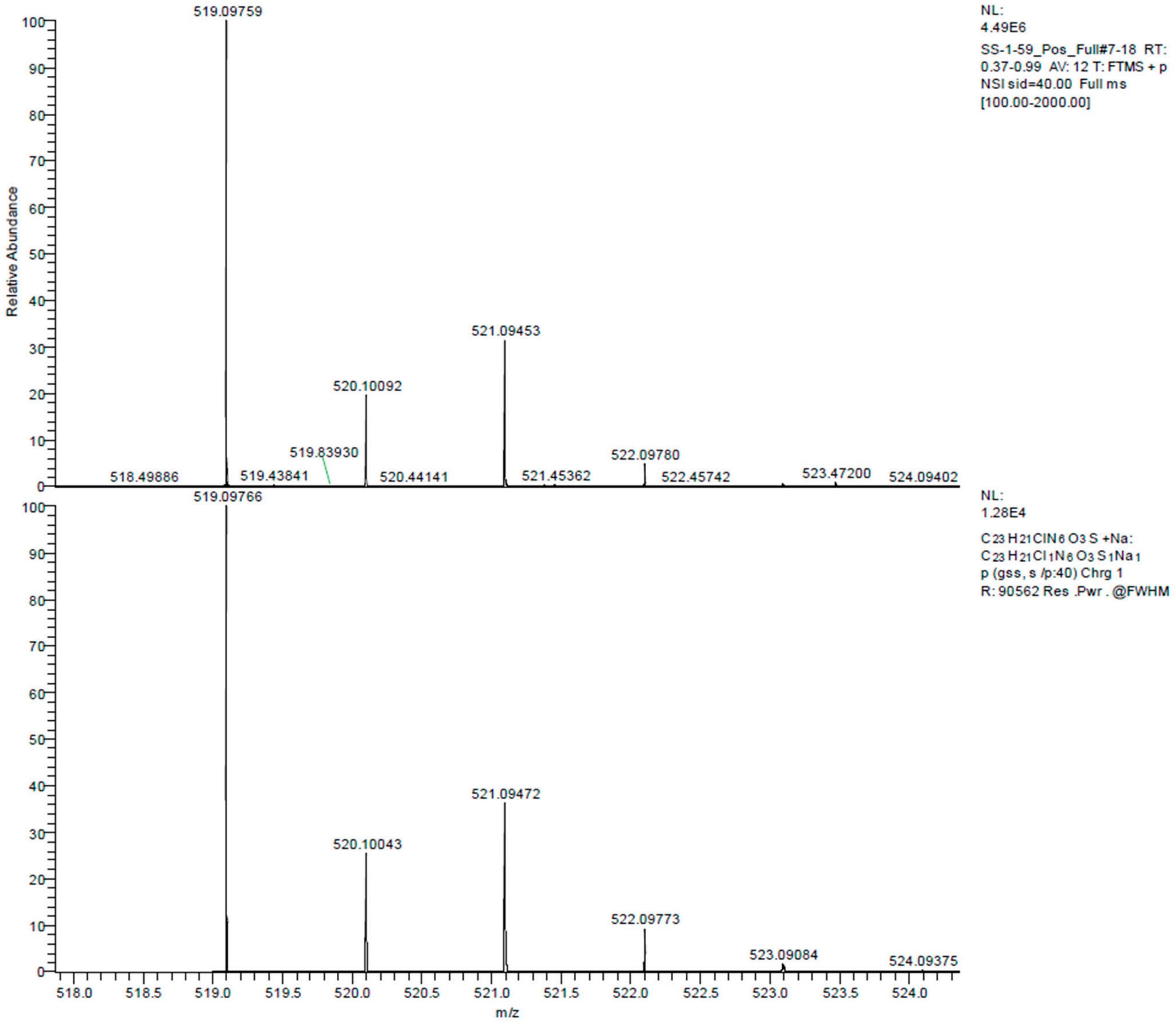

N-(4-Methoxyphenyl)-2-(4-(((4-oxo-3-phenyl-3,4-dihydroquinazolin-2-yl)thio)methyl)-1*H*-1,2,3-triazol-1-yl)acetamide (**6d**)

Full spectrum

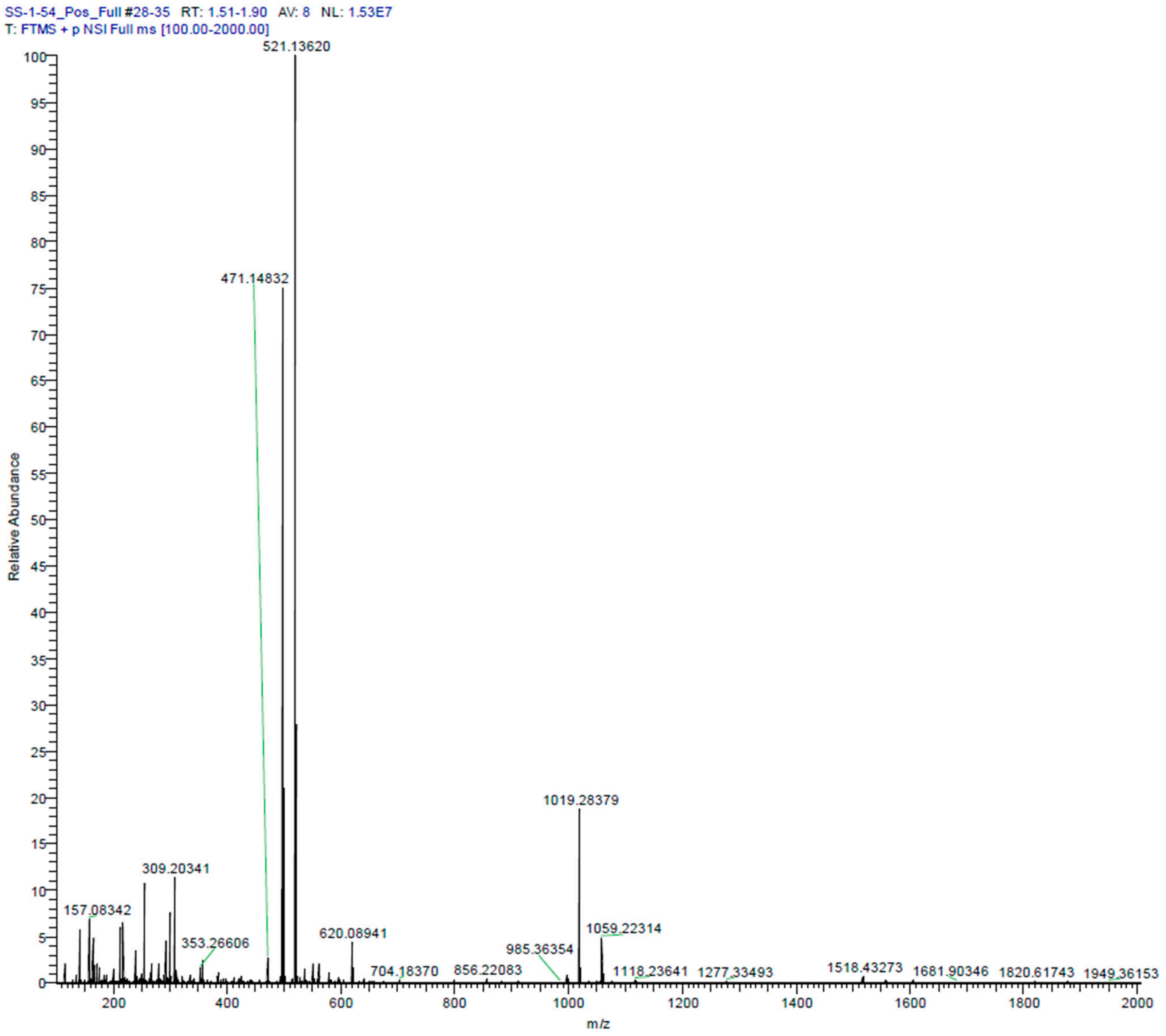

Zoomed spectrum (Top spectrum: measured mass; bottom spectrum: calculated mass)

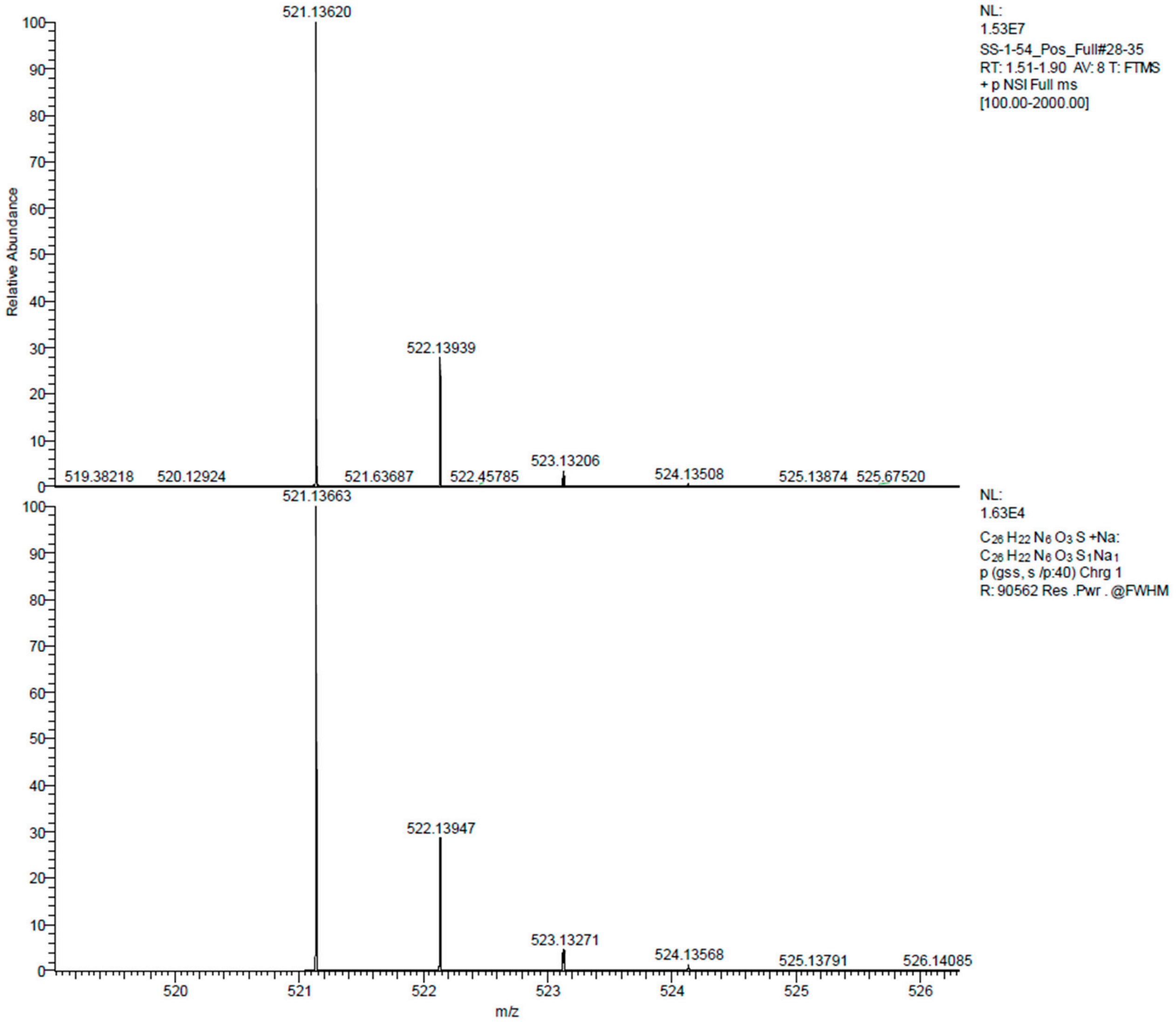

2-(4-(((3-Cyclopropyl-4-oxo-3,4-dihydroquinazolin-2-yl)thio)methyl)-1*H*-1,2,3-triazol-1-yl)-*N*-(4-nitrophenyl)acetamide (**6e**)

Full spectrum

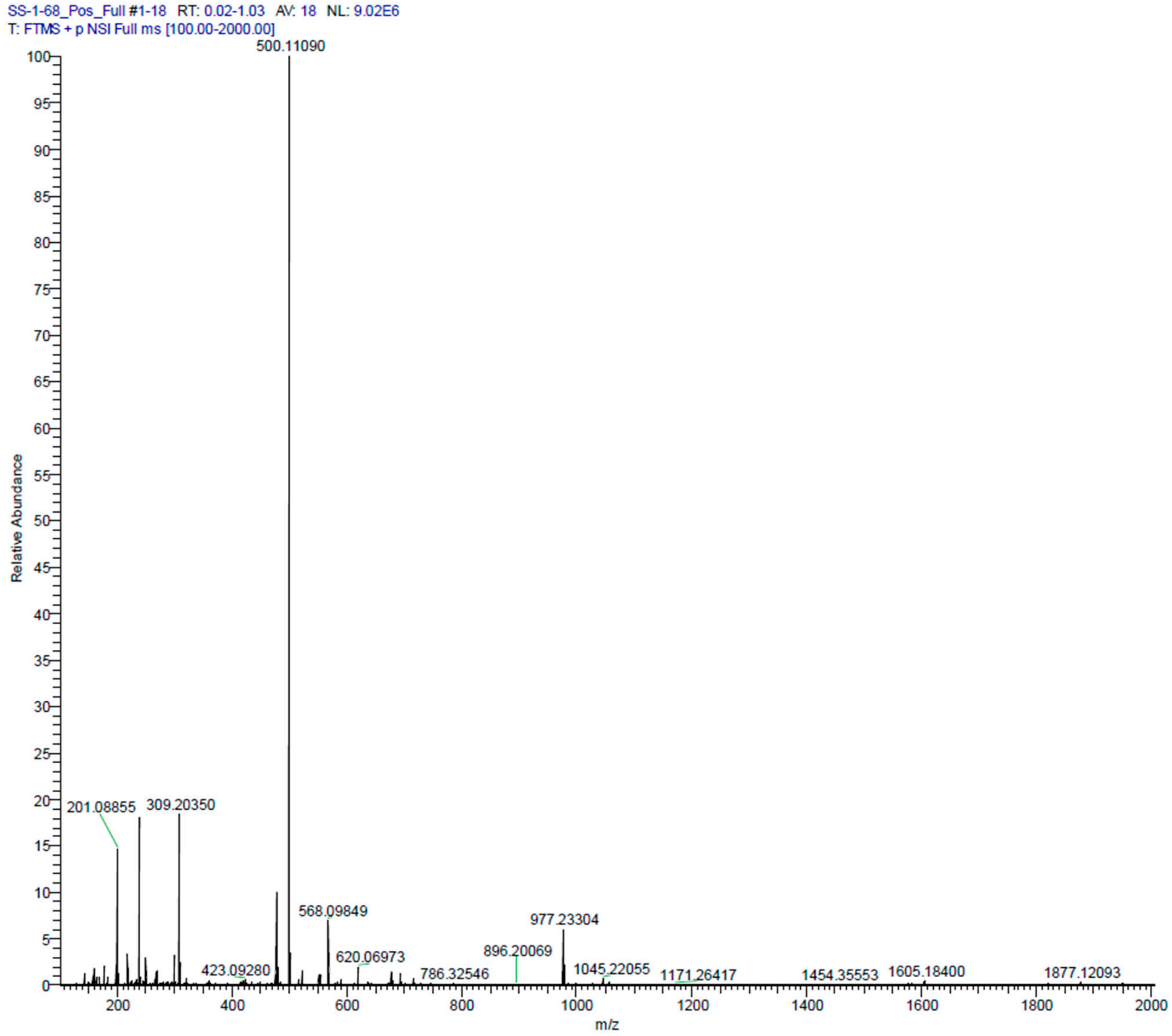

Zoomed spectrum (Top spectrum: measured mass; bottom spectrum: calculated mass)

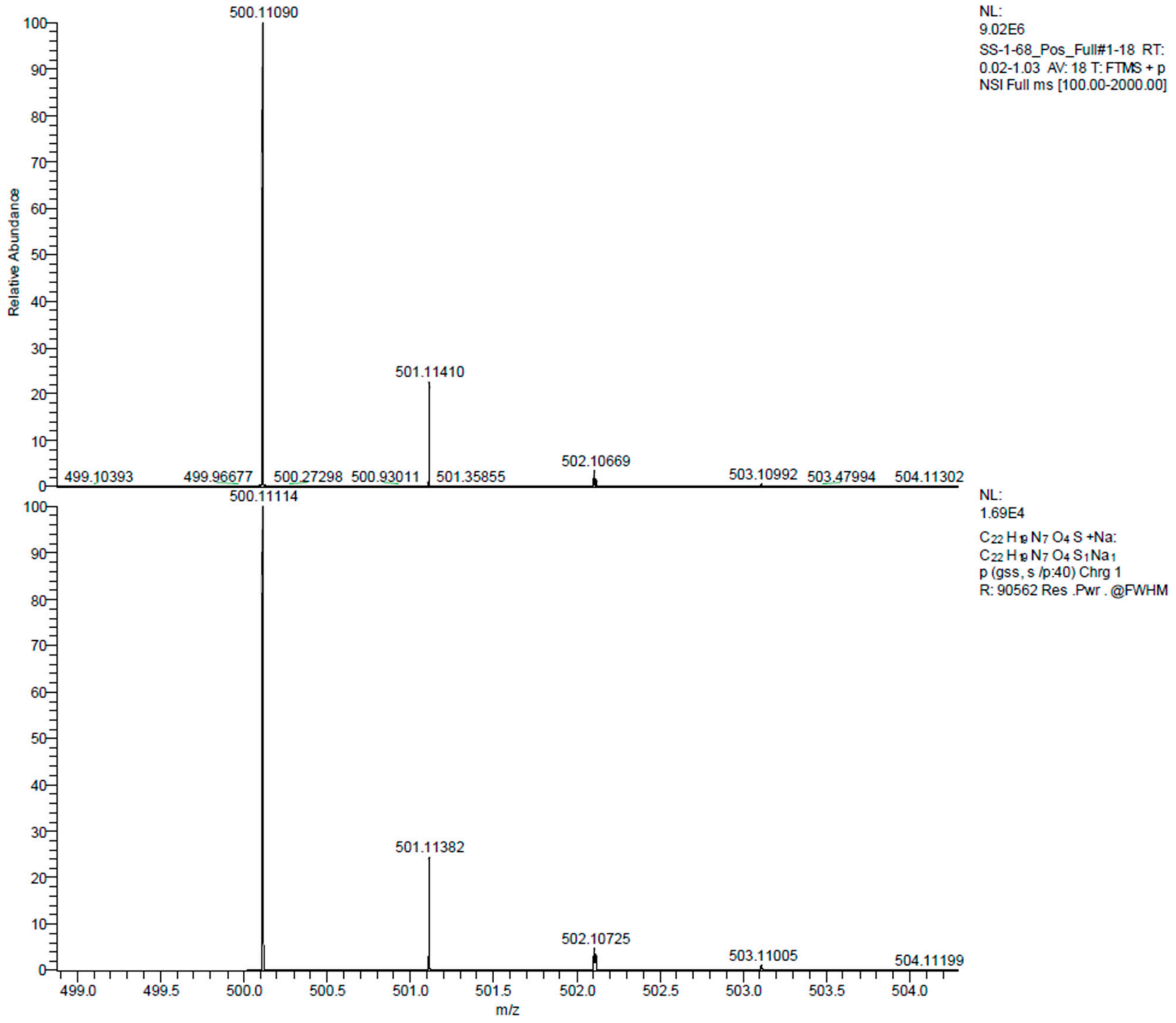

3-Cyclopropyl-2-mercaptoquinazolin-4(3*H*)-one (**8a**)

Full spectrum

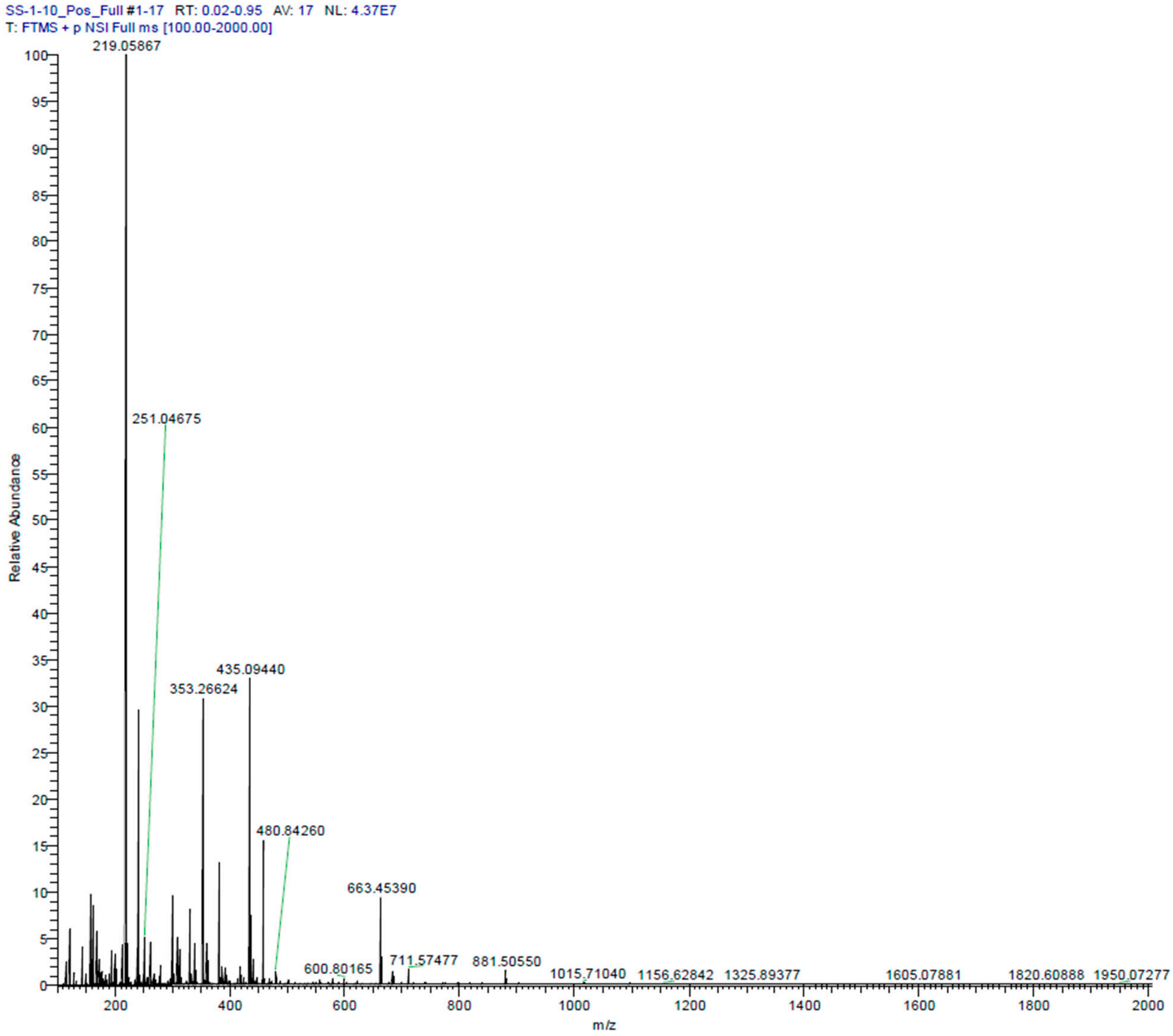

Zoomed spectrum (Top spectrum: measured mass; bottom spectrum: calculated mass)

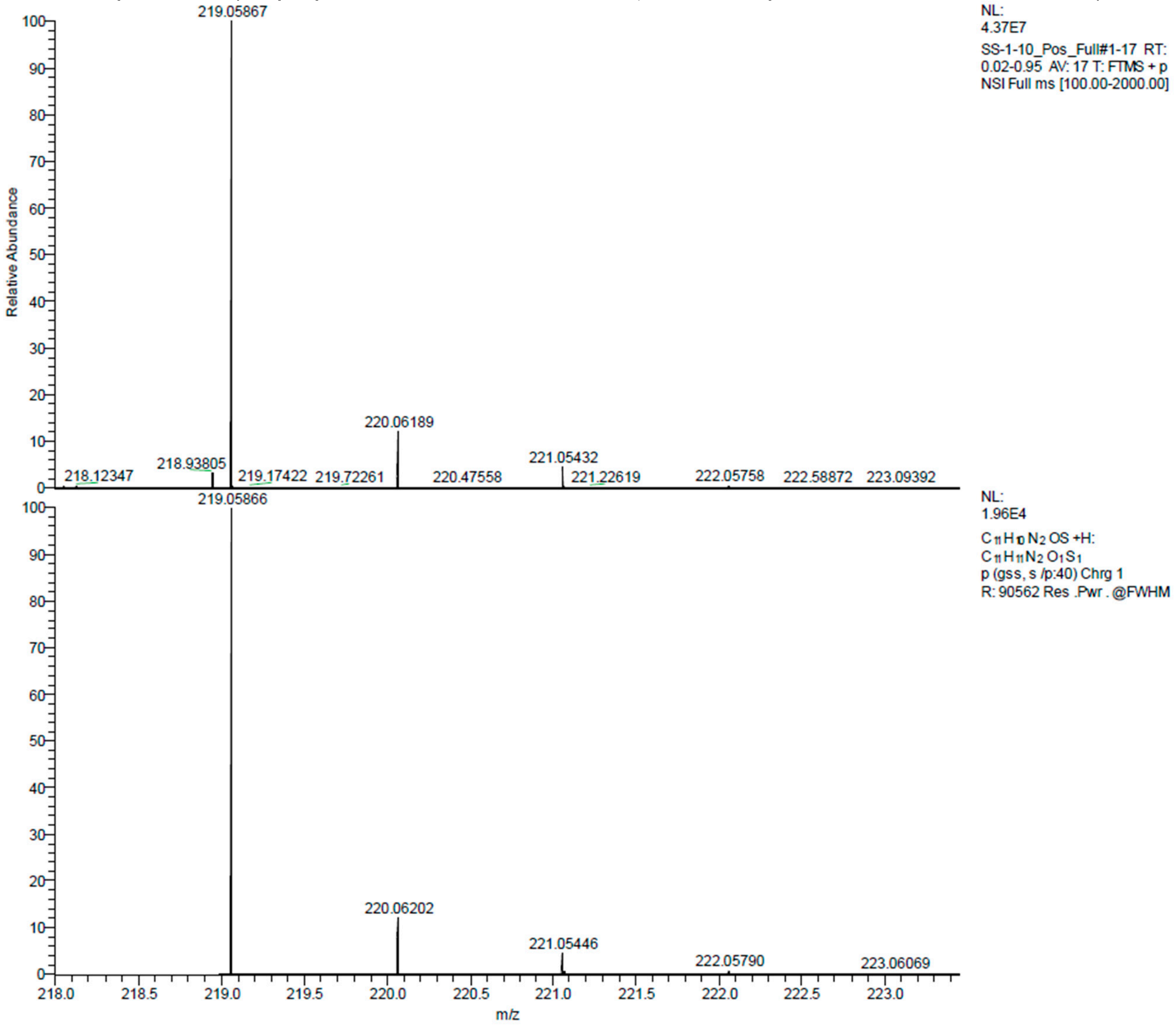

2-Mercapto-3-phenylquinazolin-4(3*H*)-one (**8b**)

Full spectrum

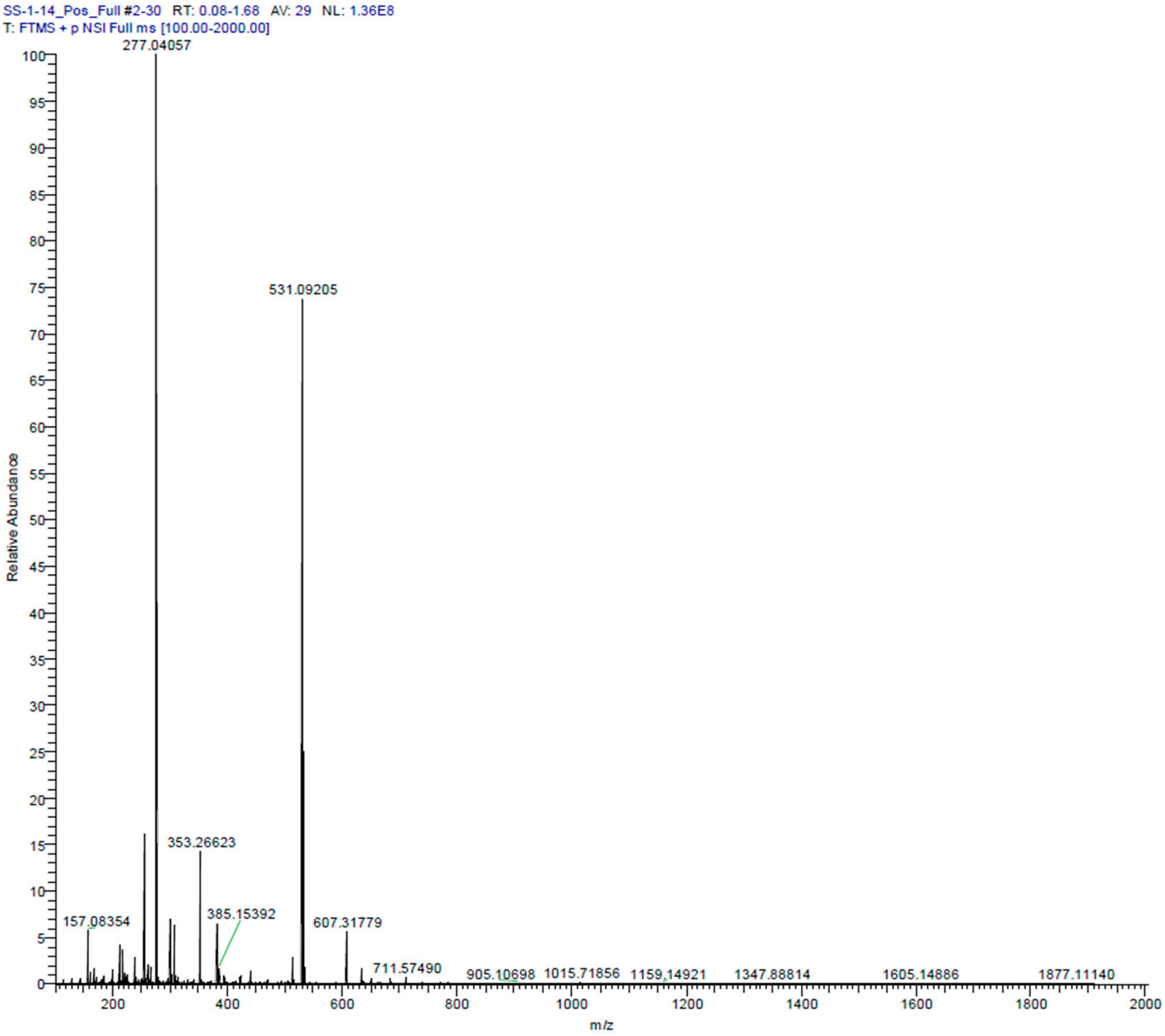

6-Chloro-3-cyclopropyl-2-mercaptoquinazolin-4(3*H*)-one (**8c**)

Full spectrum

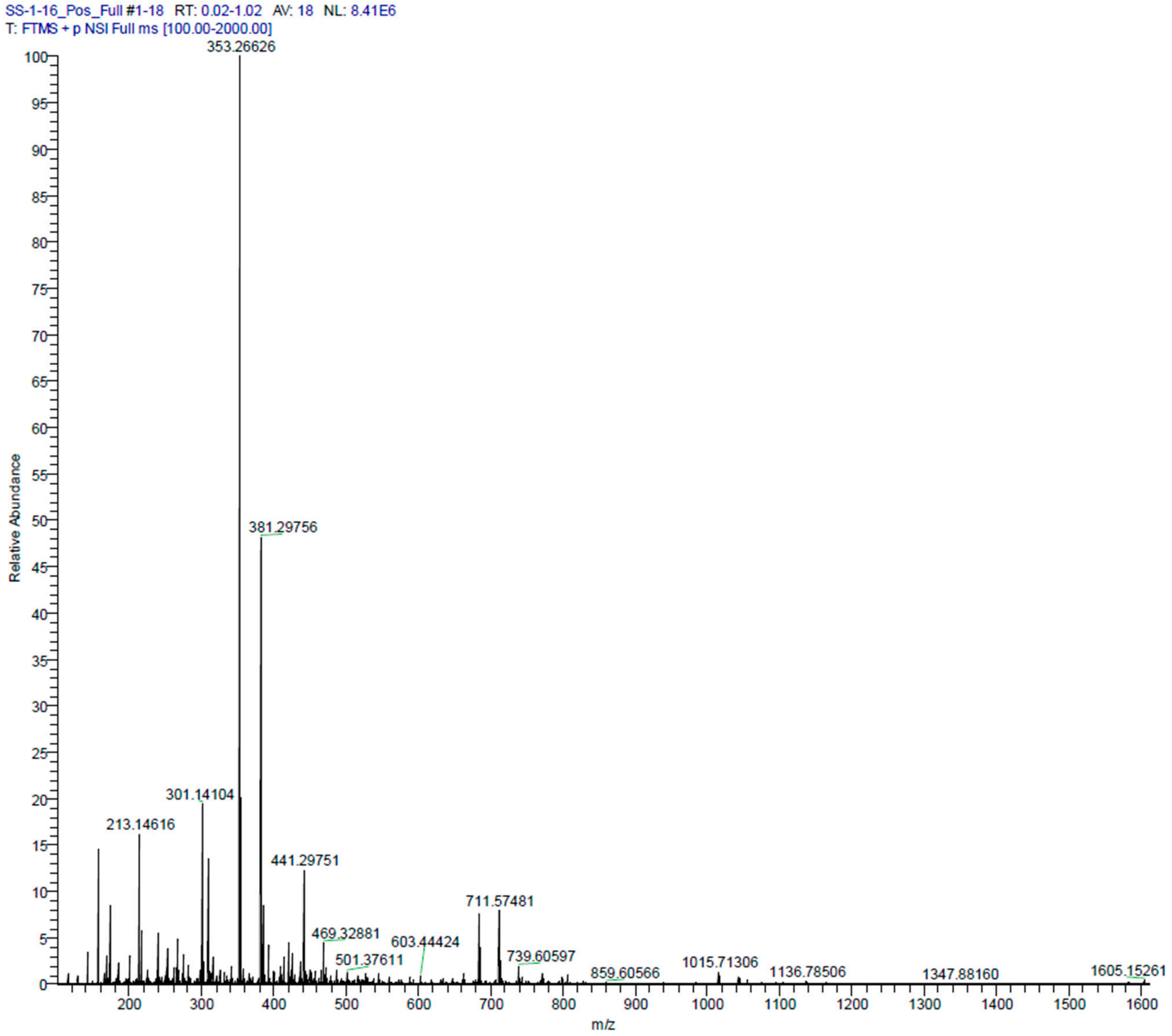

Zoomed spectrum (Top spectrum: measured mass; bottom spectrum: calculated mass)

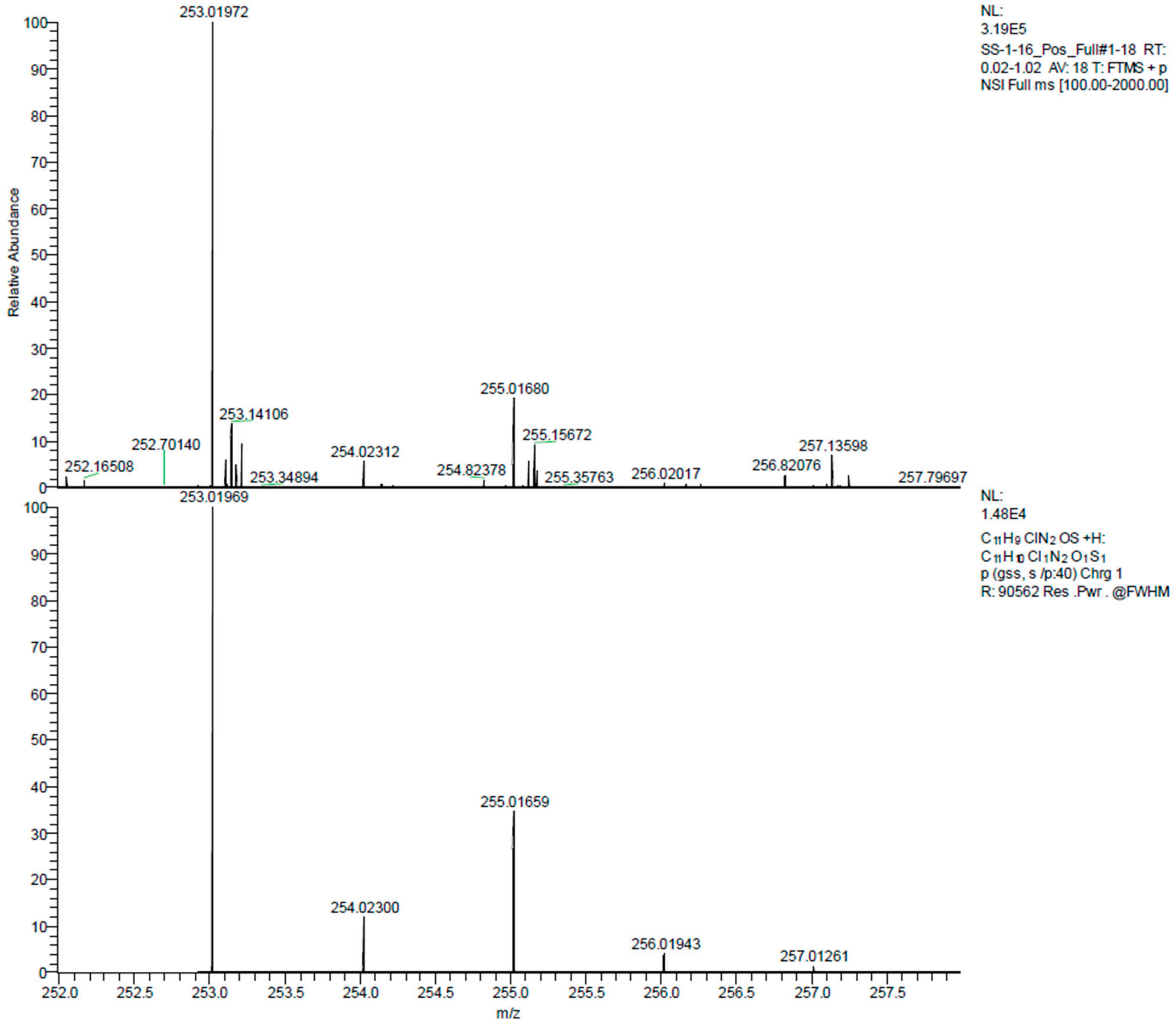

2-((3-Cyclopropyl-4-oxo-3,4-dihydroquinazolin-2-yl)thio)acetic acid (**9a**)

Full spectrum

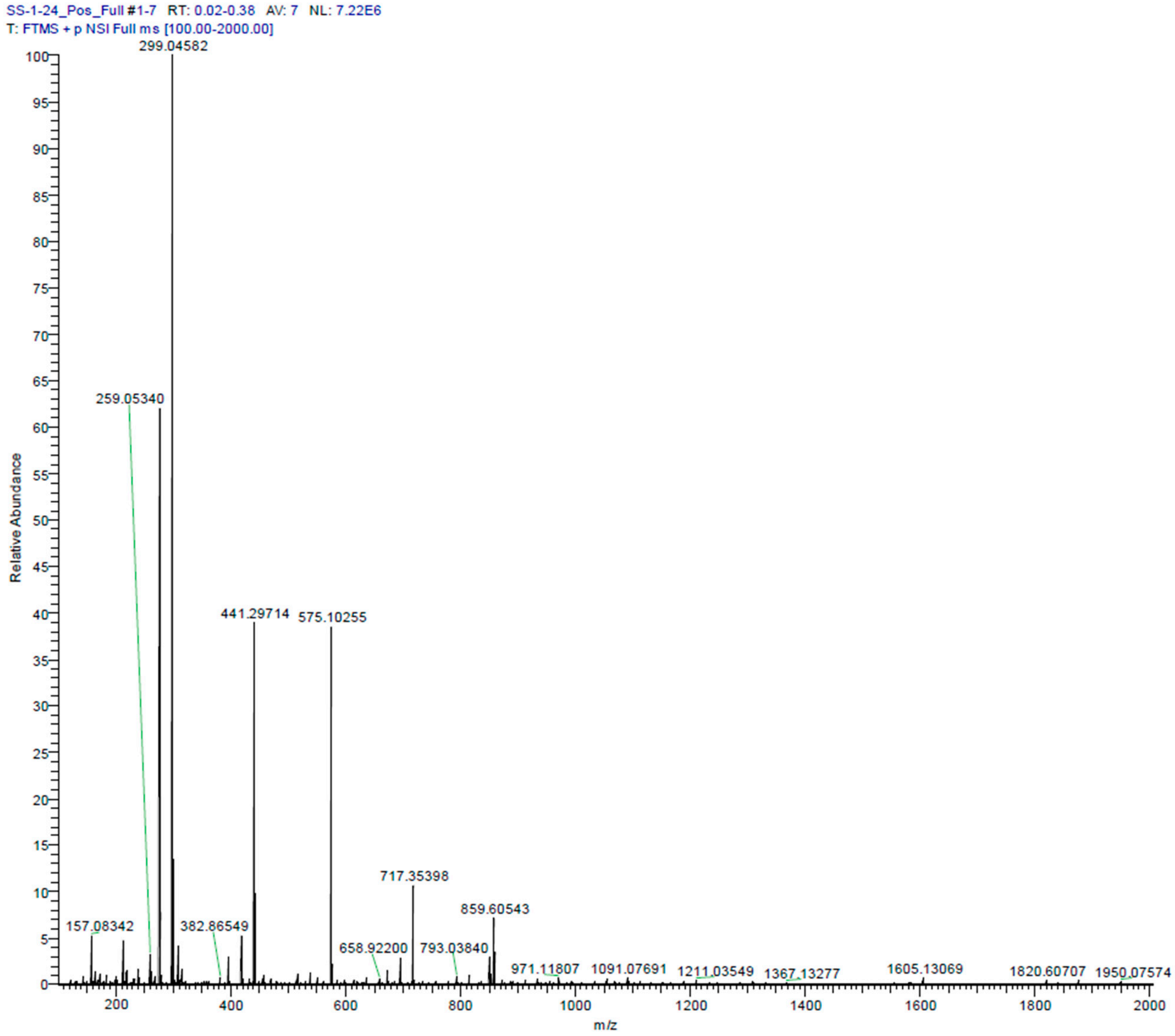

Zoomed spectrum (Top spectrum: measured mass; bottom spectrum: calculated mass)

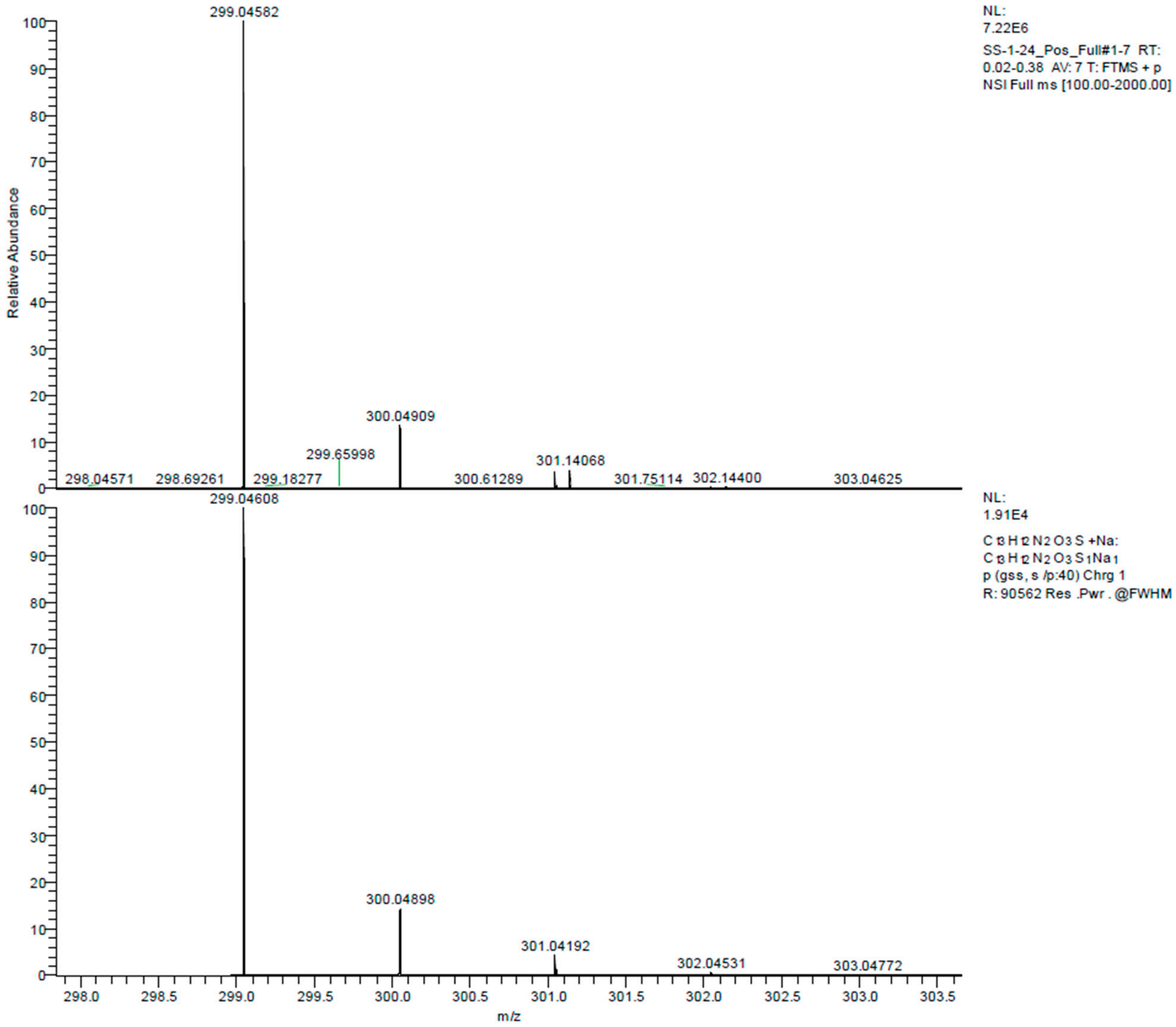

2-((4-Oxo-3-phenyl-3,4-dihydroquinazolin-2-yl)thio)acetic acid (**9b**)

Full spectrum

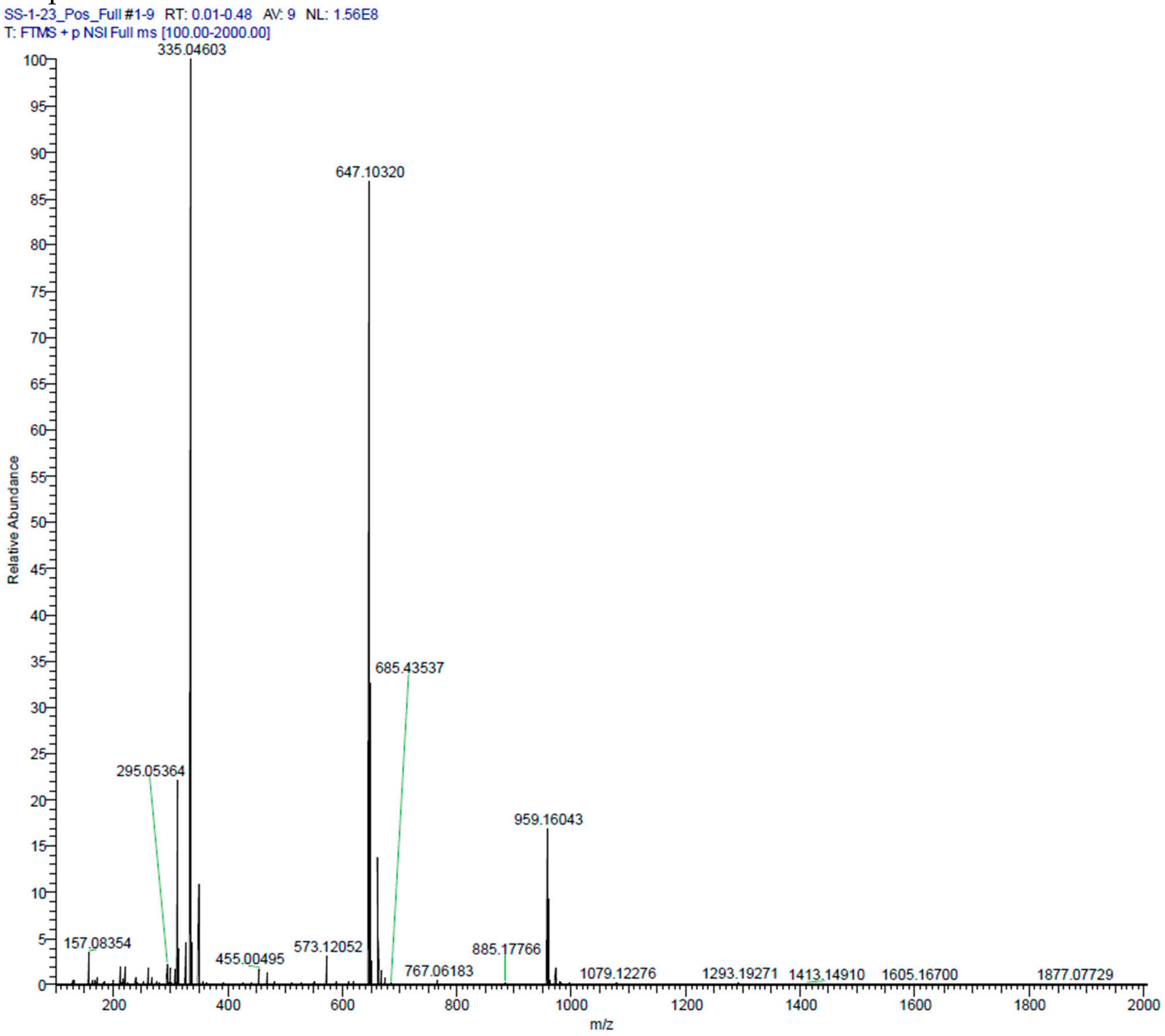

Zoomed spectrum (Top spectrum: measured mass; bottom spectrum: calculated mass)

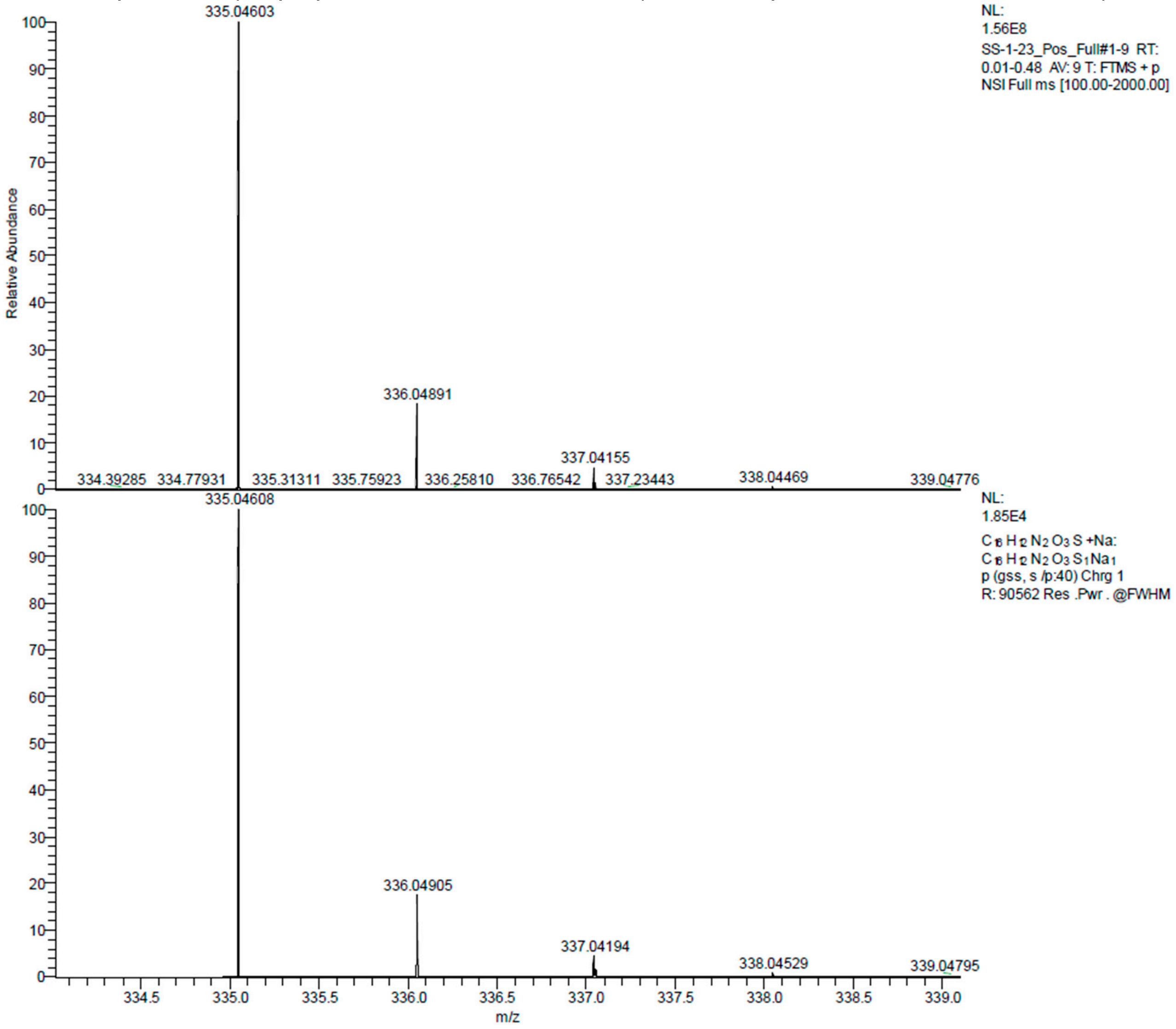

2-((6-Chloro-3-cyclopropyl-4-oxo-3,4-dihydroquinazolin-2-yl)thio)acetic acid (**9c**)

Full spectrum

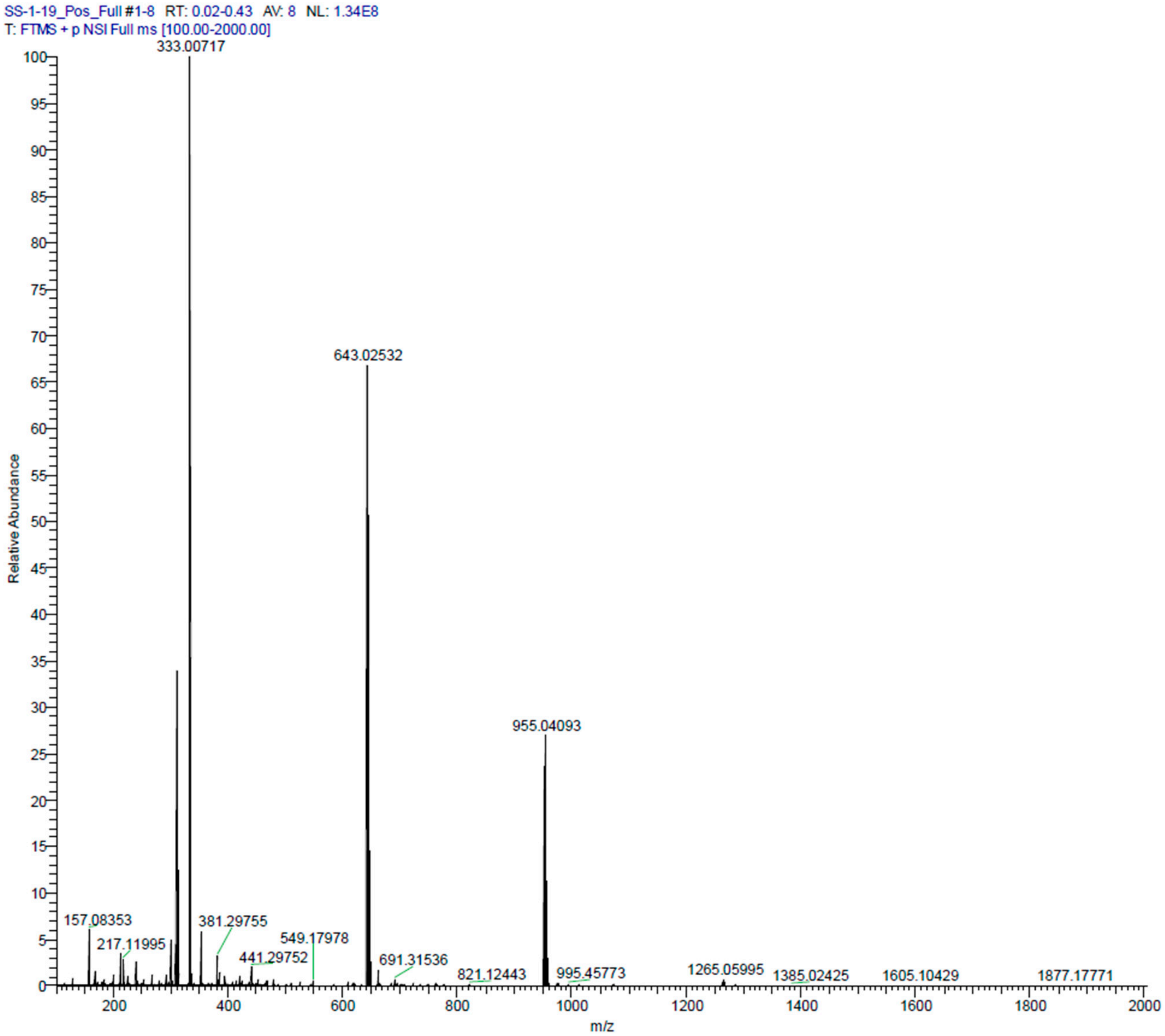

Zoomed spectrum (Top spectrum: measured mass; bottom spectrum: calculated mass)

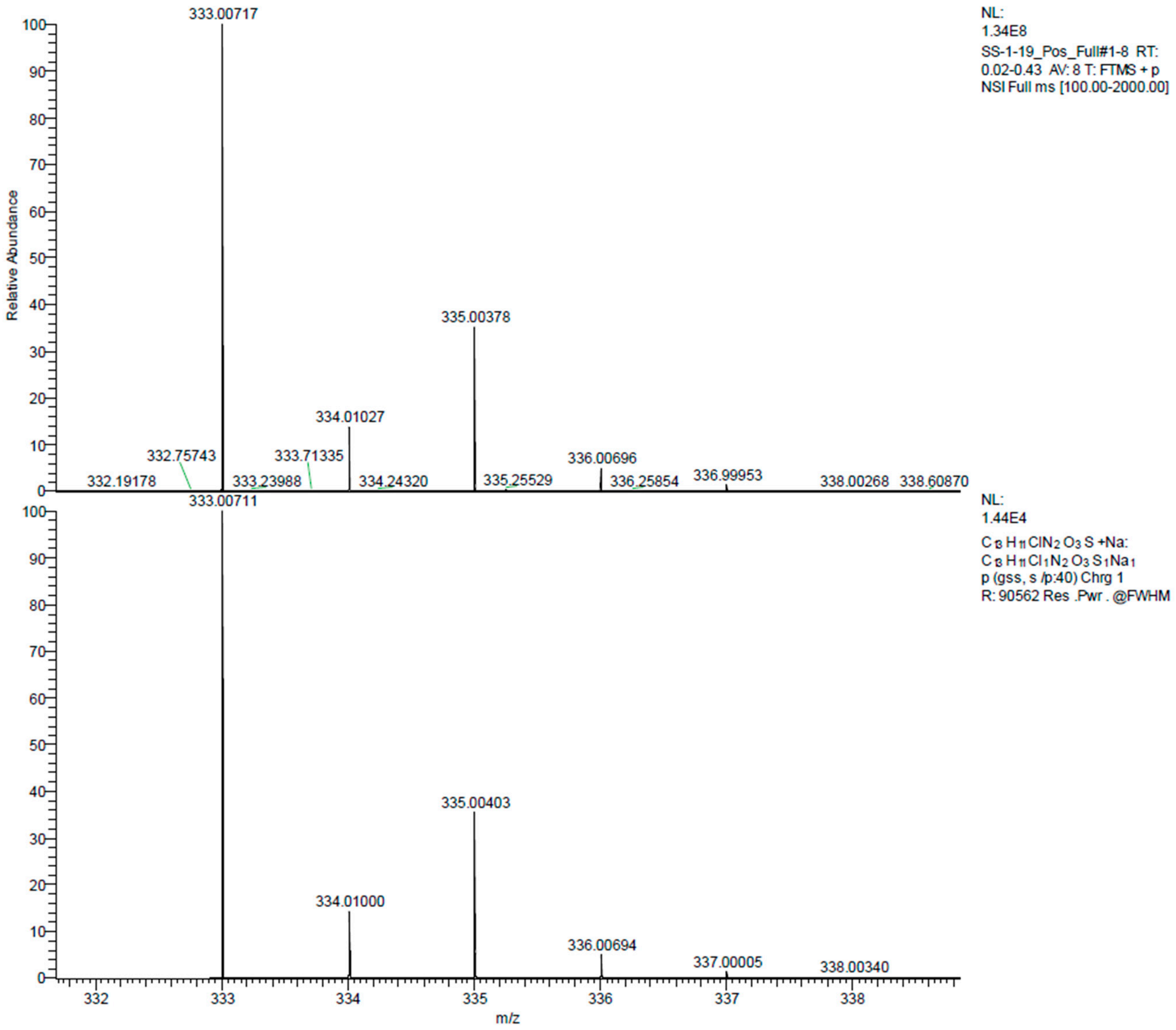

3-((4-Oxo-3-phenyl-3,4-dihydroquinazolin-2-yl)thio)propanoic acid (**9d**)

Full spectrum

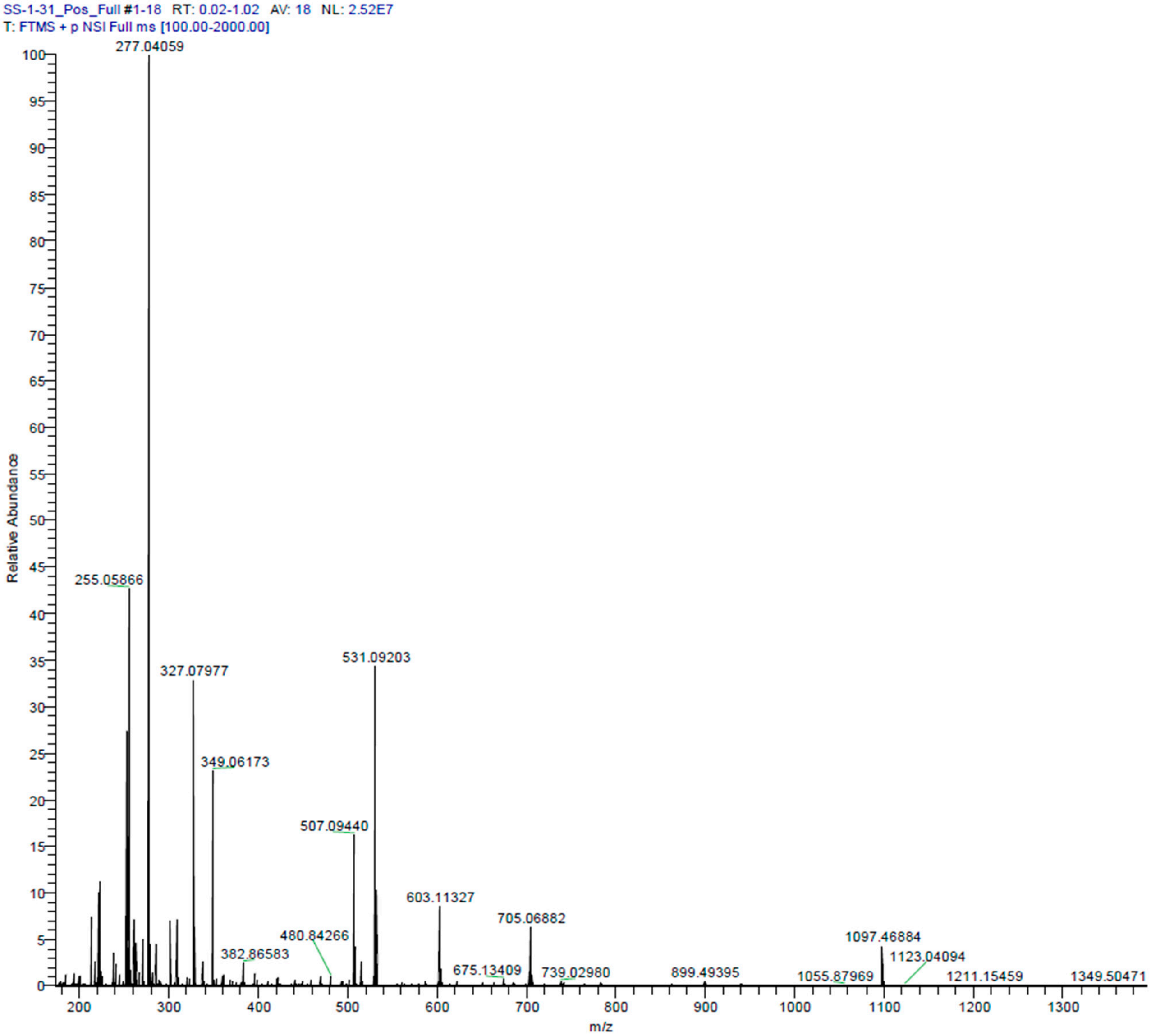

2-Chloro-*N*-(4-methoxyphenyl)acetamide (**12**)

Full spectrum

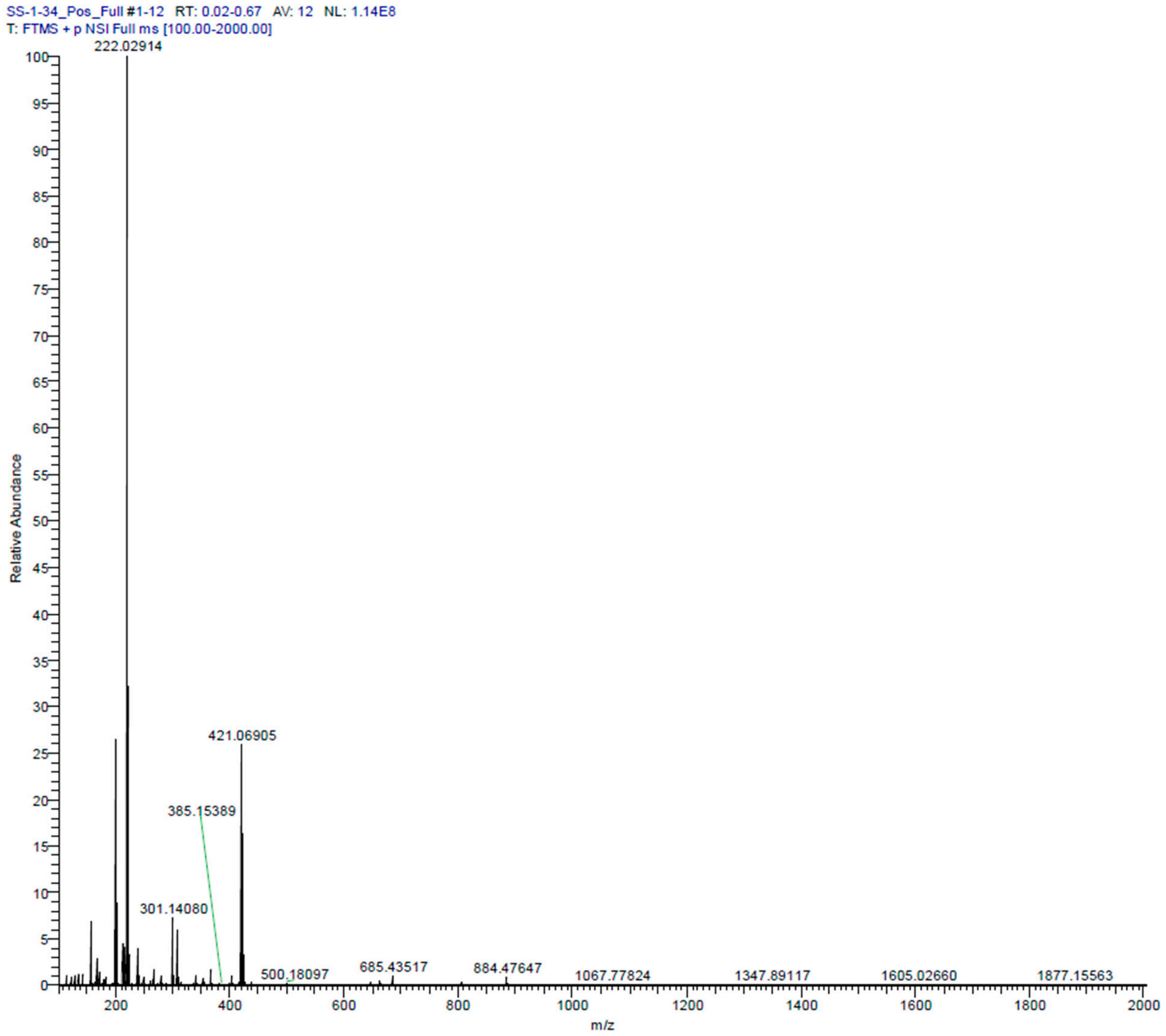

Zoomed spectrum (Top spectrum: measured mass; bottom spectrum: calculated mass)

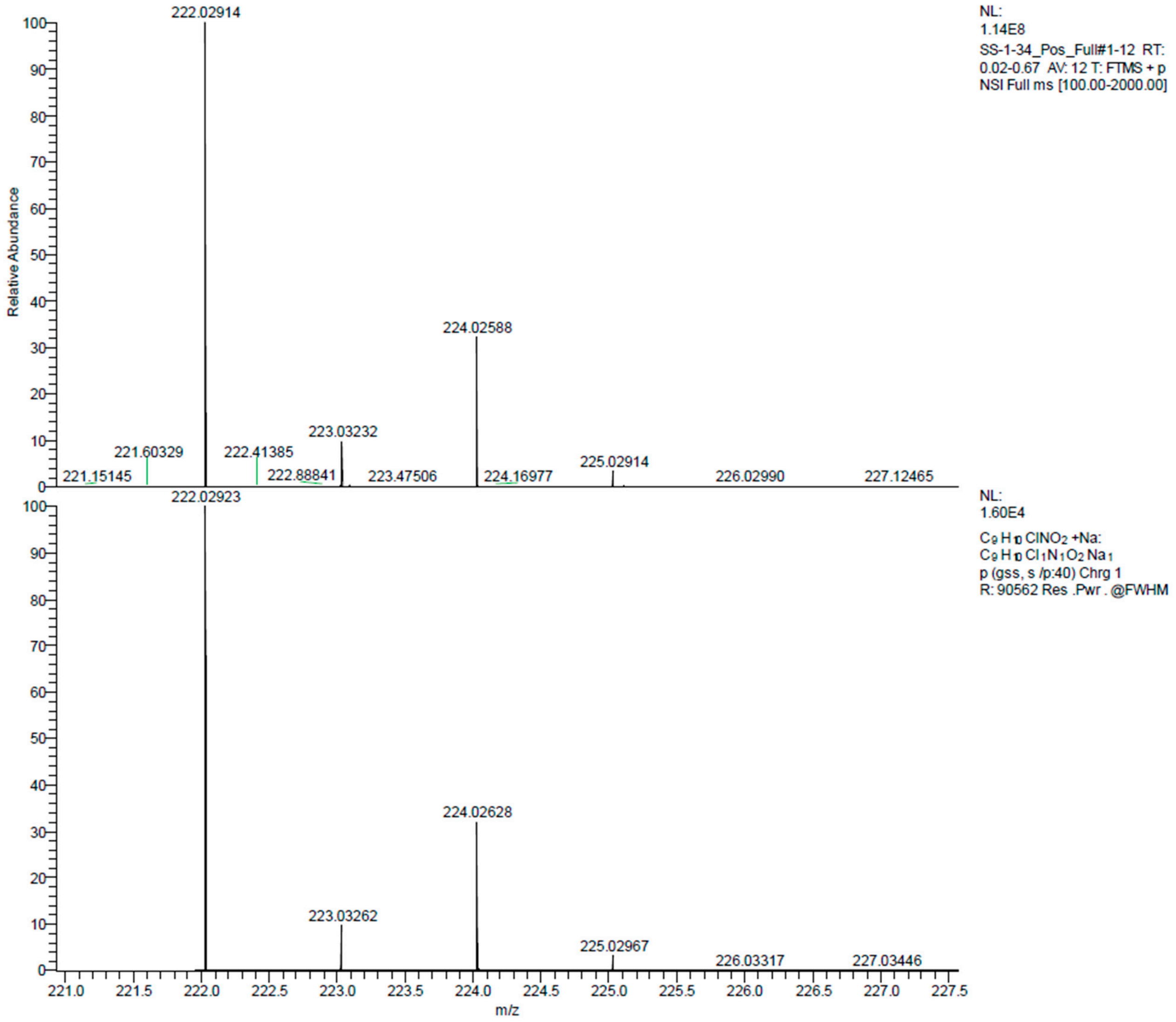

3-Cyclopropyl-2-(prop-2-yn-1-ylthio)quinazolin-4(3*H*)-one (**13a**)

Full spectrum

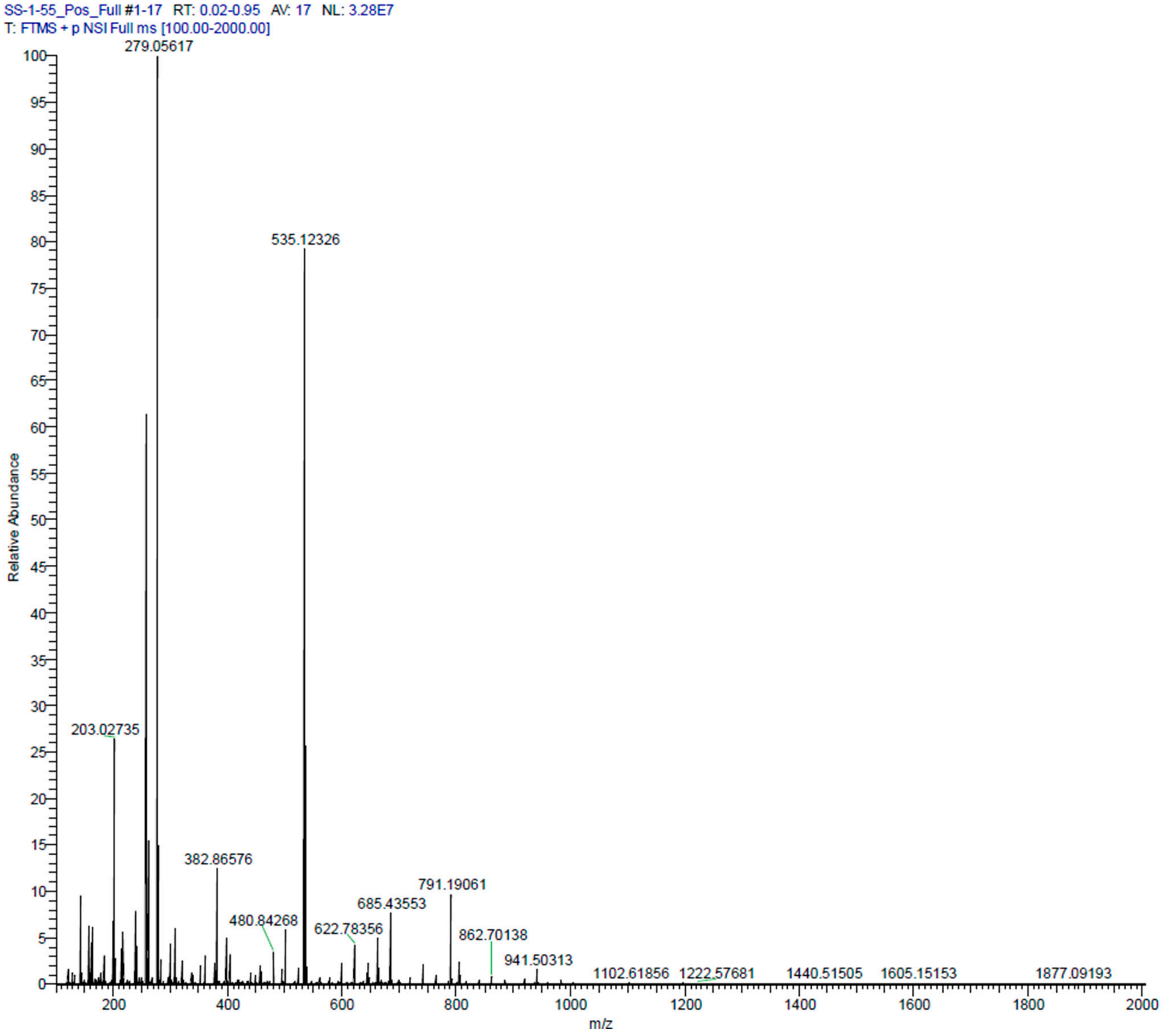

Zoomed spectrum (Top spectrum: measured mass; bottom spectrum: calculated mass)

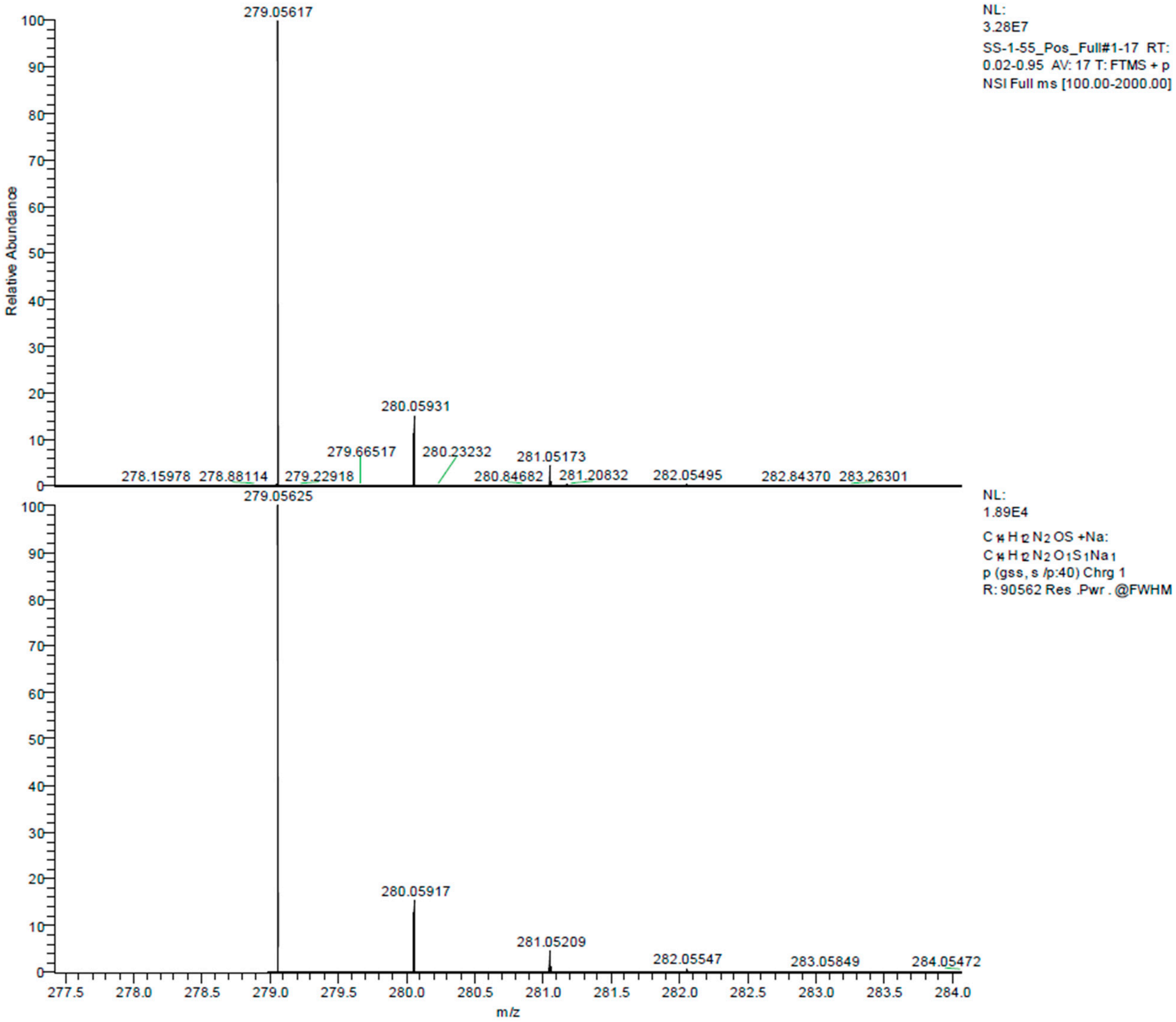

3-Phenyl-2-(prop-2-yn-1-ylthio)quinazolin-4(3*H*)-one (**13b**)

Full spectrum

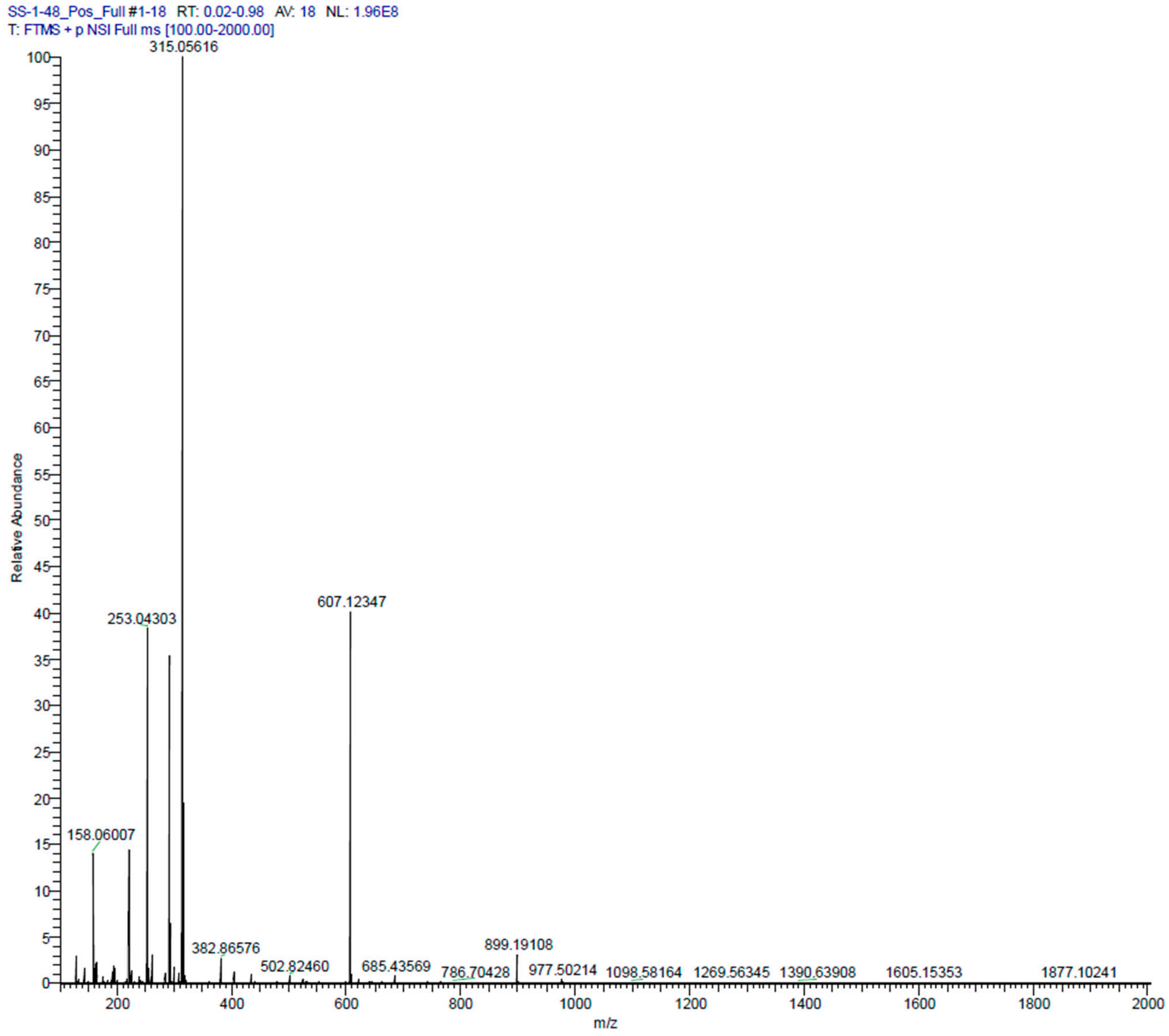

Zoomed spectrum (Top spectrum: measured mass; bottom spectrum: calculated mass)

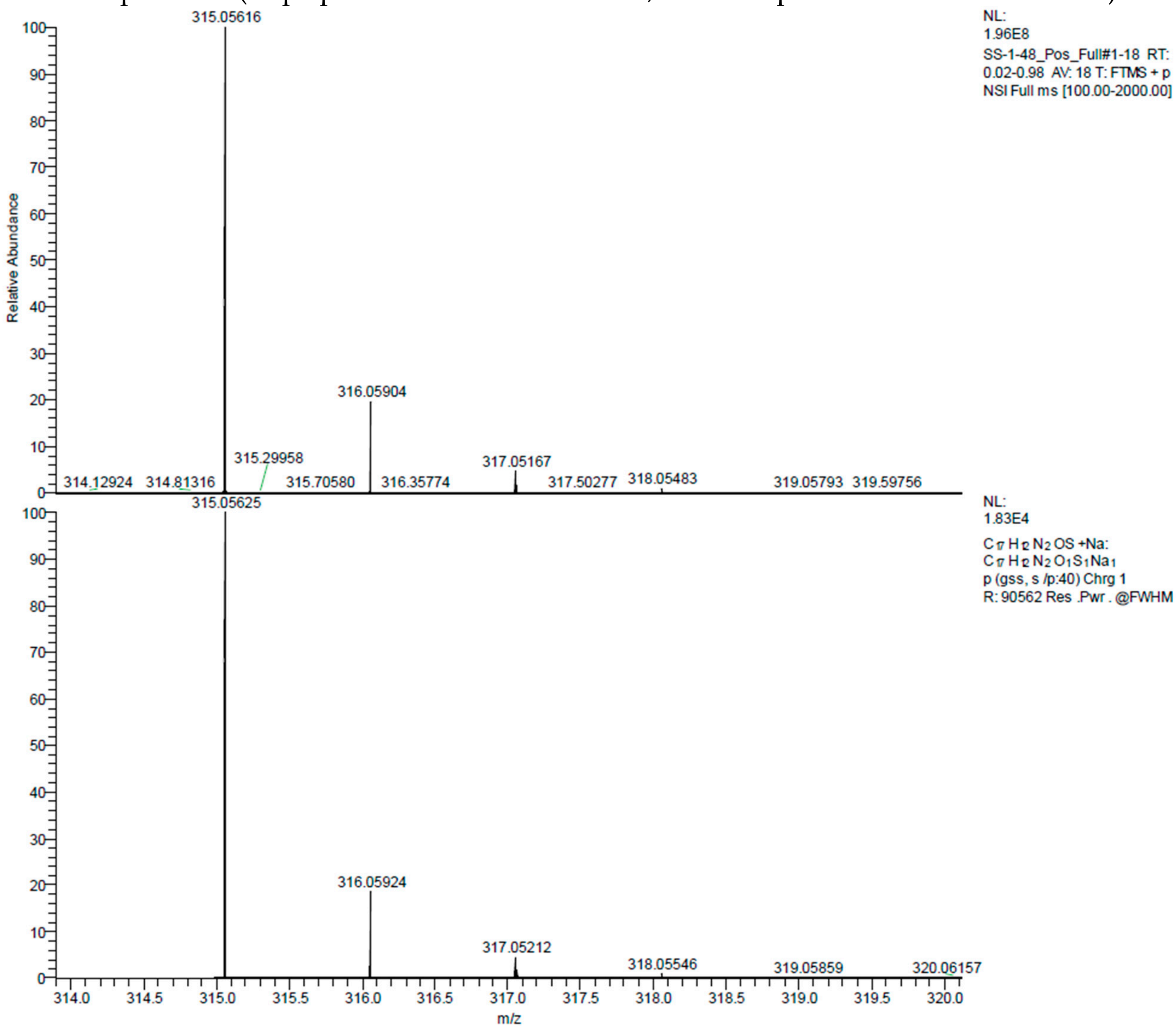

6-Chloro-3-cyclopropyl-2-(prop-2-yn-1-ylthio)quinazolin-4(3*H*)-one (**13c**)

Full spectrum

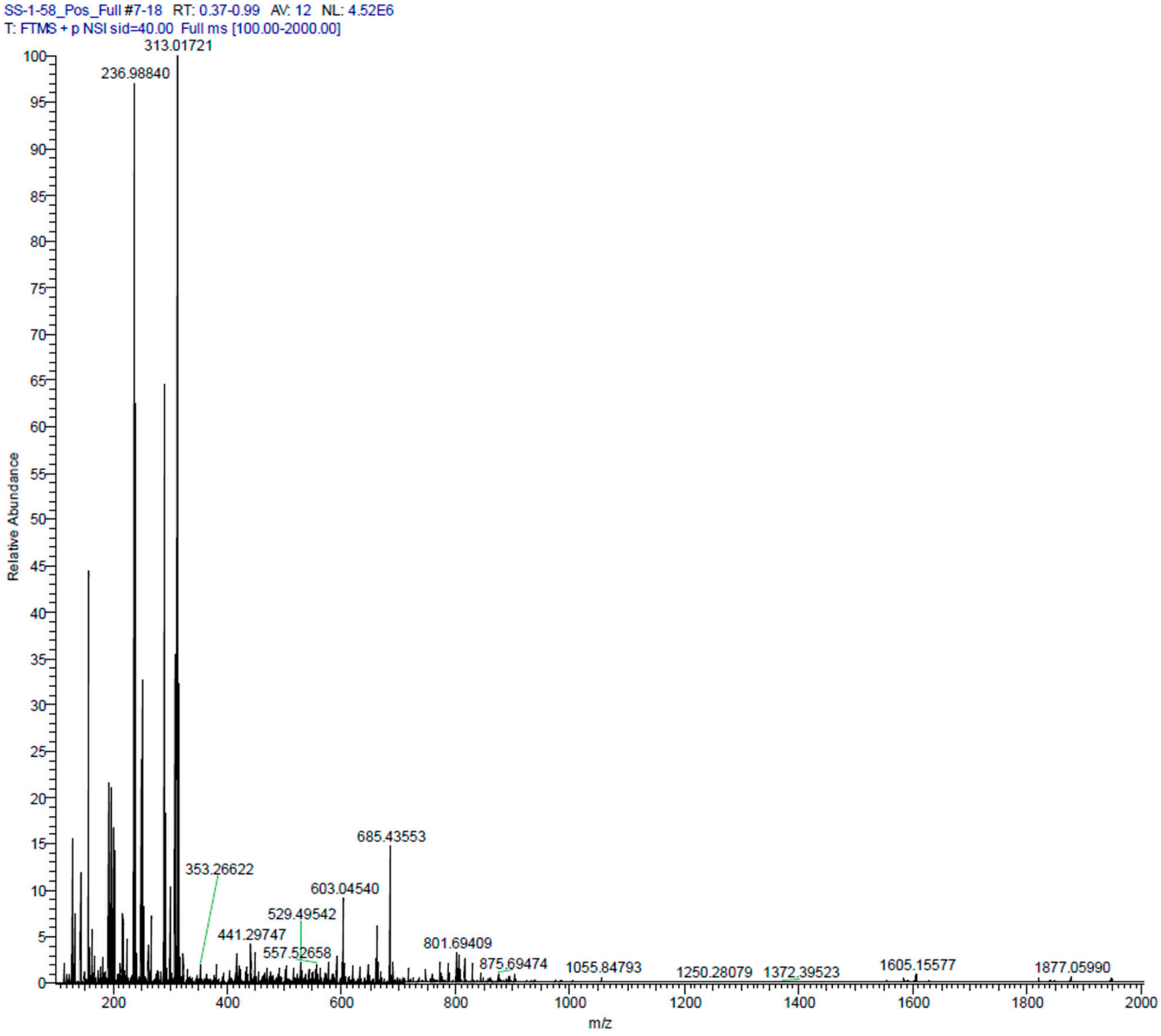

Zoomed spectrum (Top spectrum: measured mass; bottom spectrum: calculated mass)

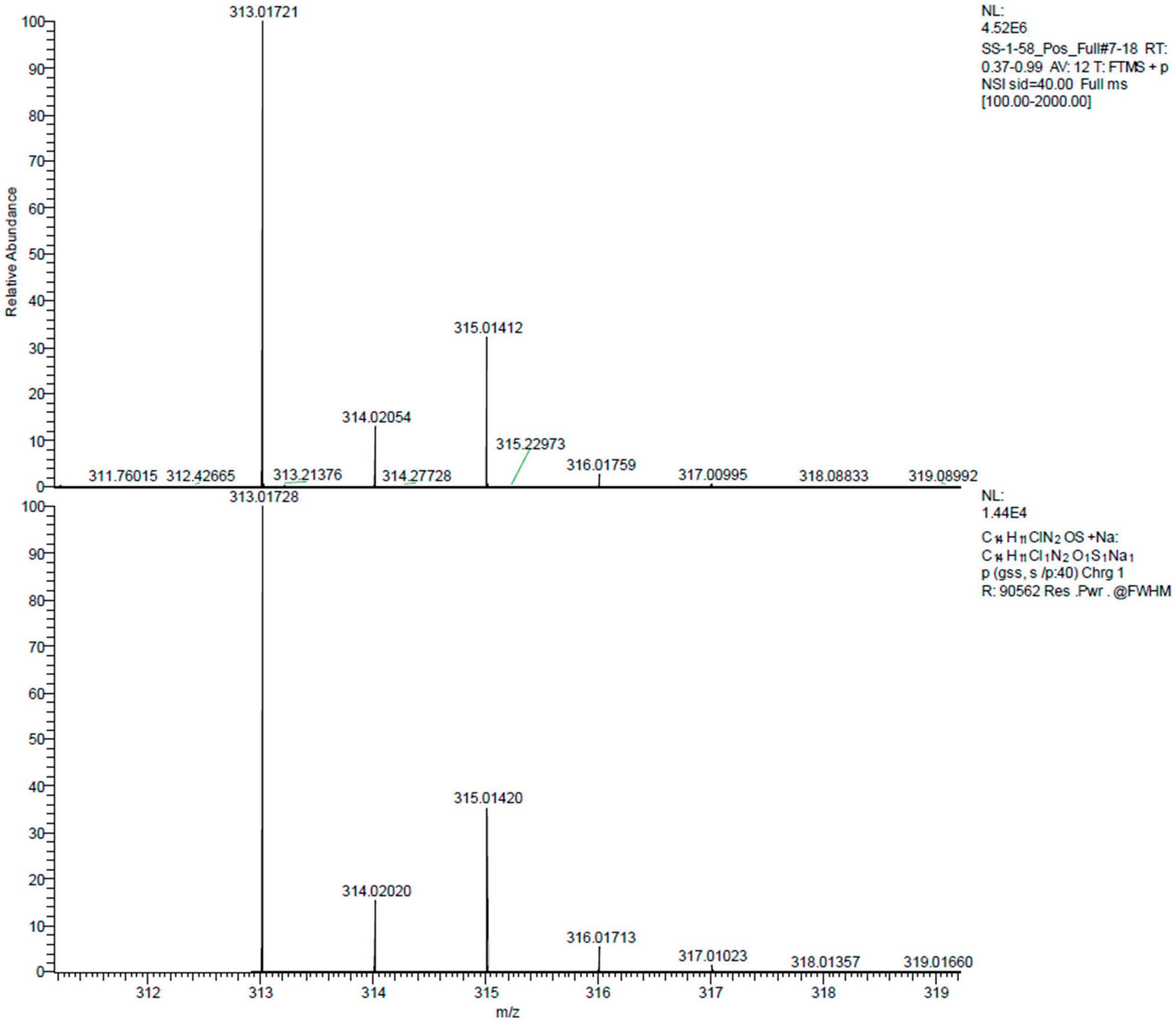

2-Bromo-*N*-(4-methoxyphenyl)acetamide (**15a**)

Full spectrum

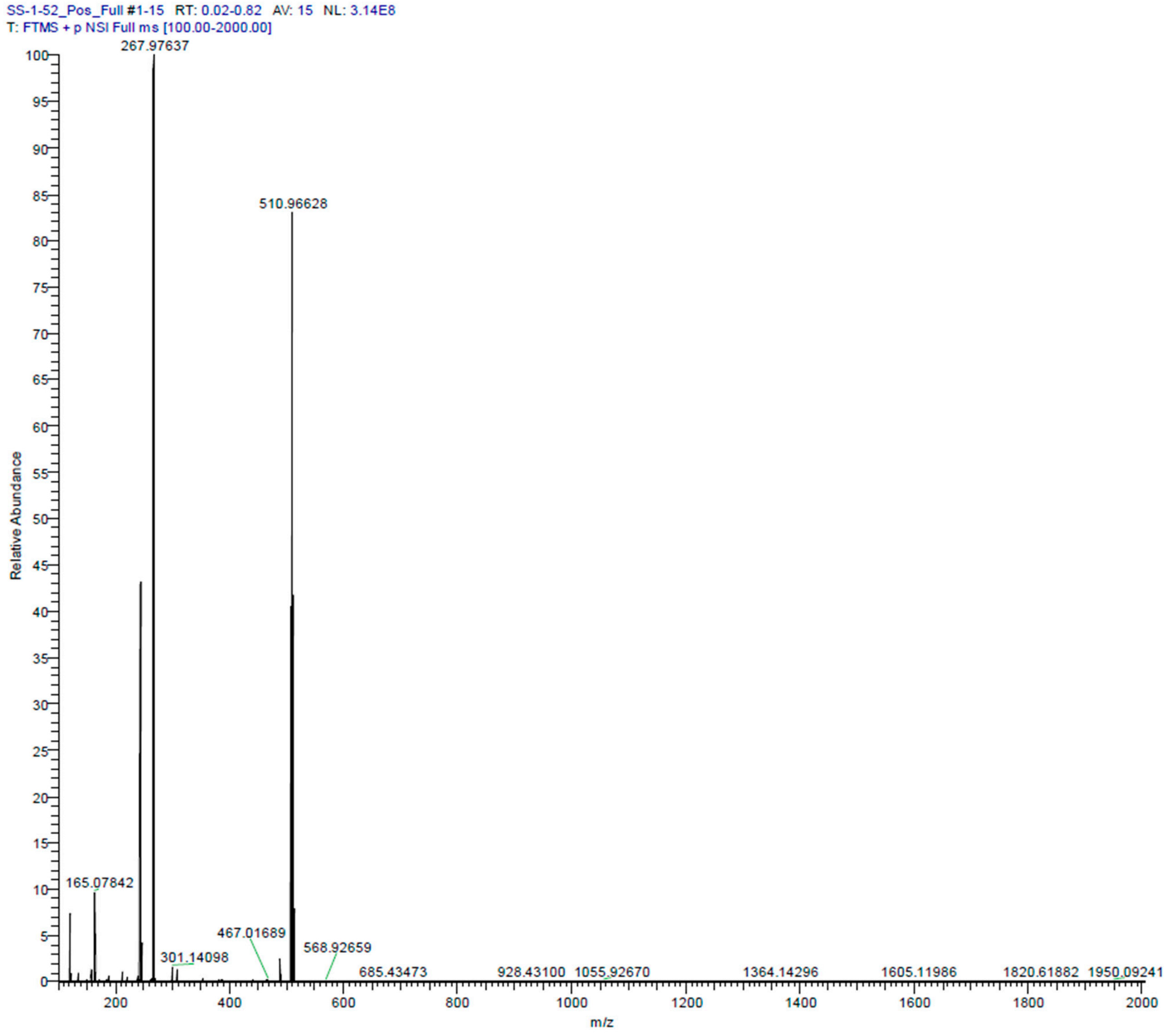

Zoomed spectrum (Top spectrum: measured mass; bottom spectrum: calculated mass)

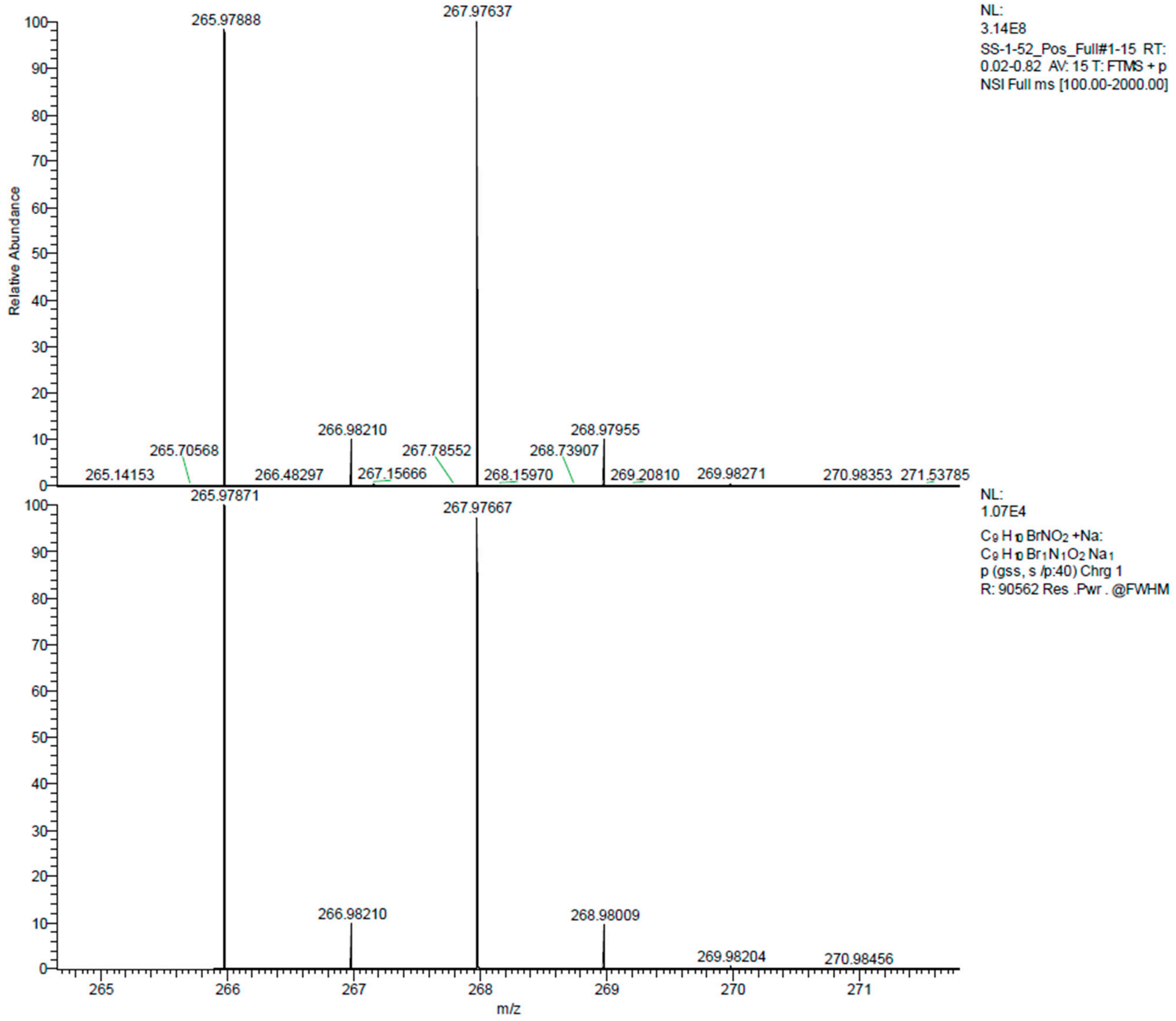

2-Bromo-*N*-phenylacetamide (**15b**)

Full spectrum

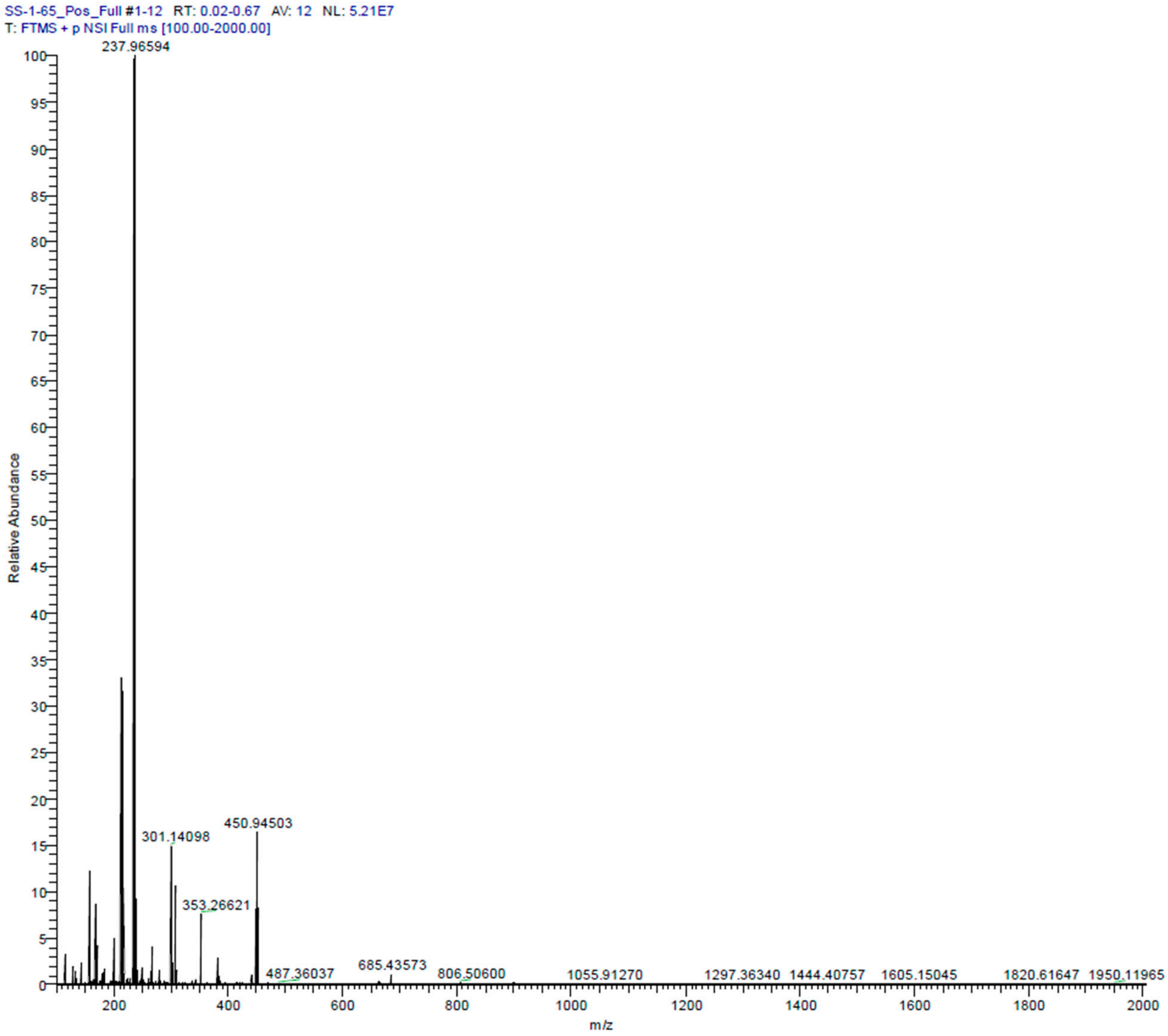

Zoomed spectrum (Top spectrum: measured mass; bottom spectrum: calculated mass)

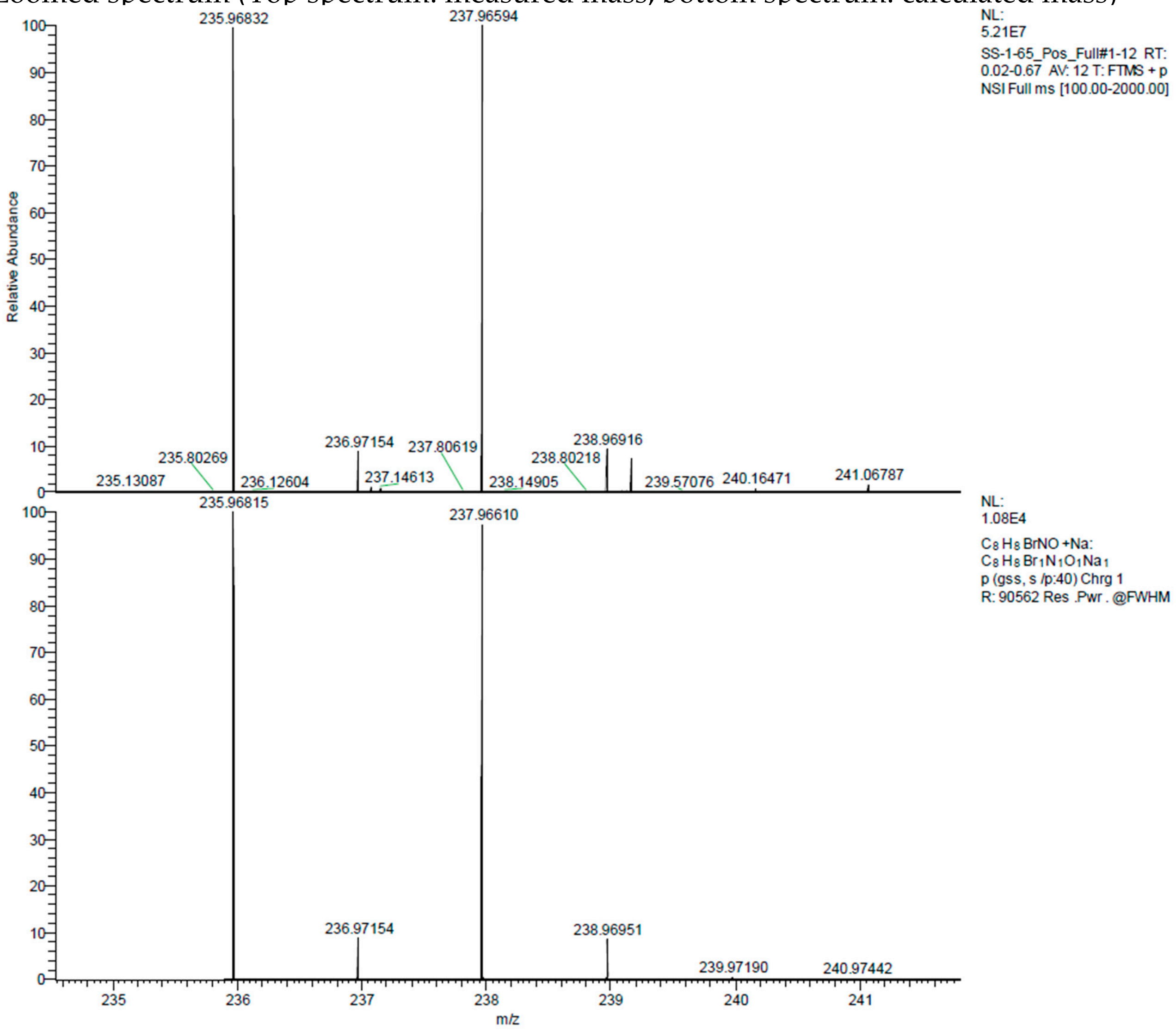

2-Bromo-*N*-(4-nitrophenyl)acetamide (**15c**)

Full spectrum

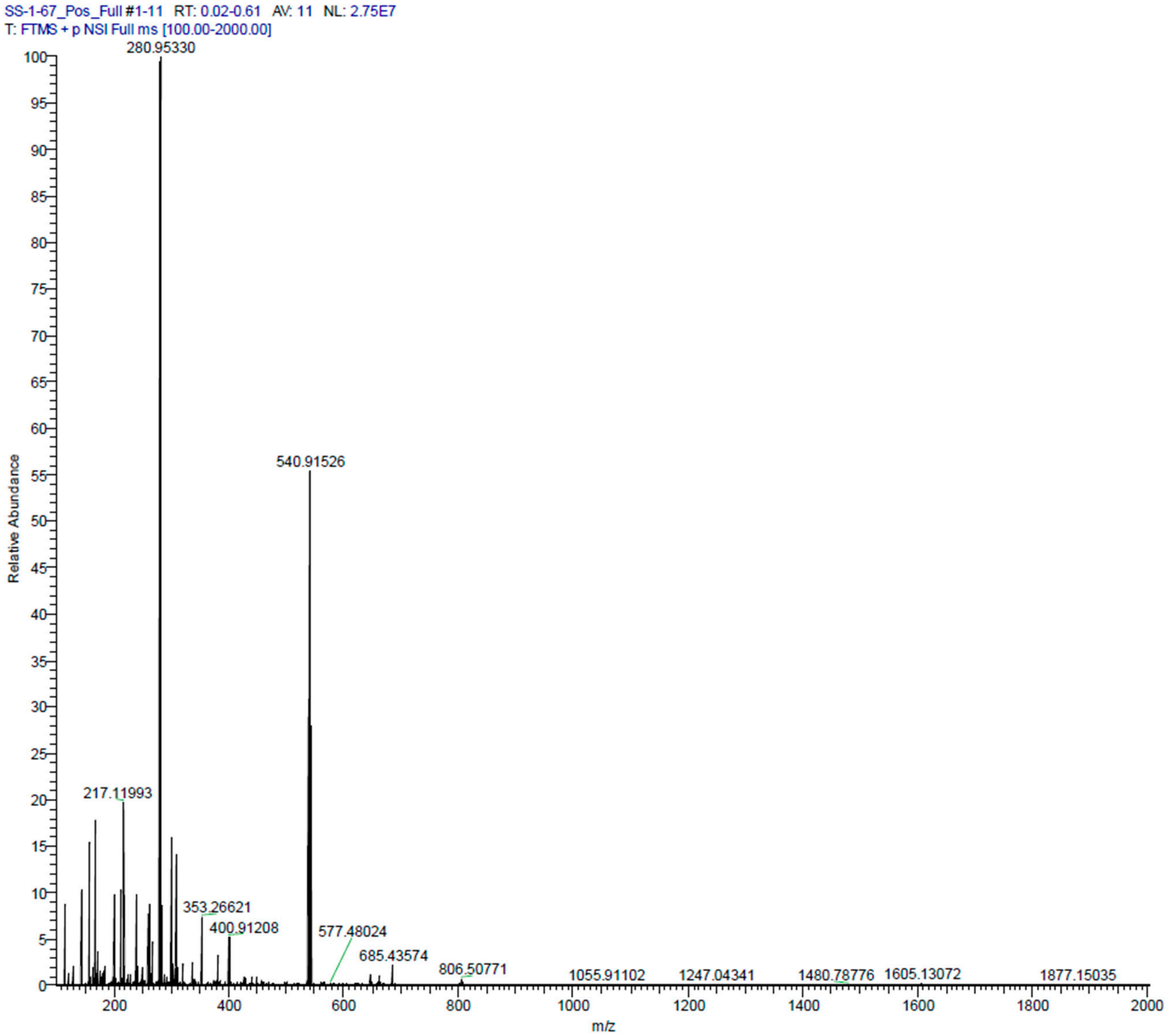

Zoomed spectrum (Top spectrum: measured mass; bottom spectrum: calculated mass)

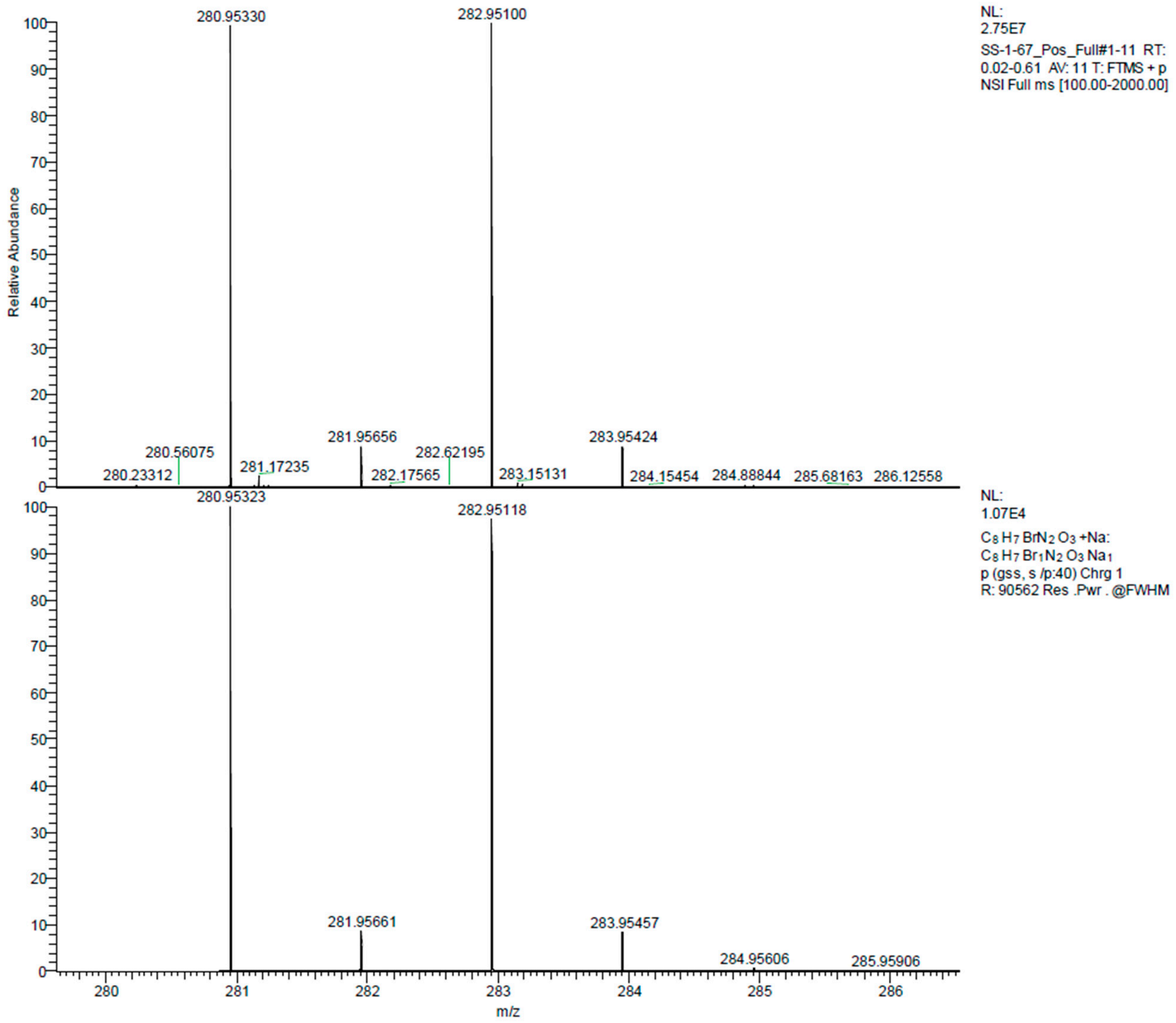

Supplement: Supplementary file 1 [file antibiotics-12-01227-s001.zip › antibiotics-2479769-supplementary.pdf]
